# High-dimensional temporal mapping of CAR T cells reveals phenotypic and functional remodeling during manufacturing

**DOI:** 10.1016/j.ymthe.2025.04.006

**Published:** 2025-05-01

**Authors:** Amaia Cadinanos-Garai, Christian L. Flugel, Anson Cheung, Enzi Jiang, Alix Vaissié, Mohamed Abou-el-Enein

**Affiliations:** 1 USC/CHLA Cell Therapy Program, University of Southern California and Children’s Hospital of Los Angeles, Los Angeles, CA 90033, USA; 2 Berlin Center for Advanced Therapies, Charité-Universitätsmedizin Berlin, Corporate Member of Freie Universität Berlin, Humboldt-Universität zu Berlin, and Berlin Institute of Health, 13353 Berlin, Germany; 3 Division of Medical Oncology, Norris Comprehensive Cancer Center, Keck School of Medicine, University of Southern California, Los Angeles, CA 90033, USA; 4 Department of Stem Cell Biology and Regenerative Medicine, Keck School of Medicine, University of Southern California, Los Angeles, CA 90033, USA; 5 Department of Regulatory and Quality Sciences, Alfred E. Mann School of Pharmacy and Pharmaceutical Sciences, University of Southern California, Los Angeles, CA 90033, USA

**Keywords:** chimeric antigen receptor, CAR T cell therapy, CAR T cell manufacturing, cytotoxicity assay, determinants of response, differentiation, exhaustion, immunoprofiling, phenotypic characterization, proliferation, spectral flow cytometry

## Abstract

Despite the notable success of chimeric antigen receptor (CAR) T cell therapies in hematological malignancies, clinical outcomes remain variable, making it critical to understand how manufacturing influences product composition and function. We developed a 36-marker spectral flow cytometry panel enabling integrated profiling of phenotypic, metabolic, and functional attributes across CAR T cell production. Mid-expansion products (day 5) retained stem-like, metabolically active CD4^+^ Th1 subsets with high proliferative capacity, whereas prolonged culture (day 10) enriched terminally differentiated CD8^+^ Tc1 cells and NK-like T cell populations. CAR^+^ and CAR^−^ T cells showed similar differentiation trajectories, suggesting that culture conditions may have a larger impact on phenotypic remodeling than CAR integration. Upon antigen encounter and restimulation, day 5 and day 10 products showed comparable cytotoxicity, while differing in their activation and checkpoint profiles. Cryopreservation modestly affected stem cell memory, activation, and metabolic markers but preserved overall phenotype and cytotoxic function. These findings establish a high-dimensional framework for mapping CAR T cell dynamics to support manufacturing optimization and next-generation cell therapy design.

## Graphical abstract

**Figure:**
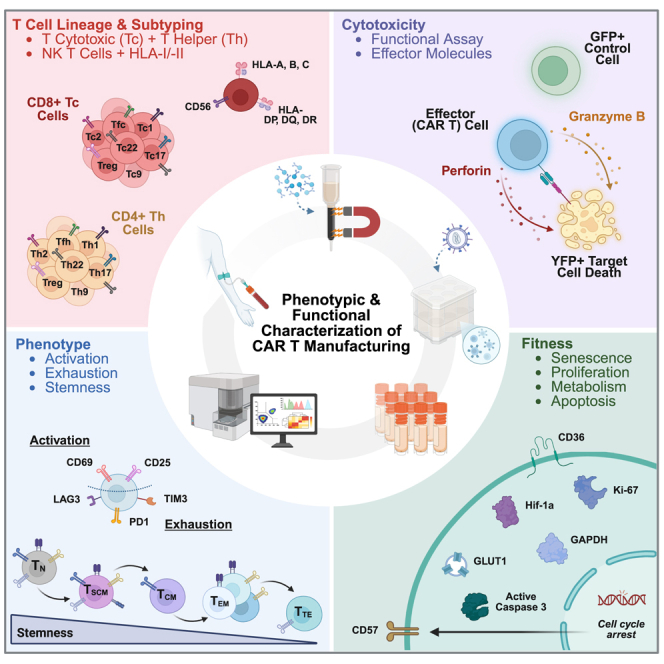

## Teaser

High-dimensional mapping of CAR T cells during manufacturing uncovers distinct trajectories of differentiation and metabolism. These insights reveal critical decision points in culture timing and offer a versatile profiling framework to refine CAR T cell design, improve product consistency, and enhance therapeutic efficacy for next-generation cell therapies.

## Introduction

Chimeric antigen receptor (CAR) T cell therapies have redefined the treatment landscape for hematologic malignancies. In B cell malignancies such as acute lymphoblastic leukemia and diffuse large B cell lymphoma, CD19-targeted CAR T cells induce high remission rates; however, response durability remains inconsistent, with many patients relapsing within 1 year of treatment.^1^^,^^2^^,^^3^^,^^4^^,^^5^ These outcomes are shaped by a complex interplay of tumor-intrinsic mechanisms, T cell fitness, and manufacturing variables. Mechanisms such as antigen loss facilitate immune evasion, ultimately undermining the durability of remission.^6^ Likewise, T cell-intrinsic dysfunctions, particularly exhaustion and loss of stemness, limit CAR T cell potency and compromise long-term persistence.^7^ An often-overlooked contributor to this variability is the potential impact of the CAR T cell manufacturing process. Reports of second primary malignancies following CAR T cell therapy further stress the need to better understand how manufacturing processes influence T cell fate, particularly in cases involving CAR⁺ T cell lymphomas.^8^

All US Food and Drug Administration-approved CAR T cell therapies rely on *ex vivo* expansion of autologous T cells, which are inherently heterogeneous and vary substantially in phenotype and composition across individuals.^9^^,^^10^ Donor-specific factors, including age, gender, comorbidities, and prior treatments, further shape the immunological profile of the T cells used for manufacturing.^11^^,^^12^ Emerging evidence suggests that these variations contribute to disparate clinical outcomes.^13^^,^^14^^,^^15^ For instance, CAR T cell products enriched in memory-like T cells tend to persist longer and mediate superior antitumor responses,^15^^,^^16^^,^^17^ while an abundance of exhausted T cells correlates with poor persistence and early relapse.^13^^,^^18^ Similarly, the presence of suppressive regulatory T cells (Tregs) in a CAR T cell product may impair antitumor efficacy.^19^ Despite these insights, defining the optimal phenotypic and functional profile of a CAR T cell product remains a major challenge.

Standard quality control assays for CAR T manufacturing, such as CAR expression, CD4:CD8 ratios, and *in vitro* cytotoxicity offer only a limited snapshot of the final product.^20^^,^^21^ While sufficient for regulatory release, these measures do not capture key attributes of CAR T cells such as differentiation state, checkpoint expression, or metabolic activity, which evolve throughout manufacturing and shape early functional responses.^14^ Although several studies have provided valuable insights into determinants of response using patient-derived CAR T cells, most have focused on final product samples, with limited evaluation of how these features evolve throughout manufacturing.^15^^,^^16^^,^^17^ As a result, little is known about when CAR T cells acquire peak functional attributes during expansion or how key phenotypic programs are modulated across distinct manufacturing stages. This lack of high-dimensional temporal resolution limits our ability to detect critical differences between products and represents a missed opportunity to link cellular dynamics with therapeutic efficacy.

To address these gaps, we developed and applied a 36-marker spectral flow cytometry panel to profile CAR T cells across the manufacturing timeline and in response to antigen stimulation. Designed as an integrated single assay, it simultaneously captures high-dimensional immunophenotyping and *in vitro* cytotoxicity, generating a detailed fingerprint of the CAR T cell product. This approach enables us to reveal key biological transitions that may inform manufacturing optimization and guide product design.

## Results

**Figure 1:**
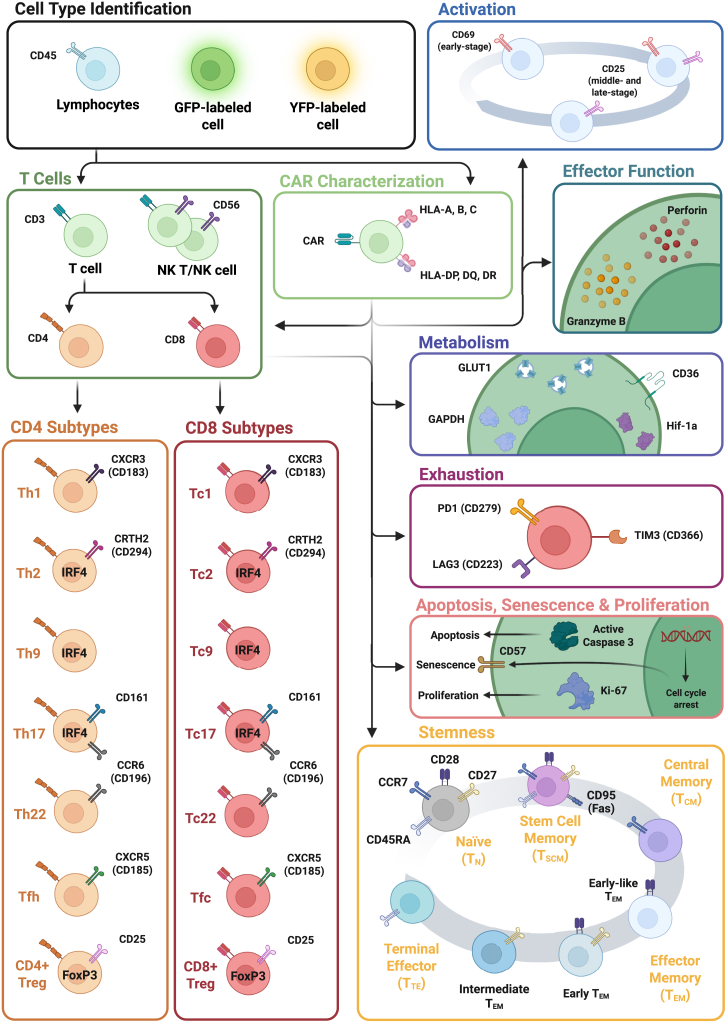
Overview of CAR T cell phenotyping framework for panel design Schematic representation of the 36-marker spectral flow cytometry panel used to profile anti-CD19 CAR T cells. The panel is organized by biological function and phenotypic relevance, encompassing key modules for cell identification, CAR characterization, lineage assignment, functional state, and differentiation status. CD3, CD4, and CD8 distinguish T cell subsets; CD56 identifies NK/NK-like T cells. YFP and GFP reporters are used to track CD19⁺ Nalm6-YFP⁺ target cells and CD19⁻ Nalm6-GFP⁺ control cells in co-culture and restimulation assays. CAR expression is detected alongside HLA-A, B, C and HLA-DP, DQ, DR to assess transgene expression and HLA knockout in allogeneic products. Functional states include activation markers (CD69, CD25), effector function mediators (granzyme B, perforin), and metabolic activity (GLUT1, GAPDH, CD36, Hif-1a). Exhaustion (PD1, LAG3, TIM3), senescence (CD57), proliferation (Ki-67), and apoptosis (active caspase 3) are tracked alongside memory and differentiation states. Naïve (T_N_), stem cell memory (T_SCM_), central memory (T_CM_), effector memory (T_EM_; early-like, early, and intermediate), and terminal effector (T_TE_) subsets are defined by CD45RA, CCR7, CD95, CD27, and CD28. CD4^+^ and CD8^+^ subsets are further resolved into Th and Tc lineages, including Th1/Tc1, Th2/Tc2, Th9/Tc9, Th17/Tc17, Th22/Tc22, Tfh/Tfc, and Tregs.

The 36-marker spectral flow cytometry panel provided a comprehensive view of CAR T cell phenotypic evolution from starting donor material through genetic engineering, expansion, and functional testing (Figure 1). Table S1 summarizes the panel design and antibody specifications.

### Longitudinal profiling of CAR T cell manufacturing

**Figure 2:**
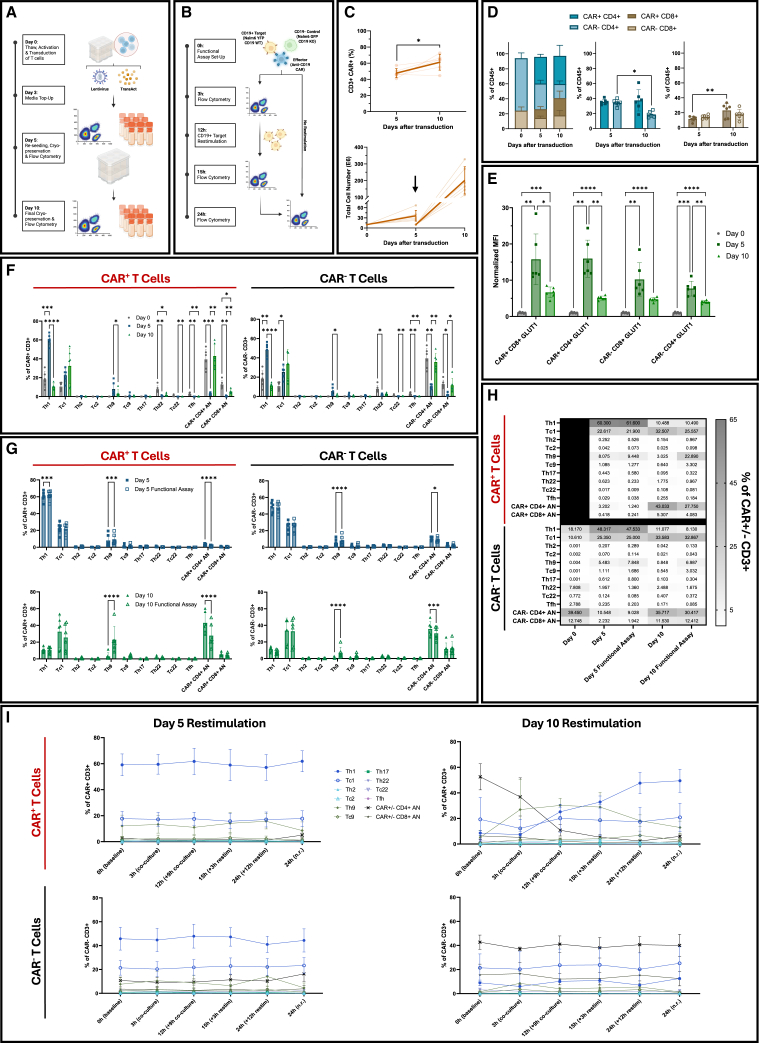
Experimental design and CAR T cell phenotypic profiling (A) Schematic of the manufacturing workflow. T cells from healthy donors were activated and transduced with anti-CD19 CAR on day 0 and harvested on days 5 and 10 for spectral flow cytometry and functional assays. (B) Functional assays included a 3 h co-culture with CD19^+^ Nalm6-YFP^+^ target and CD19^−^ Nalm6-GFP^+^ control cells at days 5 and 10, and a restimulation assay in which fresh CD19^+^ targets were added at 12 h (9 h post-initial co-culture), followed by sample collection at 15 h (3 h post-restimulation) and 24 h. (C) Top: percentage of CD3^+^CAR^+^ expression at day 5 and day 10 post-transduction, shown out of CD45^+^ population (*n* = 6 donors, 2 independent experiments). Bold line represents mean ± SD. Bottom: total cell yield at days 5 and 10. Bold arrow highlights day 5 sample harvest and re-seeding (*n* = 6 donors, 2 independent experiments). (D) Proportion of CD4^+^ and CD8^+^ cells of CAR^+^ and CAR^−^ T cells (*n* = 6 donors, 2 independent experiments). (E) Normalized median fluorescence intensity (MFI) of GLUT1 in CAR^+^ and CAR^−^ CD4^+^ and CD8^+^ subsets at days 0, 5, and 10 (*n* = 6 donors, 2 independent experiments). MFI were normalized to the average of day 0 MFI. Day 0 CAR^−^ samples served as the baseline reference. (F) Frequencies of CD4^+^ helper (Th) and CD8^+^ cytotoxic (Tc) subsets in CAR^+^ and CAR^−^ populations throughout manufacturing (*n* = 6 donors, 2 independent experiments). Day 0 CAR^−^ samples served as the baseline reference. Only subsets representing ≥0.5% of total CD3^+^ T cells for any time point or donor are shown. (G) Th and Tc subset frequencies following 3 h co-culture with target and control cells on days 5 (top) and 10 (bottom) products (*n* = 6 donors, 2 independent experiments). (H) Heatmap of CD4^+^ and CD8^+^ subset frequencies within CAR^+^ and CAR^−^ compartments across all time points and co-culture conditions. AN, all negative (CXCR3^−^IRF4^−^CRTH2^−^CCR6^−^CXCR5^−^) (*n* = 6 donors, 2 independent experiments). (I) Kinetics of CD4^+^ and CD8^+^ subset frequencies during the 24 h restimulation assay in day 5 and day 10 CAR^+^ (top) and CAR^−^ (bottom) T cells (*n* = 3 donors). n.r., non-restimulated; restim, restimulated. Statistical analyses: paired, two-tailed Student’s t test (C), two-way repeated measures ANOVA with Tukey’s (E, F and I), and Šídák’s (D and G) multiple comparisons test. Data represent mean ± SD; ∗*p* < 0.05; ∗∗*p* < 0.01; ∗∗∗*p* < 0.001; ∗∗∗∗*p* < 0.0001. The absence of *p*-values denotes non-significance at the *p* = 0.05 threshold. All *p-*values for panel I are provided in Table S6.

We generated anti-CD19 CAR T cells from six healthy donors via lentiviral transduction and tracked their phenotypes at day 0 (pre-transduction), day 5 (mid-expansion), and day 10 (final harvest) (Figure 2A). Functional responses were assessed by co-culturing day 5 and day 10 products with CD19⁺ Nalm6 cells expressing yellow fluorescent protein (YFP⁺) as targets and CD19⁻ Nalm6 cells expressing green fluorescent protein (GFP⁺) as controls (Figure 2B). In three donors, CAR T cells underwent an additional antigen rechallenge to assess the effects of repeated stimulation. In this assay, day 5 and day 10 products were exposed to fresh CD19^+^ targets at 12 h, followed by sample collection at 15 h (3 h post-restimulation) and 24 h. Parallel wells without a second stimulation served as non-restimulated controls (Figure 2B).

#### Proliferative and metabolic features define CD4:CD8 dynamics in CAR T cell expansion

Transduction and expansion were robust in all donors, with efficient CAR expression by day 5 and sustained viability (Figures 2C and S1A). At this time point, 40.9%–57.3% of T cells were CAR^+^, increasing to 44.6%–72.6% by day 10 (*p <* 0.05; Figure 2C). CAR density was high at day 5 (median fluorescence intensity [MFI] ∼23,300), with a modest decrease by day 10 (Figure S1B). CD4^+^:CD8^+^ ratios remained largely unchanged between days 0 and 5 (Figure 2D). By day 10, CAR^+^CD8^+^ cells were selectively enriched (*p* < 0.01), while the CAR^−^CD4^+^ population contracted (*p <* 0.05; Figure 2D), reflecting divergent expansion dynamics between transduced and non-transduced subsets. CAR^+^ T cells expressed higher levels of GLUT1 than CAR^−^ cells at day 5, indicating elevated glycolytic activity (Figure 2E). A subset of CAR^−^CD4^+^ cells at day 5 lacked Ki-67 expression, suggesting a non-proliferative phenotype that may contribute to the relative contraction of this compartment by day 10 (Figure S1C). This is consistent with the reduced GLUT1 expression in CAR^−^CD4^+^ cells. While GLUT1 expression in CAR^+^ cells declined by day 10, levels remained elevated compared to day 0, indicating sustained metabolic activity (Figure 2E).

#### Extended CAR T cell expansion promotes a shift from Th1 to Tc1-dominant phenotypes

We next examined how T cell subsets evolved during manufacturing. At baseline (day 0), the dominant population lacked lineage-defining markers (“all negative” [AN]: CXCR3^−^IRF4^−^CRTH2^−^CCR6^−^CXCR5^−^), although discrete T helper 1 (Th1), T cytotoxic 1 (Tc1), and T helper 22 (Th22) subsets were present (Figure 2F). By day 5, Th1 cells significantly increased compared to day 0 (*p <* 0.001; Figure 2F), while the AN population declined sharply (*p <* 0.001 for CAR^+^CD4^+^ AN reduction), as more cells expressed defined lineage markers. By day 10, Th1 frequencies returned to baseline (*p <* 0.0001 for day 5 vs. day 10), and the CAR^+^CD4^+^ AN cells rebounded (*p <* 0.01). Tc1 cells showed a gradual and sustained increase, becoming the dominant subset by day 10 (Figure 2F). T helper 9 (Th9) cells, initially present at low frequency, showed a modest rise by day 5, followed by a return to baseline by day 10 (*p <* 0.05, day 5 vs. day 10). These trends were consistent in both CAR^+^ and CAR^−^ populations, indicating that CAR transduction did not skew helper or cytotoxic lineage differentiation. Interferon regulatory factor 4 (IRF4) expression, which was nearly absent at day 0, became significantly upregulated by day 5, particularly within the CAR^+^CD4^+^ subset (*p <* 0.01; Figure S1D). IRF4 upregulation was most prominent in CXCR3^+^ Th1 and Tc1 cells (Figure S1F), suggesting a role in metabolic or proliferative support of these expanding subsets.

#### Cytotoxic challenge induces minimal shifts in CAR T cell subtype composition

Day 5 and day 10 products were co-cultured with CD19^+^ targets and CD19^−^ controls for 3 h, followed by phenotypic analysis. Despite robust functional activation, overall subset composition remained similar to the pre-assay state (Figure 2G). In day 5 CAR T cells, Th and Tc subset levels were largely unchanged following antigen exposure. Th1 and Tc1 cells remained the predominant subsets in day 5 and day 10 products, respectively (Figure 2H). One notable change was a significant increase in Th9 cells in the day 10 product (*p <* 0.0001 for 0 h vs. 3 h), consistent with IRF4 upregulation upon antigen exposure (Figure S1E). Overall, brief target engagement triggered effector functions while preserving the original T cell subtype distribution, indicating that short-term antigen encounter does not alter lineage composition.

#### Extended antigen exposure triggers subtype reprogramming in CAR T cells

To assess whether repeated antigen exposure induces phenotypic changes, day 5 and day 10 CAR T cells were rechallenged with fresh CD19^+^ targets at 12 h and analyzed at 15 h (3 h post-restimulation) and 24 h (*n* = 3 donors). In day 5 CAR T cells, subset frequencies remained relatively stable, with a persistent Th1-dominant profile (Figures 2I, left, and S2A). Tc1 and Th9 subsets contributed to a lesser extent. In contrast, day 10 CAR T cells exhibited more dynamic changes, including progressive enrichment of Th1 cells and maintenance of the Tc1 compartment (Figures 2I, right, and S2A). Th1 frequency gradually increased, ultimately reaching levels nearing those observed in day 5 cells (*p* < 0.05, 3 h vs. 12 h/15 h). In parallel, Th9 cells exhibited a transient peak (*p* < 0.0001, 0 h vs. 12 h), followed by a decline after 15 h. These shifts were accompanied by a progressive reduction in the CD4⁺ AN population starting at 3 h co-culture through 24 h (Figure 2I, right, and S2A). Phenotypic shifts were most pronounced in the CAR^+^ fraction and remained comparable between restimulated and non-restimulated cells at 24 h.

#### Stem-like memory peaks mid-expansion, followed by terminal effector enrichment

**Figure 3:**
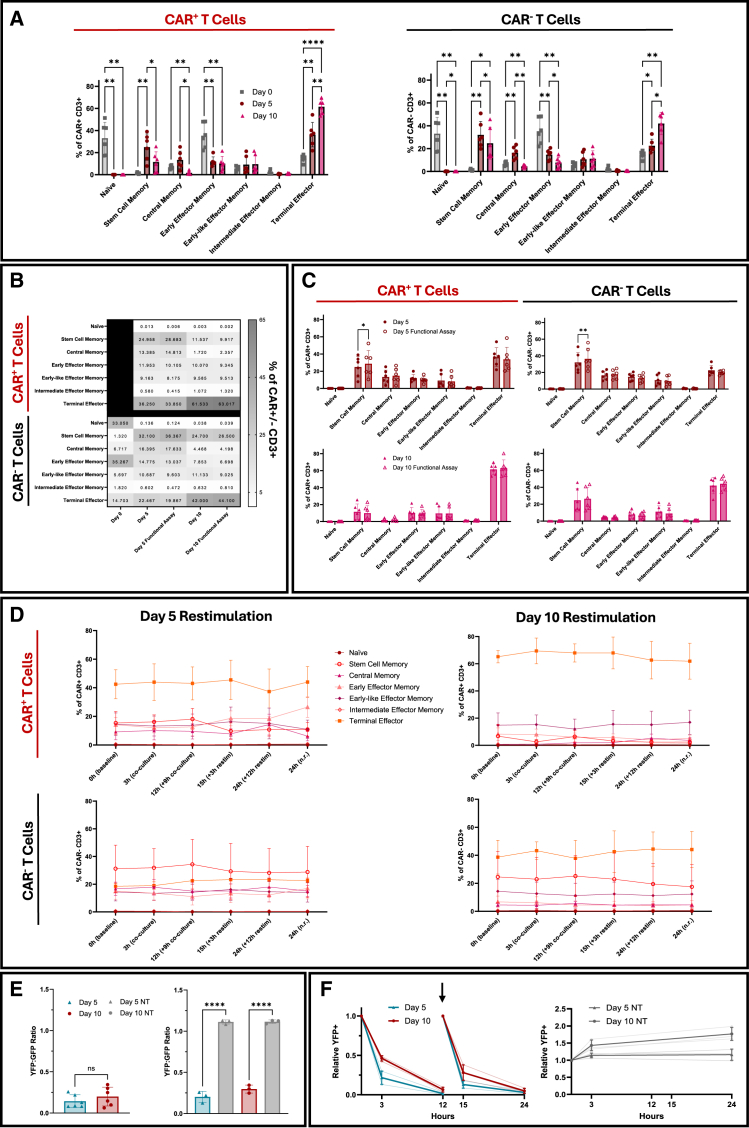
Differentiation trajectories and cytotoxic activity of CAR T cells during manufacturing (A) Frequencies of T_N_, T_SCM_, T_CM_, T_EM_, and T_TE_ subsets within CAR^+^ and CAR^−^ CD3^+^ T cells over the course of manufacturing (days 0, 5, and 10). Day 0 CAR^−^ samples served as the baseline reference (*n* = 6 donors, 2 independent experiments). (B) Heatmap showing the frequency of each differentiation subset throughout manufacturing stages and 3 h co-culture assay (*n* = 6 donors, 2 independent experiments). (C) Differentiation profile following 3 h co-culture with CD19^+^ Nalm6-YFP^+^ target and CD19^−^ Nalm6-GFP^+^ control cells at days 5 and 10 (*n* = 6 donors, 2 independent experiments). (D) Differentiation dynamics in day 5 and day 10 CAR^+^ and CAR^−^ T cells during the 24 h restimulation assay (0, 3, 12, 15, and 24 h; *n* = 3 donors). (E) YFP:GFP ratios from 3 h co-culture assays measuring cytotoxicity of CAR transduced cells (left, *n* = 6 donors) and CAR transduced versus non-transduced cells (right, n = 3 donors). (F) Left: time course of day 5 and day 10 CAR^+^ T cell-mediated killing during restimulation, with antigen additions at 0 and 12 h (*n* = 3 donors). Black arrow represents restimulation time point. Right: normalized CD19^+^ Nalm6-YFP^+^ target cell frequency during restimulation with non-transduced control cells (*n* = 3 donors). restim, restimulated. n.r., non-restimulated. Statistical analyses: two-way repeated measures ANOVA with Tukey’s (A and D), and Šídák’s (C) multiple comparisons test, two-tailed Mann-Whitney *U* test (E, left), and one-way ANOVA with Šídák’s multiple comparisons test (E, right). Data shown as mean ± SD; ∗*p* < 0.05; ∗∗*p* < 0.01; ∗∗∗∗*p* < 0.0001; ns, not significant. The absence of *p*-values denotes non-significance at the *p* = 0.05 threshold. All *p-*values for panel D are provided in Table S6.

We next evaluated how CAR T cell memory states evolved over time and in response to antigen encounter. Using CD45RA, CCR7, CD95, CD27, and CD28 expression, we observed a progressive shift from naïve and early effector memory subsets toward more differentiated phenotypes over the 10-day expansion period (Figures 3A and 3B). The naïve population (33.1% at day 0) declined significantly and fell below 0.1% of CAR^+^ cells by day 5, consistent with activation-induced differentiation (Figure 3B). As naïve cells contracted, T stem cell memory (T_SCM_; CD45RA^+^CCR7^+^CD27^+^CD28^+^CD95^+^) increased to 25.0% of CAR^+^ cells by day 5 (*p <* 0.01, day 0 vs. day 5), with 11.5% persisting at day 10, a subset associated with long-term CAR T cells’ persistence.^22^ Early effector memory cells (T_EM_; CD45RA^−^CCR7^−^CD27^+^CD28^+^) made up 35.3% at day 0, decreased by day 5, and remained stable thereafter. Central memory cells (T_CM_; CD45RA^−^CCR7^+^) peaked at day 5 and declined to 1.7% by day 10 (*p <* 0.05, day 5 vs. day 10). Terminal effector cells (T_TE_; CD45RA^+^CCR7^-^) expanded from 36.3% at day 5 to 61.5% by day 10 (*p <* 0.01), becoming the predominant population. These patterns were also observed in CAR⁻ T cells, indicating that phenotypic changes were largely driven by shared *in vitro* activation and expansion conditions (Figures 3A and 3B).

Following 3 h co-culture, the memory composition of CAR⁺ and CAR⁻ T cells remained largely unchanged (Figure 3C). In donors that underwent antigen rechallenge (*n* = 3) and extended stimulation for up to 24 h, day 5 CAR T cells maintained a predominantly T_TE_ phenotype at both 15 and 24 h, with only modest increases in T_CM_, early effector, and early-like effector memory subsets (Figures 3D and S2B). T_SCM_ frequency remained stable during the initial 3 h co-culture (15.4% at 0 h vs. 16.2% at 3 h) but declined following additional antigen exposure at 12 h and prolonged incubation to 24 h, regardless of restimulation. In day 5 cultures, T_SCM_ frequencies were consistently higher in CAR^−^ cells (28.2%–34.4%) than in CAR^+^ cells (9.7%–18.1%). Day 10 CAR T cells displayed a more terminally differentiated phenotype at baseline and showed minimal phenotypic change at 15 and 24 h, remaining dominated by T_TE_ cells regardless of restimulation. T_SCM_ frequencies in day 10 CAR^+^ cells were markedly lower (2.3%–6.8%) compared to their CAR^−^ counterparts (17.5%–25.2%), further supporting the notion that CAR signaling and antigen exposure promote differentiation away from stem-like states (Figures 3D and S2B). Although not statistically significant, day 5 CAR T cells exhibited stronger cytotoxicity following both initial stimulation and rechallenge, consistent with the increased presence of less-differentiated subsets such as T_SCM_, T_CM_, and T_EM_ (Figures 3E and 3F).

#### CAR T cell expansion drives robust activation with dynamic checkpoint expression

**Figure 4:**
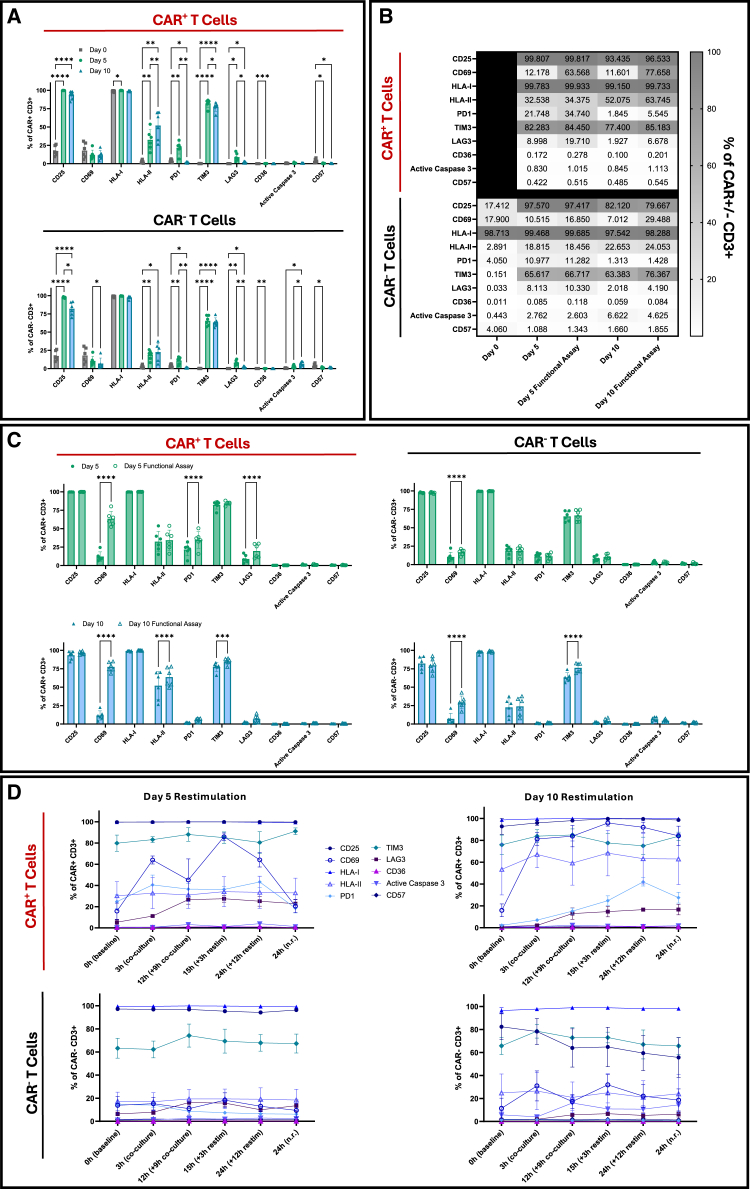
Activation, exhaustion, senescence, and apoptosis marker dynamics during expansion and antigen encounter (A) Baseline expression of CD25, CD69, HLA class I and II, PD1, TIM3, LAG3, CD36, active caspase 3, and CD57 in CAR^+^ and CAR^−^ CD3^+^ T cells on days 0, 5, and 10 (*n* = 6 donors, 2 independent experiments). Day 0 CAR^−^ samples served as the baseline reference. (B) Heatmap visualization of marker expression changes over the manufacturing period and 3 h co-culture assay (*n* = 6 donors, 2 independent experiments). (C) Summary of activation, exhaustion, senescence, and apoptosis marker expression following 3 h co-culture on day 5 and day 10 products (*n* = 6 donors, 2 independent experiments). (D) Longitudinal marker expression during the 24 h restimulation assay in CAR^+^ (top) and CAR^−^ (bottom) CD3^+^ T cells (*n* = 3 donors). n.r., non-restimulated; restim, restimulated. Statistical analyses: two-way repeated measures ANOVA with Tukey’s (A and D), and Šídák’s (C) multiple comparisons test. Data shown as mean ± SD; ∗*p* < 0.05; ∗∗*p* < 0.01; ∗∗∗*p* < 0.001; ∗∗∗∗*p* < 0.0001. The absence of *p*-values denotes non-significance at the *p* = 0.05 threshold. All *p-*values for panel D are provided in Table S7.

We assessed activation, immune checkpoint expression, senescence, and apoptosis markers in CAR T cells throughout manufacturing and in response to antigen exposure. At baseline (day 0), T cells exhibited a non-activated phenotype (Figure 4A). By day 5, both CAR^+^ and CAR^−^ T cells showed robust upregulation of CD25 relative to day 0 (*p <* 0.0001), consistent with strong activation and entry into a proliferative state. High CD25 expression was maintained through day 10, indicating sustained activation during expansion. In contrast, CD69 levels remained low throughout culture. At day 5, 12.2% of CAR^+^ and 10.5% of CAR^−^ cells expressed CD69; by day 10, CD69^+^ frequencies were 11.6% and 7.0%, respectively (Figure 4B). These modest levels reflect the transient nature of CD69 expression following early activation. Human leukocyte antigen (HLA) class I (HLA-A, B, C) expression remained high at all time points. HLA class II (HLA-DP, DQ, DR) expression increased significantly from day 0 to day 10 in both CAR^+^ (*p <* 0.01) and CAR^−^ (*p <* 0.05) cells, consistent with activation-induced upregulation. Notably, CAR^+^ T cells expressed higher levels of HLA class II compared to CAR^−^ T cells (Figure 4A). Among exhaustion markers, TIM3 was the most prominently expressed. While low at day 0, TIM3 became elevated by day 5 (*p <* 0.0001) and remained high through day 10. PD1 and LAG3 showed only transient changes, with modest upregulation by day 5 (PD1: *p <* 0.01; LAG3: *p <* 0.05) that returned to near-baseline by day 10 (Figure 4A). Cell viability remained high throughout, with >90% viability on both days 5 and 10 (Figure S1A). Senescence and apoptosis markers remained low in CAR⁺ T cells, with CD57⁺ cells at 0.4% on day 5 and 0.5% on day 10, and active caspase 3⁺ cells consistently low at 0.8% across both timepoints (Figures 4A and 4B). By day 10, CAR⁻ T cells showed slightly higher levels, with CD57⁺ cells at 1.7% and active caspase 3⁺ cells at 6.6%. CD36 remained low in both populations (CD36 positive control, Figure S3).

Upon 3 h co-culture, CD69^+^CAR^+^ T cells increased significantly on both days 5 and 10 (*p <* 0.0001, 0 h vs. 3 h) (Figure 4C). CAR^−^ T cells also upregulated CD69, likely due to bystander activation via cytokines in culture. CD25 remained high and unchanged in both CAR⁺ and CAR⁻ T cells. HLA class II expression further increased in day 10 CAR^+^ T cells (*p* < 0.0001, 0 h vs. 3 h; Figure 4C). Checkpoint expression during co-culture was subset and time point dependent. On day 5, CAR^+^ T cells showed increased PD1 (*p <* 0.0001) and LAG3 (*p <* 0.0001). CAR^−^ T cells displayed minimal PD1 or LAG3 induction, suggesting antigen-dependent exhaustion. On day 10, CAR^+^ cells showed no further upregulation of PD1 or LAG3. TIM3, which was elevated at baseline, remained high in both CAR^+^ and CAR^−^ subsets. Importantly, activation-induced apoptosis and senescence remained low. CD57^+^ and active caspase 3^+^ cells did not increase after 3 h of antigen exposure (Figure 4C).

Upon restimulation (12 h), day 5 and day 10 CAR⁺ T cells exhibited distinct activation kinetics and checkpoint profiles (Figures 4D and S2C). In day 5 products, CD69 expression increased during the initial 3 h co-culture, declined slightly at 12 h, and peaked at 15 h post restimulation. HLA class II and TIM3 remained elevated throughout. PD1 rose from 24.8% at baseline to 40.5% after 3 h co-culture and remained high following restimulation. LAG3, however, showed modest induction at 3 h (5.3% at 0 h vs. 11.1% at 3 h), reaching its highest level at 15 h (27.4%) (Figure S2C). Day 10 CAR⁺ T cells showed a sharp increase in CD69 expression from 15.9% at baseline to 81.2% after 3 h and remained high through 24 h. HLA class II expression was high and exceeded levels observed in day 5 products, consistent with the more sustained activation profile of day 10 cells. PD1 and LAG3 rose modestly during initial co-culture and increased further after restimulation. Throughout the 24 h assay, day 5 and day 10 CAR⁺ T cells maintained low active caspase 3 and CD57 expression (Figure S2C). CAR⁻ T cells expressed high levels of CD25 and TIM3 at baseline but exhibited only a modest change in HLA class II, CD69, PD1, and LAG3, indicating the absence of CAR independent activation. Together, day 5 CAR⁺ T cells respond more rapidly by upregulating PD1 and LAG3 upon first antigen encounter, whereas day 10 cells show delayed checkpoint expression, emerging only after extended antigen stimulation (Figures 4D and S2C).

#### Mid-expansion CAR T cells exhibit enhanced proliferation and metabolic activity

In the 3 h co-culture assay, both day 5 and day 10 CAR^+^ T cell products demonstrated potent target-specific tumor cell killing (Figure 3E). There was no statistically significant difference in overall cytotoxicity between day 5 and day 10 products (*p* = 0.39; Figure 3E), indicating that later harvest did not impair nor improve short-term killing capacity. Both day 5 and day 10 CAR T cells maintained strong cytotoxic activity upon antigen restimulation (12 h) and during prolonged exposure up to 24 h (Figure 3F). These data demonstrate that under a Nalm6 model, CAR T cells expanded for 5 days are as potent as those expanded for 10 days and remain capable of sustained tumor clearance *in vitro*.

**Figure 5:**
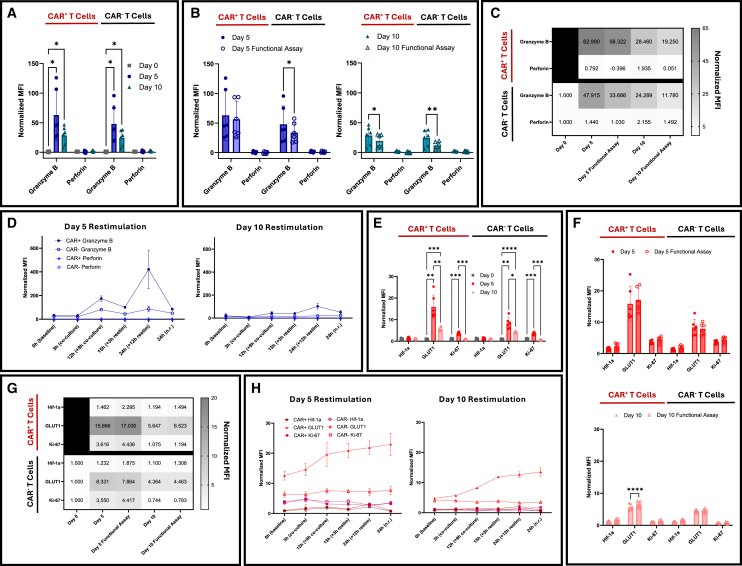
Functional and metabolic activity of CAR T cells during manufacturing (A and B) Intracellular granzyme B and perforin levels in CAR^+^ and CAR^−^ T cells at days 0, 5, and 10 (A) and after 3 h co-culture (B) (*n* = 6 donors, 2 independent experiments). Day 0 CAR^−^ samples served as the baseline reference for panel A. (C) Heatmap of normalized MFI for granzyme B and perforin across all conditions (*n* = 6 donors, 2 independent experiments). (D) Granzyme B and perforin kinetics during restimulation in CAR^+^ and CAR^−^ T cells (0–24 h, antigen additions at 0 and 12 h; *n* = 3 donors). (E and F) Expression of GLUT1, Hif-1a, and Ki-67 at days 0, 5, and 10 (E) and after 3 h co-culture (F) (*n* = 6 donors, 2 independent experiments). Day 0 CAR^−^ samples served as the baseline reference for panel E. (G) Heatmap showing MFI values for metabolic and proliferative markers across manufacturing and co-culture (*n* = 6 donors, 2 independent experiments). (H) Kinetic expression of GLUT1, Hif-1a, and Ki-67 over the 24 h restimulation assay in CAR^+^ and CAR^−^ T cells (*n* = 3 donors). All MFIs were normalized to the average of day 0 MFI of the respective marker. n.r., non-restimulated; restim, restimulated. Statistical analyses: two-way repeated measures ANOVA with Tukey’s (A, D, E and H), and Šídák’s (B and F) multiple comparisons test. Data shown as mean ± SD; ∗*p* < 0.05; ∗∗*p* < 0.01; ∗∗∗*p* < 0.001; ∗∗∗∗*p* < 0.0001. The absence of *p*-values denotes non-significance at the *p* = 0.05 threshold. All *p-*values for panels D and H are provided in Table S7.

To evaluate the cytotoxic machinery, we analyzed intracellular granzyme B and perforin levels. At baseline (day 0), both markers were minimally expressed (Figure 5A). Perforin remained undetectable through day 5 and day 10 in both CAR^+^ and CAR^−^ subsets (Figure 5A; perforin positive control, Figure S3). In contrast, granzyme B peaked at day 5 (*p <* 0.05 day 0 vs. day 5) and declined by day 10 (*p <* 0.05 day 0 vs. day 10). Following 3 h of co-culture, granzyme B levels dropped mainly in day 10 product (Figures 5B and 5C). During the restimulation assay, granzyme B levels modestly increased prior to second antigen exposure (normalized MFI: 174.1 for day 5, 42.5 for day 10; Figure S2D), then decreased again following rechallenge (normalized MFI: 100.9 for day 5, 40.2 for day 10; Figure S2D). By 24 h, granzyme B reached peak levels in restimulated cells from both day 5 and day 10 products (MFI 421.4 for day 5, 101.7 for day 10). This suggests that CAR^+^ T cells, particularly day 5 cells, can replenish and enhance their cytolytic payload after repeated antigen encounter (Figures 5D and S2D).

We next assessed metabolic fitness as a determinant of sustained T cell function. Hif-1a expression remained low during expansion and after 3 h of co-culture (Figures 5E and 5F), indicating minimal hypoxic stress. In contrast, GLUT1 was upregulated upon activation, peaking at day 5 (*p <* 0.01), then declining by day 10 (*p <* 0.01) while remaining above baseline (*p <* 0.001, day 0 vs. day 10; Figures 5E and 5G). Notably, switching from GLUT1 to GAPDH confirmed glycolytic pathway engagement, with a similar expression pattern (Figure S4, *n* = 1 donor). Ki-67 expression also peaked at day 5 (*p <* 0.001) and markedly declined by day 10 (*p <* 0.001). After 3 h of co-culture, both day 5 and day 10 CAR T cells showed minimal changes in Ki-67 and GLUT1 (Figure 5F). GLUT1 levels in CAR⁺ T cells gradually increased from 0 h through 24 h in both day 5 and day 10 products (Figures 5H and S2E), with a significant rise occurring after antigen restimulation (3 h vs. 15 h; day 5: *p* < 0.05; day 10: *p* < 0.01). Ki-67 expression remained stable, with minimal levels detected in day 10 CAR^+^ T cells. These findings indicate that day 5 CAR^+^ T cells exhibit higher metabolic and proliferative capacity.

#### Tregs decline while NK-like traits emerge over CAR T cell expansion periods

**Figure 6:**
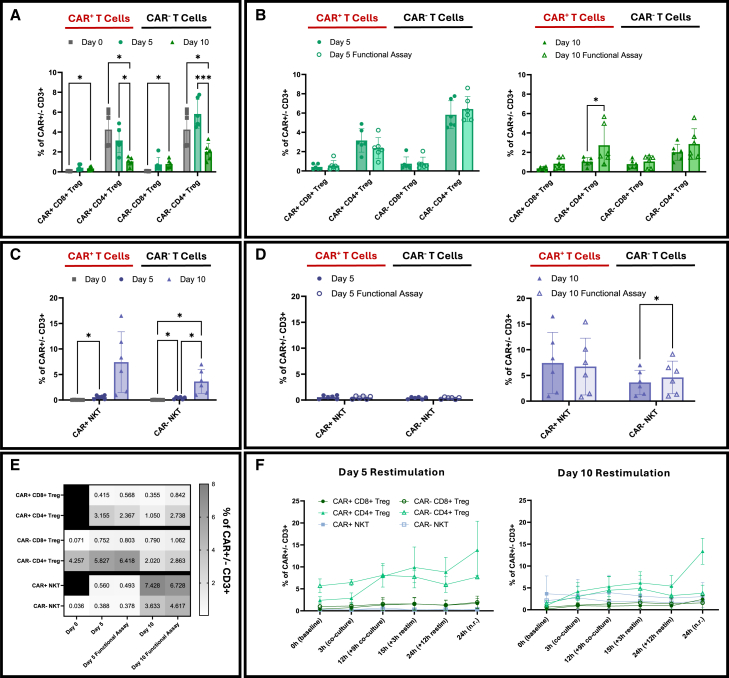
Frequencies and dynamics of Tregs and NK-like T cell subsets (A) Frequencies of CD4^+^ and CD8^+^ CD25^+^FoxP3^+^ Treg in CAR^+^ and CAR^−^ T cells across days 0, 5, and 10 (*n* = 6 donors, 2 independent experiments). Day 0 CAR^−^ samples served as the baseline reference. (B) Treg frequencies following 3 h co-culture with CD19^+^ Nalm6-YFP^+^ target and CD19^−^ Nalm6-GFP^+^ control cells on days 5 (left) and 10 (right) post-transduction (*n* = 6 donors, 2 independent experiments). (C) Frequencies of NK-like T cells in CAR^+^ and CAR^−^ T cells across days 0, 5, and 10 (*n* = 6 donors, 2 independent experiments). Day 0 CAR^−^ samples served as the baseline reference. (D) NK-like T cell frequencies following 3 h co-culture on days 5 (left) and 10 (right) post-transduction (*n* = 6 donors, 2 independent experiments). (E) Heatmap summarizing Treg and NK-like T cell subset frequencies across manufacturing time points and co-culture conditions (*n* = 6 donors, 2 independent experiments). (F) Frequencies of CD4^+^ and CD8^+^ Tregs and NK-like T cell subsets during 24 h restimulation in CAR^+^ and CAR^−^ T cells (0, 3, 12, 15, and 24 h; *n* = 3 donors). n.r., non-restimulated; restim, restimulated. Statistical analyses: two-way repeated measures ANOVA with Tukey’s (A, C and F), and Šídák’s (B and D) multiple comparisons test. Data shown as mean ± s.d.; ∗*p* < 0.05; ∗∗∗*p* < 0.001. The absence of *p*-values denotes non-significance at the *p* = 0.05 threshold. All *p-*values for panel F are provided in Table S7.

CD4⁺ Tregs (CD25⁺FoxP3⁺) comprised approximately 4.3% of total CD4⁺ T cells at baseline (day 0), while CD8⁺CD25⁺FoxP3⁺ cells were rare (<0.1%) (Figures 6A and 6E). By day 5, CD4⁺ Tregs accounted for 3.2% in the CAR⁺ fraction and 5.8% in the CAR⁻ fraction, but frequencies declined in both compartments by day 10 to 1.1% and 2.0%, respectively (Figure 6E). Following the 3 h co-culture, overall Treg frequencies remained stable. However, in two out of six donors at day 10, a modest but statistically significant increase in CAR⁺CD4⁺ Tregs was observed (*p* < 0.05; Figure 6B). In the 24 h restimulation assay, CD4⁺ Tregs increased in both CAR⁺ and CAR⁻ fractions on day 5 and day 10 products (Figures 6F and S2F). Natural killer (NK)-like (CD3⁺CD56⁺) T cells were nearly absent at baseline (<0.1%) and remained low through day 5 (<1%) in both CAR⁺ and CAR⁻ compartments. By day 10, CD56 expression increased overall, with CD56⁺CAR^+^ T cells representing an average of 7.4%, compared to 3.6% in the CAR⁻ fraction (Figures 6C and 6E). These CD56⁺ T cells were predominantly CD8⁺ (Figure S5), suggesting that extended expansion may promote NK-like features in a subset of CD8⁺ T cells. Upon the 3 h co-culture, NK-like T cell frequencies remained absent in day 5 CAR⁺ cells and were higher but stable on day 10. A small but statistically significant increase was observed in CAR⁻CD56⁺ T cells on day 10 (*p* < 0.05; Figure 6D). Throughout the restimulation, NK-like T cell frequencies remained stable in both day 5 and day 10 CAR⁺ T cells (Figures 6F and S2F).

#### Cryopreservation shows minimal impact on CAR T cell phenotype and functionality

**Figure 7:**
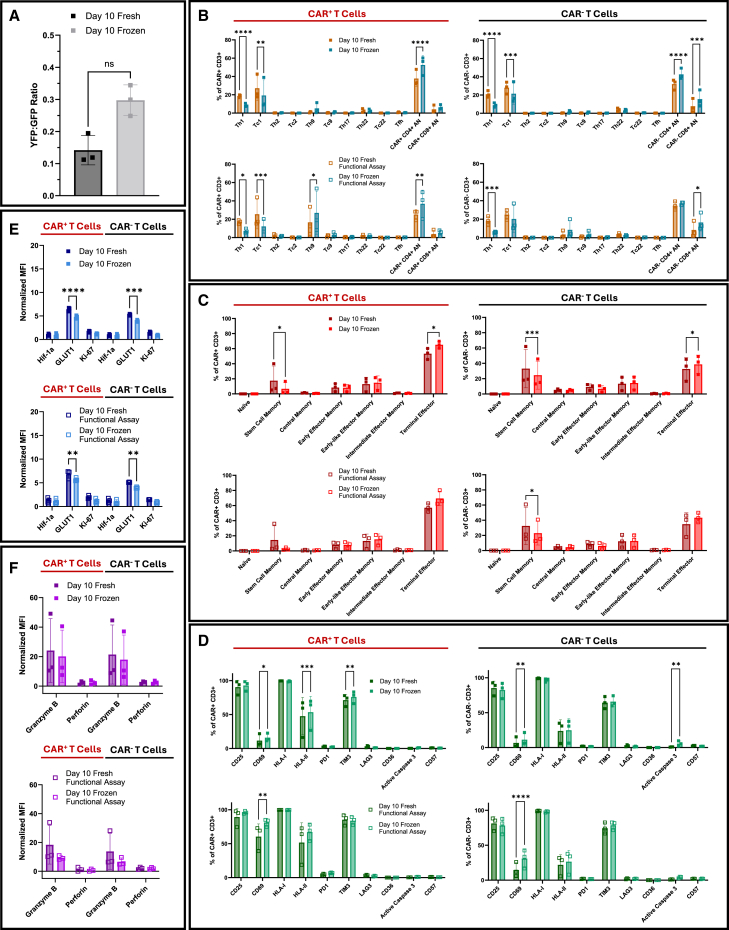
Cryopreservation effects on CAR T cell phenotype and function (A) Cytotoxicity of day 10 fresh vs. cryopreserved cells quantified by YFP:GFP ratio in 3 h co-culture assays with CD19^+^ Nalm6-YFP^+^ target and CD19^−^ Nalm6-GFP^+^ control cells (*n* = 3 donors). (B) Subset frequencies of Th1, Tc1, Th2, Tc2, Th9, Tc9, Th17, Th22, Tc22, Tfh, and uncommitted (AN) CD4^+^ and CD8^+^ cells in CAR^+^ and CAR^−^ compartments at baseline (0 h) and after 3 h co-culture (*n* = 3 donors). Only subsets representing ≥0.5% of total CD3^+^ T cells for any time point or donor are shown. (C) Differentiation state distributions (T_N_, T_SCM_, T_CM_, T_EM_, and T_TE_) in CAR^+^ and CAR^−^ CD3^+^ T cells at baseline (0 h) and after 3h co-culture (*n* = 3 donors). (D) Frequencies of CD25, CD69, HLA class I, HLA class II, PD1, TIM3, LAG3, CD36, active caspase 3, and CD57 in CAR^+^ and CAR^−^ CD3^+^ T cells at baseline (0 h) and after 3 h co-culture (*n* = 3 donors). (E) Normalized MFI of Hif-1a, GLUT1, and Ki-67 in CAR^+^ and CAR^−^ T cells at baseline (0 h) and after 3 h co-culture (*n* = 3 donors). (F) Normalized granzyme B and perforin levels in CAR^+^ and CAR^−^ T cells at baseline (0 h) and after 3 h co-culture (*n* = 3 donors). All MFIs were normalized to the average of day 0 MFI of the respective marker. Statistical tests: two-tailed Mann-Whitney *U* test (A); two-way repeated measures ANOVA with Šídák’s multiple comparisons test (B–F). Data shown as mean ± SD; ∗*p* < 0.05; ∗∗*p* < 0.01; ∗∗∗*p* < 0.001; ∗∗∗∗*p* < 0.0001. ns, not significant. The absence of *p*-values denotes non-significance at the *p* = 0.05 threshold.

To assess the impact of cryopreservation on CAR T cell function and phenotype, we analyzed day 10 CAR T cell products from three healthy donors under fresh and cryopreserved conditions. To simulate clinical practice, cells were rested for 3 hours post-thaw before evaluation at baseline (0 h) and after co-culture (Figures 7A–7F).^23^ Most markers in the 36-parameter panel were comparable between fresh and frozen samples (Figures 7, S6, and S7). Cryopreserved cells consistently exhibited a modest reduction in cytolytic activity shortly after thawing, although this was not statistically significant (Figure 7A). Compared to fresh products, frozen CAR⁺ T cells showed reduced Th1 and Tc1 frequencies and increased CD4⁺ AN cells (Th1: *p* < 0.0001; Tc1: *p* < 0.01; AN: *p <* 0.0001; Figure 7B). Memory phenotypes were minimally affected in frozen products, with a reduction in stem cell memory subsets and enrichment of terminal effector populations (Figure 7C). Cryopreservation further led to modest increases in CD69, HLA-II, and TIM3 expression and a reduction in GLUT1, while levels of exhaustion, apoptotic, senescence, and cytolytic markers remained overall stable (Figures 7D–7F). These differences largely persisted following 3 h co-culture (Figures 7B–7F), with continued reductions in Th1 (*p* < 0.05) and Tc1 (*p* < 0.001) frequencies, and a notable increase in Th9 cells in frozen samples (Figure 7B). Together, these results indicate that while cryopreservation alters some phenotypic features, the overall marker profiles remain broadly comparable.

#### High-dimensional analyses reveal distinct marker combinations

Sequential gating effectively identifies pre-defined populations but can miss marker relationships outside conventional gates. To address this, we performed Uniform Manifold Approximation and Projection (UMAP) analysis of CD45⁺ gated cells at days 0, 5, and 10. Day 0 cells clustered tightly with uniformly low expression of activation, metabolic, and cytotoxic markers, consistent with a resting, pre-engineered phenotype. By day 5, cells shifted into distinct UMAP regions characterized by co-expression of memory and metabolic markers (CCR7, CD45RA, GLUT1), proliferative and activation signals (Ki-67, CD25), and cytotoxic effectors (granzyme B). In contrast, day 10 cells populated new clusters defined by CD8 dominance, HLA class II, TIM3, and CD45RA expression with reduced CCR7, indicating terminal differentiation (Figure S8). This was accompanied by a shift from a Tc1-, Th9- and Th1-enriched T_SCM_ memory repertoire at day 5 to predominantly Tc1 subsets at day 10, consistent with selective expansion of the cytotoxic lineage observed during CAR T cell manufacturing (Figures 2F, 2H and S9). The 3 h co-culture induced rapid CD69 upregulation at both timepoints, with minimal changes in UMAP distribution, suggesting preserved phenotypic organization despite antigen exposure (Figure S10).

**Figure 8:**
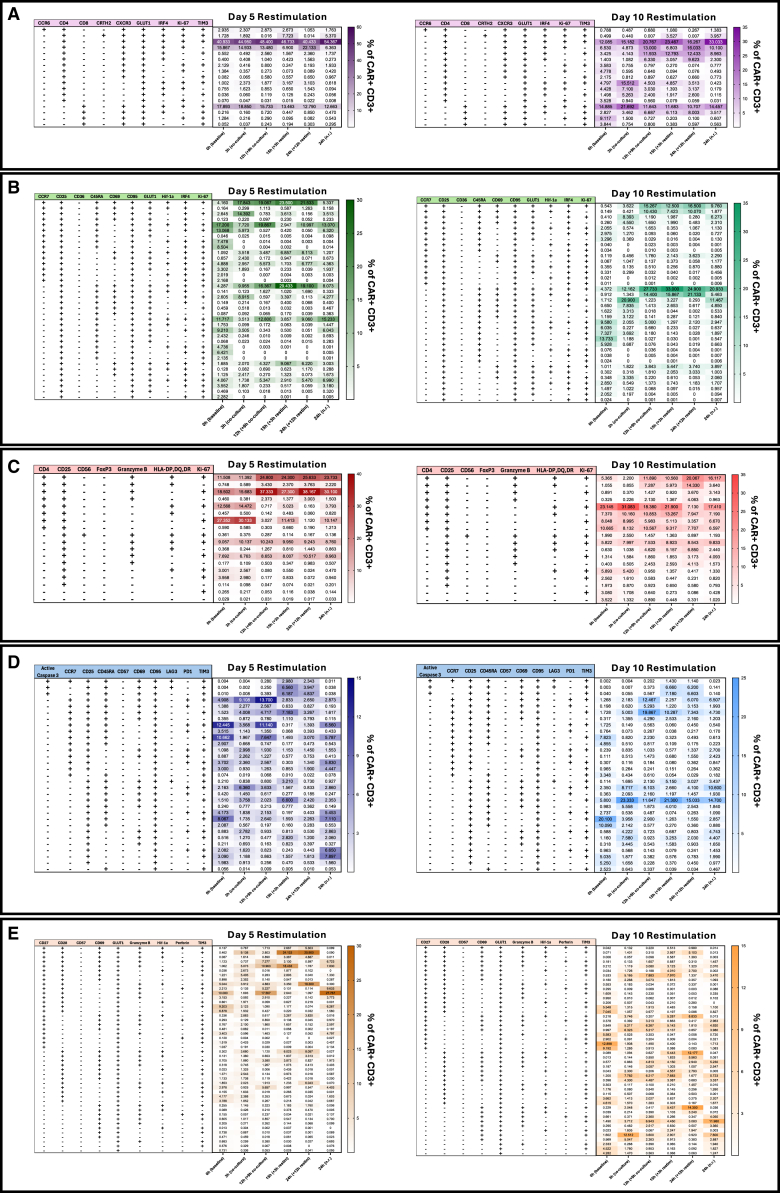
High-dimensional combinatorial analysis of CAR T cell phenotypes Combinatorial co-expression analysis of CD3^+^CAR^+^ T cells at day 5 (left) and day 10 (right) after restimulation, grouped by functional modules. Frequencies shown represent percentage of total CAR^+^CD3^+^ cells; only marker combinations with >5% expression in at least one donor or time point are shown. Data from *n* = 6 donors for 0–3 h, and *n* = 3 donors for 12–24 h. (A) Helper and cytotoxic polarization with exhaustion markers (CCR6, CD4, CD8, CRTH2, CXCR3, GLUT1, IRF4, Ki-67, TIM3). (B) Memory, proliferation, and metabolic markers (CCR7, CD25, CD36, CD45RA, CD69, CD95, GLUT1, Hif-1a, IRF4, and Ki-67). (C) Treg, NK-like T cell, proliferation, functional, and HLA class II markers (CD4, CD25, CD56, FoxP3, granzyme B, HLA-DP, DQ, DR, Ki-67). (D) Activation, stemness, apoptotic, senescence, and exhaustion markers (active caspase 3, CCR7, CD25, CD45RA, CD57, CD69, CD95, LAG3, PD1, TIM3). (E) Cytotoxic and metabolic effector markers (CD27, CD28, CD57, CD69, GLUT1, granzyme B, Hif-1a, perforin, TIM3).

To further reveal unrecognized marker relationships, combinatorial gating partitioned the CD3^+^CAR^+^ populations into sub-populations defined by specific marker co-expression patterns (Figure 8). Day 5 products were dominated by CD4⁺ Th1 cells co-expressing Ki-67 and IRF4, whereas day 10 products exhibited a more heterogeneous mix of Th1 and Tc1 subsets, likely driven by reduced CD4 proliferation (Figure 8A). A CCR7⁺CD45RA⁺ T_SCM_-like population enriched at day 5 co-expressed CD25, GLUT1, Ki-67, and IRF4, consistent with a proliferative and metabolically active state. By contrast, day 10 cells shifted toward a CCR7⁻CD45RA⁺ effector phenotype with variable Ki-67 expression, suggesting divergent proliferative capacity (Figure 8B). A highly proliferative (Ki-67⁺) and functional (CD25⁺granzyme B⁺) cell subset was prevalent in day 5 cultures upon antigen exposure. In contrast, day 10 cells lacked this distinct population and displayed more variable Ki-67 expression (Figure 8C). Day 5 T_TE_ (CD45RA⁺CCR7⁻) and T_SCM_-like (CD45RA⁺CCR7⁺CD95⁺) subsets showed distinct CD69 and PD1 expression dynamics during antigen exposure and rechallenge. By comparison, day 10 cells exhibited a more uniform phenotype, dominated by CCR7⁻CD45RA⁺TIM3⁺PD1^-^ populations (Figure 8D). This analysis supports the activation and exhaustion dynamics initially observed in Figure 4. Metabolic remodeling was also evident, as day 5 cells maintained GLUT1 along with co-stimulatory markers CD27 and CD28. Day 10 products showed a reduced proportion of CD27⁺CD28⁺ double-positive cells, a phenotype associated with limited persistence (Figure 8E). Overall, this combinatorial approach has the ability to resolve molecular diversity within defined subsets, offering mechanistic insights into CAR T cell behavior not usually captured by conventional gating.

## Discussion

In this study, we developed and applied a high-dimensional spectral flow cytometry panel to map the phenotypic and functional trajectories of CAR T cells throughout manufacturing. Our analysis revealed distinct cellular states associated with mid (day 5) and late (day 10) expansion time points. By day 5, CAR T cells exhibited a favorable profile marked by CD4^+^ Th1 predominance, T_SCM_ enrichment, elevated glycolytic activity (GLUT1), and high proliferative capacity (Ki-67). This profile was associated with robust expansion and strong killing upon antigen exposure. In contrast, day 10 cells displayed a more terminally differentiated phenotype, dominated by CD8^+^ Tc1 subsets, reduced stem cell memory subsets, and increased CD56^+^ NK-like populations. While both products retained potent killing capacity against the CD19^+^ Nalm6 model, the day 5 phenotype is more consistent with features linked to sustained *in vivo* efficacy.^22^^,^^24^^,^^25^^,^^26^ This trajectory reflects a manufacturing trade-off, where extended expansion increases yield but depletes T_SCM_ and T_CM_ subsets linked to clinical benefits. Meanwhile, it enriches for differentiated T_TE_ populations that may limit durability.^13^^,^^16^^,^^27^^,^^28^ In line with studies showing that brief *ex vivo* expansion (1–3 days) preserves memory traits and enhances engraftment,^24^^,^^29^ our findings suggest that harvesting CAR T cells around day 5 may achieve a more desirable balance between yields and sustained therapeutic potential.

During CAR T cell manufacturing, the emergence of minor yet functionally significant subsets may contribute to disparate clinical outcomes. By day 10, we observed a notable increase in CD3^+^CD56^+^ NK-like cells within the CAR^+^ fraction, particularly among CD8^+^ T cells. These subsets were absent at days 0 and 5, suggesting their development is associated with prolonged culture time. CD56 expression, traditionally a marker for NK cells, has been identified on activated T cells and is linked to enhanced cytotoxic potential.^30^ However, recent evidence indicates that sustained stimulation can drive CD8^+^ T cells to adopt NK-like features, with a shift toward functional impairment in CAR T cell models.^31^ Our findings suggest that extended culture alone may promote the emergence of these phenotypes, even without continuous antigen exposure. Monitoring CD3⁺CD56⁺ subsets may serve as a useful quality control measure to avoid enriching exhausted or dysregulated cells. Overall, this demonstrates the panel’s utility in detecting subtle phenotypic changes during manufacturing.

Integrating proliferation and metabolic markers into our analysis revealed distinct mechanisms driving the shift from Th1 to Tc1 dominance during CAR T cell expansion. This transition reflects reduced CAR^−^CD4^+^ and expanded CD8^+^ subsets within the CAR^+^ fraction. CAR^+^CD8^+^ T cells expressed high levels of glucose transporter GLUT1, suggesting a selective proliferative and metabolic advantage. In contrast, CD4^+^CAR^−^ T cells showed lower proliferation rates, potentially contributing to their relative decline by day 10. This shift in the CD4:CD8 ratio is clinically relevant, as CAR T products with defined CD4:CD8 ratios have been associated with improved clinical outcomes.^32^^,^^33^^,^^34^ Incorporating metabolic markers such as GLUT1 provides insight into the energetic state of CAR T cells during expansion.^35^^,^^36^ Peak GLUT1 and Ki-67 expression at day 5 indicate a metabolically and proliferatively active state. By day 10, these markers decline yet remain elevated compared to day 0, suggesting expanded cells maintain metabolic and cytotoxic potential despite reduced proliferation. Understanding these phenotypic shifts during manufacturing informs strategies to preserve metabolic fitness, delay exhaustion, and enhance the durability and efficacy of CAR T cell therapies.^35^^,^^36^

During manufacturing, CAR T cells exhibited distinct checkpoint and activation marker dynamics. CD25 remained constitutively high from day 5 onward, reflecting sustained activation, whereas CD69 expression was transient and context dependent, increasing during antigen exposure and rechallenge assays. Day 5 CAR^+^ cells rapidly upregulated checkpoint receptors PD1 and LAG3 upon antigen stimulation, while day 10 cells displayed delayed kinetics. TIM3 levels were elevated from day 5 through day 10, indicating early and sustained expression. Despite the presence of exhaustion-associated markers, apoptotic indicators such as active caspase 3 remained low, showing no evidence of activation-induced cell death. These findings suggest that exhaustion phenotypes dynamically emerge during manufacturing and antigen exposure but do not necessarily result in immediate functional impairment. Importantly, *in vitro* expression of inhibitory receptors does not directly predict clinical exhaustion. *In vivo*, CAR T cells encounter sustained antigen stimulation and immunosuppressive pressures from the tumor microenvironment, conditions that promote functional exhaustion and limit persistence.^18^^,^^37^^,^^38^^,^^39^^,^^40^ Therefore, *in vitro* exhaustion profiling must be interpreted within the broader clinical context. To bridge this gap, this panel can be used to correlate manufacturing phenotypes with post-infusion outcomes and identify biomarkers predictive of CAR T cell efficacy.

Comparative profiling of CAR^+^ and CAR^−^ T cells revealed parallel phenotypic trajectories, characterized by increased activation markers, shared differentiation patterns, and dynamic checkpoint receptor expression. However, CAR^+^ cells exhibited greater metabolic activation, likely due to CAR-mediated signaling or a selective growth advantage. The observation that both CAR^+^ and CAR^−^ cells undergo similar phenotypic progression suggests that expansion conditions may have a greater impact on product identity than the specific gene transfer method used. As such, this assay platform can be applied to diverse gene editing systems, including CRISPR, base editing, and transposons to evaluate phenotypic outcomes and disentangle the contributions of specific engineering platforms.

Cryopreservation remains central to CAR T cell manufacturing and clinical logistics, particularly as therapy scales globally. Our findings show that core phenotypic and functional attributes are largely preserved following freeze-thaw cycle. Cryopreserved day 10 products maintained comparable phenotypic profiles to fresh samples, with slight reductions in Th1 or Tc1 frequencies and modest changes in CD69, HLA-DP, DQ, DR, TIM3, or GLUT1 levels. Antigen responsiveness was largely preserved following cryopreservation. Clinically, these findings are reassuring and align with data showing comparable efficacy between fresh and cryopreserved CAR T cell products.^23^^,^^41^^,^^42^

Another notable finding is the presence of CAR^+^ Tregs within the product, which could have a dual effect. While they may exert immune-regulatory functions by dampening excessive inflammatory responses,^43^ they could also suppress anti-tumor activity and limit therapeutic efficacy.^19^ Additionally, HLA class II upregulation observed in CAR^+^ cells poses a known risk to allogeneic use, typically mitigated through targeted genome editing.^44^ Together, this study demonstrates the value of dynamic phenotypic profiling to capture shifts in CAR T cell state during production. By integrating differentiation, metabolic, activation, and functional attributes, this approach offers the potential to uncover mechanistic insights into how manufacturing conditions influence product features associated with clinical performance.

While our study provides detailed insights into CAR T cell manufacturing dynamics, it also lays the groundwork for broader application. Although *in vitro* co-culture assays cannot fully capture the complexity of *in vivo* responses, this framework can be extended to patient-derived products to help link cellular phenotypes with clinical outcomes. The spectral panel itself is platform-agnostic and adaptable to diverse manufacturing protocols, enabling comparative profiling across workflows. Moreover, the panel’s modular design allows marker substitution, as demonstrated by replacing GLUT1 with GAPDH, and can be extended to include mitochondrial or fatty acid metabolism to complement glycolytic readouts. This versatility makes the panel highly adaptable across diverse cell types and diseases. Building on this flexibility, we identified a focused set of markers suitable for in-process and release testing to support clinical manufacturing (Table S2).

### Conclusion

This study presents a novel high-dimensional framework for profiling CAR T cells throughout manufacturing, capturing key attributes that drive their behavior and function. Among the many insights revealed by this dataset, we show that harvest timing defines which cellular states are captured in the final drug product. Mid-harvest products consistently exhibit memory-enriched, metabolically active phenotypes, while later-harvested cells display terminal differentiation yet retain functional capacity. These findings establish a foundation for identifying previously unrecognized critical quality attributes that may influence CAR T cell product performance. By characterizing the impact of manufacturing variables, this platform can help align product features with specific therapeutic goals. Looking ahead, the application of this panel in clinical settings may uncover predictive biomarkers that link product phenotypes to patient outcomes, guiding the next generation of cell therapy design.

## Materials and methods

### Spectral flow cytometry panel development

#### Antibody titration

Antibodies were titrated on their respective positive cell populations (Table S3) at serial dilutions, and optimal concentrations were determined based on calculated stain indices and resolution between the negative and positive populations. Each titration was performed in technical duplicate using 5 serial dilutions in the appropriate staining buffer (surface antibodies in BSA stain buffer [BD Biosciences, catalog no. 554657]; intracellular antibodies in 1× permeabilization buffer prepared from 10× stock [eBioscience, catalog no. 00-8333-56]). After staining, cells were fixed in 0.5% formaldehyde (200 μL) (Thermo Scientific, catalog no. 28908) and stored at 4°C in the dark until acquisition.

#### Sample preparation and multicolor staining

During panel development, different sample sources were used to ensure broad marker coverage. For initial antibody titrations, purified cell subsets known to express the target markers were chosen (Table S3). Once optimal antibody concentrations were established, a composite sample was generated by pooling peripheral blood mononuclear cells (PBMCs), gene-edited CAR T cells, non-edited T cells, and activated T cells in varying ratios to ensure comprehensive marker representation. Nalm6 GFP and YFP cells were further spiked at different ratios. All samples (including single cell types and mixed populations) were processed using the multicolor staining protocol in the supplemental methods. Before staining, all antibody stock solutions were centrifuged at 10,000 × *g* for 5 min at 4°C to pellet any antibody aggregates. Cells were stained according to an optimized protocol consisting of four extracellular steps: Fc block, CAR detection, pre-stain, and extracellular staining, followed by an intracellular staining step after fixation and permeabilization. Master mixes were prepared for each step (see Table S4 for composition).

Staining was performed in 5-mL polystyrene fluorescence-activated cell sorting (FACS) tubes with volumes as indicated. If starting from cryopreserved PBMCs, cells were thawed according to standard protocols and allowed to rest for ≥8 h in culture medium before staining to restore surface epitopes. Samples were resuspended in DPBS (Corning, catalog no. 21-031-CV) for final acquisition.

#### Fluorescence controls

For each fluorochrome, single-color (SC) controls and fluorescence minus one (FMO) controls were included, along with unstained controls (Figure S11). All control samples were handled in parallel with fully stained samples, undergoing the same steps but omitting or substituting specific reagents as appropriate. Refer to Table S4 for further details on the antibody master mix and prepared buffers.

#### Reference controls and spectral unmixing

For spectral unmixing, both cell-based and bead-based SC reference controls were prepared (Table S5). UltraComp eBeads (Invitrogen, catalog no. 01-3333-42) or Cytek FSP CompBeads (Cytek Biosciences, catalog no. B7-10011) were used for fluorescent antibody capture when cell controls were unavailable due to low marker expression. Single-color reference samples using beads were prepared by mixing one drop of beads with the applicable antibody and stain buffer at its respective staining step. Notably, SC bead samples were only included in the relevant portions of the protocol (e.g., an intracellular marker control was not exposed to surface staining steps prior to fixation).

Spectral unmixing was performed using SpectroFlo software v3.3.0 (Cytek Biosciences). Reference controls were selected to meet strict quality criteria: each SC control was at least as bright as the corresponding signal in a fully stained sample and unmixed against a corresponding unstained bead control. For unmixing, at least 100 bright events were collected from each SC reference, ensuring a clear full-spectrum signature for each fluorochrome. Full-spectrum and normalized full-spectrum signatures were assessed for each reference control, and only controls that generated a matching spectral signature to the CytekCloud database were used (Cytek Biosciences). Specifically, controls that showed baseline expression in unexpected detectors were rejected. Using these criteria, a library of optimal reference controls (both cell-based and bead-based) was established.

Live unmixing was then applied, and results were checked by inspecting the N × N spectral matrix of all parameters in fully stained samples. Population distributions in each channel were examined for any anomalies inconsistent with expected biology (e.g. distortions or shifts due to unmixing errors). When necessary, the unmixing was refined in a stepwise manner: (1) adjusting the gating of the reference control to capture a pure population, (2) substituting a dim reference control with a brighter one, (3) using an “internal negative” cell population (cells within the sample that lack expression of the marker) in place of the universal negative for a given marker, or (4) swapping a bead-based reference for a cell-based reference (or vice versa). Any residual spillover/unmixing error remaining after these adjustments was quantified and applied using SpectroFlo’s Adjust Spillover tool. These values are reported in Figure S12, with representative unmixing errors and corrections shown in Figure S13.

#### Instrument setup and data acquisition

Flow cytometry data were acquired on a Cytek Aurora spectral cytometer equipped with four lasers (405 nm violet, 488 nm blue, 561 nm yellow-green, 640 nm red), using the configuration detailed in Figure S14. The instrument was allowed to warm up for 30 min, and standard daily QC was performed with SpectroFlo QC beads (Cytek, catalog no. B7-10001) before acquiring samples. Cytometer settings followed the Cytek-recommended assay settings. Antibody staining concentrations were optimized to yield in-scale signals for all channels, with particularly bright markers prone to fluorescence spread intentionally used at lower saturating concentrations to minimize spillover spreading. Sample tubes (200 μL volume) were gently vortexed immediately before acquisition to disrupt any cell aggregates. Unless stated otherwise, ∼200 μL of each sample was acquired per run at a high flow rate (∼80 μL/min).

#### Gating strategy

To ensure robust identification of cell populations in high-dimensional data, the gating strategy was carefully developed and validated using appropriate controls. A full set of FMO controls was used to inform gate thresholding for future experiments (Figure S11 provides a detailed overview of all FMO results). These FMO controls helped determine which markers should be included in the routine use of the panel. In addition, fluorescence spread was addressed in the gating approach. This two-dimensional gating strategy reduced the impact of spread on the discrimination of positive vs. negative populations. All gating was performed on biologically relevant parent populations (e.g., live singlet lymphocytes, CD3^+^ T cells), and gating hierarchy and logic were kept consistent across all samples (Figure 1). Figure S15 illustrates enhanced marker resolution achieved through a representative manual gating strategy.

#### Data extraction

Subset frequencies were reported as percentages of the immediate parent population (e.g., CD3^+^CAR^+^), allowing for direct comparison of phenotypic distributions over time. For some intracellular markers (GLUT1, GAPDH, granzyme B, perforin, Hif-1a), MFI was extracted from the entire parent population and normalized to the average day 0 MFI across donors. This approach enabled accurate fold-change quantification over the course of manufacturing and stimulation. Ki-67 was analyzed separately due to its negative baseline expression. Using day 0 negative signal intensities for normalization may complicate interpretation of Ki-67 expression. To enable accurate comparison of proliferative activity across timepoints, MFI values were calculated exclusively from the Ki-67⁺ gated population (Figure S16).

### Cell culture and functional assays

#### CAR construct

For all manufacturing runs, we employed a second-generation CAR targeting human CD19. The construct consisted of an FMC63-derived single-chain variable fragment (scFv) for antigen recognition, joined to a human CD8α hinge and transmembrane domain. Intracellular signaling was mediated through a 4-1BB (CD137) co-stimulatory domain followed by CD3ζ (Figure S17A).

#### CAR T cell production

Human CD3^+^ T cells were obtained from healthy donor PBMCs or purchased from Excellos (San Diego, CA). All biological materials were used under the oversight of the Institutional Biosafety Committee at the University of Southern California (BUA No. 22-00032). For lentiviral CAR T cell generation, T cells were activated with Human T Cell TransAct (Miltenyi Biotec, catalog no. 130-128-758) at a 1:100 dilution and simultaneously transduced with a second-generation anti-CD19 CAR lentiviral vector (Vector BioMed, Anti-CD19 CAR LV) at a multiplicity of infection (MOI) of 20. Transductions were carried out in G-Rex 6M (Wilson Wolf, catalog no. 80660M; 10 cm^2^ gas-permeable culture area per well) to support high-density cell growth. Three days after transduction, fresh culture medium was added to fill the G-Rex well to its maximum volume. On day 5 post-transduction, an in-process sample was harvested for analysis, and the remaining cells were re-seeded into new G-Rex 6M vessels to continue expansion until day 10. All T cell cultures were maintained in complete T cell media consisting of TexMACS medium (Miltenyi Biotec, catalog no. 170-076-306), supplemented with 3% human AB serum (BioIVT, catalog no. HUMANABSRMP-HI-1), 12.5 ng/mL recombinant human interleukin-7 (IL-7) (Bio-Techne, catalog no. BT-007-AFL-025), and 12.5 ng/mL recombinant human IL-15 (Bio-Techne, catalog no. BT-015-AFL-025). Cultures were incubated at 37°C in a humidified 5% CO_2_ atmosphere. This complete medium was used for all T cell expansion steps unless otherwise noted. Initial panel development and antibody optimization were performed using non-viral CAR T cells generated by electroporation of DNA encoding the CD19-targeting CAR construct, as previously described.^45^ These early experiments facilitated the refinement of antibody titration, staining protocols, and gating strategies. For all subsequent manufacturing runs and phenotypic analyses, lentivirally transduced CAR T cells were used.

#### Cell lines

The Nalm6 acute lymphoblastic leukemia cell line (CD19^+^) and a derivative Nalm6 GFP CD19 knockout line (lacking surface CD19 expression) were provided by the Stanford Laboratory for Cell and Gene Medicine. An additional Nalm6 variant expressing YFP (Nalm6 YFP) was generated in-house by transducing Nalm6 cells with a lentiviral EF1α-eYFP (Topaz) vector (BPS Bioscience, catalog no. 79989) at an MOI of 1.25, following the manufacturer’s protocol. On day 5 post-transduction, YFP^+^ cells were enriched by FACS (BD FACSAria II), and purity was subsequently confirmed by flow cytometry. All cell lines were cultured in Advanced RPMI 1640 medium (Gibco, catalog no. 12633020), supplemented with 10% fetal bovine serum (Corning, catalog no. 35-015-CV), and 1% GlutaMAX (Gibco, catalog no. 35050-061). Cell cultures were maintained at 37°C with 5% CO_2_.

#### Functional cytotoxicity assay

A standardized effector-target co-culture assay was used to evaluate CAR T cell cytotoxicity in tandem with phenotypic profiling. Viral CD19 CAR T cells at two expansion time points (day 5 and day 10 post-transduction) were cryopreserved in CryoStor CS10 (STEMCELL Technologies, catalog no. 07930) and stored in vapor-phase liquid nitrogen. For the assay, frozen cells were thawed, washed, resuspended in complete T cell media (TexMACS + 3% hAB serum + 12.5 ng/mL IL-7/IL-15), and rested for at least 3 h prior to co-culture. CAR T cells were then mixed with target and control tumor cells at a 2:1:1 ratio: 5E5 CAR T cells with 2.5E5 CD19^+^ Nalm6-YFP^+^ target cells and 2.5E5 CD19^−^ Nalm6-GFP^+^ control cells in 24-well plates. Plates were briefly centrifuged (100 × *g*, 3 min, room temperature) to promote cell-to-cell contact and incubated for 3 h at 37°C and 5% CO_2_. Following co-culture, cells were pooled per donor, stained with the full 36-marker spectral flow cytometry panel, and analyzed in a single-tube workflow. Antigen-specific killing was quantified as the ratio of viable YFP^+^ to GFP^+^ cells (viable CD19^+^YFP^+^)/(viable CD19^−^GFP^+^), with a reduction in this ratio indicating selective killing of CD19^+^ targets (Figure S17B). Non-transduced T cells were included as negative controls in three of six donors to confirm CAR-dependent target elimination.

#### Restimulation assay

To evaluate the durability and dynamics of CAR T cell responses following repeated antigen exposure, a restimulation assay was performed using CAR T cells from three donors (Figure 2B). At the 12 h timepoint, CD19^+^ Nalm6-YFP^+^ target cells (effector-to-target ratio 2:1) were added to the culture to simulate a second antigenic encounter. Samples were collected at baseline (0 h), after initial co-culture (3 h), and following extended incubation at 12 h, 15 h (3 h post-restimulation), and 24 h. In parallel, a matched condition without restimulation (24 h non-restimulated) was included to assess phenotype evolution independent of secondary antigen exposure. Cells were analyzed using the 36-marker spectral cytometry panel. Cytotoxic activity was assessed by measuring the frequency of viable YFP^+^ cells at each time point, enabling assessment of both initial and sustained target cell elimination.

#### T cell activation

Resting human T cells (isolated from PBMCs or obtained from Excellos) were activated with Human T cell TransAct (Miltenyi) at a 1:100 dilution. Cells were incubated in a T-25 flask for 24–72 h (37°C, 5% CO_2_) to achieve broad activation. These activated T cells were used to validate activation markers in the panel (e.g., CD69 and CD25 upregulation at 48 and 72 h).

### UMAP clustering and high-dimensional analysis

High-dimensional visualization and clustering of immune phenotypes were performed using UMAP and Flow Self-Organizing Map (FlowSOM) algorithms implemented within FlowJo (version 10.10.1). For UMAP analysis, 50,000 CD45^+^ events were randomly subsampled and concatenated per condition. The UMAP plugin (version 4.1.1) was applied using default parameters: Euclidean distance metric, 15 nearest neighbors, minimum distance of 0.5, and 2 output dimensions. All panel markers were included except for viability dye, GFP, and YFP. FlowSOM clustering was performed using the FlowJo FlowSOM plugin (version 4.1.0). SOMs were generated on the same concatenated datasets as were used for UMAP. Analysis parameters included a 10 × 10 grid size, generation of 10 meta-clusters, and default visualization settings (minimum spanning tree layout with pie chart representation of marker expression, hierarchical heatmaps based on both rows and columns, and node scaling set to 100%).

### Combinatorial gating analysis

To assess multi-marker co-expression patterns across functional and differentiation axes, combinatorial gating was performed using FlowJo (version 10.10.1). Individual gates were first defined for all markers of interest based on FMO controls and pre-established positive/negative thresholds. Boolean logic was then applied within FlowJo to generate all possible combinations of marker expression across selected functional categories (e.g., activation, memory, exhaustion, metabolic, cytotoxic, lineage). Each Boolean gate reflected a unique combination of marker expression states and was used to quantify the frequency of complex phenotypes across time points and stimulation conditions. Outputs were exported using the FlowJo Table Editor for downstream analysis and graphical representation.

### Statistical analysis and data presentation

Spectral unmixing and manual compensation were performed using SpectroFlo software (version 3.3.0, Cytek Biosciences). Gating was done using FlowJo (version 10.10.0). Unless otherwise specified, data are presented as mean ± standard deviation (SD). Differences in T cell subtypes and marker expression across key manufacturing time points (days 0, 5, and 10) and restimulation readouts were evaluated by two-way repeated measures ANOVA with Tukey’s multiple comparisons test (*p* < 0.05). CD4/CD8 CAR^+^ and CAR^−^, fresh versus frozen products, and co-culture assay comparisons for day 5 and day 10 viral CAR T cells were performed using two-way repeated measures ANOVA with Šídák’s multiple comparisons test (*p* < 0.05). For comparison of YFP:GFP ratios and relative YFP frequencies, a two-tailed Mann-Whitney *U* test or one-way ANOVA with Šídák’s multiple comparisons test was used (*p* < 0.05). Differences in CAR expression and MFI on days 5 and 10 of manufacturing were assessed by paired, two-tailed Student’s t test (*p* < 0.05). For all statistical tests, *p-*values are denoted with asterisks as follows: ∗*p* < 0.05, ∗∗*p* < 0.01, ∗∗∗*p* < 0.001, and ∗∗∗∗*p* < 0.0001. The absence of *p-*values denotes non-significance at *p* = 0.05 threshold. Schematics and graphs presented in the figures were created using BioRender (www.biorender.com), GraphPad Prism (version 10.2.3), and Microsoft Excel.

## Data availability

The datasets generated and analyzed during this study are available from the corresponding author upon reasonable request. All flow cytometry panel design and optimization details are included in the supplemental information.

## Acknowledgments

M.A. is supported in part by the 10.13039/100000054National Cancer Institute under award no. P30CA014089. The content is solely the responsibility of the authors and does not necessarily reflect the official views of the National Cancer Institute or the National Institutes of Health. The authors acknowledge Mark Edinger and Aric Bitton (Cytek Biosciences) for their support during the design and interpretation of the panel.

## Author contributions

A.C.-G., C.L.F., and M.A. conceptualized the study. A.C.-G., C.L.F., A.V., and M.A. designed the experiments. A.C.-G., C.L.F., and A.C. conducted the experiments. All authors analyzed the data. A.C.-G., C.L.F., and M.A. wrote the manuscript. All authors reviewed and approved the final manuscript.

## Declaration of interests

A.C.-G., C.L.F., and M.A. are inventors on a patent application related to the methods described in this study. A.C., E.J., and A.V. declare no competing interests.

## Supplemental information

Document S1. Supplemental methods, Tables S1–S7, and Figures S1–S27

Document S2. Article plus supplemental information
