# Supplementary material for: High-dimensional temporal mapping of CAR T cells reveals phenotypic and functional remodeling during manufacturing
Source: Mol Ther. 2025 May 1;33(5):2291–309. doi: 10.1016/j.ymthe.2025.04.006 (PMC12126796; doi:10.1016/j.ymthe.2025.04.006)
Supplement: Document S1. Supplemental methods, Tables S1–S7, and Figures S1–S27 [file mmc1.pdf]

YMTHE, Volume *33*

Supplemental Information

High-dimensional temporal mapping of CAR T cells reveals phenotypic and functional remodeling during manufacturing

Amaia Cadinanos-Garai, Christian L. Flugel, Anson Cheung, Enzi Jiang, Alix Vaissié, and Mohamed Abou-el-Enein

Supplemental Methods: Design and Optimization of a 36-Marker Spectral Flow Cytometry Panel for CAR T Cell Profiling

T Cell Subset Phenotyping

To enable comprehensive profiling of T cell lineage and polarization states, the panel was configured to distinguish $CD4^+$ helper and $CD8^+$ cytotoxic T cells along with their specialized subsets.[1,2] Chemokine receptors and transcription factors were included to delineate canonical T helper lineages (Th1, Th2, Th9, Th17, Th22, and Tfh) and their cytotoxic analogs (Tc1, Tc2, Tc9, Tc17, Tc22, Tfc) (**Figure S15**).[3–7] CD25 and FoxP3 were used to identify Tregs within both $CD4^+$ and $CD8^+$ compartments, given their impact on CAR T efficacy.[8] CD56 was included to detect $CD3^+CD56^+$ NK-like T cells, a subset implicated in CAR T function and persistence.[9]

T Cell Differentiation and Memory Phenotypes

CD45RA, CCR7, and CD95 were used to define naïve and memory subsets. Naïve T cells (T_N) were defined as $CD45RA^+CCR7^+CD27^+CD28^+CD95^-$, and stem cell memory ($T_{SCM}$) as $CD45RA^+CCR7^+CD27^+CD28^+CD95^+$. Central memory ($T_{CM}$), effector memory ($T_{EM}$), and terminal effector (T_{TE}) subsets were further classified by their distinct CD45RA and CCR7 expression.[10] CD27 and CD28 provided additional resolution of maturation stages (early-like, early, and intermediate effector memory) (**Figure S15**).

Activation, Metabolic, Exhaustion, Senescence, and Apoptotic Markers

Surface and intracellular markers were selected to monitor activation status, metabolic programming, and exhaustion. Early activation was marked by CD69, whereas CD25 captured mid and late activation states.[11] Proliferation was assessed via Ki-67.[12] To profile metabolic fitness, GLUT1 and GAPDH were included, both upregulated during glycolysis. GLUT1 reflects increased glucose transport upon T cell activation, [13] and intracellular GAPDH serves as an alternative marker of glycolytic flux.[14] Hif-1a was used to evaluate potential shifts to anaerobic metabolism.[15] Exhaustion markers included PD1, TIM3, and LAG3 (**Figure S15**).[16] CD36, a fatty acid transporter linked to lipid stress and dysfunction in $CD8^+$ tumor-infiltrating lymphocytes, was included to assess metabolic impairment.[17] CD57 and active caspase 3 were used to identify senescent and apoptotic populations, respectively (**Figure 1**). [18,19]

Functionality Assessment

A dual-fluorescent protein cytotoxicity readout was integrated directly into the phenotyping assay. CAR T cells were co-cultured with $CD19^+$ Nalm6-YFP^+ target and $CD19^-$ Nalm6-GFP^+ control cells. YFP^+/GFP^+ quantification provided a killing index for antigen-specific cytotoxicity (analogous to a VITAL assay).[20] Granzyme B and perforin were also measured intracellularly to evaluate effector function (**Figure S15**). Degranulation inhibitors were not used during co-culture to preserve physiological function and marker expression.

Engineering Readouts

To assess product identity, the panel included markers relevant to CAR T cell engineering. HLA class I (A, B, C) and class II (DP, DQ, DR) molecules are genes of interest for allogeneic products. Further, HLA class

II can also be used as an activation marker. CD3 and CAR markers allow for monitoring of TCR knockout, and CAR editing efficiency, respectively.[21]

Gating Strategy and Controls

Dead cells and debris were excluded using Zombie NIR viability dye. CD45 served as a pan-leukocyte marker to include T cells and exclude non-hematopoietic events such as Nalm6 cells. Sequential gating on viable singlets, $GFP^{-}YFP^{-}$, and $CD45^{+}$ lymphocytes ensured a clean T cell population for downstream analysis. Due to the complexity of the 36-parameter panel on a 4-laser, 48-channel spectral cytometer, fluorochrome selection, and gating hierarchy were guided by evaluating spectral similarity, signal spread, and stain index values (**Figures S18 and S19**). Fluorescence-minus-one (FMO) controls were generated for each marker (**Figure S11**), and all gates were validated using fully stained reference samples. Staining was verified across manufacturing days using internal positive controls (**Figure S3**).

Panel Optimization

Each antibody was titrated using serial dilutions (typically 1:20 to 1:320), and the lowest concentration with optimal stain index and intensity was selected. Multiple optimization rounds were performed to adjust for fluorochrome interactions in the full master mix. This was critical in a high-dimensional context, where minor shifts in intensity could affect rare subset detection. Protocol refinements included sequential (pre-stain) staining for improved resolution of CXCR3 (**Figure S20A**), higher antibody volumes for CD69 (**Figure S20B**), and lower antibody volumes for CD25 to accommodate activation-induced expression changes (**Figure S20C**).

Spectral Overlap and Unmixing Performance

Spectral overlap was addressed using rigorous controls. FMO controls were acquired for all markers to enable accurate gating in the presence of full spectral complexity (**Figure S11**). This was particularly important for dim or partially overlapping fluorochromes such as GLUT1 Alexa Fluor 647 and IRF4 APC, which may be modulated by antigen engagement. For low-abundance targets, single-stain compensation beads were used, and bead- versus cell-derived spectral signatures were compared to quantify spectral differences (**Figure S21**).

Overall staining performance was evaluated by calculating stain index values for each marker in fully stained versus single-color conditions (**Figure S22**). To understand the influence of spread on fluorochrome resolution, we assessed the robust Standard Deviation (rSD) and stain indices of single-color controls when unmixed against the single color or the entire 36-color unmixing matrix. Fluorescent spread (rSD) of some markers (VioGreen, VioBlue, PerCP-Cy5.5) exhibited a 4- to 6-fold increase in the standard deviation of negative populations when unmixed with the 36-color matrix (**Figure S23**), highlighting the importance of careful marker to fluorophore matching. Spillover spreading matrices (**Figure S24**) were further used to guide fluorochrome-marker pairing, to minimize the impact of spread in co-expressed targets. For example, CXCR5 was placed on PE-Cy7 despite potential spread from RB780 (Ki-67) and PE-Cy5 (CXCR3). This was acceptable because, at day 0, Ki-67 expression is low and $CXCR5^{+}$ Tfh cells do not co-express CXCR3. By day 5, when Ki-67 is elevated, $CXCR5^{+}$ Tfh cells are rare (**Figure S25**). Despite the complexity of the panel, minimal manual compensation adjustments were required post-unmixing, with moderate spectral spread considered acceptable given the high dimensionality of the panel (**Figure S12**).

Panel Modularity

Panel flexibility was demonstrated by substituting GLUT1 with GAPDH in one donor sample, without impacting marker expression patterns (**Figure S26**). This modularity supports future adaptation, such as integrating alternative metabolic probes or additional markers, without compromising dataset continuity.

Staining Protocol for the Full-Spectrum Flow Cytometry Panel

1. **Cell aliquots:** Count cells and aliquot 1E6 cells for each fully stained sample. For each control condition (unstained, Single Color (SC) Controls, or FMO), harvest ~1E5 cells. Transfer cells into FACS tubes.
2. **Wash and blocking:** Resuspend cells in 1 mL of BSA stain buffer and centrifuge at 300 × g for 5 min. Decant the supernatant and blot the tube on a paper towel to remove residual liquid. Next, add 10 μL of Fc blocking mix (containing 1 μL human Fc receptor blocking reagent [BD Bioscience, 564220] and 9 μL Brilliant Stain Buffer [BD Biosciences, 566349]) to each sample and incubate for 10 min at RT. *For the unstained control, add 10 μL of Brilliant Stain Buffer in place of the Fc block mix.*
3. **CAR detection:** Add 15 μL of the CAR detection mix (anti-FMC63 CAR antibody diluted in Brilliant Stain Buffer) to each tube and incubate for 10 min at RT in the dark. *For unstained, non-CAR SC, and FMO controls, add 15 μL of Brilliant Stain Buffer instead of the CAR antibody mix.*
4. **Pre-stain:** Add 25 μL of the pre-stain antibody master mix containing chemokine and chemoattractant receptor antibodies (CXCR3, CXCR5, CCR6, CCR7, CRTH2, in Brilliant Stain Buffer) to each sample. Incubate on ice for 30 min, protected from light. *For unstained/SC/FMO controls, add 25 μL of Brilliant Stain Buffer in place of the antibody master mix.*
5. **Extracellular staining:** Add 50 μL of the main extracellular antibody master mix (remaining surface markers and viability dye, all diluted in Brilliant Stain Buffer) to each tube. Incubate on ice for 30 min, protected from light. *For unstained/non-extracellular SC/FMO controls, add 50 μL of buffer instead of the antibody master mix.*
6. **Post-stain wash:** Add 1 mL of BSA buffer to each tube and centrifuge at 300 × g for 5 min. Decant the supernatant and gently blot the tube dry.
7. **Fixation/permeabilization:** Add 1 mL of Fixation/Permeabilization buffer (eBioscience™ Foxp3 / Transcription Factor Staining Buffer Set, [eBioscience Cat. 00-5523-00] prepared according to the manufacturer's instructions) to each sample. Incubate for 30 min at 4°C in the dark.
8. **Washes:** Wash cells twice with 1 mL of 1× Permeabilization buffer. For each wash, resuspend the cells in 1 mL 1× Permeabilization buffer, then centrifuge at 300 × g for 5 min and decant the supernatant.
9. **Intracellular staining:** Add 100 μL of the intracellular antibody master mix (all intracellular antibodies diluted in 1× Permeabilization buffer) to each tube. Incubate on ice for 30 min, protected from light. *For unstained/non-intracellular SC/FMO controls, add 100 μL of permeabilization buffer with no antibody.*
10. **Intracellular washes:** Wash cells two times with 1× Permeabilization buffer. For each wash, add 2 mL of 1× Permeabilization buffer, then centrifuge at 300 × g for 5 min. Decant the supernatant and blot dry between washes.
11. **Final resuspension:** Resuspend the stained cell pellet in ~200 μL of DPBS for data acquisition. Keep samples at 4°C and protected from light until acquisition.

Table S1. Composition and functional categorization of the 36-marker spectral flow cytometry panel for CAR T cell phenotyping.

This table lists all 36 markers used in the spectral panel, organized by functional category, the conjugated fluorochrome used for spectral detection, and the antibody clone.

Group	Marker	Function	Fluorochrome	Clone
Live/Dead	Viability	Discrimination of live/dead cells	Zombie NIR	N/A
Cell Identification	GFP	Track control cell % during killing assays	GFP	N/A
	YFP	Track target cell % during killing assays	YFP	N/A
	CD45	Hematopoietic lineage, target cell discrimination and gating backbone	cFluor V547	HI30
CAR Characterization	CAR	CAR expression detection	PE	REA1297
	HLA-A, B, C	KO gene of interest	PerCP	W6/32
	HLA-DP, DQ, DR	KO gene of interest, upregulation in activated T cells	Spark Blue 574	Tü39
T Cell Markers	CD3	T cell identification and purity verification	APC-Fire 750	UCHT1
	CD4	Helper T cell subset gating	cFluor R840	SK3
	CD8	Cytotoxic T cell subset gating	BV786	SK1
Cell Subtyping	CD56	NK and NKT cell identification	Super Bright 436	TULY56
	FoxP3	Regulatory T cell identification	RB613	259D/C7
T Cell Subtyping	CXCR3 (CD183)	Th1 or Tc1 subset identification ($CXCR3^{+}$)	PE-Cy5	G025H7
	CRTH2 (CD294)	Th2 or Tc2 subset identification ($IRF4^{+}$ $CRTH2^{+}$)	BV711	BM16
	IRF4	Associated with Th9- or Tc9-like phenotypes (IRF4+ CRTH2-CCR6-CD161-)	APC	REA201
	CD161	Th17 or Tc17 subset identification ($IRF4^{+}$ $CRTH2^{-}$ $CCR6^{+}CD161^{+}$)	RB705	HP-3G10
	CCR6 (CD196)	Th22 or Tc22 subset identification ($IRF4^{-}$ $CRTH2^{-}$ $CXCR5^{-}CCR6^{+}$)	BV421	G034E3
	CXCR5 (CD185)	Tfh- or Tfc-like cell identification ($IRF4^{-}CRTH2^{-}$ $CXCR5^{+}CCR6^{-}$)	PE-Cy7	J252D4
Stemness	CCR7 (CD197)	Naïve, stem cell memory and central memory T cell marker	Spark NIR 685	G043H7
	CD45RA	Naïve, stem cell memory and terminal effector T cell marker	BV570	HI100

	CD95 (Fas)	Stem cell memory subset marker within the CD45RA+CCR7+ repertoire	PE-Fire 700	DX2
	CD27	Differentiation subtyping T cell marker	BV750	O323
	CD28	Differentiation subtyping T cell marker	BV605	CD28.2
Activation	CD69	Early activation marker, upregulated upon stimulation	BV480	FN50
	CD25	IL-2 receptor alpha, mid- and late activation marker	PE-Fire 744	M-A251
Exhaustion	PD1 (CD279)	Checkpoint molecule involved in T cell exhaustion (target of anti-PD1 therapy)	PE-Fire 640	EH12.2H7
	TIM3 (CD366)	Checkpoint molecule involved in T cell exhaustion	PE-Fire 810	F38-2E2
	LAG3 (CD223)	Checkpoint molecule involved in T cell exhaustion	PE-CF594	T47-530
Senescence & Proliferation	CD57	Senescence marker	PerCP-Cy5.5	QA17A04
	Active Caspase 3	Apoptosis marker	BV650	C92-605.rMAb
	Ki-67	Proliferation marker indicating active cell cycle	RB780	B56
Metabolism	GLUT1 or GAPDH	Glucose transporter and glycolytic enzyme, metabolic activation indicator	Alexa Fluor 647	EPR3915 or EPR16891
	Hif-1a	Hypoxia-induced metabolic regulator	Alexa Fluor 700	H1alpha67
	CD36	Fatty acid transporter linked to T cell dysfunction in tumors	VioBlue	REA760
Effector Function	Granzyme B	Cytotoxic effector molecule involved in target cell apoptosis	RB744	GB11
	Perforin	Cytotoxic effector molecule involved in granule-mediated apoptosis	VioGreen	REA1061

Table S2. Suggested condensed flow cytometry panel for GMP manufacturing and release.

A streamlined subset of the full 36-marker panel, designed to support key phenotypic characteristics for GMP manufacturing and routine quality control.

Marker	Justification
**Zombie NIR**	Live/dead discrimination (vital release criteria).
**CD45**	Pan-leukocyte marker used as main marker for $CD3^+$ cell identification.
**CD3**	Confirms T cell lineage and overall T cell purity.
**CD4**	Determines helper T cell subset proportion (CD4/CD8 ratio).
**CD8**	Determines cytotoxic T cell subset proportion (CD4/CD8 ratio).
**CAR**	Measures editing efficiency, a core release metric.
**CCR7**	(Pairs with CD45RA) Tracks differentiation trajectories (T_{N}/T_{SCM}, T_{CM}, T_{EM}, T_{TE}).
**CD45RA**	(Pairs with CCR7) Tracks differentiation trajectories (T_{N}/T_{SCM}, T_{CM}, T_{EM}, T_{TE}).

Table S3. Antibody titration cell sources.

This table summarizes antibody titration conditions, including tested cell types and stimulation reagents used to optimize signal resolution for each marker.

Marker	Target Cell Type	Stimulation Condition
CXCR3 (CD183)	PBMCs	Resting
CD56	PBMCs	Resting
FoxP3	PBMCs	Resting
CD36	PBMCs	Resting
CCR6 (CD196)	Isolated T cells	Resting
CD45	Isolated T cells	Resting
CD45RA	Isolated T cells	Resting
CD28	Isolated T cells	Resting
CD161	Isolated T cells	Resting
CD27	Isolated T cells	Resting
CD8	Isolated T cells	Resting
HLA-A, B, C	Isolated T cells	Resting
CRTH2 (CD294)	Isolated T cells	Resting
CCR7 (CD197)	Isolated T cells	Resting
Viability	Isolated T cells	Live/dead stain
CD3	Isolated T cells	Resting
CD4	Isolated T cells	Resting
CD69	Isolated T cells	TransAct Activated (48h)
IRF4	Isolated T cells	TransAct Activated (48h)
HLA-DP, DQ, DR	Isolated T cells	TransAct Activated (72h)
CD57	Isolated T cells	TransAct Activated (72h)
Ki-67	Isolated T cells	TransAct Activated (72h)
LAG3 (CD223)	Isolated T cells	TransAct Activated (72h)
PD1 (CD279)	Isolated T cells	TransAct Activated (72h)
CD95	Isolated T cells	TransAct Activated (72h)
CD25	Isolated T cells	TransAct Activated (72h)
CXCR5 (CD185)	Isolated T cells	TransAct Activated (72h)
TIM3 (CD366)	Isolated T cells	TransAct Activated (72h)
GLUT1	Isolated T cells	TransAct Activated (72h)
GAPDH	Isolated T cells	TransAct Activated (72h)
Hif-1a	Isolated T cells	TransAct Activated (72h)
CAR	CAR^+ T cells	Cultured CAR^+ T cells
Perforin	CAR^+ T cells + Nalm6	Co-culture with $CD19^+$ target cells
Granzyme B	CAR^+ T cells + Nalm6	Co-culture with $CD19^+$ target cells
Active Caspase 3	CAR^+ T cells	Induced apoptosis

Table S4. Prepared buffers and master mixes for staining protocol.

This table lists the composition of all buffers and master mixes used throughout the spectral staining workflow.

Buffer/Master Mix	Components	Purpose
Fixation/Permeabilization Buffer	1:4 dilution of Fixation/Permeabilization concentrate to diluent solution	Fixation buffer
1× Permeabilization Buffer	1:10 dilution of 10× Permeabilization Buffer in cell culture water	Intracellular staining and wash buffer
Cell Culture Media (Option A)	RPMI + 10% FBS + 1% GlutaMAX	Standard CAR T and T cell culture medium
Cell Culture Media (Option B)	TexMACS + 3% hABS + 12.5 ng/mL IL-7 and IL-15	Standard CAR T and T cell culture medium
Fc Receptor Blocking Master Mix	Fc block reagent + Brilliant Stain Buffer	Reduce non-specific Fc receptor antibody binding
CAR Detection Master Mix	Anti-FMC63 CAR antibody + Brilliant Stain Buffer	CAR detection reagent
Pre-Stain Master Mix	CXCR5 (CD185), CCR6 (CD196), CRTH2 (CD294), CCR7 (CD197), CXCR3 (CD183) + Brilliant Stain Buffer	Chemokine and chemoattractant receptor staining before extracellular stain
Extracellular Master Mix	CD36, CD69, HLA-DP, DQ, DR, CD57, LAG3 (CD223), PD1 (CD279), CD95, CD25, TIM3 (CD366), Viability, CD56, CD45, CD45RA, CD28, CD161, CD27, CD3, CD8, HLA-A, B, C, CD4 + Brilliant Stain Buffer	Comprehensive extracellular staining panel
Intracellular Master Mix	Ki-67, Hif-1a, Perforin, Granzyme B, Active Caspase 3, GLUT1 or GAPDH, FoxP3, IRF4 + Permeabilization Buffer	Comprehensive intracellular staining panel

Table S5. Reference controls for spectral unmixing

This table summarizes reference controls for each marker, including fluorophore and sample type, and reports the MFI of the brightest 200 events to support accurate spectral unmixing.

Marker	Fluorochrome	Reference Control	
		Type	MFI of top >200 events
GLUT1	Alexa Fluor 647	Cells	1.07E+06
GAPDH			
Hif-1a	Alexa Fluor 700	Beads	2.59E+05
IRF4	APC	Cells	3.38E+05
CD3	APC-Fire 750	Cells	2.65E+05
CCR6 (CD196)	BV421	Beads	3.37E+05
CD69	BV480	Cells	6.67E+05
CD45RA	BV570	Cells	2.00E+05
CD28	BV605	Beads	5.39E+05
Active Caspase 3	BV650	Cells	4.76E+05
CRTH2 (CD294)	BV711	Beads	4.56E+05
CD27	BV750	Beads	3.89E+05
CD8	BV786	Cells	2.70E+06
CD4	cFluor R840	Cells	4.13E+05
CD45	cFluor V547	Cells	1.73E+05
GFP	GFP	Cells	1.55E+06
CAR	PE	Cells	4.18E+06
LAG3 (CD223)	PE-CF594	Beads	5.70E+05
CXCR3 (CD183)	PE-Cy5	Cells	1.10E+06
CXCR5 (CD185)	PE-Cy7	Beads	1.26E+06
PD1 (CD279)	PE-Fire 640	Cells	5.29E+05
CD95 (Fas)	PE-Fire 700	Beads	1.20E+06
CD25	PE-Fire 744	Cells	1.51E+06
TIM3 (CD366)	PE-Fire 810	Beads	4.31E+05
HLA-A, B, C	PerCP	Cells	1.59E+06
CD57	PerCP-Cy5.5	Cells	1.25E+06
FoxP3	RB613	Beads	3.60E+05
CD161	RB705	Beads	1.09E+06
Granzyme B	RB744	Cells	9.44E+05
Ki-67	RB780	Cells	8.11E+05
HLA-DP, DQ, DR	Spark Blue 574	Cells	1.28E+06
CCR7 (CD197)	Spark NIR 685	Beads	2.79E+05
CD56	Super Bright 436	Beads	1.76E+06
CD36	VioBlue	Cells	2.50E+05
Perforin	VioGreen	Cells	1.00E+06
YFP	YFP	Cells	9.81E+04
Viability	Zombie NIR	Cells	4.13E+05

Table S6. Statistical analysis of Th/Tc and T cell differentiation subsets during 24 h restimulation.

p-values from two-way repeated measures ANOVA with Tukey's multiple comparisons test (p = 0.05) are shown for $CD4^{+}$ helper (Th), $CD8^{+}$ cytotoxic (Tc), T_{N}, T_{SCM}, T_{CM}, T_{EM}, and T_{TE} subsets within CAR^{+} and CAR^{-} compartments across 0 h, 3 h, 12 h, 15 h, and 24 h. Data are presented separately for day 5 and day 10 products (n = 3 donors). Only Th/Tc subsets representing ≥0.5% of total $CD3^{+}$ T cells at any time point or in any donor are shown. AN, "all negative" ($CXCR3^{-}IRF4^{-}CRTH2^{-}CCR6^{-}CXCR5^{-}$). "NA" denotes comparisons with no significant difference between timepoints.

			Th / Tc Subsets												Memory & Differentiation Subsets						
Timepoint	CAR+/-	Comparison	Th1	Tc1	Th2	Tc2	Th9	Tc9	Th17	Th22	Tc22	Tfh	CD4+ AN	CD8+ AN	T_{N}	T_{SCM}	T_{CM}	Early T_{EM}	Early-like T_{EM}	Intermediate T_{EM}	T_{TE}
Day 5	CAR+	0h (baseline) vs. 3h (co-culture)	0.9937	0.0925	0.3862	0.8185	0.8356	0.7795	0.2631	0.1335	0.7600	0.8527	0.0243	0.4268	0.5985	0.9993	0.7132	0.5068	0.8429	0.8448	0.9295
		0h (baseline) vs. 12h (+9h co-culture)	0.8731	0.9998	0.5976	0.8368	0.9931	0.9793	0.9995	0.0750	0.7866	0.9883	0.4127	0.1258	0.6467	0.0457	0.9959	0.5133	0.8647	0.7911	0.9833
		0h (baseline) vs. 15h (+3h restim)	>0.9999	0.5066	0.5526	0.8596	0.3864	0.7991	0.7565	0.7288	0.4977	>0.9999	0.7813	0.0421	0.5313	0.2816	0.8743	0.4446	0.8575	0.8680	0.8085
		0h (baseline) vs. 24h (+12h restim)	0.8796	0.7894	0.4878	0.7832	0.3924	0.3622	0.5859	0.0106	0.3453	>0.9999	0.4309	0.1880	>0.9999	0.6405	0.1348	0.4870	0.9998	>0.9999	0.6953
		0h (baseline) vs. 24h (n.r.)	0.1355	>0.9999	0.3409	0.7833	0.5256	0.7825	0.4278	0.7538	0.9940	0.6089	0.6160	0.9500	0.9807	0.4331	0.4671	0.0560	0.1580	0.9488	0.8895
		3h (co-culture) vs. 12h (+9h co-culture)	0.8467	0.9297	0.8401	0.8543	0.8182	0.2798	0.5931	0.9993	0.9909	0.9987	>0.9999	>0.9999	0.7604	0.9415	0.9833	0.9978	0.9991	0.9099	0.9849
		3h (co-culture) vs. 15h (+3h restim)	0.9997	0.6977	0.8485	0.8891	0.9788	0.8142	0.4863	0.8778	0.9996	0.9851	0.9998	>0.9999	0.5733	0.4084	0.8108	0.3396	0.8431	0.9926	0.9684
		3h (co-culture) vs. 24h (+12h restim)	0.6680	>0.9999	0.5979	0.8374	0.1100	0.9998	0.4458	0.9867	0.8996	0.9894	0.9983	0.2288	0.7397	0.0055	0.0387	0.1280	0.9454	0.1205	0.3819
		3h (co-culture) vs. 24h (n.r.)	0.1079	0.9381	0.3467	0.8027	0.1379	0.7790	0.3685	0.4730	0.7942	0.5776	0.3792	0.8169	0.7448	0.1282	0.5333	0.1029	0.4109	0.0133	>0.9999
		12h (+9h co-culture) vs. 15h (+3h restim)	0.7429	0.2586	0.5258	0.9760	0.6259	0.6928	0.7370	0.6315	0.7491	0.8356	0.9751	0.9998	0.6869	0.1157	0.6558	0.2705	0.6016	0.5316	0.6467
		12h (+9h co-culture) vs. 24h (+12h restim)	0.0558	0.9911	0.8180	0.8475	0.3260	0.9998	0.6443	0.5702	0.3103	0.9417	0.7936	0.5087	0.7630	0.3195	0.2158	0.4649	0.9955	0.5695	0.4465
		12h (+9h co-culture) vs. 24h (n.r.)	>0.9999	>0.9999	0.4136	0.8237	0.9045	0.8683	0.4611	0.3142	0.0545	0.3207	0.1668	0.8103	0.7538	0.1586	0.4736	0.0147	0.3487	0.2514	0.9168
		15h (+3h restim) vs. 24h (+12h restim)	0.8454	0.9207	0.6115	0.8720	0.8991	0.9309	0.5900	0.5607	0.1149	>0.9999	0.5748	0.6208	0.7178	0.9965	0.3271	>0.9999	0.9939	0.5881	0.0870
		15h (+3h restim) vs. 24h (n.r.)	0.8059	0.7864	0.4559	0.8443	0.4256	0.7928	0.4017	0.2066	0.3152	0.2291	0.0774	0.7646	0.7276	0.9556	0.5596	0.1559	0.4439	0.1864	0.9228
		24h (+12h restim) vs. 24h (n.r.)	0.3069	0.1294	0.2956	0.7734	0.0708	0.4949	0.2982	0.3234	0.1399	0.2530	0.1184	0.7359	0.7637	>0.9999	0.2535	0.4039	0.7320	0.3834	0.4650
	CAR-	0h (baseline) vs. 3h (co-culture)	0.9059	0.9385	0.3255	0.7313	0.3691	0.4698	0.4629	0.6201	0.9007	0.9912	0.9698	0.9839	>0.9999	0.9991	0.4506	0.3633	0.8992	0.7728	0.9906
		0h (baseline) vs. 12h (+9h co-culture)	0.9391	0.9981	0.4277	0.7974	0.9461	0.9924	0.9791	0.1056	0.4582	0.9075	0.0858	0.3411	0.2827	0.1351	0.4255	0.0161	>0.9999	0.6192	0.0845
		0h (baseline) vs. 15h (+3h restim)	0.8348	0.2288	0.3999	0.2988	0.7592	>0.9999	0.4687	0.2829	0.2476	0.7120	0.9853	0.6525	0.2942	0.8708	0.1249	0.2697	0.6285	0.6760	0.2063
		0h (baseline) vs. 24h (+12h restim)	0.3155	0.9760	0.9977	0.6729	0.1790	0.5267	0.8142	0.1221	0.3002	0.0208	0.9998	0.6414	0.9576	0.3626	0.8264	0.0624	>0.9999	0.6853	0.0623
		0h (baseline) vs. 24h (n.r.)	0.4588	0.2676	0.0956	0.5168	0.3596	0.7071	0.3240	0.9990	0.5179	0.5507	0.3075	0.9996	0.7467	0.3809	0.3915	0.5791	0.9990	0.9375	0.4602
		3h (co-culture) vs. 12h (+9h co-culture)	0.4550	0.1669	0.7204	0.9267	0.9671	0.7226	0.7889	0.5292	0.8385	0.8563	>0.9999	0.8329	0.2317	0.8880	0.0811	0.2889	0.8298	0.6228	0.3794
		3h (co-culture) vs. 15h (+3h restim)	0.8194	0.6378	0.2880	0.8122	0.4383	0.5296	0.4560	0.9992	0.9904	0.7784	0.9758	0.9998	0.2173	0.9720	0.0854	0.9999	0.7117	0.7113	0.4059
		3h (co-culture) vs. 24h (+12h restim)	0.5103	0.3258	0.1501	0.8093	0.0555	0.9964	0.6266	0.3172	0.3421	0.9998	0.9702	0.2015	0.7360	0.6313	0.9993	0.4479	0.9927	0.6915	0.1756
		3h (co-culture) vs. 24h (n.r.)	0.9789	0.2141	0.0692	0.6455	0.3220	0.5490	0.3545	0.6722	0.9835	0.3995	0.5348	0.8775	0.7593	0.8521	0.3864	0.3296	0.9929	0.9833	0.7030
		12h (+9h co-culture) vs. 15h (+3h restim)	>0.9999	0.9329	0.5438	0.8534	0.8474	0.9975	0.7144	0.6552	0.9965	0.4680	0.7173	0.6331	0.3413	0.2468	0.9998	0.1248	0.7831	0.4378	0.9053
		12h (+9h co-culture) vs. 24h (+12h restim)	0.3541	0.8356	0.5023	0.8586	0.5747	0.3811	0.9997	0.2926	0.1038	0.3947	0.9905	0.9984	0.6775	0.0755	0.3195	0.3964	0.9999	0.5859	0.8518
		12h (+9h co-culture) vs. 24h (n.r.)	0.5918	0.3570	0.2535	0.7152	0.5781	0.8659	0.3397	0.3225	0.1463	0.4239	0.2599	0.7207	0.0557	0.0121	>0.9999	0.1234	0.9997	0.0624	>0.9999
		15h (+3h restim) vs. 24h (+12h restim)	0.1171	0.9765	0.9299	0.9948	0.2330	0.6134	0.3714	0.5951	0.6192	0.3317	0.9977	0.8222	0.7291	0.9932	0.3949	0.7978	0.9728	0.7405	>0.9999
		15h (+3h restim) vs. 24h (n.r.)	0.6487	0.9750	0.0508	0.4629	0.0746	0.6047	0.1622	0.4987	0.9972	0.4999	0.2870	0.9795	0.2000	0.9972	>0.9999	0.1273	0.5163	0.3129	0.8960
		24h (+12h restim) vs. 24h (n.r.)	0.5334	0.4731	0.1482	0.5051	0.1996	0.6096	0.2084	0.3147	0.0574	0.4150	0.6690	0.6028	0.9978	0.9828	0.5334	0.2700	0.9988	>0.9999	0.9928
Day 10	CAR+	0h (baseline) vs. 3h (co-culture)	0.1954	0.8128	0.1895	0.4122	0.5030	0.5115	0.5774	0.5521	0.4424	0.9070	0.8433	0.8262	NA	0.8596	0.4590	0.9941	0.9969	0.5557	0.7458
		0h (baseline) vs. 12h (+9h co-culture)	0.0388	0.9998	0.1817	0.7316	<0.0001	0.2835	0.6825	0.4384	0.4816	0.3611	0.0218	0.0622	NA	>0.9999	0.3069	0.4503	0.3421	0.7115	0.4948
		0h (baseline) vs. 15h (+3h restim)	0.0165	0.9998	0.2056	0.6861	0.0715	0.1867	0.5947	0.4554	0.2278	0.3148	0.0360	0.1766	NA	0.8404	0.4529	0.4723	0.9635	0.9240	0.9746
		0h (baseline) vs. 24h (+12h restim)	0.0663	0.9855	0.3704	0.7388	0.1052	0.3426	0.7870	0.4371	0.1349	0.2520	0.0459	0.1398	NA	0.8421	0.3382	0.4682	0.9810	>0.9999	0.9934
		0h (baseline) vs. 24h (n.r.)	0.0627	0.9973	0.3089	0.7511	0.4609	0.4177	0.8727	0.4207	0.6133	0.2744	0.0391	0.1802	0.7197	0.8863	0.2927	0.4788	0.1254	0.0436	0.9732
		3h (co-culture) vs. 12h (+9h co-culture)	0.0408	0.2511	0.4486	0.9193	0.9989	>0.9999	0.9580	0.6764	0.7918	0.6311	0.4319	0.4942	NA	0.4027	0.2858	0.2314	0.0067	0.2549	0.9839
		3h (co-culture) vs. 15h (+3h restim)	0.0114	0.4734	0.3480	0.8358	0.9994	0.9499	0.8448	0.6205	0.3524	0.4256	0.2825	0.4117	NA	0.9652	0.4523	0.3623	>0.9999	0.3151	0.8348
		3h (co-culture) vs. 24h (+12h restim)	0.0694	0.5545	0.9978	0.8960	0.9614	0.8304	0.8843	0.5415	0.3138	0.3274	0.1966	0.3206	NA	0.6474	0.3351	0.4198	>0.9999	0.9392	0.3902
		3h (co-culture) vs. 24h (n.r.)	0.0650	0.2021	0.6071	0.9253	0.8612	>0.9999	0.5984	0.6080	0.9987	0.3316	0.2853	0.4558	0.7197	0.9997	0.2864	0.4944	0.6260	0.7046	0.3047
		12h (+9h co-culture) vs. 15h (+3h restim)	0.2506	0.5351	0.2839	0.4901	0.9981	0.5856	0.9013	0.6435	0.7371	0.2814	0.2318	0.7939	NA	0.1461	0.9641	0.9403	0.5818	0.9243	>0.9999
		12h (+9h co-culture) vs. 24h (+12h restim)	0.1274	0.5307	0.0153	0.8380	0.1527	0.4123	0.5917	0.4698	0.8501	0.0964	0.2555	0.7650	NA	0.3646	0.3623	0.5943	0.4889	0.3090	0.8197
		12h (+9h co-culture) vs. 24h (n.r.)	0.0947	0.9091	0.1204	0.7235	0.1478	0.1509	0.5644	0.7888	0.0091	0.0540	0.3233	0.9964	0.7197	0.4960	0.3288	0.8916	0.1017	0.2982	0.6913
		15h (+3h restim) vs. 24h (+12h restim)	0.4671	0.9669	0.0848	0.9998	0.4585	0.7481	0.4087	0.2756	0.9842	0.0149	0.4097	>0.9999	NA	0.8931	0.3165	0.4962	0.9985	0.9253	0.2263
		15h (+3h restim) vs. 24h (n.r.)	0.3758	0.4278	0.1712	0.6635	0.3365	0.4593	0.4247	0.8092	0.2786	0.2337	0.1743	0.2271	0.7197	0.9780	0.2747	0.8673	0.9031	0.4652	0.1937
		24h (+12h restim) vs. 24h (n.r.)	0.8730	0.6685	0.4451	0.7341	0.1423	0.3256	0.5534	0.5375	0.4730	0.4817	0.2520	0.0456	0.7197	0.9998	0.4280	0.5757	0.3378	0.7926	0.8159
	CAR-	0h (baseline) vs. 3h (co-culture)	0.2826	0.9774	0.2836	0.3918	0.7436	0.7486	0.5314	0.5684	0.5833	0.3889	0.7797	0.9903	0.1207	0.8712	0.9933	0.3012	0.0309	0.0536	0.7879
		0h (baseline) vs. 12h (+9h co-culture)	0.9471	0.5302	0.0142	0.6905	0.0469	0.4382	0.7677	0.2768	0.5155	0.7250	0.8910	0.7478	0.8948	0.9991	0.5672	0.4760	0.0211	0.9257	0.9963
		0h (baseline) vs. 15h (+3h restim)	0.6103	0.8547	0.4491	0.6744	0.1112	0.4719	>0.9999	0.1318	0.5934	>0.9999	0.3120	0.7520	0.8383	0.7027	0.9997	0.4353	0.0289	0.7411	0.5607
		0h (baseline) vs. 24h (+12h restim)	0.7906	0.9274	0.5251	0.7714	0.1992	0.4003	0.9391	0.1804	0.4356	0.3267	0.1757	>0.9999	0.0085	0.4334	>0.9999	0.3782	0.0538	>0.9999	0.3369
		0h (baseline) vs. 24h (n.r.)	0.4089	0.4345	>0.9999	0.8095	>0.9999	0.9970	0.5099	0.0728	0.6506	0.1128	0.7507	0.5852	0.8148	0.1423	>0.9999	0.3177	0.2091	0.6713	0.5727
		3h (co-culture) vs. 12h (+9h co-culture)	0.1145	0.1527	0.7743	0.9195	0.9156	0.8576	0.5877	0.6014	0.5064	0.0239	0.9407	0.6363	0.7227	0.1815	0.5673	0.6189	0.2108	0.5887	0.7223
		3h (co-culture) vs. 15h (+3h restim)	0.0307	0.2487	0.7378	0.8137	0.9398	0.9624	0.6762	>0.9999	0.8989	0.5090	>0.9999	0.7813	0.6639	>0.9999	>0.9999	0.5987	0.8866	0.2423	>0.9999
		3h (co-culture) vs. 24h (+12h restim)	0.5770	>0.9999	0.9999	0.9711	0.9723	>0.9999	0.8342	0.8826	0.6074	0.9031	0.9580	0.9943	0.0263	0.1701	0.9989	0.5079	0.2055	0.6304	0.9985
		3h (co-culture) vs. 24h (n.r.)	0.0286	0.1346	0.5223	0.5070	0.7950	0.8076	0.5171	0.9997	0.8108	0.9768	0.9932	0.6139	0.6318	0.0408	0.9997	0.4649	0.9940	0.8969	0.9998
		12h (+9h co-culture) vs. 15h (+3h restim)	0.9794	>0.9999	0.8253	0.6615	0.9401	0.7627	0.6591	0.8294	0.8705	0.8832	0.6139	0.9982	0.1982	0.9210	0.1952	>0.9999	0.3128	>0.9999	0.2738
		12h (+9h co-culture) vs. 24h (+12h restim)	0.0606	0.1108	0.7546	0.0208	0.8194	0.5195	0.6072	0.9997	0.8765	0.5597	0.9997	0.0043	0.2590	0.0109	0.0372	0.6078	>0.9999	0.6031	0.0118
		12h (+9h co-culture) vs. 24h (n.r.)	0.0336	0.6747	0.0696	0.6314	0.2849	0.4547	0.6106	0.7696	0.7929	0.2317	0.9911	0.9939	0.9604	0.0264	0.4596	>0.9999	0.2321	0.4889	0.0811
		15h (+3h restim) vs. 24h (+12h restim)	0.1341	0.1735	0.4377	0.5487	0.5705	0.3415	0.5637	0.4825	0.9956	0.8763	0.4552	0.2858	0.2337	0.7455	0.9829	0.0369	0.5171	0.2569	0.8779
		15h (+3h restim) vs. 24h (n.r.)	0.5861	0.5138	0.4166	0.6415	0.2644	0.3564	0.6335	0.9219	0.9809	0.2659	0.1685	>0.9999	>0.9999	0.3166	0.9593	0.9999	>0.9999	0.4234	0.9653
		24h (+12h restim) vs. 24h (n.r.)	0.0056	0.0201	0.3754	0.7079	0.2916	0.3244	0.6059	0.6772	0.9990	0.8410	0.9956	0.2362	0.2057	0.3276	>0.9999	0.6785	0.6174	0.7920	0.9972

Table S7. Statistical analysis of phenotypic and functional markers, and Treg/NK-like T cell subsets during 24 h restimulation.

p-values from two-way repeated measures ANOVA with Tukey's multiple comparisons test (p = 0.05), comparing the expression activation, checkpoint, apoptotic, senescence, cytotoxic, proliferative, and metabolic markers (CD25, CD69, HLA-I, HLA-II, PD1, TIM3, LAG3, CD36, active caspase 3, CD57, granzyme B, perforin, Hif-1a, GLUT1, Ki-67), as well as Treg and NK-like T cell subsets in CAR$^+$ and CAR$^-$ compartments across 0 h, 3 h, 12 h, 15 h, and 24 h. Data are presented separately for day 5 and day 10 products (n = 3 donors). "NA" denotes comparisons with no significant difference between timepoints.

			Activation, Checkpoint, Apoptotic & Senescence Markers										Cytotoxic Function		Metabolic & Proliferative Markers			Treg & NK T		
Timepoint	CAR+/-	Comparison	CD25	CD69	HLA-I	HLA-II	PD1	TIM3	LAG3	CD36	Active Caspase 3	CD57	Granzyme B	Perforin	Hif-1a	GLUT1	Ki-67	CD8+ Treg	CD4+ Treg	NK T
Day 5	CAR+	0h (baseline) vs. 3h (co-culture)	>0.9999	0.0201	0.0692	0.3612	0.0419	0.8314	0.0270	0.2762	0.2117	0.7201	0.9966	0.1679	0.7002	0.1166	0.2047	0.7422	0.9349	0.9210
		0h (baseline) vs. 12h (+9h co-culture)	0.1294	0.3340	0.1207	0.9947	0.0210	0.0359	0.0637	0.0514	0.4670	0.4787	0.0579	0.1523	0.0753	0.1351	0.6280	0.6308	0.1066	0.7628
		0h (baseline) vs. 15h (+3h restim)	0.1294	0.0140	0.0536	0.7248	0.2749	0.2370	0.0613	0.0477	0.1229	0.9740	0.0475	0.6701	0.4083	0.0171	0.2377	0.5860	0.2595	0.9818
		0h (baseline) vs. 24h (+12h restim)	0.1294	0.0405	0.9856	0.3585	0.0706	0.9995	0.0965	0.0154	0.2094	0.9937	0.1961	0.4632	<0.0001	0.0100	0.2827	0.5595	0.1644	0.9737
		0h (baseline) vs. 24h (n.r.)	0.9996	0.7840	0.3790	0.0516	0.2214	0.1970	0.0302	0.3548	0.0591	0.7497	0.1062	0.1466	0.9741	0.0582	0.4386	0.3102	0.2509	0.9985
		3h (co-culture) vs. 12h (+9h co-culture)	0.5647	0.5257	0.8866	0.9736	0.6885	0.4591	0.0957	0.0363	0.5117	0.7748	0.0324	<0.0001	0.9778	0.1625	0.3476	0.6008	0.1052	0.6207
		3h (co-culture) vs. 15h (+3h restim)	0.5647	0.0037	0.8866	0.9770	0.9412	0.8471	0.0786	0.0694	0.1727	0.8621	0.0095	0.0522	0.9989	0.0245	0.0899	0.5451	0.3554	0.9992
		3h (co-culture) vs. 24h (+12h restim)	0.5647	>0.9999	0.7616	0.4491	0.9729	0.9787	0.1476	0.0162	0.2149	>0.9999	0.1799	0.0825	0.3268	0.0130	0.0938	0.4825	0.2581	0.8245
		3h (co-culture) vs. 24h (n.r.)	0.9997	0.0024	0.2219	0.9990	0.0808	0.0191	0.0740	0.8517	0.0283	0.6320	0.0324	0.0458	0.7962	0.0484	0.0923	0.1713	0.3063	0.7748
		12h (+9h co-culture) vs. 15h (+3h restim)	NA	0.1693	0.8866	0.9664	>0.9999	0.3457	0.9896	0.9133	0.5935	0.6802	0.0688	0.4185	0.0156	0.7941	0.9999	0.3720	0.8657	0.4776
		12h (+9h co-culture) vs. 24h (+12h restim)	NA	0.4056	0.5753	0.9228	0.2092	0.3283	0.5166	0.9999	0.9167	0.9999	0.2977	0.9678	0.0464	0.5023	0.8414	0.9524	0.9314	0.4542
		12h (+9h co-culture) vs. 24h (n.r.)	0.7748	0.2941	0.1979	0.8896	0.0079	0.6976	0.5614	0.3086	0.6785	0.3947	0.0428	>0.9999	0.0082	0.0587	0.9829	0.3586	0.5110	0.6142
		15h (+3h restim) vs. 24h (+12h restim)	NA	0.0272	0.6688	0.9995	0.2166	0.8028	0.5792	0.9956	0.2388	0.5651	0.2358	0.6379	0.0335	0.0448	0.3926	0.6559	0.7703	0.5244
		15h (+3h restim) vs. 24h (n.r.)	0.7748	0.0013	0.2003	0.9951	0.0427	0.2749	0.6731	0.1293	0.9886	0.9996	0.1677	0.5428	0.0240	0.5312	0.0852	0.9868	0.2407	0.8705
		24h (+12h restim) vs. 24h (n.r.)	0.7748	0.0050	0.9040	0.9803	0.0088	0.3721	0.9270	0.1994	0.2759	0.3947	0.2185	0.8049	0.0174	0.8602	0.1730	0.0118	0.3689	0.8333
	CAR-	0h (baseline) vs. 3h (co-culture)	0.8233	0.8291	0.9725	0.9331	0.9996	0.8328	0.1287	0.9992	0.7518	0.9220	0.6812	0.1001	0.6898	0.9857	0.1003	0.8765	0.7160	0.8899
		0h (baseline) vs. 12h (+9h co-culture)	0.4672	0.4698	0.2387	0.1514	0.1114	0.0438	0.0279	0.2684	0.5367	0.9997	0.0695	0.0165	0.1108	0.7884	0.9055	0.5911	0.2245	0.8372
		0h (baseline) vs. 15h (+3h restim)	0.0748	0.1315	0.2177	0.1717	0.0687	0.1008	0.0225	0.6035	0.1169	0.9920	0.2022	0.0133	0.5874	0.7648	0.2389	0.3906	0.0164	<0.0001
		0h (baseline) vs. 24h (+12h restim)	0.3371	0.9864	0.8866	0.8103	0.0265	0.4409	0.2100	0.1723	0.3724	0.9897	0.2840	0.6157	0.0089	0.9751	0.4306	0.3578	0.5191	0.9922
		0h (baseline) vs. 24h (n.r.)	0.6552	0.1276	0.0768	0.9486	0.0764	0.4553	0.2517	0.6408	0.3342	0.0003	0.2470	0.3237	0.9288	0.9038	0.9939	0.1797	0.3503	0.0912
		3h (co-culture) vs. 12h (+9h co-culture)	>0.9999	0.0406	0.0618	0.0298	0.1077	0.0946	0.0338	0.2984	0.4567	0.8801	0.0204	<0.0001	0.9079	0.5985	0.6707	0.8519	0.7245	0.0311
		3h (co-culture) vs. 15h (+3h restim)	0.0780	0.2094	0.3579	0.0923	0.1613	0.1934	0.0310	0.6232	0.1891	0.8917	0.0125	0.0012	0.9988	0.5315	0.0269	0.7081	0.2525	0.9565
		3h (co-culture) vs. 24h (+12h restim)	0.3161	0.1601	0.9921	0.6330	0.0792	0.3392	0.4866	0.1085	0.2714	0.8920	0.1778	0.2061	0.4991	0.9259	0.1169	0.8906	0.9574	0.2984
		3h (co-culture) vs. 24h (n.r.)	0.7004	0.0100	0.2250	0.7621	0.0169	0.4102	0.3661	0.6687	0.1769	0.5549	0.0563	0.1167	0.7638	0.8250	0.1820	0.3908	0.2499	0.5526
		12h (+9h co-culture) vs. 15h (+3h restim)	0.0622	0.0925	0.3693	>0.9999	0.4218	0.2628	0.3143	>0.9999	0.8755	0.9942	0.0274	0.0803	<0.0001	0.8940	0.9953	0.9946	0.9934	0.7566
		12h (+9h co-culture) vs. 24h (+12h restim)	0.3513	0.4009	0.2887	0.9991	0.0928	0.2715	0.0080	0.5359	0.0205	0.9962	0.9945	0.8916	0.3118	0.8545	0.2926	0.8129	0.1868	0.7616
		12h (+9h co-culture) vs. 24h (n.r.)	0.8433	0.4180	0.0706	0.8909	0.1006	0.1385	0.5183	0.0256	>0.9999	0.0612	0.0008	>0.9999	0.0066	0.9983	0.8274	0.2001	0.9992	>0.9999
		15h (+3h restim) vs. 24h (+12h restim)	0.7348	0.1192	0.2706	0.9988	0.5756	0.9892	0.0067	0.8470	0.5909	0.9938	0.3287	0.6678	0.0557	0.9945	0.2280	0.4445	0.0692	0.9513
		15h (+3h restim) vs. 24h (n.r.)	0.2431	0.0151	0.0629	0.8887	0.8058	0.9433	0.6689	0.5809	0.6516	0.6259	0.3701	0.9576	0.0476	0.9910	0.5056	0.2237	>0.9999	0.0659
		24h (+12h restim) vs. 24h (n.r.)	0.1933	0.1365	0.1252	0.7679	0.9969	0.9046	0.2959	0.9916	0.8036	0.1213	0.3340	0.4496	0.0098	0.3599	0.0337	0.1749	0.5342	0.9161
Day 10	CAR+	0h (baseline) vs. 3h (co-culture)	0.8814	0.0121	0.3865	0.5402	0.0024	0.1604	0.3830	0.2311	0.7175	0.9821	0.8381	0.1673	0.7552	0.3038	0.9996	0.3947	0.2181	0.5364
		0h (baseline) vs. 12h (+9h co-culture)	0.8431	0.0402	0.3258	0.4805	0.0558	0.1729	0.2041	0.0896	0.3177	0.2227	0.0875	0.9920	0.1986	0.0334	0.0086	0.2404	0.1870	0.2364
		0h (baseline) vs. 15h (+3h restim)	0.6506	0.0085	0.3560	0.0642	0.0404	0.9425	0.1373	0.2059	0.2456	0.3871	0.2072	0.5573	0.4903	<0.0001	0.4082	0.0690	0.1562	0.2234
		0h (baseline) vs. 24h (+12h restim)	0.6609	0.0123	0.3148	0.0077	0.0101	0.7699	0.0082	0.1709	0.7806	0.5385	0.0362	0.9996	0.1456	0.0254	0.5268	0.0877	0.1934	0.6174
		0h (baseline) vs. 24h (n.r.)	0.7474	0.0300	0.3894	0.1348	0.0433	0.3951	0.1105	0.0104	0.0061	0.3721	0.0089	0.9817	0.9864	0.0192	0.1843	0.1364	0.0453	0.7322
		3h (co-culture) vs. 12h (+9h co-culture)	0.7637	0.9611	0.2113	0.6421	0.1128	0.8792	0.1863	0.0892	0.2760	0.5090	0.1473	0.0506	0.6039	0.0560	0.5868	0.9917	0.9331	0.1816
		3h (co-culture) vs. 15h (+3h restim)	0.3172	0.0562	0.3748	0.9996	0.0573	0.4566	0.1193	0.2075	0.4448	0.6639	0.0697	0.0545	0.9106	0.0067	0.7760	>0.9999	0.5715	0.6919
		3h (co-culture) vs. 24h (+12h restim)	0.3410	0.0596	0.1919	0.9844	0.0113	0.0593	0.0002	0.1967	0.8346	0.5227	0.0672	0.0528	0.3409	0.0403	0.9419	>0.9999	0.8745	0.6681
		3h (co-culture) vs. 24h (n.r.)	0.5128	0.8415	0.7452	0.9777	0.0586	>0.9999	0.0954	0.0207	0.0450	0.9292	0.1261	0.0437	0.9236	0.0422	0.5427	0.1266	0.0681	0.8874
		12h (+9h co-culture) vs. 15h (+3h restim)	0.5111	0.3180	0.8866	0.0161	0.0976	0.2522	0.2088	0.3875	0.7866	0.6267	0.9346	0.1864	0.4031	0.0194	0.3347	0.9945	0.6085	0.0747
		12h (+9h co-culture) vs. 24h (+12h restim)	0.5996	0.3735	0.5302	0.6117	0.0158	0.1061	0.7003	0.3677	0.1544	>0.9999	0.1160	0.1985	0.1166	0.0322	0.1103	0.9998	0.9965	0.4318
		12h (+9h co-culture) vs. 24h (n.r.)	0.9514	0.9995	0.3999	0.5324	0.0472	0.9568	0.1657	0.7059	0.8649	0.1501	0.3925	>0.9999	0.0807	0.1155	0.0728	0.2329	0.0178	0.4774
		15h (+3h restim) vs. 24h (+12h restim)	0.3901	0.2193	0.6977	0.3095	0.0063	0.8656	0.9374	0.9772	0.9142	0.9606	0.1022	0.1866	0.0131	0.8093	0.0171	0.8687	0.2492	0.8470
		15h (+3h restim) vs. 24h (n.r.)	0.0070	0.2560	0.2400	0.2535	0.3473	0.6388	0.1387	0.9076	0.6625	0.6436	0.3822	0.5102	0.0377	0.4521	0.1346	0.1788	0.0056	0.9920
		24h (+12h restim) vs. 24h (n.r.)	0.0193	0.2751	0.6177	>0.9999	0.0444	0.2351	>0.9999	0.6565	0.4233	0.8312	0.0818	0.9586	0.0088	0.9593	0.3469	0.1770	0.0073	0.3024
	CAR-	0h (baseline) vs. 3h (co-culture)	<0.0001	0.1864	0.7352	0.6180	0.4526	0.2149	0.7407	0.6230	0.8495	>0.9999	0.7418	0.3770	0.9584	>0.9999	>0.9999	0.6852	0.6901	0.9123
		0h (baseline) vs. 12h (+9h co-culture)	0.1593	0.6363	0.4324	0.5959	0.0390	0.3699	0.1438	0.2378	0.2036	0.9999	>0.9999	>0.9999	0.2645	0.3973	0.8390	0.6884	0.5548	0.4577
		0h (baseline) vs. 15h (+3h restim)	0.1619	0.0272	0.3896	>0.9999	>0.9999	0.3803	0.1578	0.4672	0.1416	0.8351	0.9690	0.9961	0.9641	0.9955	0.3335	0.6909	0.4485	0.7185
		0h (baseline) vs. 24h (+12h restim)	0.0702	0.0217	0.6464	0.4790	0.4647	0.9998	0.2357	0.6263	0.2015	0.9918	0.9982	0.9996	0.2779	0.0644	0.0156	0.8294	0.5977	0.3954
		0h (baseline) vs. 24h (n.r.)	0.0719	0.0254	0.5809	0.2283	0.0406	>0.9999	0.2593	0.2625	0.1465	0.2523	0.9537	0.9999	0.6510	0.4800	0.6587	0.8096	0.3873	0.4760
		3h (co-culture) vs. 12h (+9h co-culture)	0.2453	0.2504	0.1145	0.2716	0.0112	0.3579	0.1318	0.2092	0.2168	>0.9999	0.3521	0.3122	0.7634	0.1547	0.9144	0.8419	0.3912	0.7121
		3h (co-culture) vs. 15h (+3h restim)	0.2498	>0.9999	0.0416	0.6358	0.9947	0.1109	0.1793	0.4655	0.3107	0.7587	0.4891	0.1512	>0.9999	0.9887	0.5229	0.7977	0.2542	0.7773
		3h (co-culture) vs. 24h (+12h restim)	0.0991	0.6209	0.3029	0.1515	0.2375	0.2962	0.4420	0.6251	0.1234	0.8689	0.2978	0.6463	0.7858	0.1734	0.0691	0.9766	0.9384	0.7778
		3h (co-culture) vs. 24h (n.r.)	0.0959	0.3395	0.5534	0.4129	0.0141	0.0730	0.3602	0.2837	0.1602	0.1374	0.3380	0.3217	0.7372	0.4412	0.7927	0.9403	0.7767	0.8686
		12h (+9h co-culture) vs. 15h (+3h restim)	>0.9999	0.2934	0.9995	0.4813	0.2168	>0.9999	0.4683	0.5938	0.3184	0.6977	0.0079	0.3819	0.1761	0.7707	0.9982	0.7359	0.5868	0.7046
		12h (+9h co-culture) vs. 24h (+12h restim)	0.9536	0.8511	0.3082	0.9998	0.8557	0.4770	0.9982	0.8844	0.3449	0.8767	0.2020	0.9267	0.9687	0.6266	0.5602	>0.9999	0.5401	0.9016
		12h (+9h co-culture) vs. 24h (n.r.)	0.7026	0.9978	0.1122	0.7764	0.0455	0.5416	0.9870	0.9997	0.5977	0.3027	0.3733	0.9839	0.0696	0.9995	0.8329	0.9831	0.9511	0.9986
		15h (+3h restim) vs. 24h (+12h restim)	0.1601	0.0999	0.1769	0.3362	0.0331	0.7744	0.8435	0.6331	0.9998	0.6000	0.0744	0.7239	0.0851	0.0887	0.4054	0.3598	0.2719	0.7798
		15h (+3h restim) vs. 24h (n.r.)	0.0053	0.0977	0.0389	0.9893	0.0921	0.3535	0.9940	0.5690	0.3041	0.1198	0.1917	0.6818	0.0283	0.0134	>0.9999	0.9998	0.7202	>0.9999
		24h (+12h restim) vs. 24h (n.r.)	0.3745	0.1694	>0.9999	0.6819	0.2743	0.9989	0.8111	0.7769	0.2307	0.3023	0.6439	0.9984	0.0398	0.5957	0.5051	0.7722	0.6489	0.3542

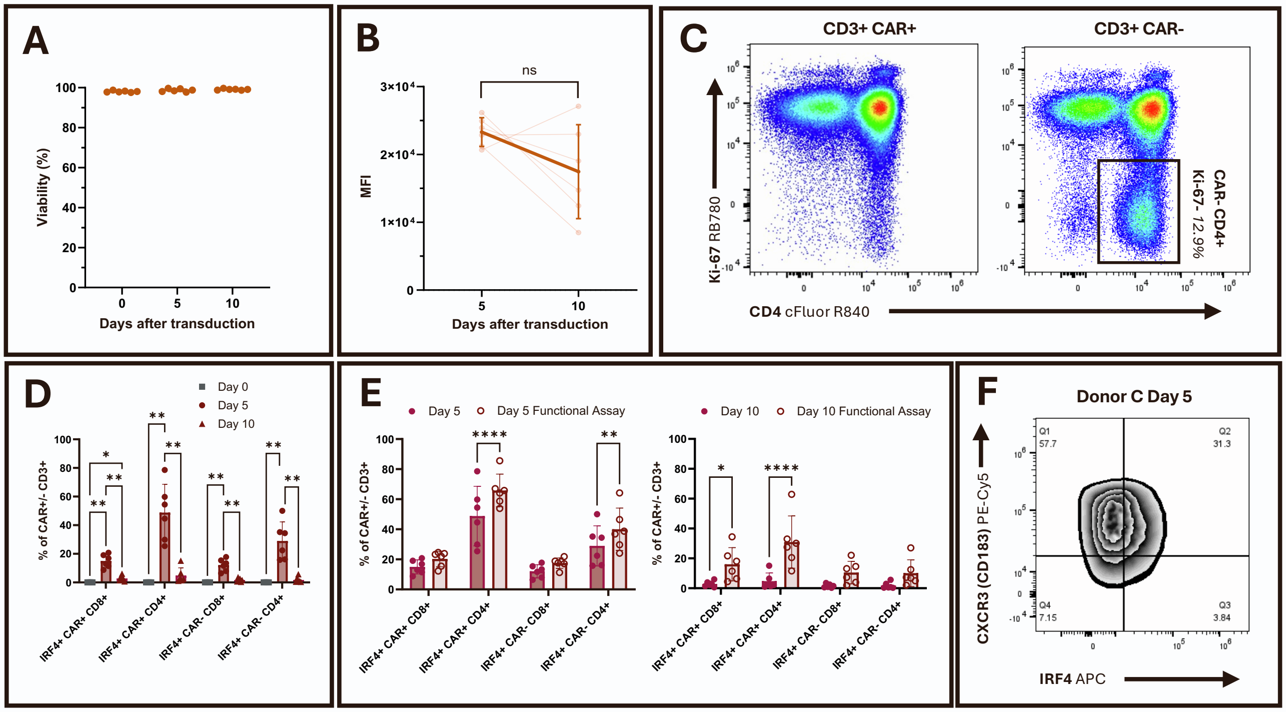

Figure S1. CAR T cell viability and subset-specific proliferation.

(**A**) Viability of CD3⁺ T cells on days 0, 5, and 10 of culture (*n* = 6 donors, two independent experiments). (**B**) MFI of CAR in total CD3⁺ T cells at days 5 and 10 (*n* = 6 donors, two independent experiments). (**C**) Representative flow cytometry plots showing Ki-67 and CD4 expression in CD3⁺CAR⁺ and CD3⁺CAR⁻ cells at day 5 (*n* = 1 donor). (**D**) Frequencies of IRF4⁺ T cell subsets within CAR⁺CD4⁺, CAR⁺CD8⁺, CAR⁻CD4⁺, and CAR⁻CD8⁺ populations on days 0, 5, and 10 (*n* = 6 donors, two independent experiments). (**E**) Frequencies of IRF4⁺ subsets following 3 h co-culture at days 5 and 10 in CAR⁺ and CAR⁻, CD4⁺ and CD8⁺ compartments (*n* = 6 donors, two independent experiments). (**F**) Contour plot showing IRF4 and CXCR3 co-expression in CD3⁺CAR⁺ T cells from a representative day 5 donor (*n* = 1 donor). Statistical analyses were performed using a paired, two-tailed Student's t-test (B), two-way repeated measures ANOVA with Tukey's (D) or Šídák's (E) multiple comparisons test. Data represent mean ± s.d.; *$p < 0.05$; **$p < 0.01$; ****$p < 0.0001$; n.s., not significant. Absence of *p*-values indicates non-significance at the $p = 0.05$ threshold.

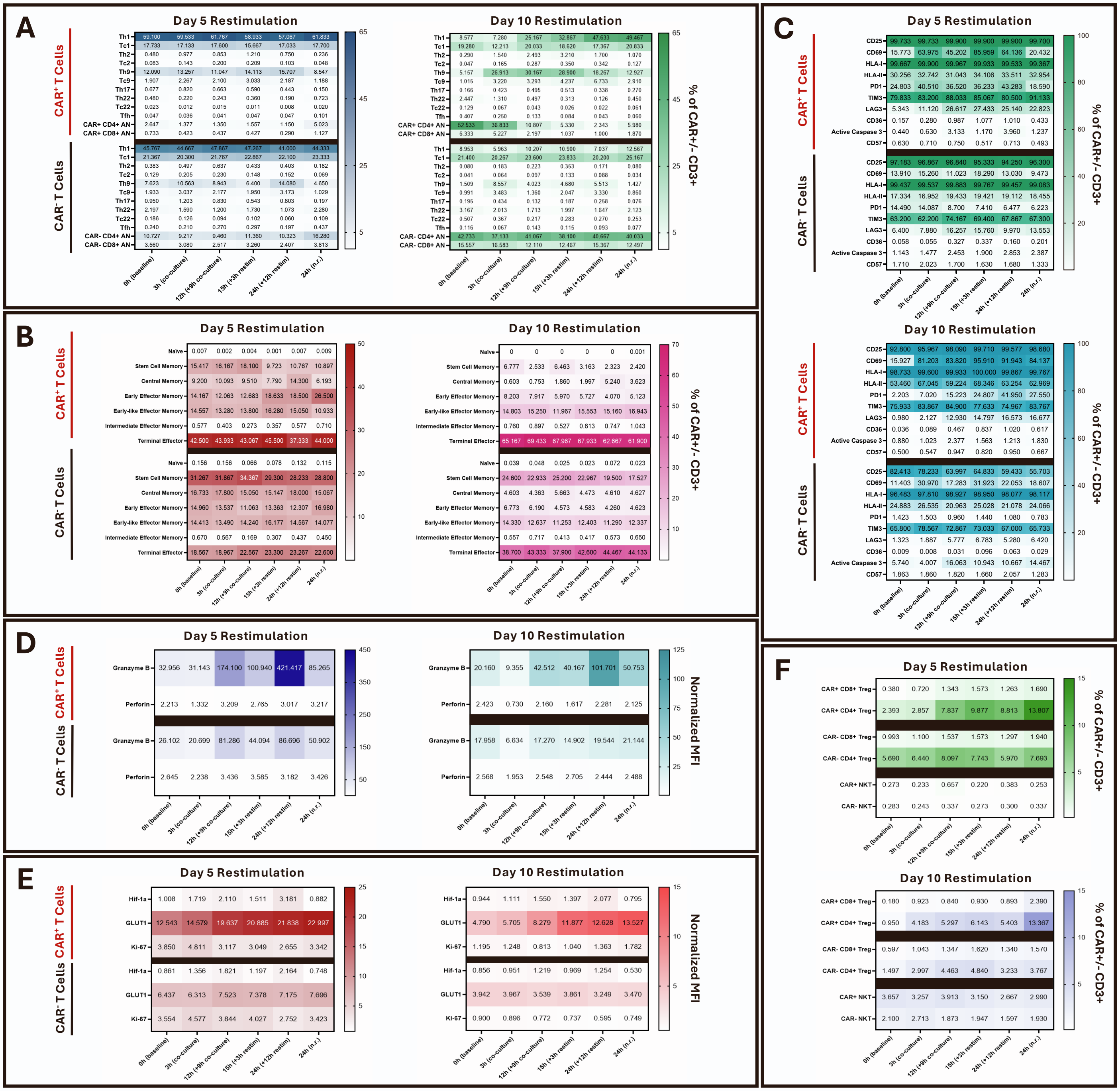

Figure S2. Heatmap of CAR$^+$ and CAR$^-$ T cell profiling during 24 h restimulation.

(**A**) Frequencies of CD4$^+$ helper (Th) and CD8$^+$ cytotoxic (Tc) subsets within CAR$^+$ and CAR$^-$ compartments across the 24 h restimulation assay, shown for day 5 (left) and day 10 (right) products (*n* = 3 donors). Only subsets representing ≥0.5% of total CD3$^+$ T cells at any time point or in any donor are shown. AN, "all negative" (CXCR3$^-$IRF4$^-$CRTH2$^-$CCR6$^-$CXCR5$^-$). (**B**) Distribution of memory and differentiation subsets (Naïve, Stem Cell Memory, Central Memory, Early Effector Memory, Early-like Effector Memory, Intermediate Effector Memory, Terminal Effector) within CAR$^+$ and CAR$^-$ T cells (*n* = 3 donors). (**C**) Frequencies of activation, checkpoint, apoptotic, and senescence markers (CD25, CD69, HLA-I and -II, PD1, TIM3, LAG3, CD36, active caspase 3, CD57) within CAR$^+$ and CAR$^-$ T cells (*n* = 3 donors). (**D**) Normalized MFI of granzyme B and perforin in CAR$^+$ and CAR$^-$ T cells (*n* = 3 donors). (**E**) Normalized MFI of Hif-1a, GLUT1, and Ki-67 in CAR$^+$ and CAR$^-$ T cells (*n* = 3 donors). (**F**) Frequencies of Tregs in CAR$^+$CD4$^+$, CAR$^+$CD8$^+$, CAR$^-$CD4$^+$, and CAR$^-$CD8$^+$ compartments, and NK-like T cell subsets in CAR$^+$ and CAR$^-$ T cells (*n* = 3 donors). Statistical analyses were performed using two-way repeated measures ANOVA with Tukey's multiple comparisons test (Tables S6 and S7). Data represent mean values from three healthy donors. n.r., non-restimulated; restim, restimulated.

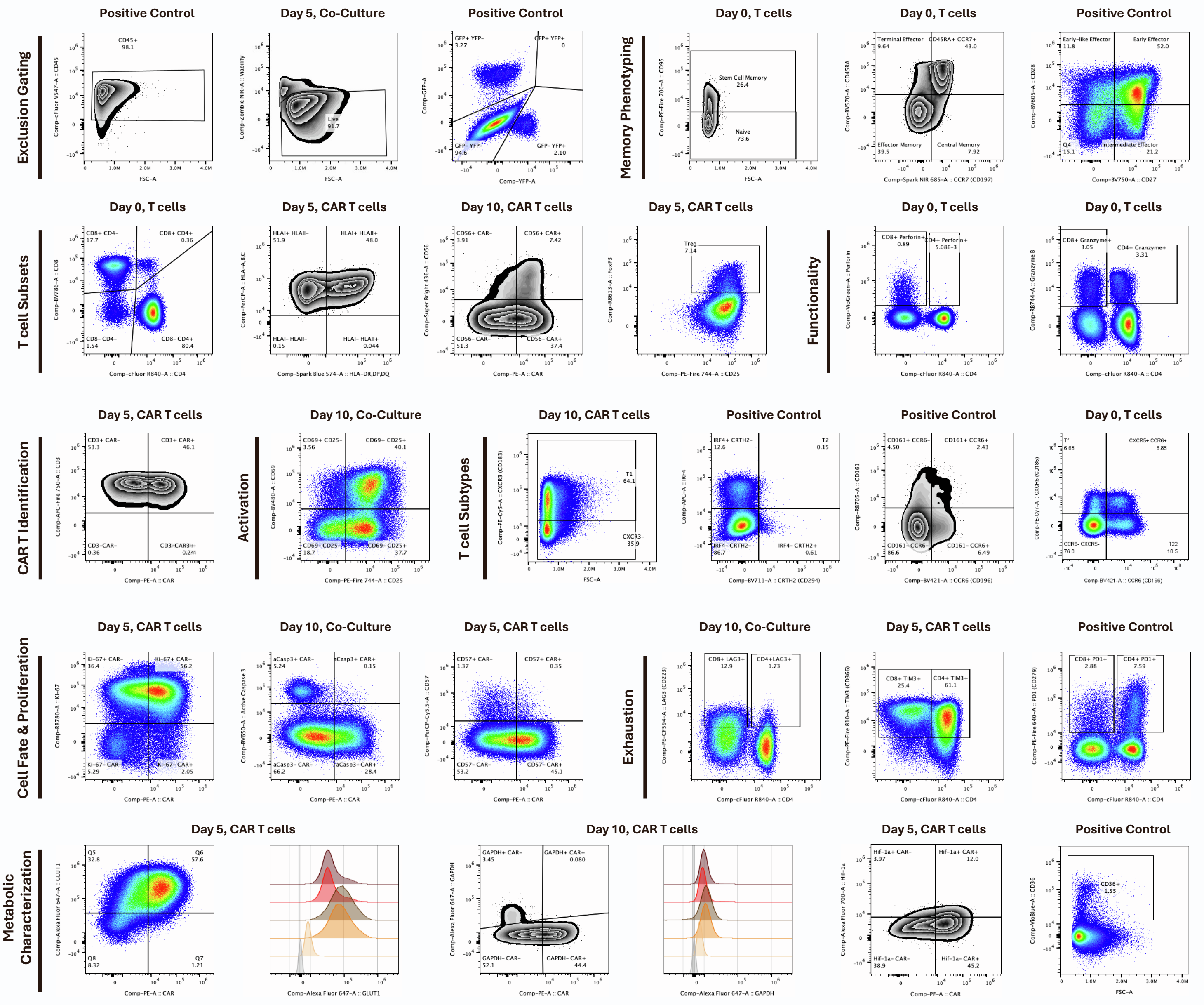

Figure S3. Representative positive controls for all major CAR T cell marker categories.

(**Top row**) CD45 lineage, viability, and YFP⁻/GFP⁻ exclusion gating were applied to isolate viable CD3⁺ T cells. Memory subsets were defined using CD45RA, CCR7, CD27, CD28, and CD95. (**Second row**) CD4⁺ and CD8⁺ subsets were classified using CD3, CD4 and CD8. HLA-A, B, C and HLA-DP, DQ, DR were used to assess HLA knockout efficiency in allogeneic products. CD3⁺CD56⁺ cells were gated as NK-like T cells, and Tregs were identified as CD25⁺FoxP3⁺, example shown as CD4⁺CD25⁺FoxP3⁺. Functional markers included intracellular granzyme B and perforin. (**Third row**) CAR⁺ and CAR⁻ populations were defined using anti-CAR staining and CD3 expression. Activation markers (CD25, CD69) were assessed in antigen-stimulated cells. Th and Tc subsets were phenotyped based on CXCR3, CRTH2, IRF4, CD161, CCR6, and CXCR5. (**Fourth row**) Proliferation was assessed by intracellular Ki-67 staining, apoptosis by active caspase 3, and senescence by extracellular CD57 staining. Exhaustion profiling included LAG3, TIM3, and PD1. (**Bottom row**) Metabolic profiling included GLUT1, GAPDH, Hif-1a, and CD36. Histograms illustrate fluorescence resolution for GLUT1 and GAPDH across unstained, day 0, day 5, day 5 co-culture, day 10, and day 10 co-culture conditions. Fluorescence intensities are shown as normalized counts. All gates were defined using FMO, biological negative controls, or validated positive controls. Plot titles indicate the sample source.

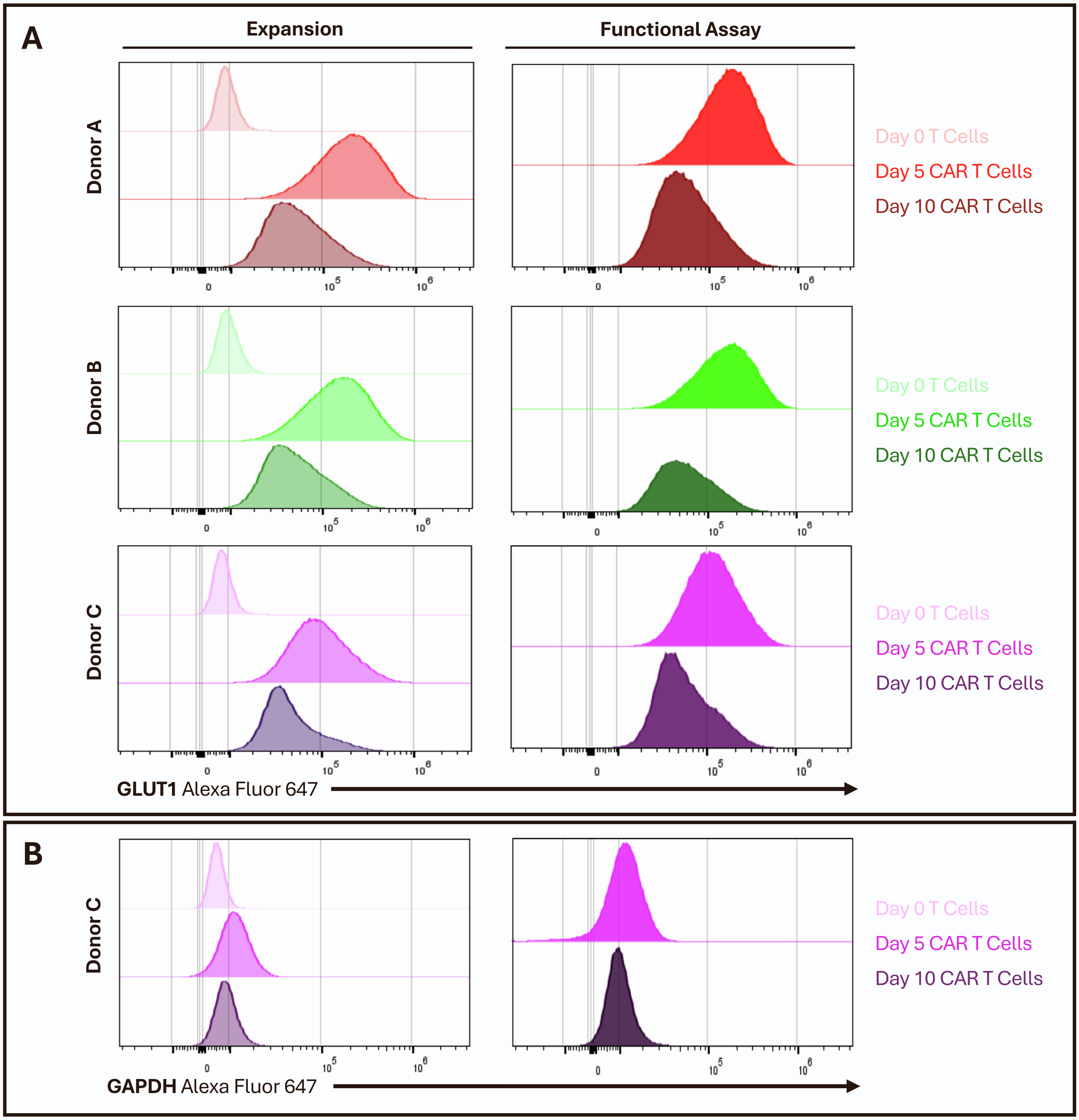

Figure S4. Switchable GLUT1 and GAPDH staining enables modular metabolic profiling of CAR T cells.

(**A**) Histogram overlays showing GLUT1 expression (Alexa Fluor 647) in three healthy donors (Donors A–C), measured at day 0, day 5, and day 10 (left), and after 3 h co-culture (right). (**B**) Histogram overlays for GAPDH expression (Alexa Fluor 647) from Donor C under the same conditions. Fluorescence intensities are shown as normalized counts. A darker color scheme (right) distinguishes co-culture (functional assay) histograms from expansion histograms.

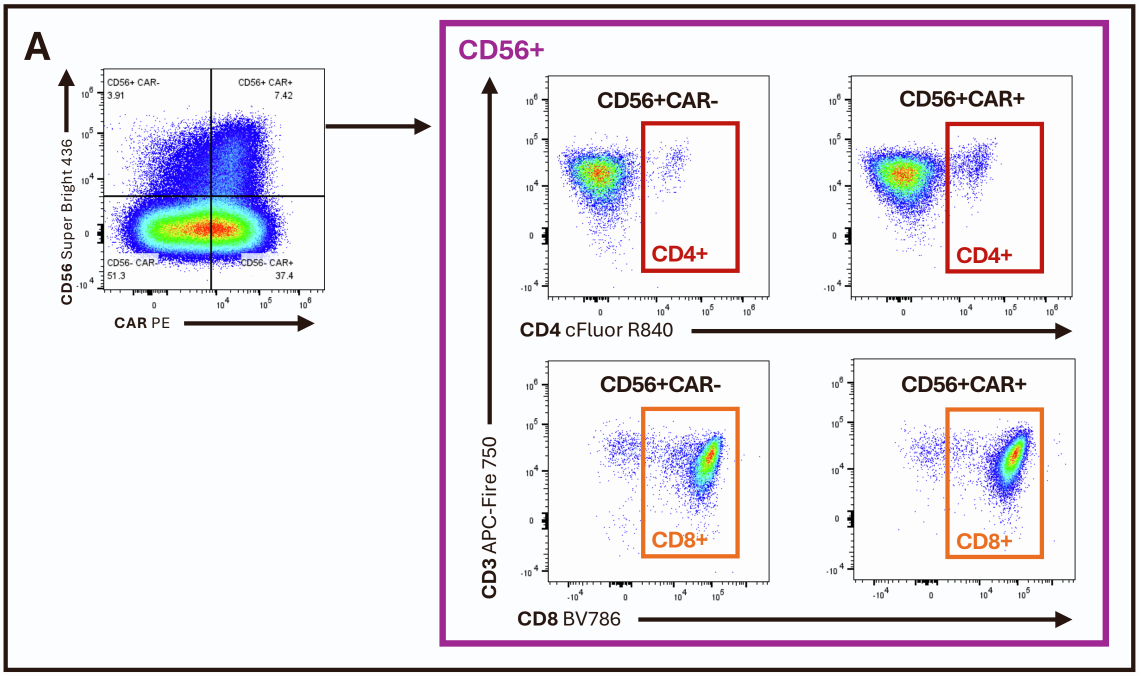

Figure S5. CD56⁺ CAR⁺ T cell enrichment within CD8⁺ compartment.

(**A**) Representative gating of $CD56^{+}CAR^{+}$ and CAR^{-} T cells from a day 10 sample. Plots show $CD56^{+}$ subsets based on CAR, CD4, and CD8 expression. $CD56^{+}CAR^{+}$ cells were predominantly localized within the $CD8^{+}$ compartment, indicating that CD56 expression in CAR^{+} T cells was largely restricted to $CD8^{+}CD3^{+}$ cells. Data are representative of one donor (*n* = 1).

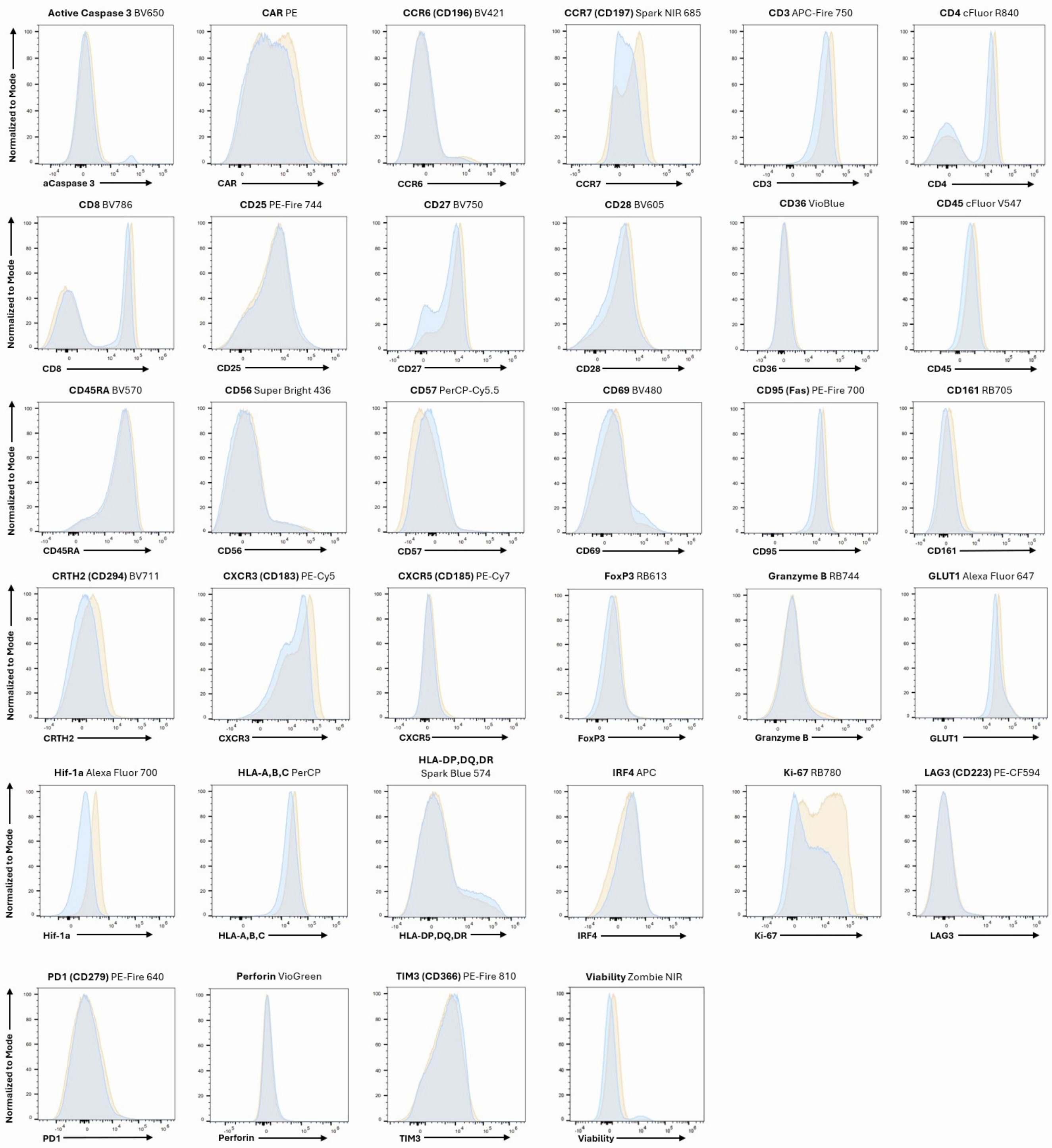

Figure S6. Histogram overlays of fresh versus cryopreserved cells.

Overlay plots showing normalized expression profiles of 34 spectral cytometry markers in day 10 cells under fresh (**orange**) and cryopreserved (**blue**) conditions. Fluorescence intensities are shown as normalized counts. Viability Zombie NIR was gated on single cells, and CD45 was gated on $GFP^{-}YFP^{-}$. All other markers were gated on $CD45^{+}$.

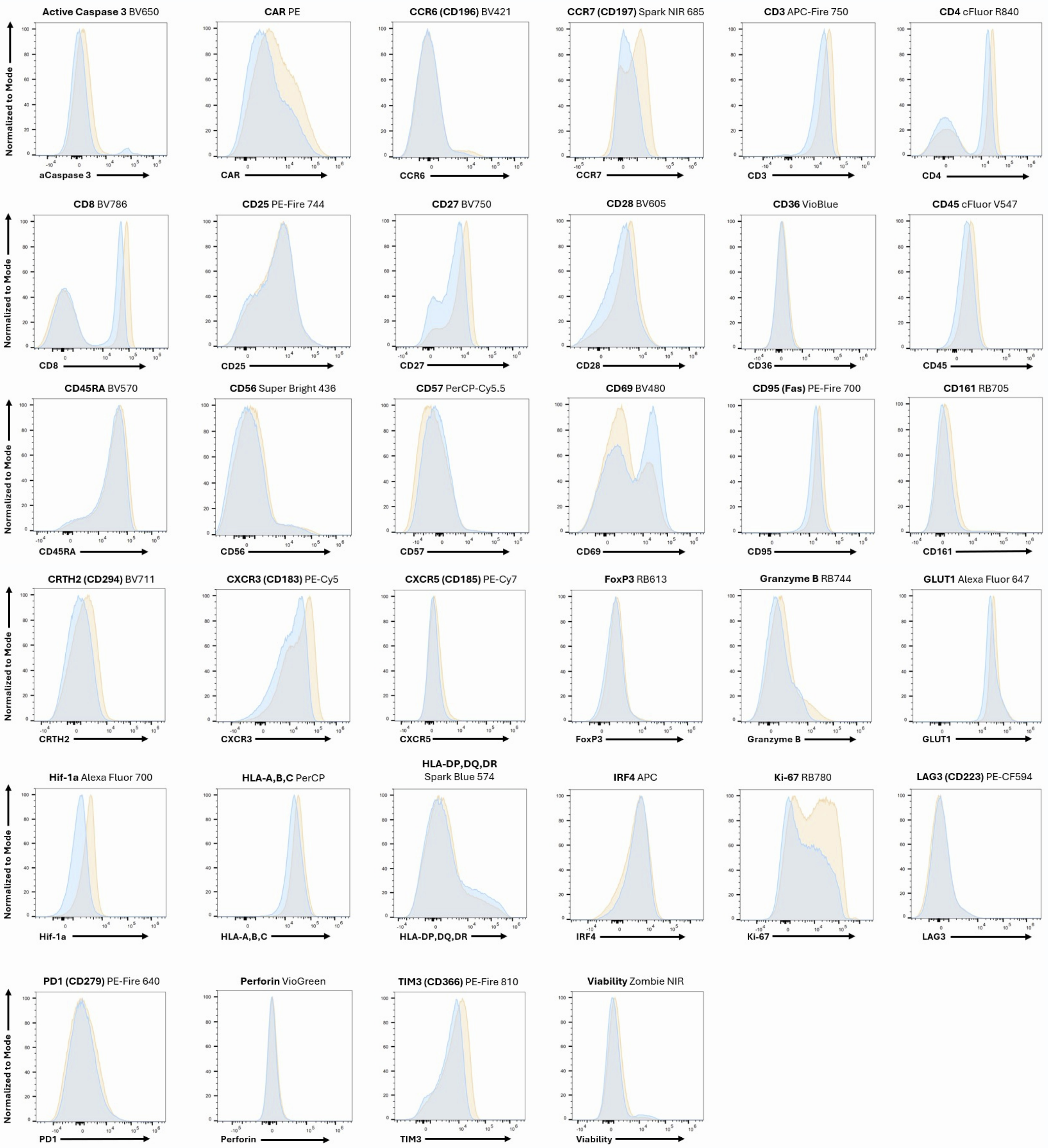

Figure S7. Marker expression in fresh versus cryopreserved cells following 3 h co-culture.

Histogram overlays comparing 34 spectral cytometry markers in day 10 cells under fresh (**orange**) and cryopreserved (**blue**) conditions after 3 h co-culture with $CD19^{+}$ Nalm6-YFP^{+} target and $CD19^{-}$ Nalm6-GFP^{+} control cells. Fluorescence intensities are shown as normalized counts. GFP and YFP were excluded from analysis, as they were introduced post-thaw. Viability Zombie NIR was gated on single cells, and CD45 was gated on GFP^{-} YFP^{-}. All other markers were gated on $CD45^{+}$.

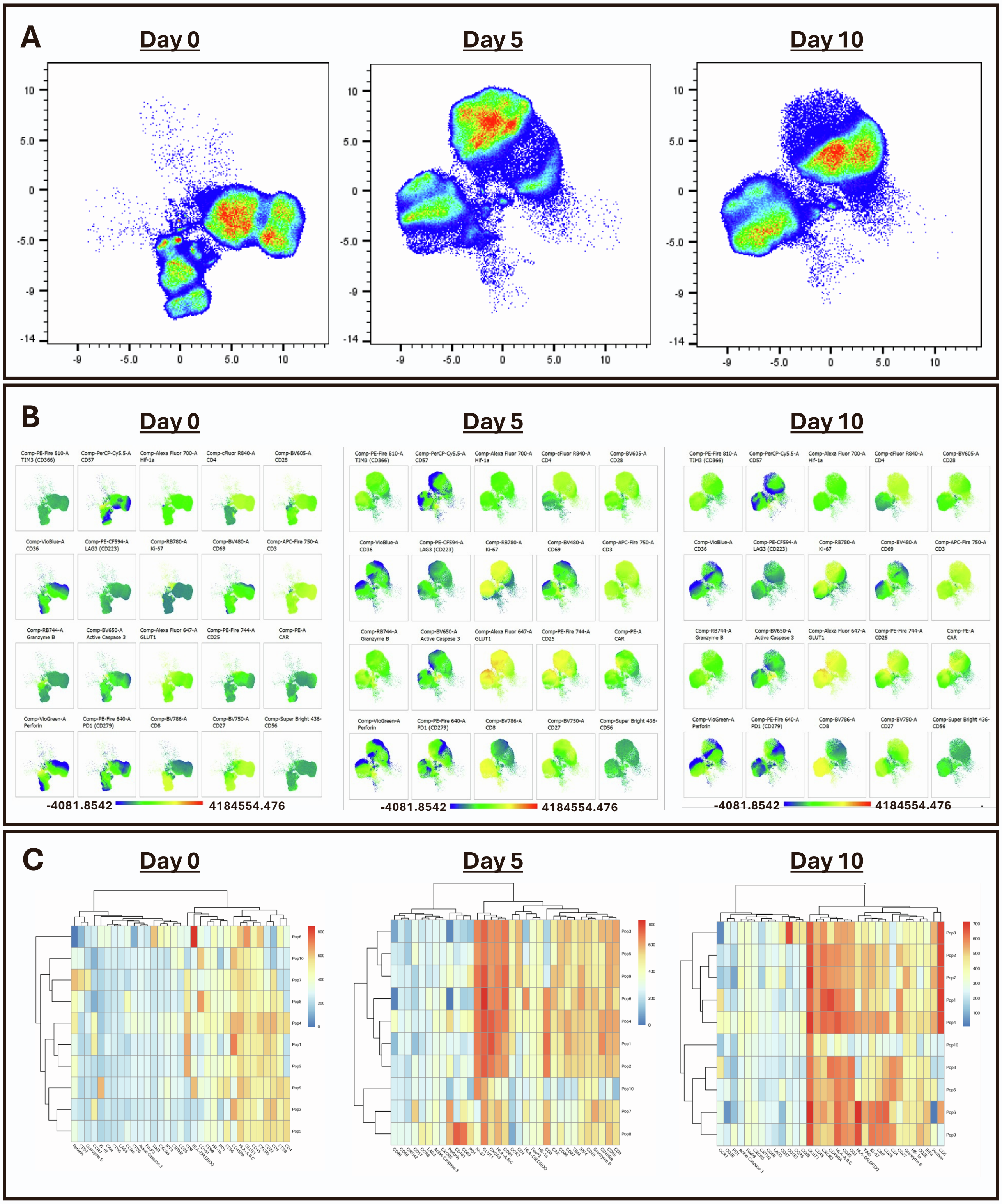

Figure S8. UMAP clustering across manufacturing timepoints.

(**A**) UMAP clustering of CD45^{+} gated cells at day 0, day 5, and day 10, illustrating population shifts during expansion (*n* = 6 donors, two independent experiments). (**B**) UMAP marker overlay plots showing relative expression intensities of selected surface and intracellular markers across all timepoints (*n* = 6 donors, two independent experiments). (**C**) Hierarchically clustered heatmaps displaying MFI values for each marker, highlighting temporal changes in expression. Data represent concatenated and downsampled events (*n* = 6 donors, two independent experiments).

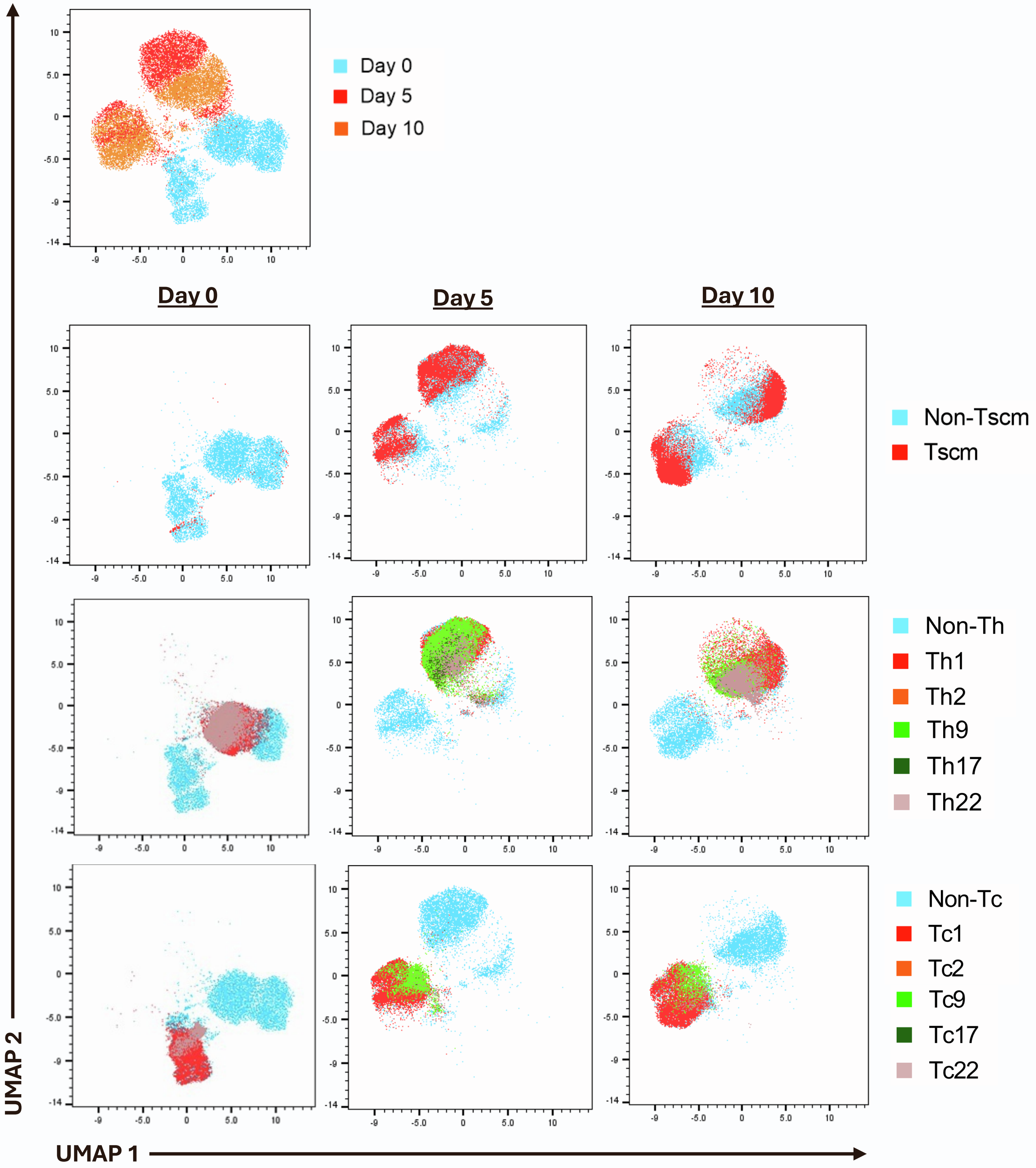

Figure S9. UMAP-based projection of T cell subset localization across manufacturing timepoints.

UMAP overlays showing the distribution of major T cell subsets within the clustered CD45^{+} population at day 0, day 5, and day 10 (*n* = 6 donors, two independent experiments). **Top row**: Combined UMAP illustrating global shifts in phenotypes during expansion. **Subsequent rows**: Subset-specific overlays highlighting stem cell memory (T_{SCM}), CD4^{+} helper subsets (Th1, Th2, Th9, Th17, Th22), and CD8^{+} cytotoxic subsets (Tc1, Tc2, Tc9, Tc17, Tc22), with distinct localization and temporal remodeling. Each dot represents a single cell, colored by subset identity; blue populations represent cells that do not fall under an annotated subset identity.

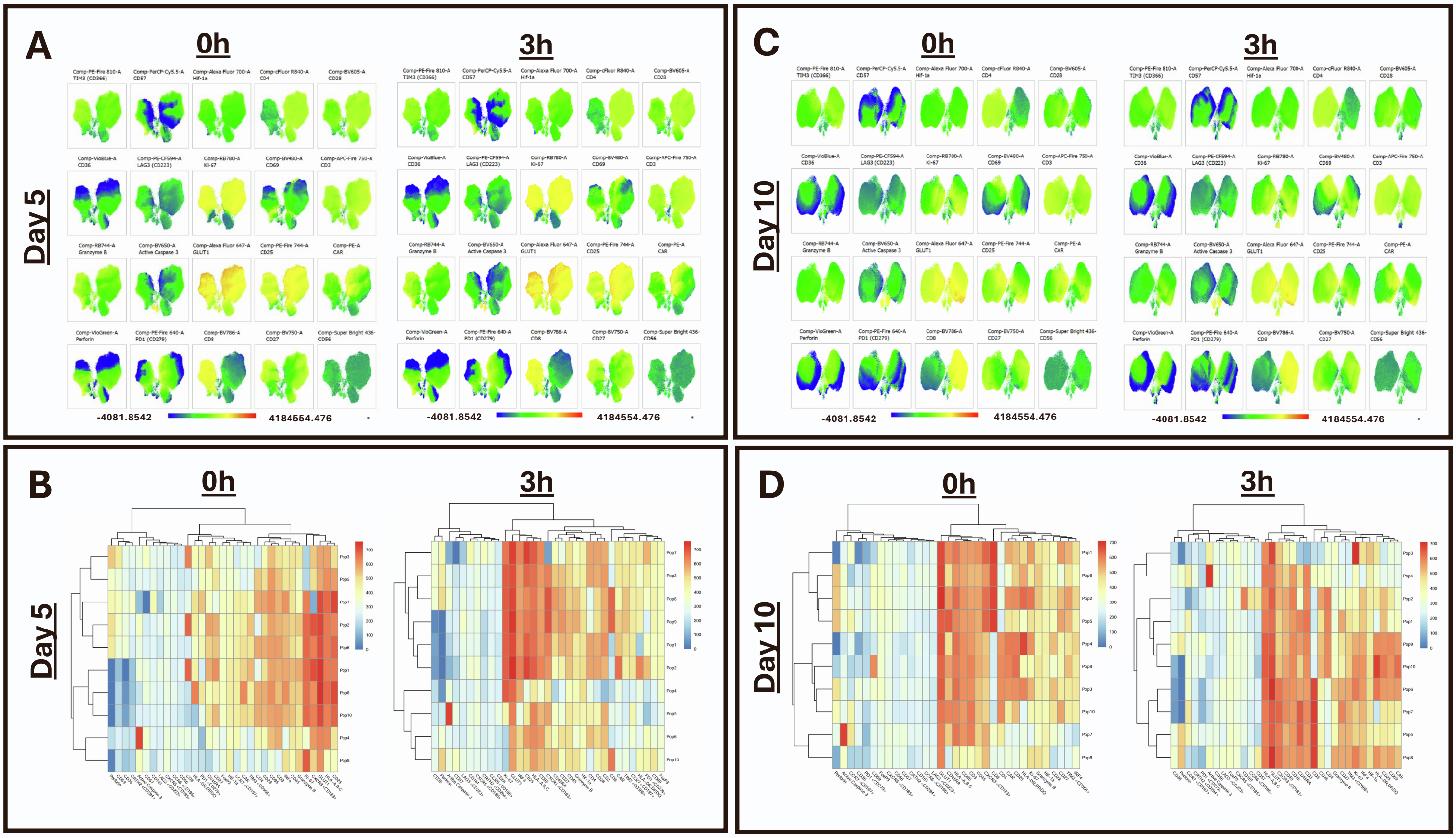

Figure S10. UMAP clustering of day 5 and day 10 cells at baseline and after 3 h co-culture.

(**A, C**) UMAP overlays of clustered CD45^{+} gated cells at day 5 (A) and day 10 (C), comparing baseline (0 h) and 3 h co-culture with CD19^{+} Nalm6-YFP^{+} target and CD19^{-} Nalm6-GFP^{+} control cells. Expression patterns are shown for selected lineage, activation, checkpoint, metabolic, and functional markers (*n* = 6 donors, two independent experiments). (**B, D**) Heatmaps displaying normalized MFI of markers at day 5 (B) and day 10 (D), comparing 0 h (left) and 3 h (right) conditions (*n* = 6 donors, two independent experiments).

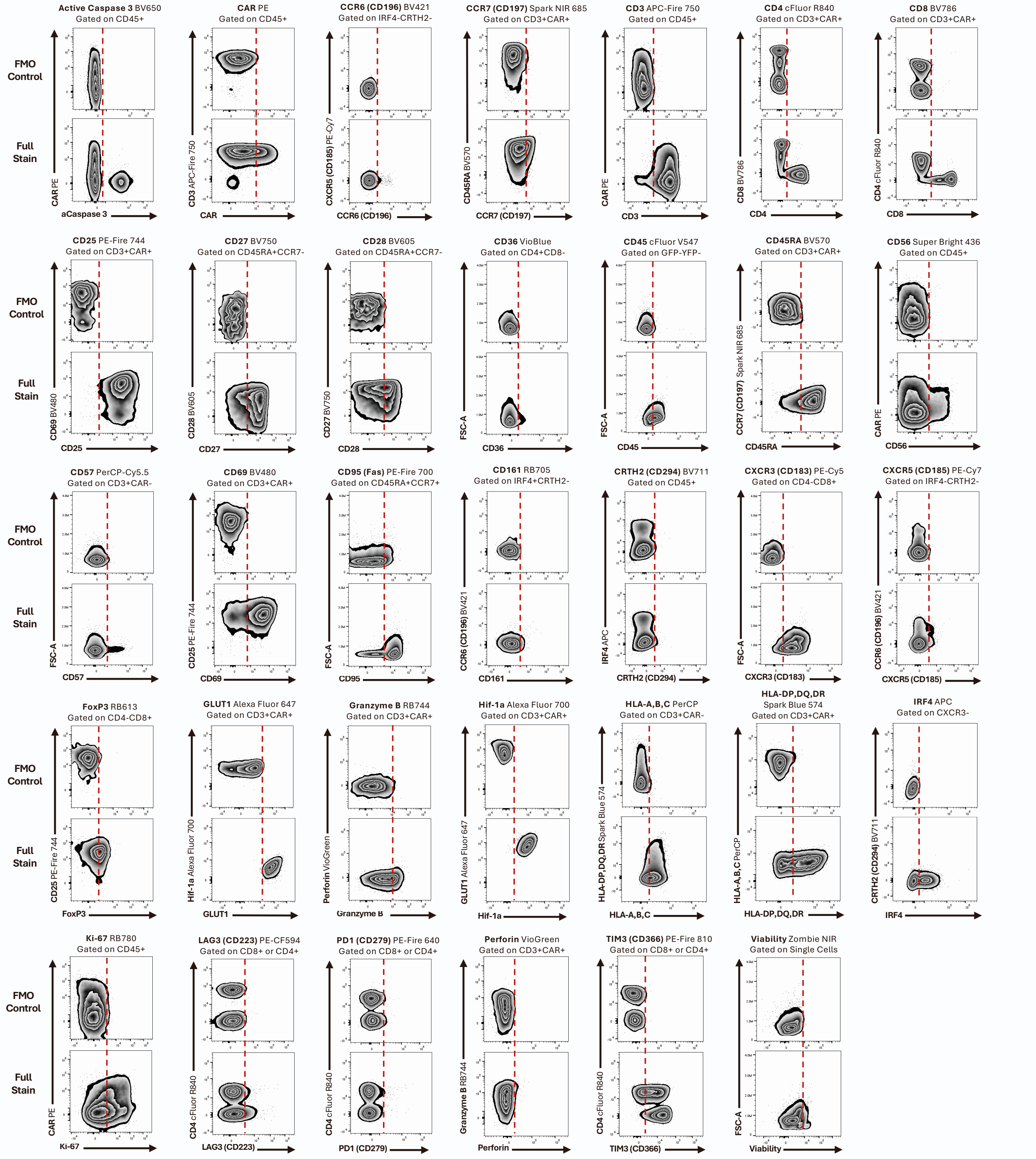

Figure S11. Fluorescence-minus-one (FMO) controls for spectral cytometry panel validation.

Representative contour plots comparing FMO controls (**top rows**) with fully stained samples (**bottom rows**) for each of the 34 antibody markers used in the spectral panel. Gating thresholds (red dashed lines) were defined using FMO controls and applied to fully stained samples for consistent marker quantification. Gates were drawn within biologically relevant parent populations, as indicated on each plot (e.g. Gated on $CD3^{+}CAR^{+}$, $CD45^{+}$). Data shown are from CAR^{+} T cells following 3 h co-culture or from composite samples generated by pooling PBMCs, gene-edited CAR T cells, non-edited T cells, and activated T cells.

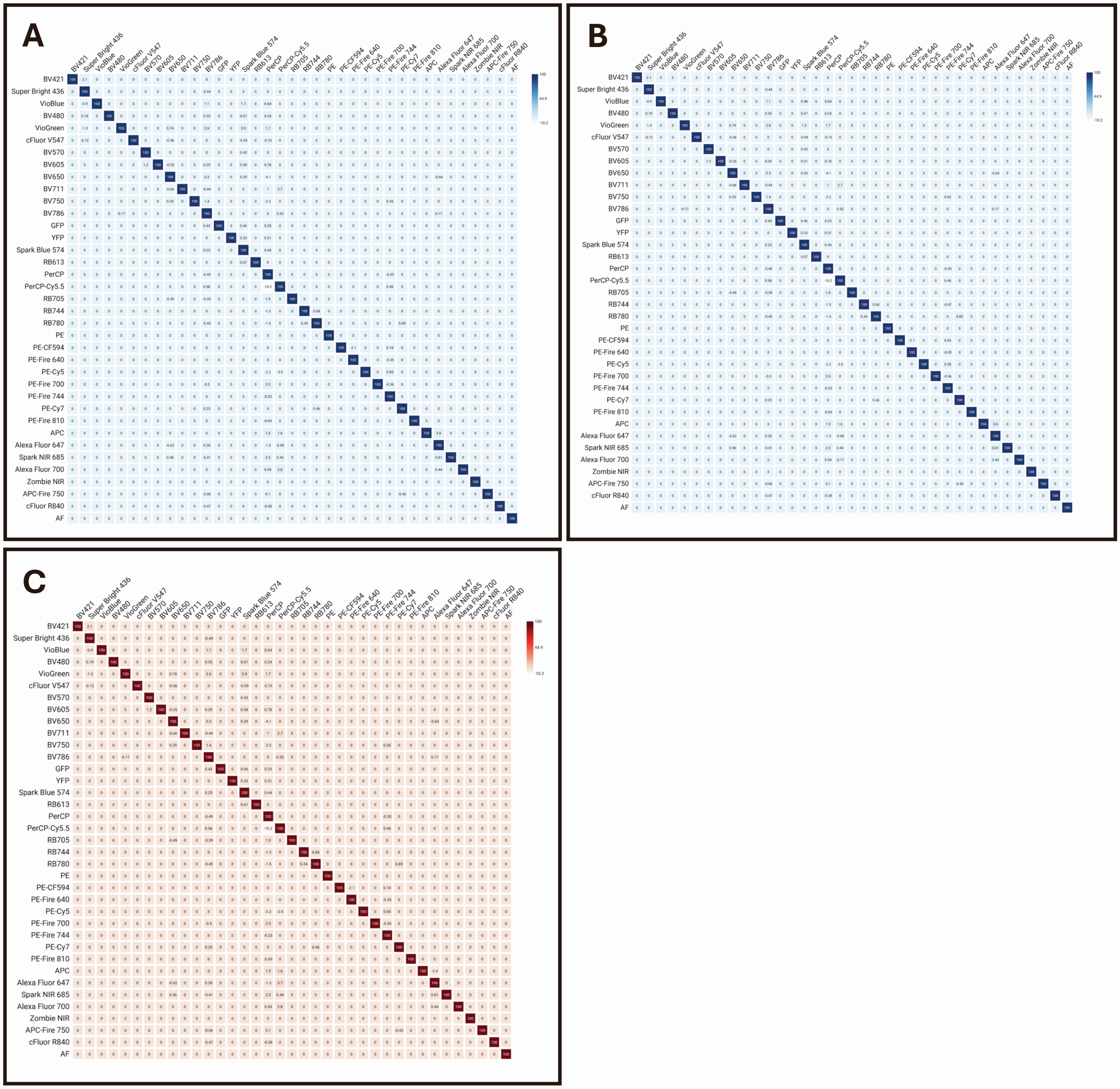

Figure S12. Compensation matrices and spectral spillover assessment.

(**A–C**) Compensation matrices for the 36-color panel applied to samples from independent experiments: (**A**) GLUT1 Experiment 1, (**B**) GLUT1 Experiment 2, and (**C**) GAPDH. Each matrix displays spillover values from individual fluorochromes (“from” columns) into other detectors (“to” rows), where higher values indicate greater unmixing error. For example, in panel A, manual compensation was applied from Alexa Fluor 647 into APC (spillover value: 3.6). Matrices were generated using manual compensation of residual unmixing errors in SpectroFlo.

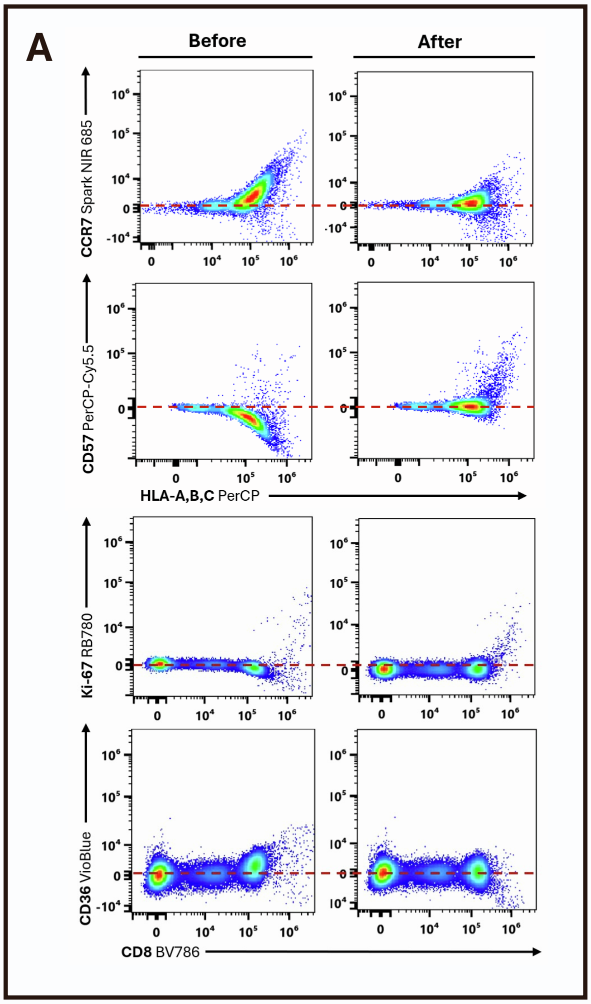

B

Fluorochromes		Correction of Unmixing Errors
From	To	
PerCP	Spark NIR 685	2.5
PerCP	PerCP-Cy5.5	-10.2
BV786	RB780	-0.45
BV786	VioBlue	1.1

Figure S13. Correction of spectral unmixing errors in 36-color panel analysis.

(**A**) Dot plots showing representative examples of signal artifacts caused by unmixing errors before (left) and after (right) manual correction in SpectroFlo. (**B**) Summary table of unmixing corrections applied between specific fluorochrome pairs, with correction values indicating the magnitude of signal adjusted (“From” donor fluorochrome into “To” receiver fluorochrome).

A

Violet (405nm, 100mW)			
**Detector Channel**	**Center Wavelength (nm)**	**Bandwidth (nm)**	**Fluorochrome**
V1	428	15	*BV421*
V2	443	15	*Super Bright 436*
V3	458	15	*VioBlue*
V4	473	15	
V5	508	20	*BV480*
V6	525	17	
V7	542	17	*VioGreen*
V8	581	19	*BV570 / cFluor V547*
V9	598	20	
V10	615	20	*BV605*
V11	664	27	*BV650*
V12	692	28	
V13	720	29	*BV711*
V14	750	30	*BV750*
V15	780	30	*BV786*
V16	812	34	

Blue (488nm, 50mW)			
**Detector Channel**	**Center Wavelength (nm)**	**Bandwidth (nm)**	**Fluorochrome**
B1	508	20	*GFP*
B2	525	17	
B3	542	17	*YFP*
B4	581	19	*Spark Blue 574*
B5	598	20	
B6	615	20	*RB613*
B7	661	17	
B8	679	18	*PerCP*
B9	697	19	*PerCP-Cy5.5*
B10	717	20	*RB705*
B11	738	21	
B12	760	23	*RB744*
B13	783	23	
B14	812	34	*RB780*

Yellow-Green (561nm, 50mW)			
**Detector Channel**	**Center Wavelength (nm)**	**Bandwidth (nm)**	**Fluorochrome**
YG1	577	20	*PE*
YG2	598	20	
YG3	615	20	*PE-CF594*
YG4	661	17	*PE-Fire 640*
YG5	679	18	*PE-Cy5*
YG6	697	19	
YG7	720	29	*PE-Fire 700*
YG8	750	30	*PE-Fire 744*
YG9	780	30	*PE-Cy7*
YG10	812	34	*PE-Fire 810*

Red (640nm, 80mW)			
**Detector Channel**	**Center Wavelength (nm)**	**Bandwidth (nm)**	**Fluorochrome**
R1	661	17	*APC*
R2	679	18	*Alexa Fluor 647*
R3	697	19	*Spark NIR 685*
R4	717	20	*Alexa Fluor 700*
R5	738	21	
R6	760	23	*Zombie NIR*
R7	783	23	*APC-Fire 750*
R8	812	34	*cFluor R840*

B

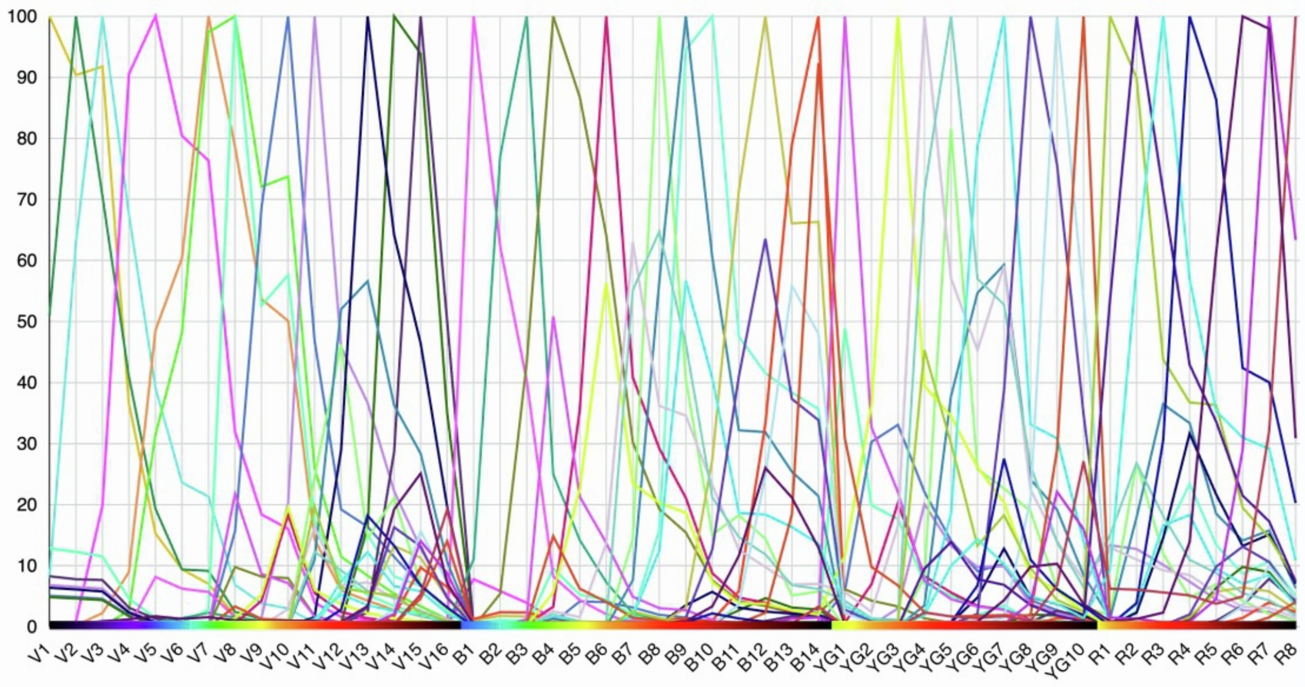

Figure S14. Instrument configuration for 4-Laser Cytek Aurora.

(**A**) Detector channels and bandpass filter specifications for the violet, blue, yellow-green, and red lasers on the Cytek Aurora. Fluorochromes used in the 36-color panel are organized by primary excitation laser and peak detector assignment. BV570 and cFluor V547 are grouped together due to overlapping peak emission in the same detector channel. (**B**) Normalized emission spectra of all 36 fluorochromes, illustrating intensity across detector channels.

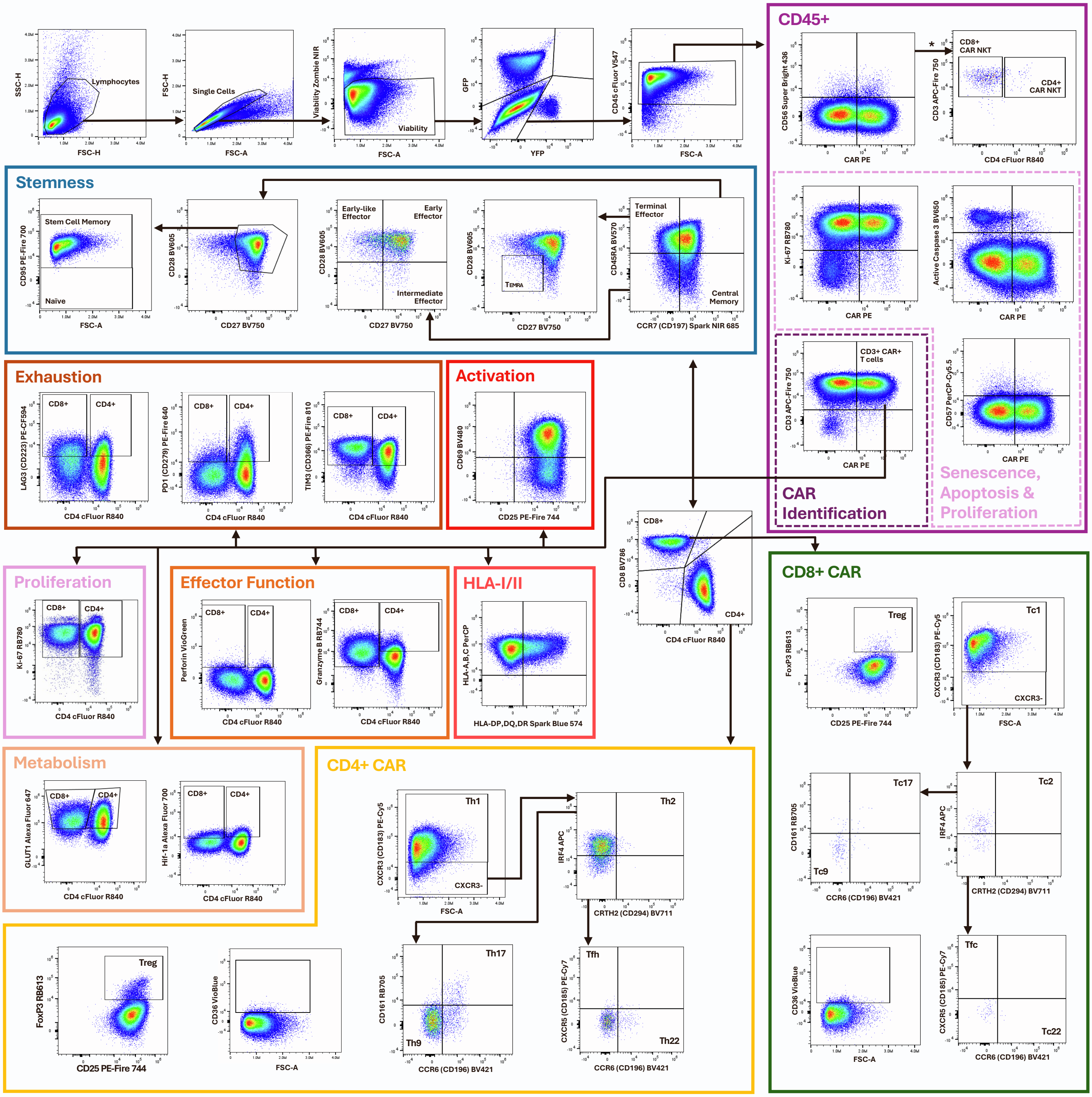

Figure S15. Manual gating strategy for multiparameter phenotypic analysis of CAR T cells.

Marker resolution using the 36-color panel under an exploratory gating strategy (*n* = 1 representative sample). Populations were first gated on FSC, SSC, viability, and CD45, followed by separation based on fluorescent proteins (GFP, YFP) and CD3/CAR expression. Subsequent gating included stemness (T_N, T_{SCM}, T_{CM}, T_{EM}, T_{TE}), exhaustion (LAG3, PD1, TIM3), activation (CD69, CD25), proliferation (Ki-67), effector function (perforin, granzyme B), HLA-I/-II expression, metabolism (GLUT1, Hif-1a), and CD4/CD8 subtypes (Th1, Tc1, Th2, Tc2, Th9, Tc9, Th17, Tc17, Th22, Tc22, Tfh, Tfc, Treg). High-level CD45 gating also included NK-like T cells, Ki-67, active caspase 3, and CD57. *CAR NK T cells and the NxCD4 panel of gates were defined using CD4 vs. CD8 (gate not shown) to exclude $CD4^-CD8^-$ and $CD4^+CD8^+$ populations, ensuring accurate representation in the subsequent CD3 vs. CD4 plot.

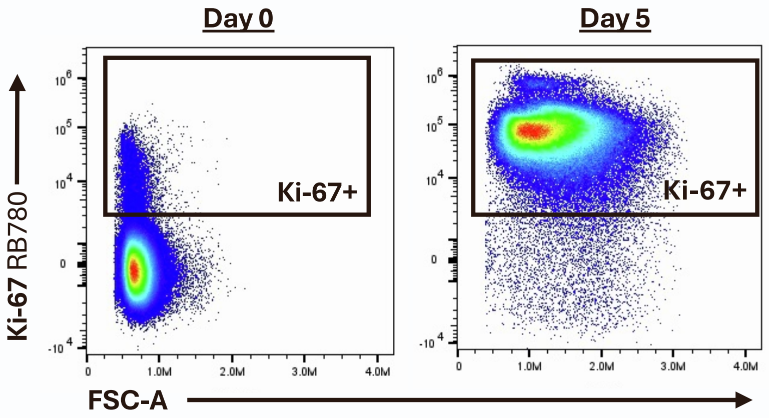

Figure S16. Representative Ki-67^{+} plots from day 0 and day 5 CAR T cells.

Representative flow cytometry plots showing Ki-67 versus forward scatter area (FSC-A) at day 0 and day 5. At day 0, most CAR T cells are Ki-67^{-}, with negative baseline MFI attributable to spectral unmixing. To facilitate data interpretation, Ki-67 MFI values were calculated from the positively gated population only. Data are from a representative donor (*n* = 1).

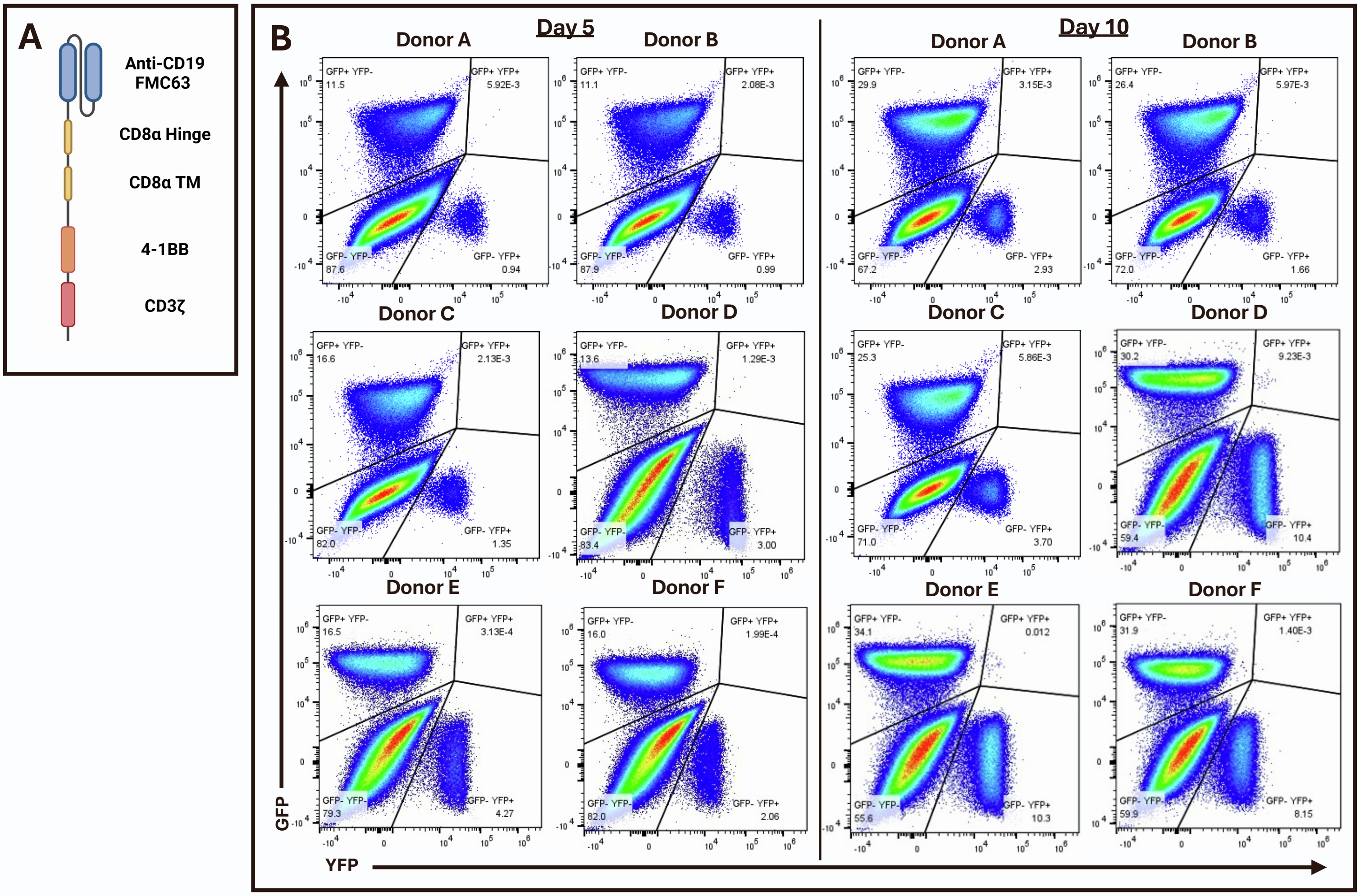

Figure S17. Representative CAR construct and cytotoxicity assay plots.

(**A**) Schematic of the anti-CD19 CAR construct used in this study, composed of the FMC63 single-chain variable fragment (scFv), a CD8α hinge and transmembrane domain, a 4-1BB costimulatory domain, and a CD3ζ signaling domain. (**B**) Representative flow cytometry plots showing cytotoxicity assay results from six healthy donors at day 5 and day 10 post-transduction. CAR T cells were co-cultured for 3 h at a 2:1:1 ratio (Effector : $CD19^+$ Nalm6-YFP^+ target : $CD19^-$ Nalm6-GFP^+ control). YFP:GFP ratios were calculated to assess antigen-specific killing. Each panel represents one donor and one timepoint.

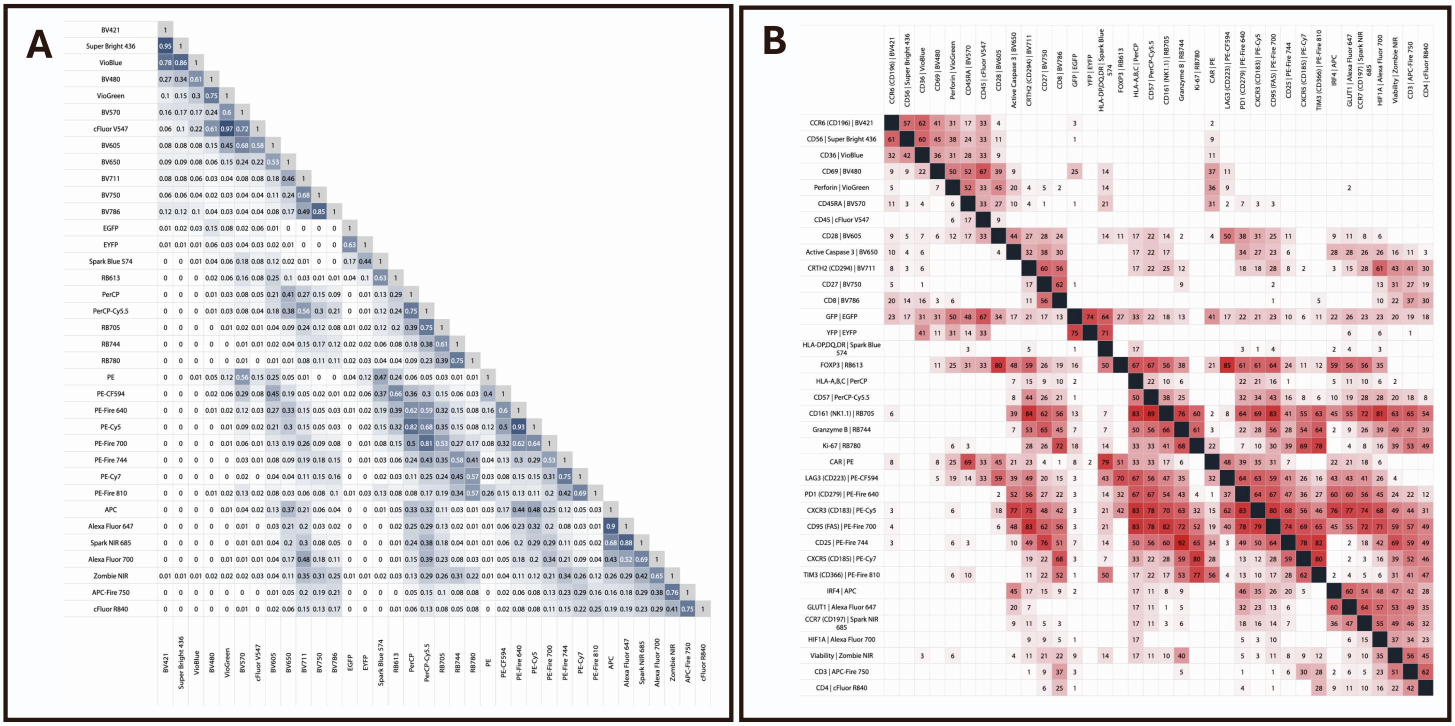

Figure S18. Spectral overlap and marker co-expression matrices.

(**A**) Pairwise similarity index illustrating spectral overlap between fluorochromes in the 36-color panel. Values indicate the degree of spectral overlap. (**B**) Expected reduction in stain index for each fluorochrome (columns) due to signal spread from other fluorochromes (rows). The theoretical stain index reduction is based on the Cytek Cloud library.

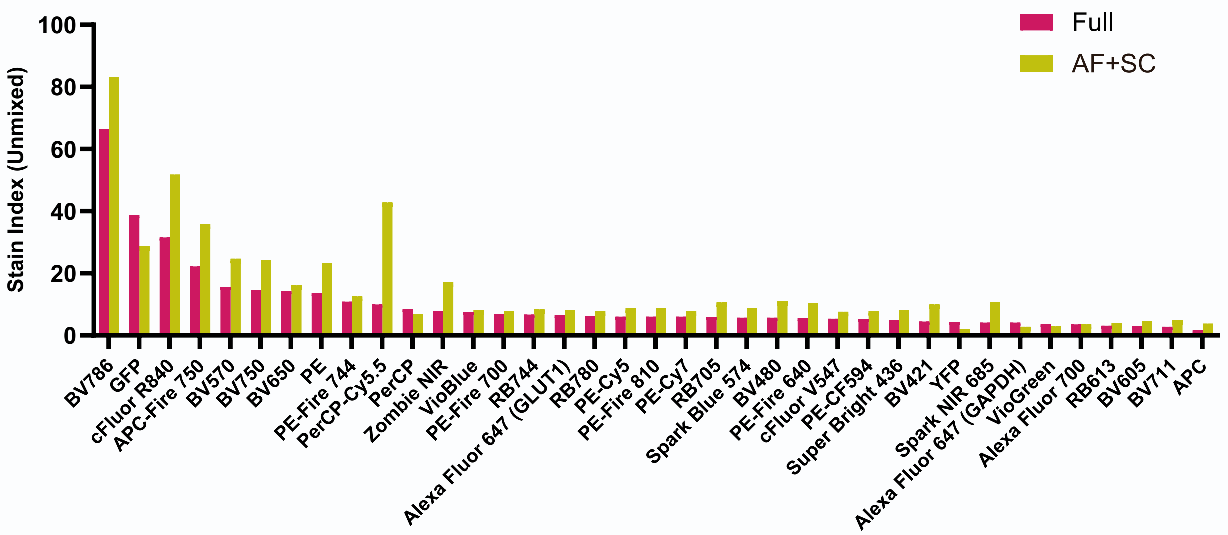

Figure S19. Stain index comparison of single color (SC) controls under full and SC unmixing matrix.

Stain index (SI) values were calculated for single-color (SC) controls when unmixed either with the full 36-marker panel and autofluorescence (“Full,” magenta) or with the individual SC tube and autofluorescence (“AF+SC,” yellow-green). Each bar represents the SI for the respective fluorochrome. Fluorochromes are ranked by SI magnitude under full unmixing conditions.

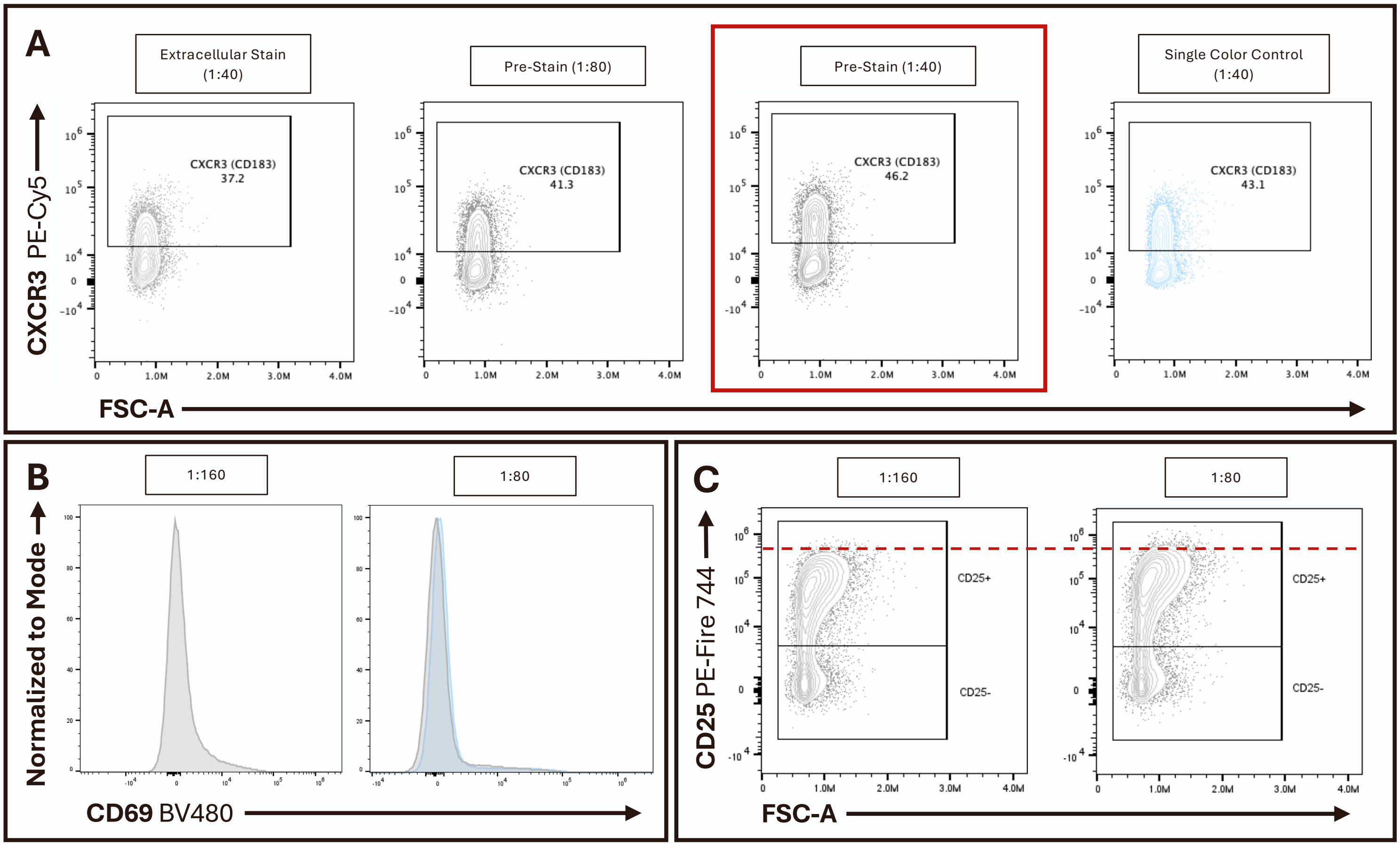

Figure S20. Titration and sequential staining optimization for CXCR3, CD69, and CD25.

(**A**) Resolution of CXCR3 (CD183) was improved by incorporating the antibody into a pre-stain step at its optimal titer (red), separated from the remaining extracellular panel. (**B**) Histogram overlays showing CD69 signal intensity at low (1:160) versus optimal (1:80) antibody titers, demonstrating enhanced resolution at the higher concentration. (**C**) Contour plots showing adjusted titers for CD25. Optimal staining was achieved at 1:160, which maintained marker resolution while reducing overall fluorescence intensity. Ratios above each plot indicate the antibody titer used. Populations in gray and blue represent fully stained and single-color samples, respectively.

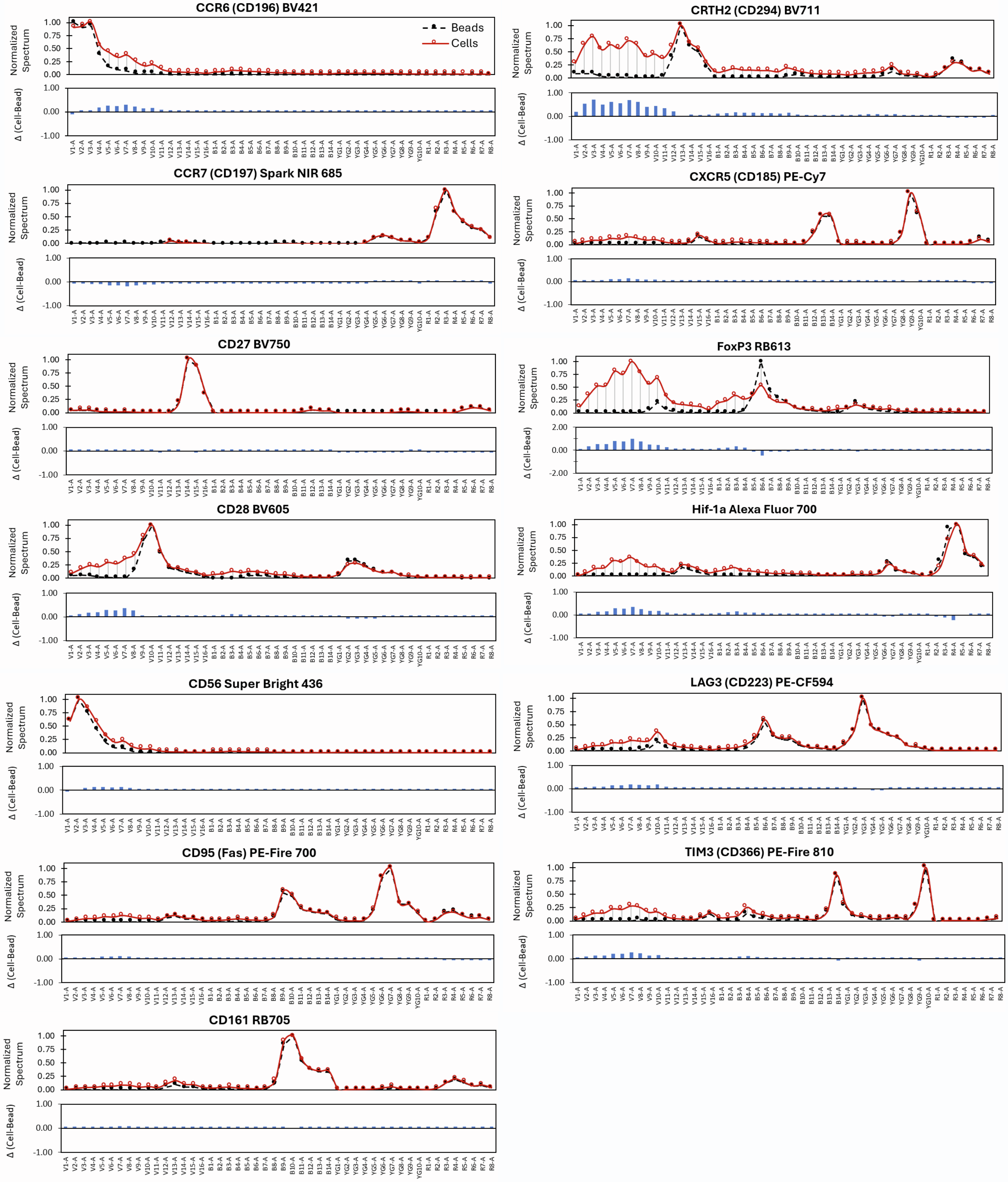

Figure S21. Spectral emission profiles and Δ values for cell- and bead-based reference controls.

Normalized emission spectra for 13 representative markers comparing bead-based (black, dashed) and cell-based (red, solid) reference controls across all 48 detectors. For each marker, the top panel displays normalized signal intensity, while the bottom panel shows the delta (Δ) values representing channel-by-channel differences in emission between cell and bead controls.

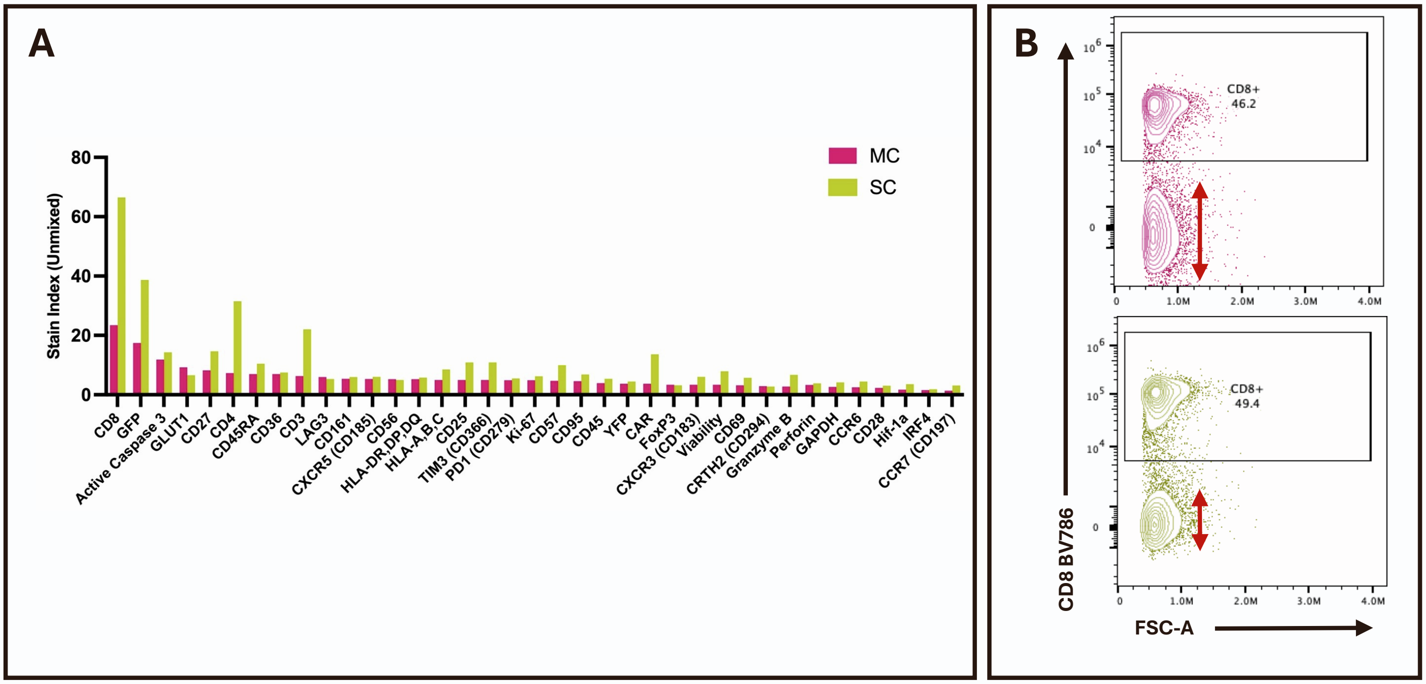

Figure S22. Comparison of stain index and resolution under single-color and multi-color staining conditions.

(**A**) Unmixed stain index (SI) values for all 36 markers under single-color (SC, yellow-green) and full multi-color (MC, magenta) staining conditions. Fluorochromes are ranked by SI magnitude under MC conditions. The greatest SI reductions were observed for CD8, GFP, CD4, and CD3 under MC staining. (**B**) Representative plots showing CD8 resolution in SC versus MC conditions from a representative sample. MC staining resulted in reduced CD8$^+$ signal intensity and increased spread of the negative population compared to SC staining.

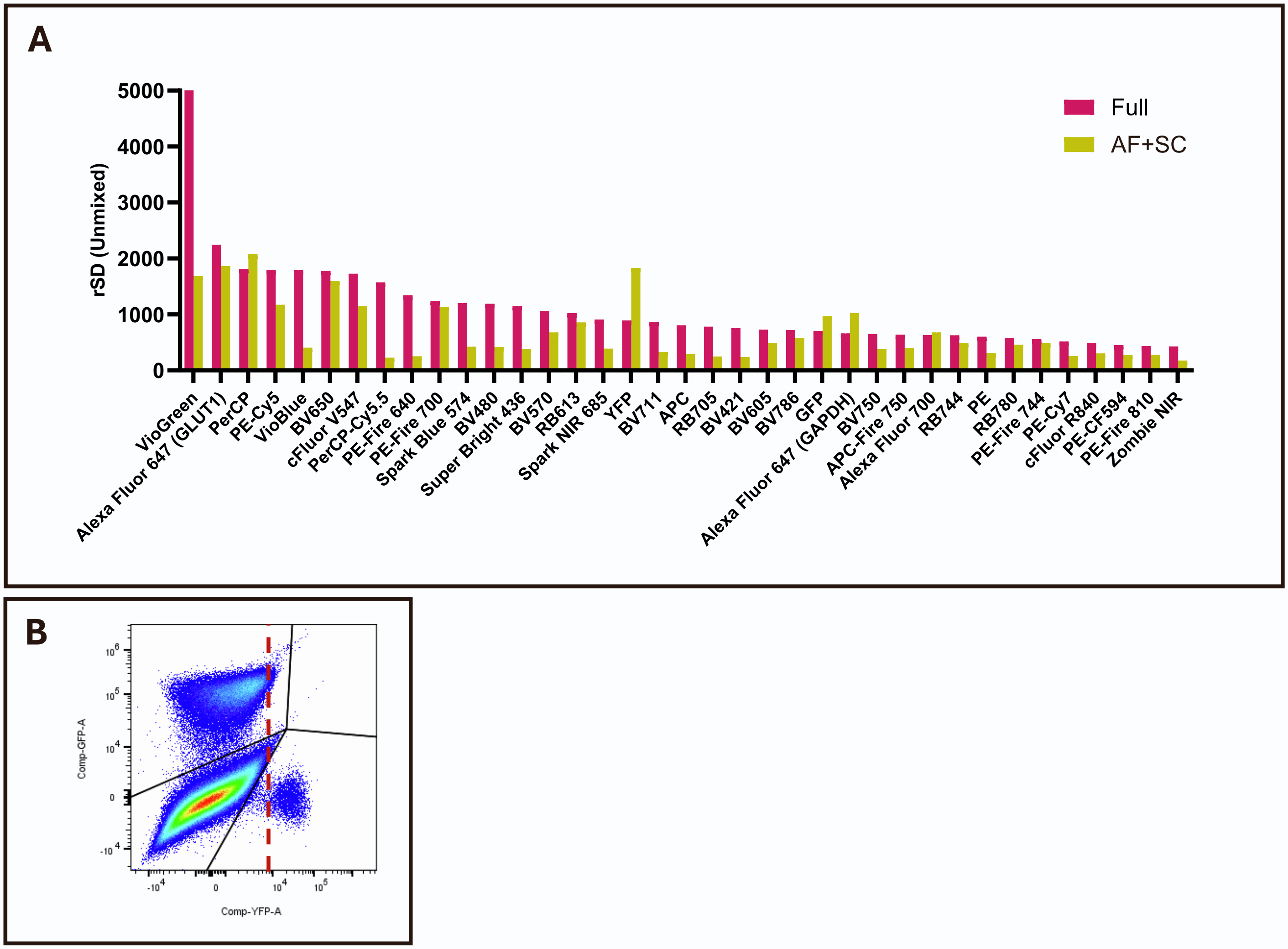

Figure S23. Spreading error of the negative population following spectral unmixing.

(**A**) Robust standard deviation (rSD) was used to quantify spreading error in the negative population after unmixing. rSD values were calculated for each single-color (SC) control when unmixed using either autofluorescence plus SC control (AF+SC, yellow-green) or the full 36-color panel with AF extraction (Full, magenta). Fluorochromes are ranked by rSD magnitude under full unmixing conditions. (**B**) Representative YFP vs. GFP plot from a day 5 CAR T cell co-culture showing unmixing-induced spreading in the GFP$^-$YFP$^-$ double-negative gate. This increased spread contributes to higher rSD and gating challenges in the YFP SC control. Biaxial gating of YFP versus GFP improves separation and enables resolution of GFP$^+$, YFP$^+$, and double-negative populations.

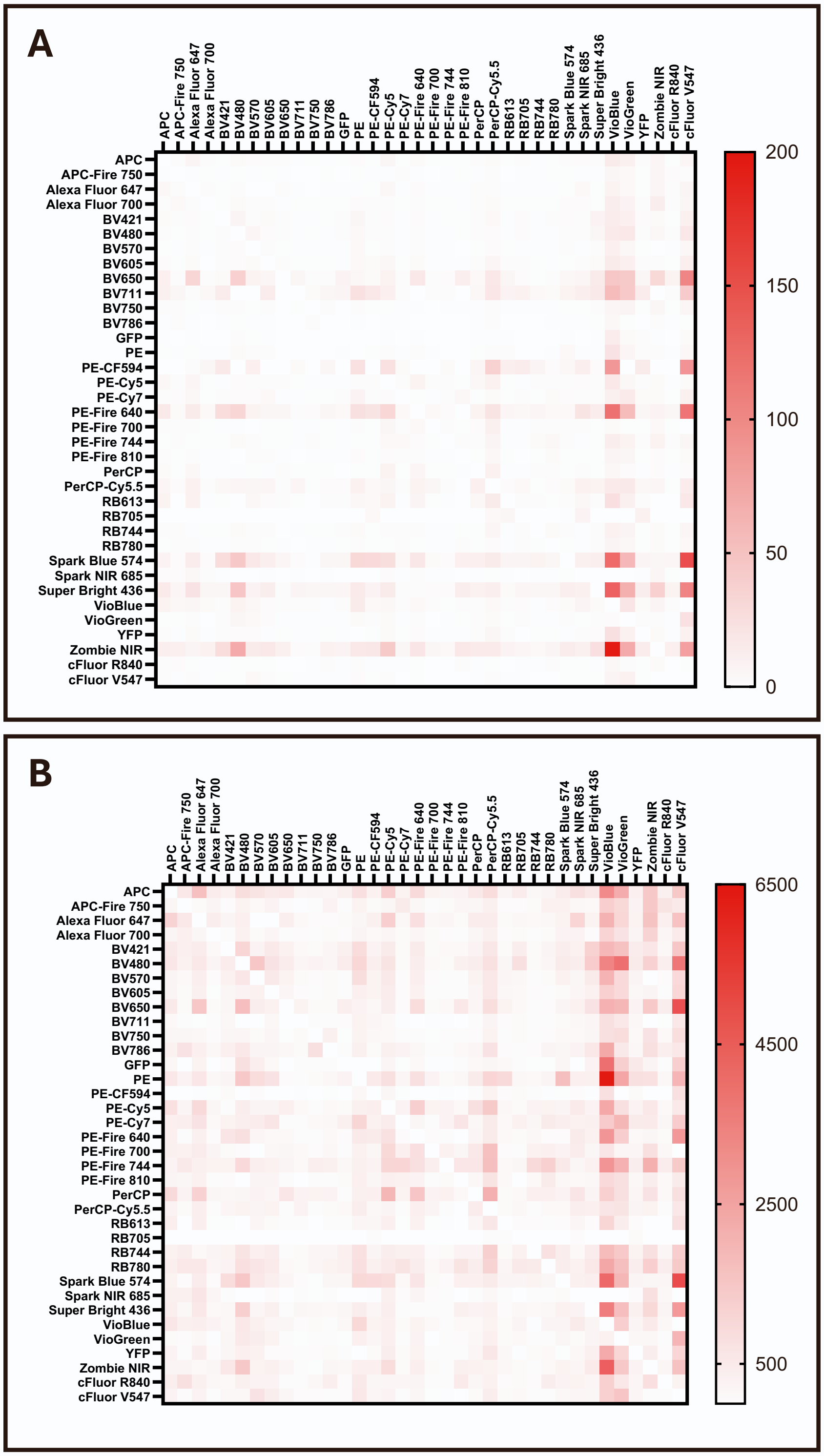

Figure S24. Spillover spreading and total spread matrices for the final 36-color panel.

(**A**) Spillover spreading matrix (SSM) and (**B**) total spread matrix (TSM) calculated from unmixed single-color stained cells for each fluorochrome in the 36-color panel. The SSM identifies potential spreading errors resulting from marker co-expression. The TSM highlights fluorochromes that contribute to spreading error due to spectral overlap or brightness.

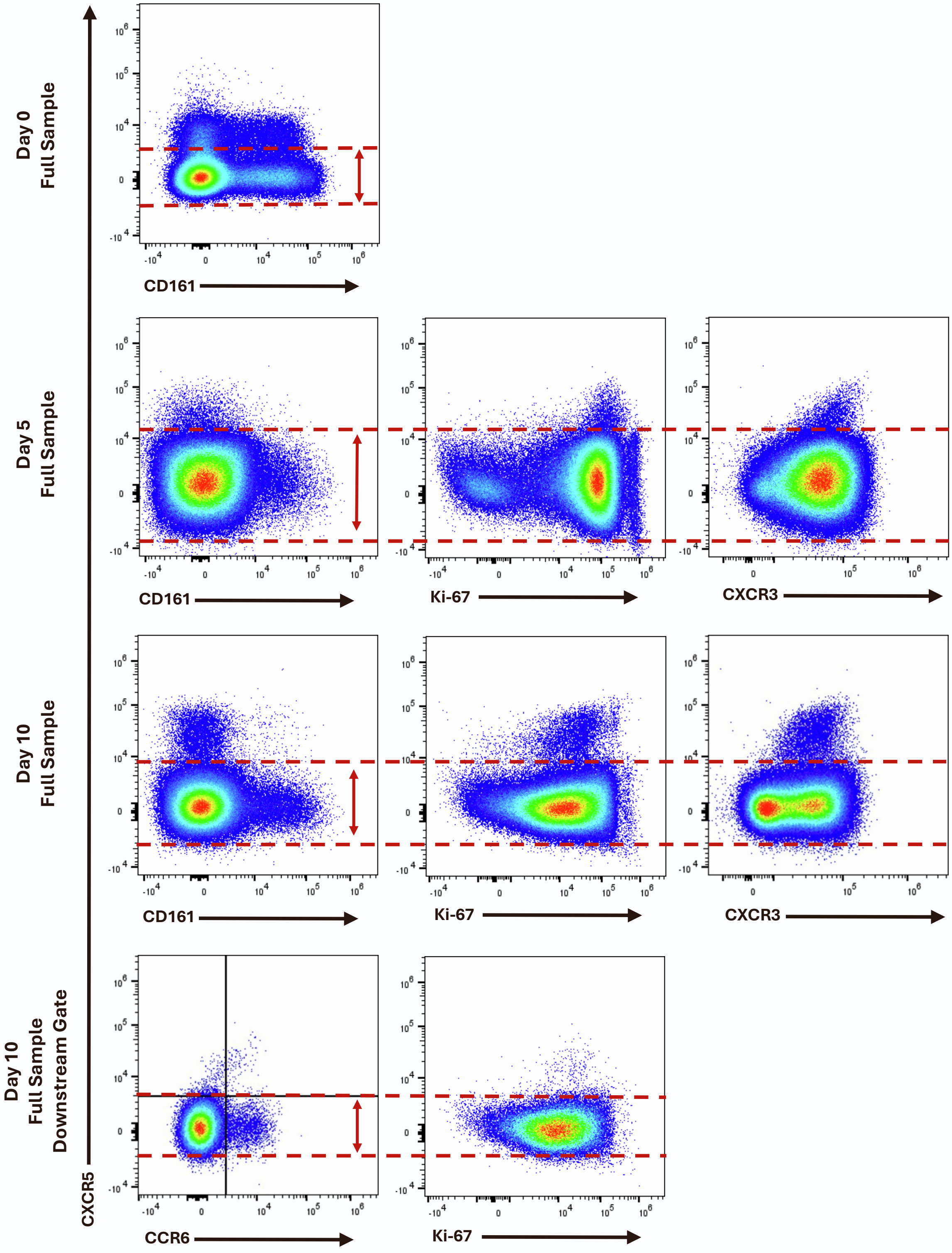

Figure S25. Improved CXCR5 resolution via downstream gating strategy.

CXCR5 resolution across 10 days of manufacturing or co-culture in a representative donor. Rows 1–3 show CXCR5 detection gated from total CD45^{+} cells. Row 4 depicts the same sample gated from a downstream population based on the full gating hierarchy. This approach reduces spectral spread originating from CXCR3 (PE-Cy5), thereby improving CXCR5 (PE-Cy7) resolution.

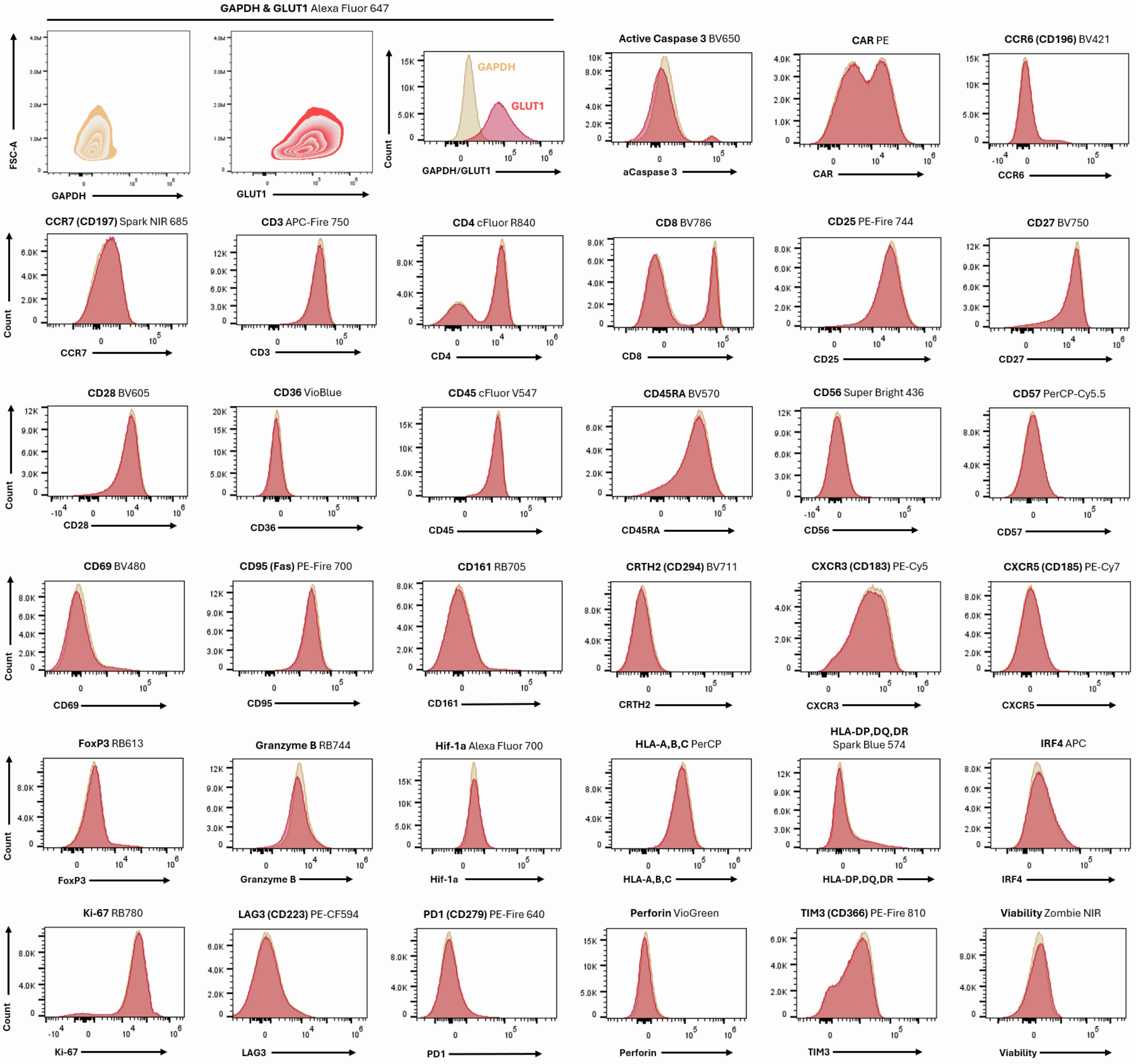

Figure S26. Marker expression profiles using GAPDH or GLUT1 (AF647) in spectral cytometry.

Histogram overlays showing expression of all 36 markers in day 5 CAR$^+$ T cells incorporating either GAPDH or GLUT1 conjugated to Alexa Fluor 647 in the fully stained sample. Markers are grouped by functional category and displayed as normalized fluorescence intensity histograms. Data represent fully stained CAR$^+$ cells from a single donor (Donor C, day 5 manufacturing). Fluorescence profiles for all markers were acquired under identical staining conditions and gating strategy.

Supplemental References

1. Sadelain, M., Rivière, I. & Brentjens, R. Targeting tumours with genetically enhanced T lymphocytes. *Nat Rev Cancer* **3**, 35–45 (2003).
2. Turtle, C. J. *et al.* CD19 CAR–T cells of defined CD4+:CD8+ composition in adult B cell ALL patients. *Journal of Clinical Investigation* **126**, 2123–2138 (2016).
3. Lohoff, M. *et al.* Dysregulated T helper cell differentiation in the absence of interferon regulatory factor 4. *Proceedings of the National Academy of Sciences* **99**, 11808–11812 (2002).
4. Quigley, M. F., Gonzalez, V. D., Granath, A., Andersson, J. & Sandberg, J. K. CXCR5 + CCR7 – CD8 T cells are early effector memory cells that infiltrate tonsil B cell follicles. *Eur J Immunol* **37**, 3352–3362 (2007).
5. Tsuda, H. *et al.* A novel surface molecule of Th2- and Tc2-type cells, CRTH2 expression on human peripheral and decidual CD4+ and CD8+ T cells during the early stage of pregnancy. *Clin Exp Immunol* **123**, 105–111 (2001).
6. Cosmi, L. *et al.* Human interleukin 17–producing cells originate from a CD161+CD4+ T cell precursor. *J Exp Med* **205**, 1903–1916 (2008).
7. Liu, Y. *et al.* Interleukin-21 induces the differentiation of human Tc22 cells via phosphorylation of signal transducers and activators of transcription. *Immunology* **132**, 540–548 (2011).
8. Good, Z. *et al.* Post-infusion CAR TReg cells identify patients resistant to CD19-CAR therapy. *Nat Med* **28**, 1860–1871 (2022).
9. Lanier, L. L., Testi, R., Bindl, J. & Phillips, J. H. Identity of Leu-19 (CD56) leukocyte differentiation antigen and neural cell adhesion molecule. *J Exp Med* **169**, 2233–2238 (1989).
10. Rufer, N. *et al.* Ex vivo characterization of human CD8+ T subsets with distinct replicative history and partial effector functions. *Blood* **102**, 1779–1787 (2003).
11. D'Ambrosio, D. *et al.* Transcriptional regulation of interleukin-2 gene expression by CD69-generated signals. *Eur J Immunol* **23**, 2993–2997 (1993).
12. Soares, A. *et al.* Novel application of Ki67 to quantify antigen-specific in vitro lymphoproliferation. *J Immunol Methods* **362**, 43–50 (2010).
13. Macintyre, A. N. *et al.* The Glucose Transporter Glut1 Is Selectively Essential for CD4 T Cell Activation and Effector Function. *Cell Metab* **20**, 61–72 (2014).
14. Balmer, M. L. *et al.* Memory CD8 + T Cells Require Increased Concentrations of Acetate Induced by Stress for Optimal Function. *Immunity* **44**, 1312–1324 (2016).
15. Palazon, A. *et al.* An HIF-1α/VEGF-A Axis in Cytotoxic T Cells Regulates Tumor Progression. *Cancer Cell* **32**, 669-683.e5 (2017).
16. Jin, H.-T. *et al.* Cooperation of Tim-3 and PD-1 in CD8 T-cell exhaustion during chronic viral infection. *Proceedings of the National Academy of Sciences* **107**, 14733–14738 (2010).
17. Ma, X. *et al.* CD36-mediated ferroptosis dampens intratumoral CD8+ T cell effector function and impairs their antitumor ability. *Cell Metab* **33**, 1001-1012.e5 (2021).
18. Brenchley, J. M. *et al.* Expression of CD57 defines replicative senescence and antigen-induced apoptotic death of CD8+ T cells. *Blood* **101**, 2711–2720 (2003).
19. Porter, A. G. & Jänicke, R. U. Emerging roles of caspase-3 in apoptosis. *Cell Death Differ* **6**, 99–104 (1999).
20. Hermans, I. F. *et al.* The VITAL assay: a versatile fluorometric technique for assessing CTL- and NKT-mediated cytotoxicity against multiple targets in vitro and in vivo. *J Immunol Methods* **285**, 25–40 (2004).
21. Depil, S., Duchateau, P., Grupp, S. A., Mufti, G. & Poirot, L. 'Off-the-shelf' allogeneic CAR T cells: development and challenges. *Nat Rev Drug Discov* **19**, 185–199 (2020).
