# Supplementary material for: High-dimensional temporal mapping of CAR T cells reveals phenotypic and functional remodeling during manufacturing
Source: Mol Ther. 2025 May 1;33(5):2291–309. doi: 10.1016/j.ymthe.2025.04.006 (PMC12126796; doi:10.1016/j.ymthe.2025.04.006)
Supplement: Document S2. Article plus supplemental information [file mmc2.pdf]

Molecular Therapy
Original Article

High-dimensional temporal mapping of CAR T cells reveals phenotypic and functional remodeling during manufacturing

Amaia Cadinanos-Garai,[1] Christian L. Flugel,[1,2] Anson Cheung,[1] Enzi Jiang,[1] Alix Vaissié,[1] and Mohamed Abou-el-Enein[1,3,4,5]

[1]USC/CHLA Cell Therapy Program, University of Southern California and Children's Hospital of Los Angeles, Los Angeles, CA 90033, USA; [2]Berlin Center for Advanced Therapies, Charité-Universitätsmedizin Berlin, Corporate Member of Freie Universität Berlin, Humboldt-Universität zu Berlin, and Berlin Institute of Health, 13353 Berlin, Germany; [3]Division of Medical Oncology, Norris Comprehensive Cancer Center, Keck School of Medicine, University of Southern California, Los Angeles, CA 90033, USA; [4]Department of Stem Cell Biology and Regenerative Medicine, Keck School of Medicine, University of Southern California, Los Angeles, CA 90033, USA; [5]Department of Regulatory and Quality Sciences, Alfred E. Mann School of Pharmacy and Pharmaceutical Sciences, University of Southern California, Los Angeles, CA 90033, USA

Despite the notable success of chimeric antigen receptor (CAR) T cell therapies in hematological malignancies, clinical outcomes remain variable, making it critical to understand how manufacturing influences product composition and function. We developed a 36-marker spectral flow cytometry panel enabling integrated profiling of phenotypic, metabolic, and functional attributes across CAR T cell production. Mid-expansion products (day 5) retained stem-like, metabolically active $CD4^+$ Th1 subsets with high proliferative capacity, whereas prolonged culture (day 10) enriched terminally differentiated $CD8^+$ Tc1 cells and NK-like T cell populations. CAR^+ and CAR^- T cells showed similar differentiation trajectories, suggesting that culture conditions may have a larger impact on phenotypic remodeling than CAR integration. Upon antigen encounter and restimulation, day 5 and day 10 products showed comparable cytotoxicity, while differing in their activation and checkpoint profiles. Cryopreservation modestly affected stem cell memory, activation, and metabolic markers but preserved overall phenotype and cytotoxic function. These findings establish a high-dimensional framework for mapping CAR T cell dynamics to support manufacturing optimization and next-generation cell therapy design.

INTRODUCTION

Chimeric antigen receptor (CAR) T cell therapies have redefined the treatment landscape for hematologic malignancies. In B cell malignancies such as acute lymphoblastic leukemia and diffuse large B cell lymphoma, CD19-targeted CAR T cells induce high remission rates; however, response durability remains inconsistent, with many patients relapsing within 1 year of treatment.[1–5] These outcomes are shaped by a complex interplay of tumor-intrinsic mechanisms, T cell fitness, and manufacturing variables. Mechanisms such as antigen loss facilitate immune evasion, ultimately undermining the durability of remission.[6] Likewise, T cell-intrinsic dysfunctions, particularly exhaustion and loss of stemness, limit CAR T cell potency and compromise long-term persistence.[7] An often-overlooked contributor to this variability is the potential impact of the CAR T cell manufacturing process. Reports of second primary malignancies following CAR T cell therapy further stress the need to better understand how manufacturing processes influence T cell fate, particularly in cases involving CAR^+ T cell lymphomas.[8]

All US Food and Drug Administration-approved CAR T cell therapies rely on *ex vivo* expansion of autologous T cells, which are inherently heterogeneous and vary substantially in phenotype and composition across individuals.[9,10] Donor-specific factors, including age, gender, comorbidities, and prior treatments, further shape the immunological profile of the T cells used for manufacturing.[11,12] Emerging evidence suggests that these variations contribute to disparate clinical outcomes.[13–15] For instance, CAR T cell products enriched in memory-like T cells tend to persist longer and mediate superior antitumor responses,[15–17] while an abundance of exhausted T cells correlates with poor persistence and early relapse.[13,18] Similarly, the presence of suppressive regulatory T cells (Tregs) in a CAR T cell product may impair antitumor efficacy.[19] Despite these insights, defining the optimal phenotypic and functional profile of a CAR T cell product remains a major challenge.

Standard quality control assays for CAR T manufacturing, such as CAR expression, CD4:CD8 ratios, and *in vitro* cytotoxicity offer only a limited snapshot of the final product.[20,21] While sufficient for regulatory release, these measures do not capture key attributes

Received 4 March 2025; accepted 2 April 2025;
https://doi.org/10.1016/j.ymthe.2025.04.006.

Correspondence: Mohamed Abou-el-Enein, USC/CHLA Cell Therapy Program, University of Southern California and Children's Hospital of Los Angeles, Los Angeles, CA 90033, USA.
E-mail: mohamed.abouelenein@med.usc.edu

Published by Elsevier Inc. on behalf of The American Society of Gene and Cell Therapy.
This is an open access article under the CC BY license (http://creativecommons.org/licenses/by/4.0/).

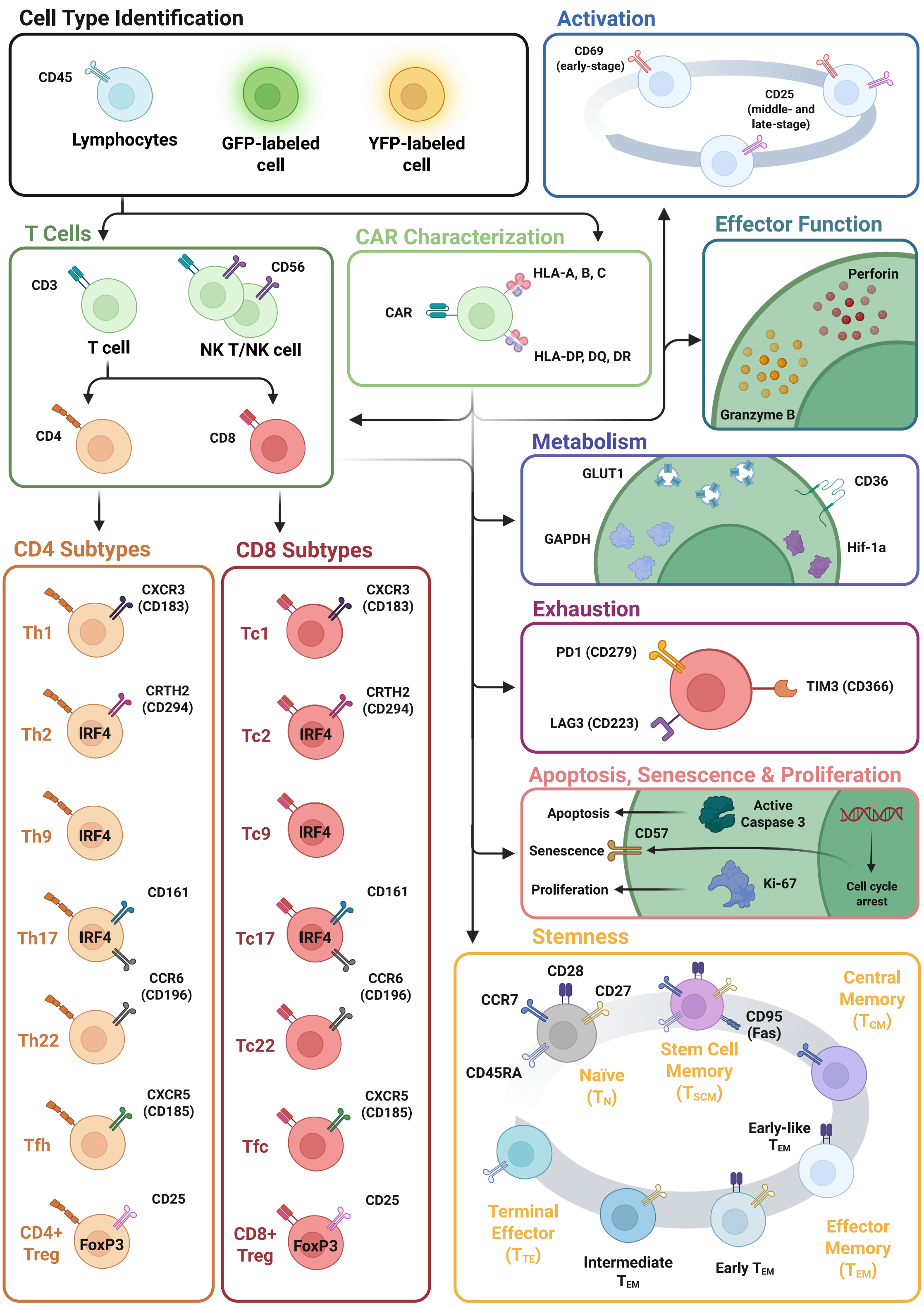

(legend on next page)

of CAR T cells such as differentiation state, checkpoint expression, or metabolic activity, which evolve throughout manufacturing and shape early functional responses.[14] Although several studies have provided valuable insights into determinants of response using patient-derived CAR T cells, most have focused on final product samples, with limited evaluation of how these features evolve throughout manufacturing.[15–17] As a result, little is known about when CAR T cells acquire peak functional attributes during expansion or how key phenotypic programs are modulated across distinct manufacturing stages. This lack of high-dimensional temporal resolution limits our ability to detect critical differences between products and represents a missed opportunity to link cellular dynamics with therapeutic efficacy.

To address these gaps, we developed and applied a 36-marker spectral flow cytometry panel to profile CAR T cells across the manufacturing timeline and in response to antigen stimulation. Designed as an integrated single assay, it simultaneously captures high-dimensional immunophenotyping and *in vitro* cytotoxicity, generating a detailed fingerprint of the CAR T cell product. This approach enables us to reveal key biological transitions that may inform manufacturing optimization and guide product design.

RESULTS

The 36-marker spectral flow cytometry panel provided a comprehensive view of CAR T cell phenotypic evolution from starting donor material through genetic engineering, expansion, and functional testing (Figure 1). Table S1 summarizes the panel design and antibody specifications.

Longitudinal profiling of CAR T cell manufacturing

We generated anti-CD19 CAR T cells from six healthy donors via lentiviral transduction and tracked their phenotypes at day 0 (pre-transduction), day 5 (mid-expansion), and day 10 (final harvest) (Figure 2A). Functional responses were assessed by co-culturing day 5 and day 10 products with $CD19^+$ Nalm6 cells expressing yellow fluorescent protein (YFP^+) as targets and $CD19^-$ Nalm6 cells expressing green fluorescent protein (GFP^+) as controls (Figure 2B). In three donors, CAR T cells underwent an additional antigen rechallenge to assess the effects of repeated stimulation. In this assay, day 5 and day 10 products were exposed to fresh $CD19^+$ targets at 12 h, followed by sample collection at 15 h (3 h post-restimulation) and 24 h. Parallel wells without a second stimulation served as non-restimulated controls (Figure 2B).

Proliferative and metabolic features define CD4:CD8 dynamics in CAR T cell expansion

Transduction and expansion were robust in all donors, with efficient CAR expression by day 5 and sustained viability (Figures 2C and S1A). At this time point, 40.9%–57.3% of T cells were CAR^+, increasing to 44.6%–72.6% by day 10 ($p < 0.05$; Figure 2C). CAR density was high at day 5 (median fluorescence intensity [MFI] ∼23,300), with a modest decrease by day 10 (Figure S1B). $CD4^+$:$CD8^+$ ratios remained largely unchanged between days 0 and 5 (Figure 2D). By day 10, CAR^+CD8^+ cells were selectively enriched ($p < 0.01$), while the CAR^-CD4^+ population contracted ($p < 0.05$; Figure 2D), reflecting divergent expansion dynamics between transduced and non-transduced subsets. CAR^+ T cells expressed higher levels of GLUT1 than CAR^- cells at day 5, indicating elevated glycolytic activity (Figure 2E). A subset of CAR^-CD4^+ cells at day 5 lacked Ki-67 expression, suggesting a non-proliferative phenotype that may contribute to the relative contraction of this compartment by day 10 (Figure S1C). This is consistent with the reduced GLUT1 expression in CAR^-CD4^+ cells. While GLUT1 expression in CAR^+ cells declined by day 10, levels remained elevated compared to day 0, indicating sustained metabolic activity (Figure 2E).

Extended CAR T cell expansion promotes a shift from Th1 to Tc1-dominant phenotypes

We next examined how T cell subsets evolved during manufacturing. At baseline (day 0), the dominant population lacked lineage-defining markers ("all negative" [AN]: $CXCR3^-IRF4^-CRTH2^-CCR6^-CXCR5^-$), although discrete T helper 1 (Th1), T cytotoxic 1 (Tc1), and T helper 22 (Th22) subsets were present (Figure 2F). By day 5, Th1 cells significantly increased compared to day 0 ($p < 0.001$; Figure 2F), while the AN population declined sharply ($p < 0.001$ for CAR^+CD4^+ AN reduction), as more cells expressed defined lineage markers. By day 10, Th1 frequencies returned to baseline ($p < 0.0001$ for day 5 vs. day 10), and the CAR^+CD4^+ AN cells rebounded ($p < 0.01$). Tc1 cells showed a gradual and sustained increase, becoming the dominant subset by day 10 (Figure 2F). T helper 9 (Th9) cells, initially present at low frequency, showed a modest rise by day 5, followed by a return to baseline by day 10 ($p < 0.05$, day 5 vs. day 10). These trends were consistent in both CAR^+ and CAR^- populations, indicating that CAR transduction did not skew

Figure 1. Overview of CAR T cell phenotyping framework for panel design

Schematic representation of the 36-marker spectral flow cytometry panel used to profile anti-CD19 CAR T cells. The panel is organized by biological function and phenotypic relevance, encompassing key modules for cell identification, CAR characterization, lineage assignment, functional state, and differentiation status. CD3, CD4, and CD8 distinguish T cell subsets; CD56 identifies NK/NK-like T cells. YFP and GFP reporters are used to track $CD19^+$ Nalm6-YFP^+ target cells and $CD19^-$ Nalm6-GFP^+ control cells in co-culture and restimulation assays. CAR expression is detected alongside HLA-A, B, C and HLA-DP, DQ, DR to assess transgene expression and HLA knockout in allogeneic products. Functional states include activation markers (CD69, CD25), effector function mediators (granzyme B, perforin), and metabolic activity (GLUT1, GAPDH, CD36, Hif-1a). Exhaustion (PD1, LAG3, TIM3), senescence (CD57), proliferation (Ki-67), and apoptosis (active caspase 3) are tracked alongside memory and differentiation states. Naïve (T_N), stem cell memory (T_{SCM}), central memory (T_{CM}), effector memory (T_{EM}; early-like, early, and intermediate), and terminal effector (T_{TE}) subsets are defined by CD45RA, CCR7, CD95, CD27, and CD28. $CD4^+$ and $CD8^+$ subsets are further resolved into Th and Tc lineages, including Th1/Tc1, Th2/Tc2, Th9/Tc9, Th17/Tc17, Th22/Tc22, Tfh/Tfc, and Tregs.

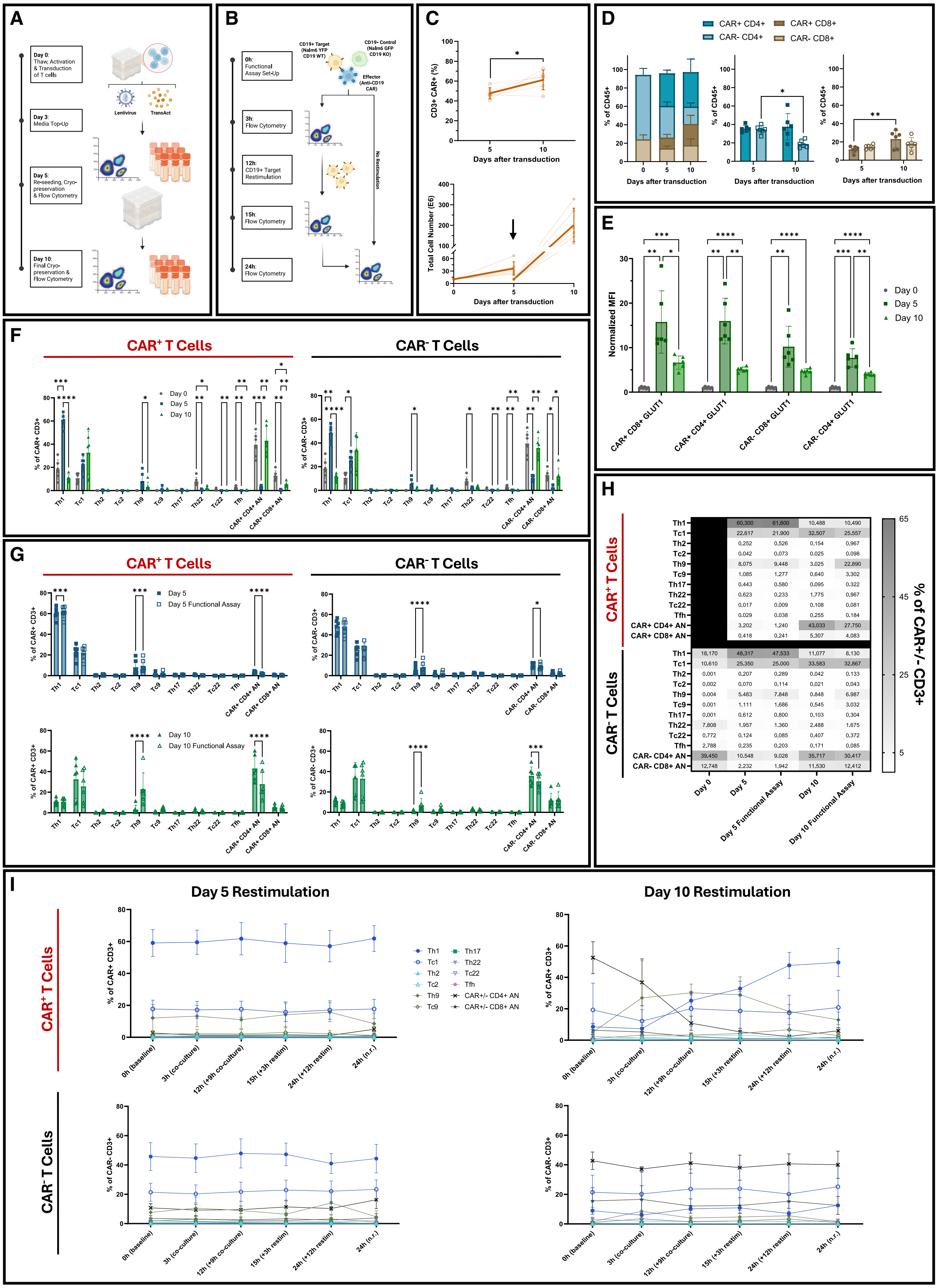

(legend on next page)

helper or cytotoxic lineage differentiation. Interferon regulatory factor 4 (IRF4) expression, which was nearly absent at day 0, became significantly upregulated by day 5, particularly within the CAR$^+$ CD4$^+$ subset ($p < 0.01$; Figure S1D). IRF4 upregulation was most prominent in CXCR3$^+$ Th1 and Tc1 cells (Figure S1F), suggesting a role in metabolic or proliferative support of these expanding subsets.

Cytotoxic challenge induces minimal shifts in CAR T cell subtype composition

Day 5 and day 10 products were co-cultured with CD19$^+$ targets and CD19$^-$ controls for 3 h, followed by phenotypic analysis. Despite robust functional activation, overall subset composition remained similar to the pre-assay state (Figure 2G). In day 5 CAR T cells, Th and Tc subset levels were largely unchanged following antigen exposure. Th1 and Tc1 cells remained the predominant subsets in day 5 and day 10 products, respectively (Figure 2H). One notable change was a significant increase in Th9 cells in the day 10 product ($p < 0.0001$ for 0 h vs. 3 h), consistent with IRF4 upregulation upon antigen exposure (Figure S1E). Overall, brief target engagement triggered effector functions while preserving the original T cell subtype distribution, indicating that short-term antigen encounter does not alter lineage composition.

Extended antigen exposure triggers subtype reprogramming in CAR T cells

To assess whether repeated antigen exposure induces phenotypic changes, day 5 and day 10 CAR T cells were rechallenged with fresh CD19$^+$ targets at 12 h and analyzed at 15 h (3 h post-restimulation) and 24 h ($n = 3$ donors). In day 5 CAR T cells, subset frequencies remained relatively stable, with a persistent Th1-dominant profile (Figures 2I, left, and S2A). Tc1 and Th9 subsets contributed to a lesser extent. In contrast, day 10 CAR T cells exhibited more dynamic changes, including progressive enrichment of Th1 cells and maintenance of the Tc1 compartment (Figures 2I, right, and S2A). Th1 frequency gradually increased, ultimately reaching levels nearing those observed in day 5 cells ($p < 0.05$, 3 h vs. 12 h/15 h). In parallel, Th9 cells exhibited a transient peak ($p < 0.0001$, 0 h vs. 12 h), followed by a decline after 15 h. These shifts were accompanied by a progressive reduction in the CD4$^+$ AN population starting at 3 h co-culture through 24 h (Figure 2I, right, and S2A). Phenotypic shifts were most pronounced in the CAR$^+$ fraction and remained comparable between restimulated and non-restimulated cells at 24 h.

Stem-like memory peaks mid-expansion, followed by terminal effector enrichment

We next evaluated how CAR T cell memory states evolved over time and in response to antigen encounter. Using CD45RA, CCR7, CD95, CD27, and CD28 expression, we observed a progressive shift from naïve and early effector memory subsets toward more differentiated phenotypes over the 10-day expansion period (Figures 3A and 3B). The naïve population (33.1% at day 0) declined significantly and fell below 0.1% of CAR$^+$ cells by day 5, consistent with activation-induced differentiation (Figure 3B). As naïve cells contracted, T stem cell memory (T_{SCM}; CD45RA$^+$CCR7$^+$CD27$^+$CD28$^+$CD95$^+$) increased to 25.0% of CAR$^+$ cells by day 5 ($p < 0.01$, day 0 vs. day 5), with 11.5% persisting at day 10, a subset associated with long-term CAR T cells' persistence.[22] Early effector memory cells (T_{EM}; CD45RA$^-$CCR7$^-$CD27$^+$CD28$^+$) made up 35.3% at day 0, decreased by day 5, and remained stable thereafter. Central memory cells (T_{CM}; CD45RA$^-$CCR7$^+$) peaked at day 5 and declined to 1.7% by day 10 ($p < 0.05$, day 5 vs. day 10). Terminal effector cells (T_{TE}; CD45RA$^+$CCR7$^-$) expanded from 36.3% at day 5 to 61.5% by day 10 ($p < 0.01$), becoming the predominant population. These patterns were also observed in CAR$^-$ T cells, indicating that phenotypic changes were largely driven by shared *in vitro* activation and expansion conditions (Figures 3A and 3B).

Following 3 h co-culture, the memory composition of CAR$^+$ and CAR$^-$ T cells remained largely unchanged (Figure 3C). In donors that underwent antigen rechallenge ($n = 3$) and extended stimulation for up to 24 h, day 5 CAR T cells maintained a predominantly T_{TE} phenotype at both 15 and 24 h, with only modest

Figure 2. Experimental design and CAR T cell phenotypic profiling

(A) Schematic of the manufacturing workflow. T cells from healthy donors were activated and transduced with anti-CD19 CAR on day 0 and harvested on days 5 and 10 for spectral flow cytometry and functional assays. (B) Functional assays included a 3 h co-culture with CD19$^+$ Nalm6-YFP$^+$ target and CD19$^-$ Nalm6-GFP$^+$ control cells at days 5 and 10, and a restimulation assay in which fresh CD19$^+$ targets were added at 12 h (9 h post-initial co-culture), followed by sample collection at 15 h (3 h post-restimulation) and 24 h. (C) Top: percentage of CD3$^+$CAR$^+$ expression at day 5 and day 10 post-transduction, shown out of CD45$^+$ population ($n = 6$ donors, 2 independent experiments). Bold line represents mean ± SD. Bottom: total cell yield at days 5 and 10. Bold arrow highlights day 5 sample harvest and re-seeding ($n = 6$ donors, 2 independent experiments). (D) Proportion of CD4$^+$ and CD8$^+$ cells of CAR$^+$ and CAR$^-$ T cells ($n = 6$ donors, 2 independent experiments). (E) Normalized median fluorescence intensity (MFI) of GLUT1 in CAR$^+$ and CAR$^-$ CD4$^+$ and CD8$^+$ subsets at days 0, 5, and 10 ($n = 6$ donors, 2 independent experiments). MFI were normalized to the average of day 0 MFI. Day 0 CAR$^-$ samples served as the baseline reference. (F) Frequencies of CD4$^+$ helper (Th) and CD8$^+$ cytotoxic (Tc) subsets in CAR$^+$ and CAR$^-$ populations throughout manufacturing ($n = 6$ donors, 2 independent experiments). Day 0 CAR$^-$ samples served as the baseline reference. Only subsets representing ≥0.5% of total CD3$^+$ T cells for any time point or donor are shown. (G) Th and Tc subset frequencies following 3 h co-culture with target and control cells on days 5 (top) and 10 (bottom) products ($n = 6$ donors, 2 independent experiments). (H) Heatmap of CD4$^+$ and CD8$^+$ subset frequencies within CAR$^+$ and CAR$^-$ compartments across all time points and co-culture conditions. AN, all negative (CXCR3$^-$IRF4$^-$CRTH2$^-$CCR6$^-$CXCR5$^-$) ($n = 6$ donors, 2 independent experiments). (I) Kinetics of CD4$^+$ and CD8$^+$ subset frequencies during the 24 h restimulation assay in day 5 and day 10 CAR$^+$ (top) and CAR$^-$ (bottom) T cells ($n = 3$ donors). n.r., non-restimulated; restim, restimulated. Statistical analyses: paired, two-tailed Student's t test (C), two-way repeated measures ANOVA with Tukey's (E, F and I), and Šídák's (D and G) multiple comparisons test. Data represent mean ± SD; *$p < 0.05$; **$p < 0.01$; ***$p < 0.001$; ****$p < 0.0001$. The absence of p-values denotes non-significance at the $p = 0.05$ threshold. All p-values for panel I are provided in Table S6.

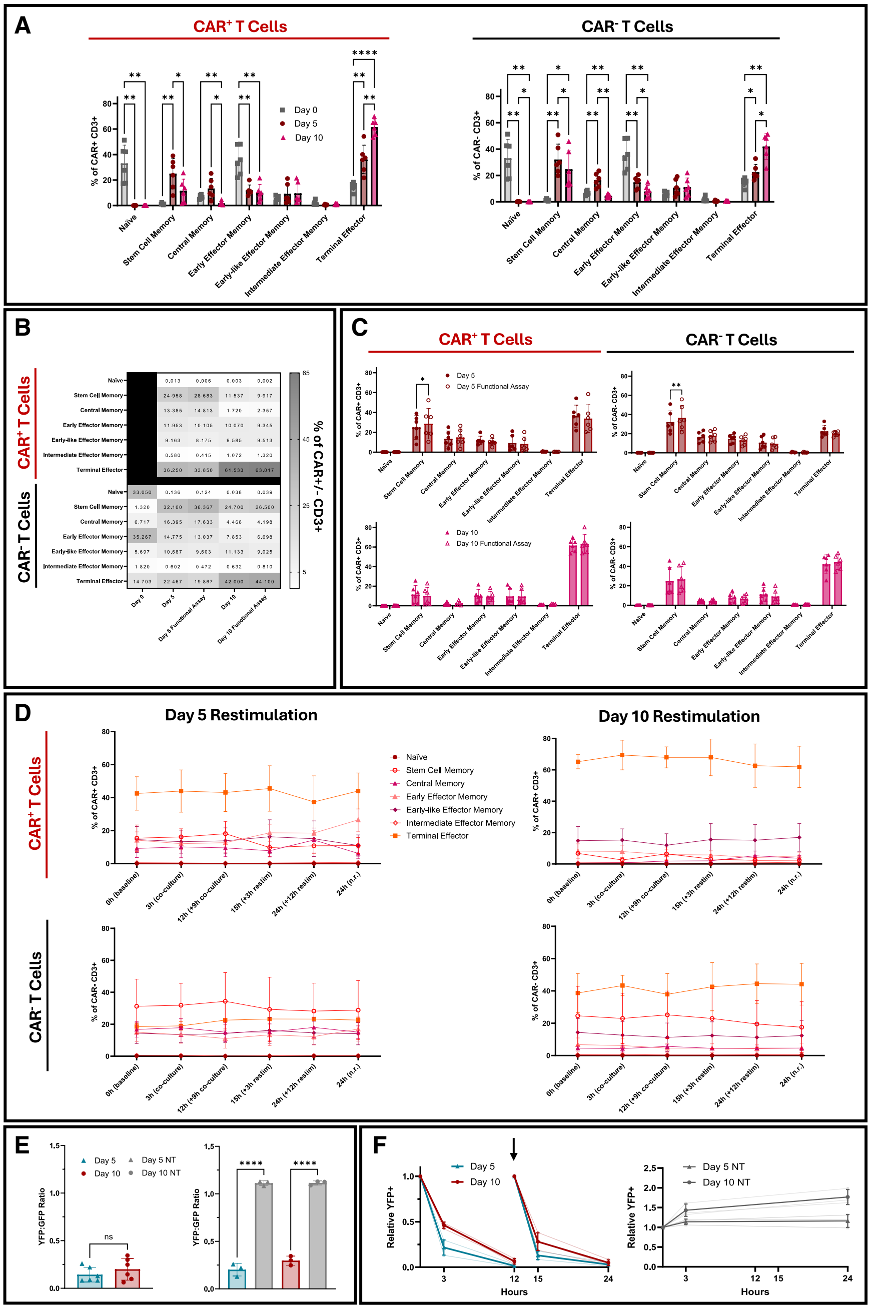

Figure 3. Differentiation trajectories and cytotoxic activity of CAR T cells during manufacturing

(A) Frequencies of T_N, T_{SCM}, T_{CM}, T_{EM}, and T_{TE} subsets within CAR^+ and CAR^- $CD3^+$ T cells over the course of manufacturing (days 0, 5, and 10). Day 0 CAR^- samples served as the baseline reference (n = 6 donors, 2 independent experiments). (B) Heatmap showing the frequency of each differentiation subset throughout manufacturing stages and 3 h co-culture assay (n = 6 donors, 2 independent experiments). (C) Differentiation profile following 3 h co-culture with $CD19^+$ Nalm6-YFP^+ target and $CD19^-$ Nalm6-GFP^+ control cells at days 5 and 10 (n = 6 donors, 2 independent experiments). (D) Differentiation dynamics in day 5 and day 10 CAR^+ and CAR^- T cells during the 24 h restimulation assay (0, 3, 12, 15, and 24 h; n = 3 donors). (E) YFP:GFP ratios from 3 h co-culture assays measuring cytotoxicity of CAR transduced cells (left, n = 6 donors) and CAR transduced versus non-transduced cells (right, n = 3 donors). (F) Left: time course of day 5 and day 10 CAR^+ T cell-mediated killing during restimulation, with antigen additions at 0 and 12 h (n = 3 donors). Black arrow represents restimulation time point. Right: normalized $CD19^+$ Nalm6-YFP^+ target cell frequency during restimulation with non-transduced control cells (n = 3 donors). restim, restimulated. n.r., non-restimulated. Statistical analyses: two-way repeated measures ANOVA with Tukey's (A and D), and Šídák's (C) multiple comparisons test, two-tailed Mann-Whitney U test (E, left), and one-way ANOVA with Šídák's multiple comparisons test (E, right). Data shown as mean ± SD; $*p < 0.05$; $**p < 0.01$; $****p < 0.0001$; ns, not significant. The absence of p-values denotes non-significance at the p = 0.05 threshold. All p-values for panel D are provided in Table S6.

increases in T_{CM}, early effector, and early-like effector memory subsets (Figures 3D and S2B). T_{SCM} frequency remained stable during the initial 3 h co-culture (15.4% at 0 h vs. 16.2% at 3 h) but declined following additional antigen exposure at 12 h and prolonged incubation to 24 h, regardless of restimulation. In day 5 cultures, T_{SCM} frequencies were consistently higher in CAR^- cells (28.2%–34.4%) than in CAR^+ cells (9.7%–18.1%). Day 10 CAR T cells displayed a more terminally differentiated phenotype at baseline and showed minimal phenotypic change at 15 and 24 h, remaining dominated by T_{TE} cells regardless of restimulation. T_{SCM} frequencies in day 10 CAR^+ cells were markedly lower (2.3%–6.8%) compared to their CAR^- counterparts (17.5%–25.2%), further supporting the notion that CAR signaling and antigen exposure promote differentiation away from stem-like states (Figures 3D and S2B). Although not statistically significant, day 5 CAR T cells exhibited stronger cytotoxicity following both initial stimulation and rechallenge, consistent with the increased presence of less-differentiated subsets such as T_{SCM}, T_{CM}, and T_{EM} (Figures 3E and 3F).

CAR T cell expansion drives robust activation with dynamic checkpoint expression

We assessed activation, immune checkpoint expression, senescence, and apoptosis markers in CAR T cells throughout manufacturing and in response to antigen exposure. At baseline (day 0), T cells exhibited a non-activated phenotype (Figure 4A). By day 5, both CAR^+ and CAR^- T cells showed robust upregulation of CD25 relative to

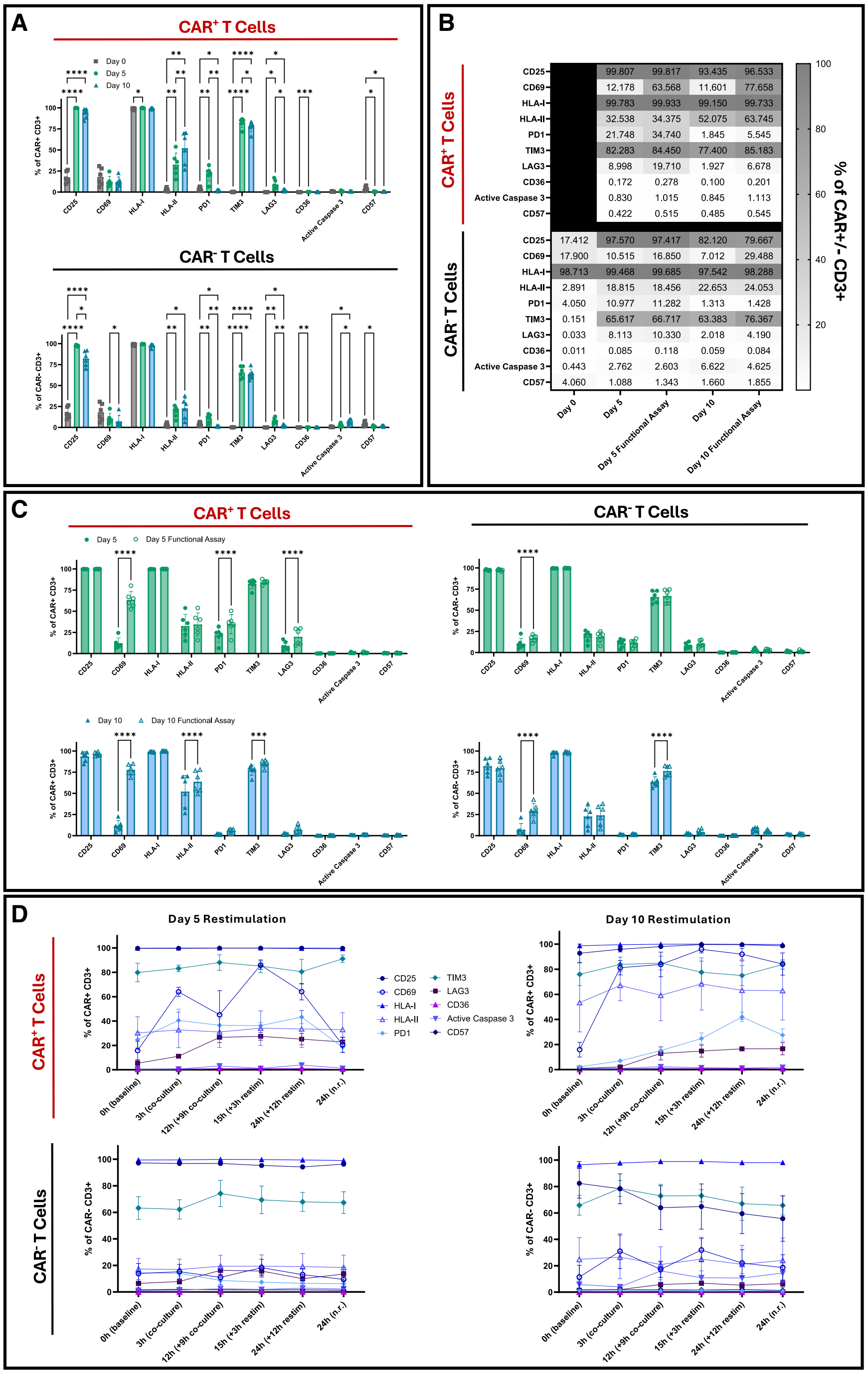

Figure 4. Activation, exhaustion, senescence, and apoptosis marker dynamics during expansion and antigen encounter

(A) Baseline expression of CD25, CD69, HLA class I and II, PD1, TIM3, LAG3, CD36, active caspase 3, and CD57 in CAR$^+$ and CAR$^-$ CD3$^+$ T cells on days 0, 5, and 10 ($n = 6$ donors, 2 independent experiments). Day 0 CAR$^-$ samples served as the baseline reference. (B) Heatmap visualization of marker expression changes over the manufacturing period and 3 h co-culture assay ($n = 6$ donors, 2 independent experiments). (C) Summary of activation, exhaustion, senescence, and apoptosis marker expression following 3 h co-culture on day 5 and day 10 products ($n = 6$ donors, 2 independent experiments). (D) Longitudinal marker expression during the 24 h restimulation assay in CAR$^+$ (top) and CAR$^-$ (bottom) CD3$^+$ T cells ($n = 3$ donors). n.r., non-restimulated; restim, restimulated. Statistical analyses: two-way repeated measures ANOVA with Tukey's (A and D), and Šídák's (C) multiple comparisons test. Data shown as mean ± SD; *$p < 0.05$; **$p < 0.01$; ***$p < 0.001$; ****$p < 0.0001$. The absence of p-values denotes non-significance at the $p = 0.05$ threshold. All p-values for panel D are provided in Table S7.

day 0 ($p < 0.0001$), consistent with strong activation and entry into a proliferative state. High CD25 expression was maintained through day 10, indicating sustained activation during expansion. In contrast, CD69 levels remained low throughout culture. At day 5, 12.2% of CAR$^+$ and 10.5% of CAR$^-$ cells expressed CD69; by day 10, CD69$^+$ frequencies were 11.6% and 7.0%, respectively (Figure 4B). These modest levels reflect the transient nature of CD69 expression following early activation. Human leukocyte antigen (HLA) class I (HLA-A, B, C) expression remained high at all time points. HLA class II (HLA-DP, DQ, DR) expression increased significantly from day 0 to day 10 in both CAR$^+$ ($p < 0.01$) and CAR$^-$ ($p < 0.05$) cells, consistent with activation-induced upregulation. Notably, CAR$^+$ T cells expressed higher levels of HLA class II compared to CAR$^-$ T cells (Figure 4A). Among exhaustion markers, TIM3 was the most prominently expressed. While low at day 0, TIM3 became elevated by day 5 ($p < 0.0001$) and remained high through day 10. PD1 and LAG3 showed only transient changes, with modest upregulation by day 5 (PD1: $p < 0.01$; LAG3: $p < 0.05$) that returned to near-baseline by day 10 (Figure 4A). Cell viability remained high throughout, with >90% viability on both days 5 and 10 (Figure S1A). Senescence and apoptosis markers remained low in CAR$^+$ T cells, with CD57$^+$ cells at 0.4% on day 5 and 0.5% on day 10, and active caspase 3$^+$ cells consistently low at 0.8% across both timepoints (Figures 4A and 4B). By day 10, CAR$^-$ T cells showed slightly higher levels, with CD57$^+$ cells at 1.7% and active caspase 3$^+$ cells at 6.6%. CD36 remained low in both populations (CD36 positive control, Figure S3).

Upon 3 h co-culture, CD69$^+$CAR$^+$ T cells increased significantly on both days 5 and 10 ($p < 0.0001$, 0 h vs. 3 h) (Figure 4C). CAR$^-$ T cells

also upregulated CD69, likely due to bystander activation via cytokines in culture. CD25 remained high and unchanged in both CAR$^+$ and CAR$^-$ T cells. HLA class II expression further increased in day 10 CAR$^+$ T cells ($p < 0.0001$, 0 h vs. 3 h; Figure 4C). Checkpoint expression during co-culture was subset and time point dependent. On day 5, CAR$^+$ T cells showed increased PD1 ($p < 0.0001$) and LAG3 ($p < 0.0001$). CAR$^-$ T cells displayed minimal PD1 or LAG3 induction, suggesting antigen-dependent exhaustion. On day 10, CAR$^+$ cells showed no further upregulation of PD1 or LAG3. TIM3, which was elevated at baseline, remained high in both CAR$^+$ and CAR$^-$ subsets. Importantly, activation-induced apoptosis and senescence remained low. CD57$^+$ and active caspase 3$^+$ cells did not increase after 3 h of antigen exposure (Figure 4C).

Upon restimulation (12 h), day 5 and day 10 CAR$^+$ T cells exhibited distinct activation kinetics and checkpoint profiles (Figures 4D and S2C). In day 5 products, CD69 expression increased during the initial 3 h co-culture, declined slightly at 12 h, and peaked at 15 h post restimulation. HLA class II and TIM3 remained elevated throughout. PD1 rose from 24.8% at baseline to 40.5% after 3 h co-culture and remained high following restimulation. LAG3, however, showed modest induction at 3 h (5.3% at 0 h vs. 11.1% at 3 h), reaching its highest level at 15 h (27.4%) (Figure S2C). Day 10 CAR$^+$ T cells showed a sharp increase in CD69 expression from 15.9% at baseline to 81.2% after 3 h and remained high through 24 h. HLA class II expression was high and exceeded levels observed in day 5 products, consistent with the more sustained activation profile of day 10 cells. PD1 and LAG3 rose modestly during initial co-culture and increased further after restimulation. Throughout the 24 h assay, day 5 and day 10 CAR$^+$ T cells maintained low active caspase 3 and CD57 expression (Figure S2C). CAR$^-$ T cells expressed high levels of CD25 and TIM3 at baseline but exhibited only a modest change in HLA class II, CD69, PD1, and LAG3, indicating the absence of CAR independent activation. Together, day 5 CAR$^+$ T cells respond more rapidly by upregulating PD1 and LAG3 upon first antigen encounter, whereas day 10 cells show delayed checkpoint expression, emerging only after extended antigen stimulation (Figures 4D and S2C).

Mid-expansion CAR T cells exhibit enhanced proliferation and metabolic activity

In the 3 h co-culture assay, both day 5 and day 10 CAR$^+$ T cell products demonstrated potent target-specific tumor cell killing (Figure 3E). There was no statistically significant difference in overall cytotoxicity between day 5 and day 10 products ($p = 0.39$; Figure 3E), indicating that later harvest did not impair nor improve short-term killing capacity. Both day 5 and day 10 CAR T cells maintained strong cytotoxic activity upon antigen restimulation (12 h) and during prolonged exposure up to 24 h (Figure 3F). These data demonstrate that under a Nalm6 model, CAR T cells expanded for 5 days are as potent as those expanded for 10 days and remain capable of sustained tumor clearance *in vitro*.

To evaluate the cytotoxic machinery, we analyzed intracellular granzyme B and perforin levels. At baseline (day 0), both markers were minimally expressed (Figure 5A). Perforin remained undetectable through day 5 and day 10 in both CAR$^+$ and CAR$^-$ subsets (Figure 5A; perforin positive control, Figure S3). In contrast, granzyme B peaked at day 5 ($p < 0.05$ day 0 vs. day 5) and declined by day 10 ($p < 0.05$ day 0 vs. day 10). Following 3 h of co-culture, granzyme B levels dropped mainly in day 10 product (Figures 5B and 5C). During the restimulation assay, granzyme B levels modestly increased prior to second antigen exposure (normalized MFI: 174.1 for day 5, 42.5 for day 10; Figure S2D), then decreased again following rechallenge (normalized MFI: 100.9 for day 5, 40.2 for day 10; Figure S2D). By 24 h, granzyme B reached peak levels in restimulated cells from both day 5 and day 10 products (MFI 421.4 for day 5, 101.7 for day 10). This suggests that CAR$^+$ T cells, particularly day 5 cells, can replenish and enhance their cytolytic payload after repeated antigen encounter (Figures 5D and S2D).

We next assessed metabolic fitness as a determinant of sustained T cell function. Hif-1a expression remained low during expansion and after 3 h of co-culture (Figures 5E and 5F), indicating minimal hypoxic stress. In contrast, GLUT1 was upregulated upon activation, peaking at day 5 ($p < 0.01$), then declining by day 10 ($p < 0.01$) while remaining above baseline ($p < 0.001$, day 0 vs. day 10; Figures 5E and 5G). Notably, switching from GLUT1 to GAPDH confirmed glycolytic pathway engagement, with a similar expression pattern (Figure S4, $n = 1$ donor). Ki-67 expression also peaked at day 5 ($p < 0.001$) and markedly declined by day 10 ($p < 0.001$). After 3 h of co-culture, both day 5 and day 10 CAR T cells showed minimal changes in Ki-67 and GLUT1 (Figure 5F). GLUT1 levels in CAR$^+$ T cells gradually increased from 0 h through 24 h in both day 5 and day 10 products (Figures 5H and S2E), with a significant rise occurring after antigen restimulation (3 h vs. 15 h; day 5: $p < 0.05$; day 10: $p < 0.01$). Ki-67 expression remained stable, with minimal levels detected in day 10 CAR$^+$ T cells. These findings indicate that day 5 CAR$^+$ T cells exhibit higher metabolic and proliferative capacity.

Tregs decline while NK-like traits emerge over CAR T cell expansion periods

CD4$^+$ Tregs (CD25$^+$FoxP3$^+$) comprised approximately 4.3% of total CD4$^+$ T cells at baseline (day 0), while CD8$^+$CD25$^+$FoxP3$^+$ cells were rare (<0.1%) (Figures 6A and 6E). By day 5, CD4$^+$ Tregs accounted for 3.2% in the CAR$^+$ fraction and 5.8% in the CAR$^-$ fraction, but frequencies declined in both compartments by day 10 to 1.1% and 2.0%, respectively (Figure 6E). Following the 3 h co-culture, overall Treg frequencies remained stable. However, in two out of six donors at day 10, a modest but statistically significant increase in CAR$^+$CD4$^+$ Tregs was observed ($p < 0.05$; Figure 6B). In the 24 h restimulation assay, CD4$^+$ Tregs increased in both CAR$^+$ and CAR$^-$ fractions on day 5 and day 10 products (Figures 6F and S2F). Natural killer (NK)-like (CD3$^+$CD56$^+$) T cells were nearly absent at baseline (<0.1%) and remained low through day 5 (<1%) in both CAR$^+$ and CAR$^-$ compartments. By day 10, CD56 expression increased overall,

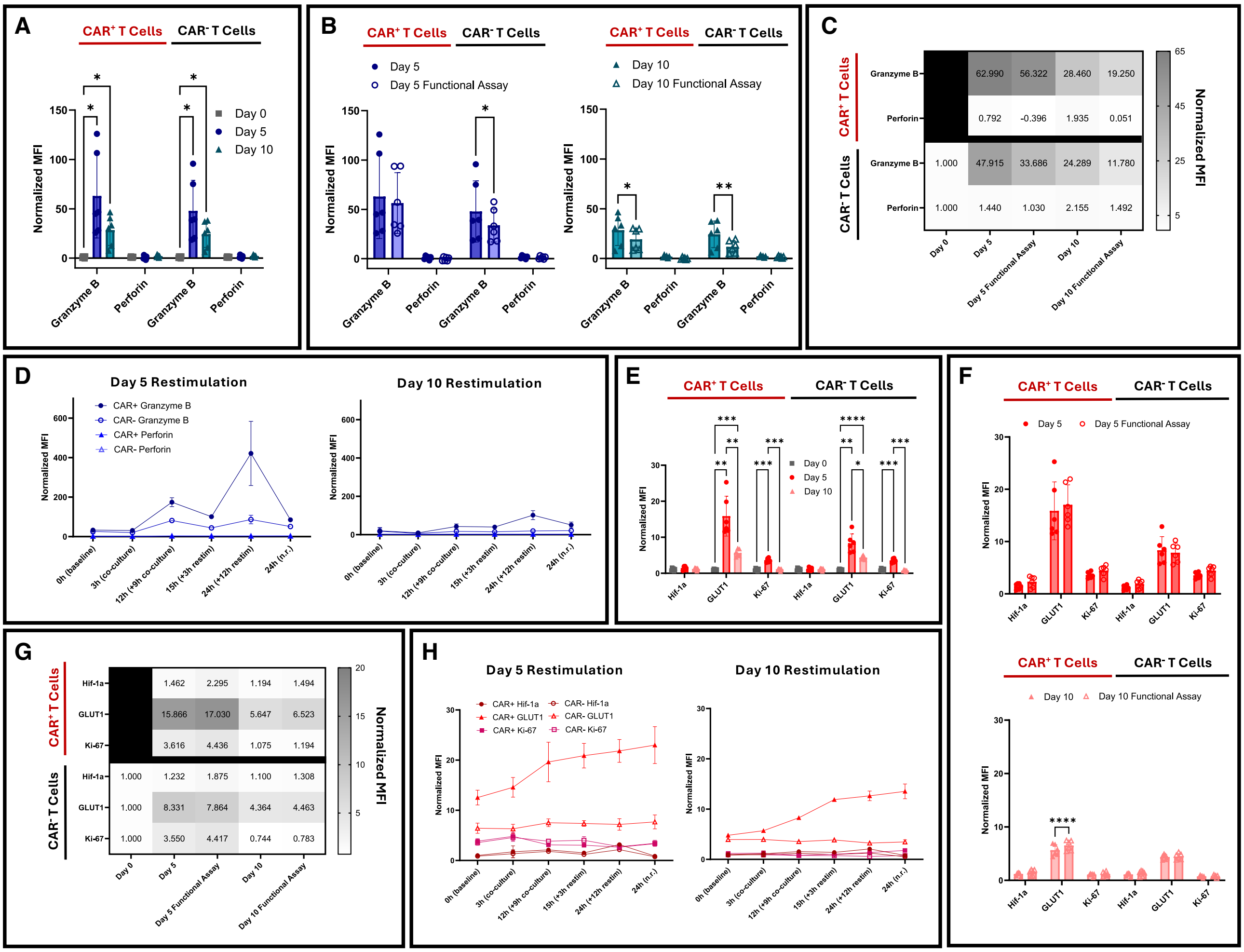

Figure 5. Functional and metabolic activity of CAR T cells during manufacturing

(A and B) Intracellular granzyme B and perforin levels in CAR$^+$ and CAR$^-$ T cells at days 0, 5, and 10 (A) and after 3 h co-culture (B) (n = 6 donors, 2 independent experiments). Day 0 CAR$^-$ samples served as the baseline reference for panel A. (C) Heatmap of normalized MFI for granzyme B and perforin across all conditions (n = 6 donors, 2 independent experiments). (D) Granzyme B and perforin kinetics during restimulation in CAR$^+$ and CAR$^-$ T cells (0–24 h, antigen additions at 0 and 12 h; n = 3 donors). (E and F) Expression of GLUT1, Hif-1a, and Ki-67 at days 0, 5, and 10 (E) and after 3 h co-culture (F) (n = 6 donors, 2 independent experiments). Day 0 CAR$^-$ samples served as the baseline reference for panel E. (G) Heatmap showing MFI values for metabolic and proliferative markers across manufacturing and co-culture (n = 6 donors, 2 independent experiments). (H) Kinetic expression of GLUT1, Hif-1a, and Ki-67 over the 24 h restimulation assay in CAR$^+$ and CAR$^-$ T cells (n = 3 donors). All MFIs were normalized to the average of day 0 MFI of the respective marker. n.r., non-restimulated; restim, restimulated. Statistical analyses: two-way repeated measures ANOVA with Tukey's (A, D, E and H), and Šídák's (B and F) multiple comparisons test. Data shown as mean ± SD; *$p < 0.05$; **$p < 0.01$; ***$p < 0.001$; ****$p < 0.0001$. The absence of p-values denotes non-significance at the p = 0.05 threshold. All p-values for panels D and H are provided in Table S7.

with CD56$^+$CAR$^+$ T cells representing an average of 7.4%, compared to 3.6% in the CAR$^-$ fraction (Figures 6C and 6E). These CD56$^+$ T cells were predominantly CD8$^+$ (Figure S5), suggesting that extended expansion may promote NK-like features in a subset of CD8$^+$ T cells. Upon the 3 h co-culture, NK-like T cell frequencies remained absent in day 5 CAR$^+$ cells and were higher but stable on day 10. A small but statistically significant increase was observed in CAR$^-$CD56$^+$ T cells on day 10 ($p < 0.05$; Figure 6D). Throughout the restimulation, NK-like T cell frequencies remained stable in both day 5 and day 10 CAR$^+$ T cells (Figures 6F and S2F).

Cryopreservation shows minimal impact on CAR T cell phenotype and functionality

To assess the impact of cryopreservation on CAR T cell function and phenotype, we analyzed day 10 CAR T cell products from three healthy donors under fresh and cryopreserved conditions. To simulate clinical practice, cells were rested for 3 hours post-thaw before evaluation at baseline (0 h) and after co-culture (Figures 7A–7F).[23] Most markers in the 36-parameter panel were comparable between fresh and frozen samples (Figures 7, S6, and S7). Cryopreserved cells consistently exhibited a modest reduction in cytolytic activity

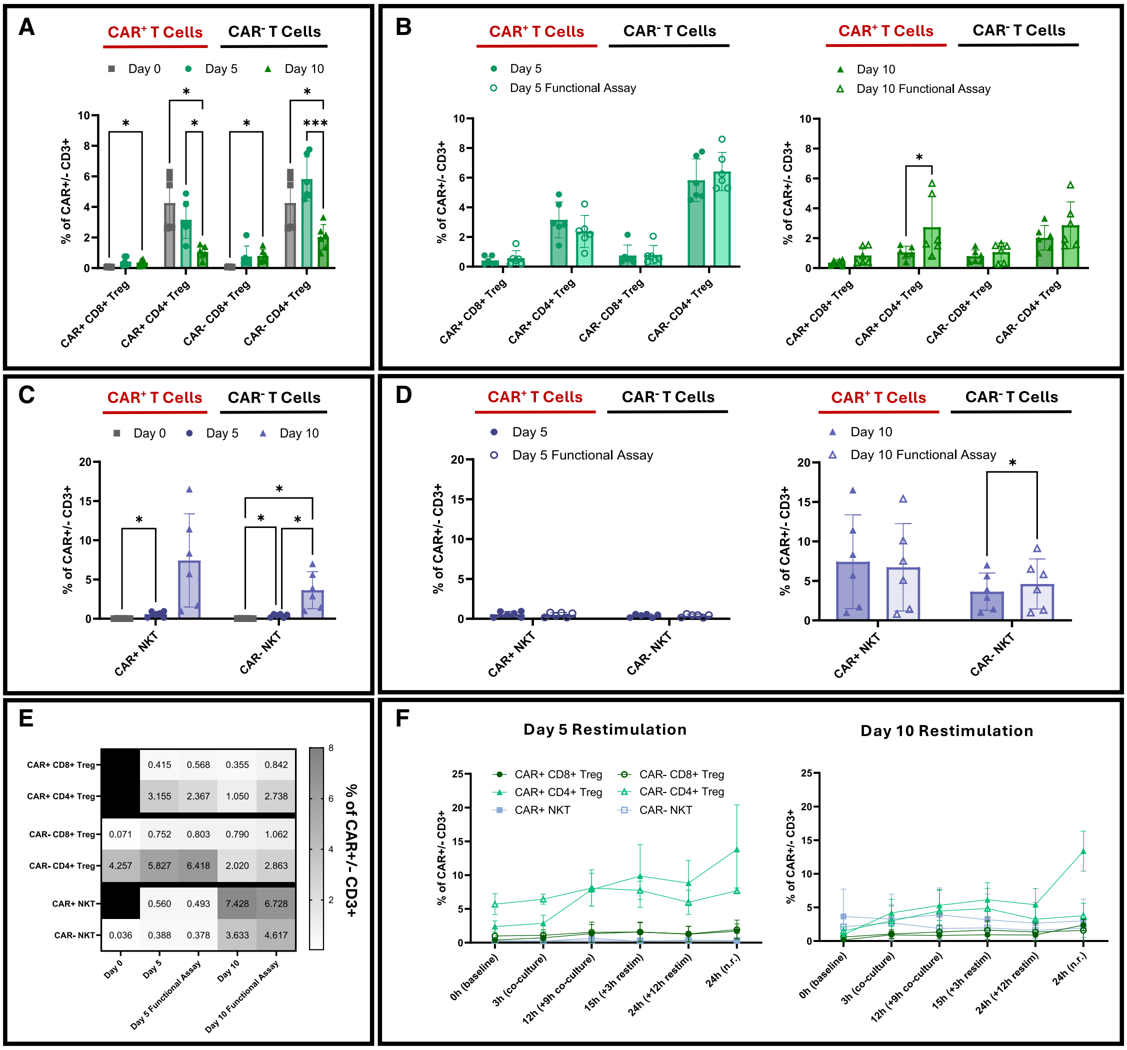

Figure 6. Frequencies and dynamics of Tregs and NK-like T cell subsets

(A) Frequencies of $CD4^+$ and $CD8^+$ $CD25^+FoxP3^+$ Treg in CAR^+ and CAR^- T cells across days 0, 5, and 10 ($n = 6$ donors, 2 independent experiments). Day 0 CAR^- samples served as the baseline reference. (B) Treg frequencies following 3 h co-culture with $CD19^+$ Nalm6-YFP^+ target and $CD19^-$ Nalm6-GFP^+ control cells on days 5 (left) and 10 (right) post-transduction ($n = 6$ donors, 2 independent experiments). (C) Frequencies of NK-like T cells in CAR^+ and CAR^- T cells across days 0, 5, and 10 ($n = 6$ donors, 2 independent experiments). Day 0 CAR^- samples served as the baseline reference. (D) NK-like T cell frequencies following 3 h co-culture on days 5 (left) and 10 (right) post-transduction ($n = 6$ donors, 2 independent experiments). (E) Heatmap summarizing Treg and NK-like T cell subset frequencies across manufacturing time points and co-culture conditions ($n = 6$ donors, 2 independent experiments). (F) Frequencies of $CD4^+$ and $CD8^+$ Tregs and NK-like T cell subsets during 24 h restimulation in CAR^+ and CAR^- T cells (0, 3, 12, 15, and 24 h; $n = 3$ donors). n.r., non-restimulated; restim, restimulated. Statistical analyses: two-way repeated measures ANOVA with Tukey's (A, C and F), and Šídák's (B and D) multiple comparisons test. Data shown as mean ± s.d.; $^*p < 0.05$; $^{***}p < 0.001$. The absence of p-values denotes non-significance at the $p = 0.05$ threshold. All p-values for panel F are provided in Table S7.

shortly after thawing, although this was not statistically significant (Figure 7A). Compared to fresh products, frozen CAR^+ T cells showed reduced Th1 and Tc1 frequencies and increased $CD4^+$ AN cells (Th1: $p < 0.0001$; Tc1: $p < 0.01$; AN: $p < 0.0001$; Figure 7B). Memory phenotypes were minimally affected in frozen products, with a reduction in stem cell memory subsets and enrichment of terminal effector populations (Figure 7C). Cryopreservation further led to modest increases in CD69, HLA-II, and TIM3 expression and a reduction in GLUT1, while levels of exhaustion, apoptotic, senescence, and cytolytic markers remained overall stable (Figures 7D–7F). These differences largely persisted following 3 h co-culture (Figures 7B–7F), with continued reductions in Th1 ($p < 0.05$) and Tc1 ($p < 0.001$) frequencies, and a notable increase in Th9 cells in frozen samples (Figure 7B). Together, these results indicate that while cryopreservation alters some phenotypic features, the overall marker profiles remain broadly comparable.

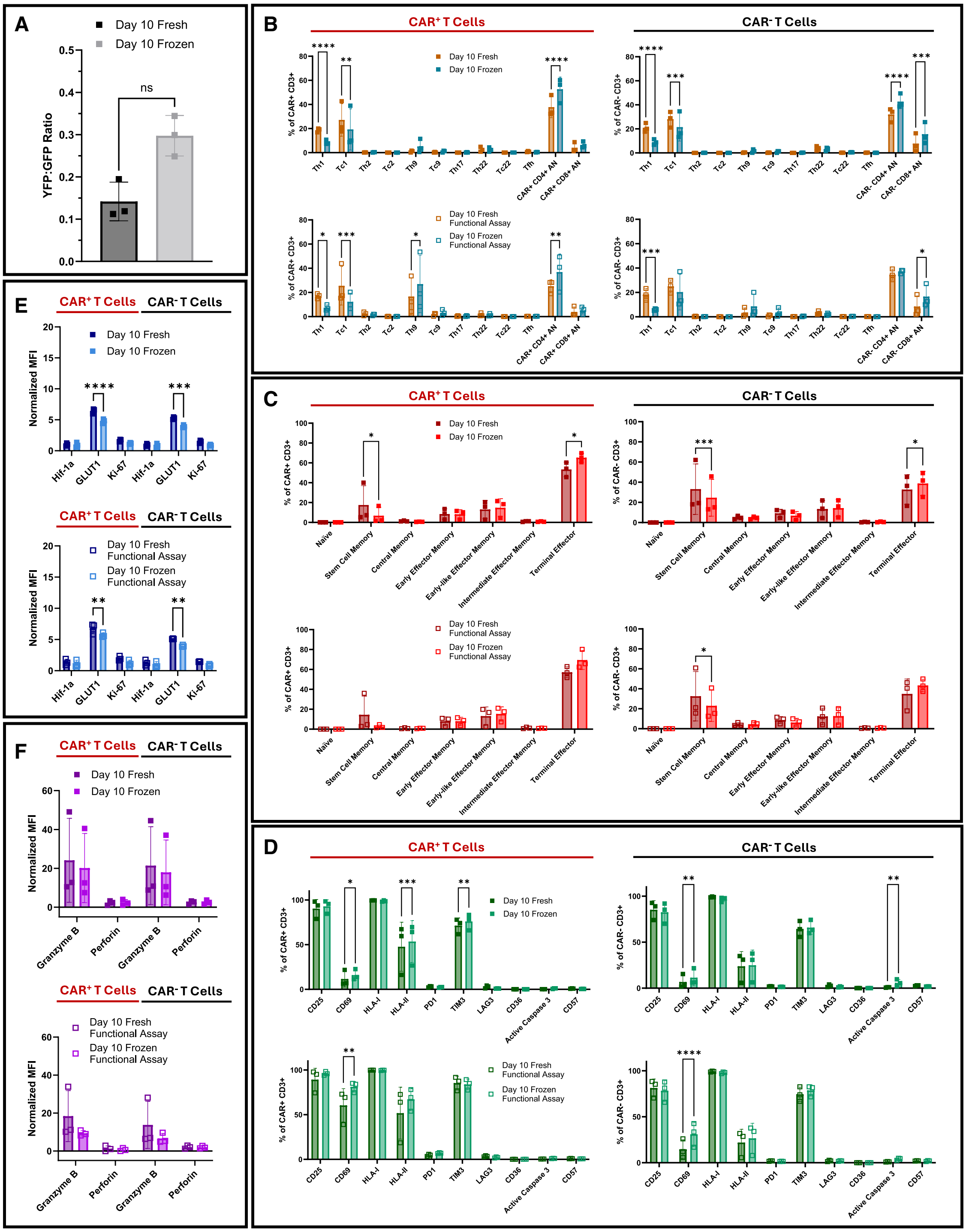

Figure 7. Cryopreservation effects on CAR T cell phenotype and function

(A) Cytotoxicity of day 10 fresh vs. cryopreserved cells quantified by YFP:GFP ratio in 3 h co-culture assays with CD19+ Nalm6-YFP+ target and CD19− Nalm6-GFP+ control cells (*n* = 3 donors). (B) Subset frequencies of Th1, Tc1, Th2, Tc2, Th9, Tc9, Th17, Th22, Tc22, Tfh, and uncommitted (AN) CD4+ and CD8+ cells in CAR+ and CAR− compartments at baseline (0 h) and after 3 h co-culture (*n* = 3 donors). Only subsets representing ≥0.5% of total CD3+ T cells for any time point or donor are shown.

(legend continued on next page)

High-dimensional analyses reveal distinct marker combinations

Sequential gating effectively identifies pre-defined populations but can miss marker relationships outside conventional gates. To address this, we performed Uniform Manifold Approximation and Projection (UMAP) analysis of CD45⁺ gated cells at days 0, 5, and 10. Day 0 cells clustered tightly with uniformly low expression of activation, metabolic, and cytotoxic markers, consistent with a resting, pre-engineered phenotype. By day 5, cells shifted into distinct UMAP regions characterized by co-expression of memory and metabolic markers (CCR7, CD45RA, GLUT1), proliferative and activation signals (Ki-67, CD25), and cytotoxic effectors (granzyme B). In contrast, day 10 cells populated new clusters defined by CD8 dominance, HLA class II, TIM3, and CD45RA expression with reduced CCR7, indicating terminal differentiation (Figure S8). This was accompanied by a shift from a Tc1-, Th9- and Th1-enriched T_{SCM} memory repertoire at day 5 to predominantly Tc1 subsets at day 10, consistent with selective expansion of the cytotoxic lineage observed during CAR T cell manufacturing (Figures 2F, 2H and S9). The 3 h co-culture induced rapid CD69 upregulation at both timepoints, with minimal changes in UMAP distribution, suggesting preserved phenotypic organization despite antigen exposure (Figure S10).

To further reveal unrecognized marker relationships, combinatorial gating partitioned the $CD3^+CAR^+$ populations into sub-populations defined by specific marker co-expression patterns (Figure 8). Day 5 products were dominated by $CD4^+$ Th1 cells co-expressing Ki-67 and IRF4, whereas day 10 products exhibited a more heterogeneous mix of Th1 and Tc1 subsets, likely driven by reduced CD4 proliferation (Figure 8A). A $CCR7^+CD45RA^+$ T_{SCM}-like population enriched at day 5 co-expressed CD25, GLUT1, Ki-67, and IRF4, consistent with a proliferative and metabolically active state. By contrast, day 10 cells shifted toward a $CCR7^-CD45RA^+$ effector phenotype with variable Ki-67 expression, suggesting divergent proliferative capacity (Figure 8B). A highly proliferative ($Ki\text{-}67^+$) and functional ($CD25^+$granzyme B^+) cell subset was prevalent in day 5 cultures upon antigen exposure. In contrast, day 10 cells lacked this distinct population and displayed more variable Ki-67 expression (Figure 8C). Day 5 T_{TE} ($CD45RA^+CCR7^-$) and T_{SCM}-like ($CD45RA^+CCR7^+$ $CD95^+$) subsets showed distinct CD69 and PD1 expression dynamics during antigen exposure and rechallenge. By comparison, day 10 cells exhibited a more uniform phenotype, dominated by $CCR7^-$ $CD45RA^+TIM3^+PD1^-$ populations (Figure 8D). This analysis supports the activation and exhaustion dynamics initially observed in Figure 4. Metabolic remodeling was also evident, as day 5 cells maintained GLUT1 along with co-stimulatory markers CD27 and CD28. Day 10 products showed a reduced proportion of $CD27^+CD28^+$ double-positive cells, a phenotype associated with limited persistence (Figure 8E). Overall, this combinatorial approach has the ability to resolve molecular diversity within defined subsets, offering mechanistic insights into CAR T cell behavior not usually captured by conventional gating.

DISCUSSION

In this study, we developed and applied a high-dimensional spectral flow cytometry panel to map the phenotypic and functional trajectories of CAR T cells throughout manufacturing. Our analysis revealed distinct cellular states associated with mid (day 5) and late (day 10) expansion time points. By day 5, CAR T cells exhibited a favorable profile marked by $CD4^+$ Th1 predominance, T_{SCM} enrichment, elevated glycolytic activity (GLUT1), and high proliferative capacity (Ki-67). This profile was associated with robust expansion and strong killing upon antigen exposure. In contrast, day 10 cells displayed a more terminally differentiated phenotype, dominated by $CD8^+$ Tc1 subsets, reduced stem cell memory subsets, and increased $CD56^+$ NK-like populations. While both products retained potent killing capacity against the $CD19^+$ Nalm6 model, the day 5 phenotype is more consistent with features linked to sustained *in vivo* efficacy.[22,24–26] This trajectory reflects a manufacturing trade-off, where extended expansion increases yield but depletes T_{SCM} and T_{CM} subsets linked to clinical benefits. Meanwhile, it enriches for differentiated T_{TE} populations that may limit durability.[13,16,27,28] In line with studies showing that brief *ex vivo* expansion (1–3 days) preserves memory traits and enhances engraftment,[24,29] our findings suggest that harvesting CAR T cells around day 5 may achieve a more desirable balance between yields and sustained therapeutic potential.

During CAR T cell manufacturing, the emergence of minor yet functionally significant subsets may contribute to disparate clinical outcomes. By day 10, we observed a notable increase in $CD3^+CD56^+$ NK-like cells within the CAR^+ fraction, particularly among $CD8^+$ T cells. These subsets were absent at days 0 and 5, suggesting their development is associated with prolonged culture time. CD56 expression, traditionally a marker for NK cells, has been identified on activated T cells and is linked to enhanced cytotoxic potential.[30] However, recent evidence indicates that sustained stimulation can drive $CD8^+$ T cells to adopt NK-like features, with a shift toward functional impairment in CAR T cell models.[31] Our findings suggest that extended culture alone may promote the emergence of these phenotypes, even without continuous antigen exposure. Monitoring $CD3^+CD56^+$ subsets may serve as a useful quality control measure to avoid enriching exhausted or dysregulated cells. Overall,

(C) Differentiation state distributions (T_N, T_{SCM}, T_{CM}, T_{EM}, and T_{TE}) in CAR^+ and CAR^- $CD3^+$ T cells at baseline (0 h) and after 3h co-culture (n = 3 donors). (D) Frequencies of CD25, CD69, HLA class I, HLA class II, PD1, TIM3, LAG3, CD36, active caspase 3, and CD57 in CAR^+ and CAR^- $CD3^+$ T cells at baseline (0 h) and after 3 h co-culture (n = 3 donors). (E) Normalized MFI of Hif-1a, GLUT1, and Ki-67 in CAR^+ and CAR^- T cells at baseline (0 h) and after 3 h co-culture (n = 3 donors). (F) Normalized granzyme B and perforin levels in CAR^+ and CAR^- T cells at baseline (0 h) and after 3 h co-culture (n = 3 donors). All MFIs were normalized to the average of day 0 MFI of the respective marker. Statistical tests: two-tailed Mann-Whitney U test (A); two-way repeated measures ANOVA with Šídák's multiple comparisons test (B–F). Data shown as mean ± SD; *$p < 0.05$; **$p < 0.01$; ***$p < 0.001$; ****$p < 0.0001$. ns, not significant. The absence of p-values denotes non-significance at the p = 0.05 threshold.

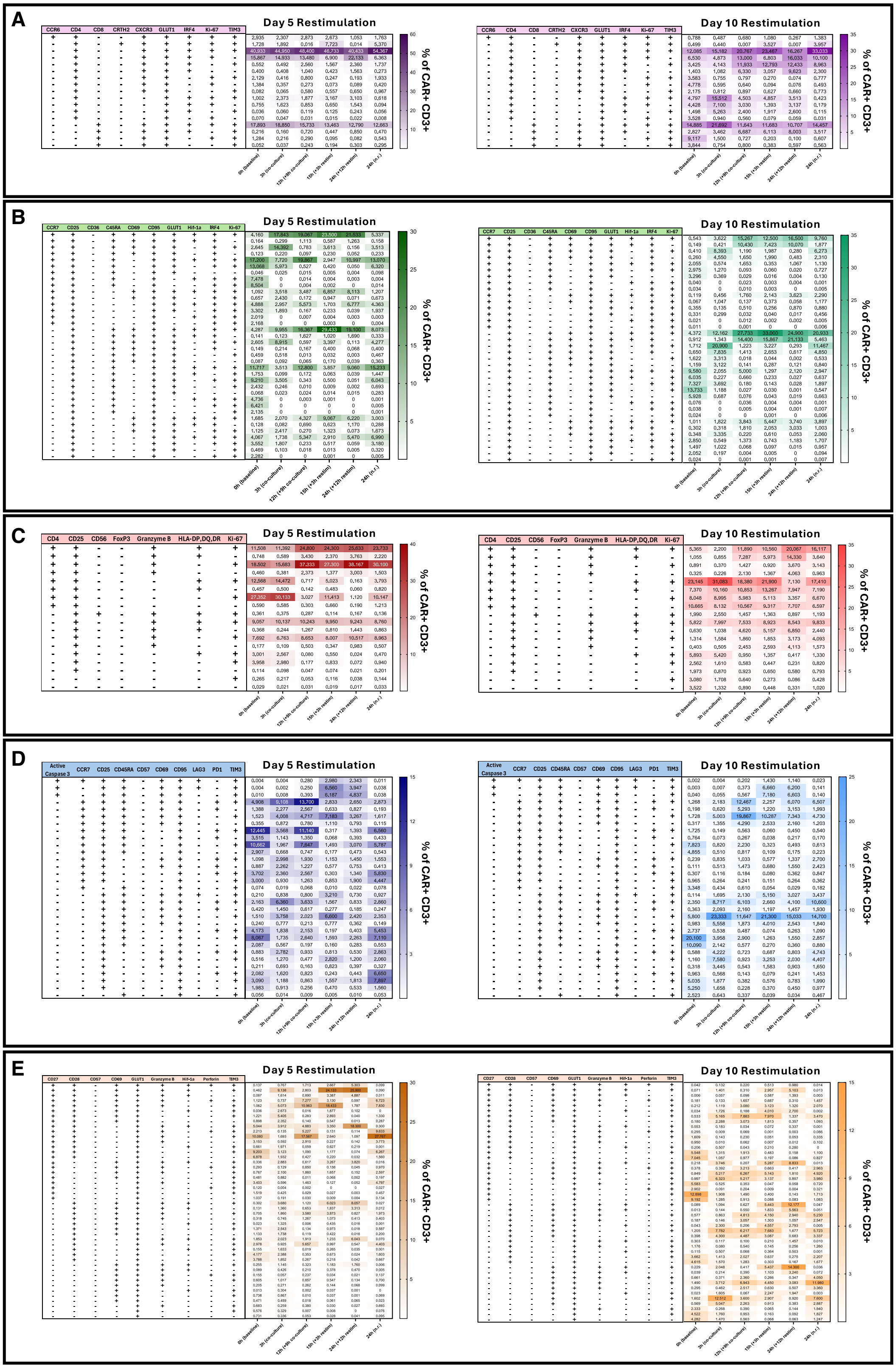

(legend on next page)

this demonstrates the panel's utility in detecting subtle phenotypic changes during manufacturing.

Integrating proliferation and metabolic markers into our analysis revealed distinct mechanisms driving the shift from Th1 to Tc1 dominance during CAR T cell expansion. This transition reflects reduced CAR^-CD4^+ and expanded $CD8^+$ subsets within the CAR^+ fraction. CAR^+CD8^+ T cells expressed high levels of glucose transporter GLUT1, suggesting a selective proliferative and metabolic advantage. In contrast, $CD4^+CAR^-$ T cells showed lower proliferation rates, potentially contributing to their relative decline by day 10. This shift in the CD4:CD8 ratio is clinically relevant, as CAR T products with defined CD4:CD8 ratios have been associated with improved clinical outcomes.[32–34] Incorporating metabolic markers such as GLUT1 provides insight into the energetic state of CAR T cells during expansion.[35,36] Peak GLUT1 and Ki-67 expression at day 5 indicate a metabolically and proliferatively active state. By day 10, these markers decline yet remain elevated compared to day 0, suggesting expanded cells maintain metabolic and cytotoxic potential despite reduced proliferation. Understanding these phenotypic shifts during manufacturing informs strategies to preserve metabolic fitness, delay exhaustion, and enhance the durability and efficacy of CAR T cell therapies.[35,36]

During manufacturing, CAR T cells exhibited distinct checkpoint and activation marker dynamics. CD25 remained constitutively high from day 5 onward, reflecting sustained activation, whereas CD69 expression was transient and context dependent, increasing during antigen exposure and rechallenge assays. Day 5 CAR^+ cells rapidly upregulated checkpoint receptors PD1 and LAG3 upon antigen stimulation, while day 10 cells displayed delayed kinetics. TIM3 levels were elevated from day 5 through day 10, indicating early and sustained expression. Despite the presence of exhaustion-associated markers, apoptotic indicators such as active caspase 3 remained low, showing no evidence of activation-induced cell death. These findings suggest that exhaustion phenotypes dynamically emerge during manufacturing and antigen exposure but do not necessarily result in immediate functional impairment. Importantly, *in vitro* expression of inhibitory receptors does not directly predict clinical exhaustion. *In vivo*, CAR T cells encounter sustained antigen stimulation and immunosuppressive pressures from the tumor microenvironment, conditions that promote functional exhaustion and limit persistence.[18,37–40] Therefore, *in vitro* exhaustion profiling must be interpreted within the broader clinical context. To bridge this gap, this panel can be used to correlate manufacturing phenotypes with post-infusion outcomes and identify biomarkers predictive of CAR T cell efficacy.

Comparative profiling of CAR^+ and CAR^- T cells revealed parallel phenotypic trajectories, characterized by increased activation markers, shared differentiation patterns, and dynamic checkpoint receptor expression. However, CAR^+ cells exhibited greater metabolic activation, likely due to CAR-mediated signaling or a selective growth advantage. The observation that both CAR^+ and CAR^- cells undergo similar phenotypic progression suggests that expansion conditions may have a greater impact on product identity than the specific gene transfer method used. As such, this assay platform can be applied to diverse gene editing systems, including CRISPR, base editing, and transposons to evaluate phenotypic outcomes and disentangle the contributions of specific engineering platforms.

Cryopreservation remains central to CAR T cell manufacturing and clinical logistics, particularly as therapy scales globally. Our findings show that core phenotypic and functional attributes are largely preserved following freeze-thaw cycle. Cryopreserved day 10 products maintained comparable phenotypic profiles to fresh samples, with slight reductions in Th1 or Tc1 frequencies and modest changes in CD69, HLA-DP, DQ, DR, TIM3, or GLUT1 levels. Antigen responsiveness was largely preserved following cryopreservation. Clinically, these findings are reassuring and align with data showing comparable efficacy between fresh and cryopreserved CAR T cell products.[23,41,42]

Another notable finding is the presence of CAR^+ Tregs within the product, which could have a dual effect. While they may exert immune-regulatory functions by dampening excessive inflammatory responses,[43] they could also suppress anti-tumor activity and limit therapeutic efficacy.[19] Additionally, HLA class II upregulation observed in CAR^+ cells poses a known risk to allogeneic use, typically mitigated through targeted genome editing.[44] Together, this study demonstrates the value of dynamic phenotypic profiling to capture shifts in CAR T cell state during production. By integrating differentiation, metabolic, activation, and functional attributes, this approach offers the potential to uncover mechanistic insights into how manufacturing conditions influence product features associated with clinical performance.

While our study provides detailed insights into CAR T cell manufacturing dynamics, it also lays the groundwork for broader application. Although *in vitro* co-culture assays cannot fully capture

Figure 8. High-dimensional combinatorial analysis of CAR T cell phenotypes

Combinatorial co-expression analysis of $CD3^+CAR^+$ T cells at day 5 (left) and day 10 (right) after restimulation, grouped by functional modules. Frequencies shown represent percentage of total CAR^+CD3^+ cells; only marker combinations with >5% expression in at least one donor or time point are shown. Data from $n = 6$ donors for 0–3 h, and $n = 3$ donors for 12–24 h. (A) Helper and cytotoxic polarization with exhaustion markers (CCR6, CD4, CD8, CRTH2, CXCR3, GLUT1, IRF4, Ki-67, TIM3). (B) Memory, proliferation, and metabolic markers (CCR7, CD25, CD36, CD45RA, CD69, CD95, GLUT1, Hif-1a, IRF4, and Ki-67). (C) Treg, NK-like T cell, proliferation, functional, and HLA class II markers (CD4, CD25, CD56, FoxP3, granzyme B, HLA-DP, DQ, DR, Ki-67). (D) Activation, stemness, apoptotic, senescence, and exhaustion markers (active caspase 3, CCR7, CD25, CD45RA, CD57, CD69, CD95, LAG3, PD1, TIM3). (E) Cytotoxic and metabolic effector markers (CD27, CD28, CD57, CD69, GLUT1, granzyme B, Hif-1a, perforin, TIM3).

the complexity of *in vivo* responses, this framework can be extended to patient-derived products to help link cellular phenotypes with clinical outcomes. The spectral panel itself is platform-agnostic and adaptable to diverse manufacturing protocols, enabling comparative profiling across workflows. Moreover, the panel's modular design allows marker substitution, as demonstrated by replacing GLUT1 with GAPDH, and can be extended to include mitochondrial or fatty acid metabolism to complement glycolytic readouts. This versatility makes the panel highly adaptable across diverse cell types and diseases. Building on this flexibility, we identified a focused set of markers suitable for in-process and release testing to support clinical manufacturing (Table S2).

Conclusion

This study presents a novel high-dimensional framework for profiling CAR T cells throughout manufacturing, capturing key attributes that drive their behavior and function. Among the many insights revealed by this dataset, we show that harvest timing defines which cellular states are captured in the final drug product. Mid-harvest products consistently exhibit memory-enriched, metabolically active phenotypes, while later-harvested cells display terminal differentiation yet retain functional capacity. These findings establish a foundation for identifying previously unrecognized critical quality attributes that may influence CAR T cell product performance. By characterizing the impact of manufacturing variables, this platform can help align product features with specific therapeutic goals. Looking ahead, the application of this panel in clinical settings may uncover predictive biomarkers that link product phenotypes to patient outcomes, guiding the next generation of cell therapy design.

MATERIALS AND METHODS

Spectral flow cytometry panel development

Antibody titration

Antibodies were titrated on their respective positive cell populations (Table S3) at serial dilutions, and optimal concentrations were determined based on calculated stain indices and resolution between the negative and positive populations. Each titration was performed in technical duplicate using 5 serial dilutions in the appropriate staining buffer (surface antibodies in BSA stain buffer [BD Biosciences, catalog no. 554657]; intracellular antibodies in 1× permeabilization buffer prepared from 10× stock [eBioscience, catalog no. 00-8333-56]). After staining, cells were fixed in 0.5% formaldehyde (200 μL) (Thermo Scientific, catalog no. 28908) and stored at 4°C in the dark until acquisition.

Sample preparation and multicolor staining

During panel development, different sample sources were used to ensure broad marker coverage. For initial antibody titrations, purified cell subsets known to express the target markers were chosen (Table S3). Once optimal antibody concentrations were established, a composite sample was generated by pooling peripheral blood mononuclear cells (PBMCs), gene-edited CAR T cells, non-edited T cells, and activated T cells in varying ratios to ensure comprehensive marker representation. Nalm6 GFP and YFP cells were further spiked at different ratios. All samples (including single cell types and mixed populations) were processed using the multicolor staining protocol in the supplemental methods. Before staining, all antibody stock solutions were centrifuged at 10,000 × g for 5 min at 4°C to pellet any antibody aggregates. Cells were stained according to an optimized protocol consisting of four extracellular steps: Fc block, CAR detection, pre-stain, and extracellular staining, followed by an intracellular staining step after fixation and permeabilization. Master mixes were prepared for each step (see Table S4 for composition).

Staining was performed in 5-mL polystyrene fluorescence-activated cell sorting (FACS) tubes with volumes as indicated. If starting from cryopreserved PBMCs, cells were thawed according to standard protocols and allowed to rest for ≥8 h in culture medium before staining to restore surface epitopes. Samples were resuspended in DPBS (Corning, catalog no. 21-031-CV) for final acquisition.

Fluorescence controls

For each fluorochrome, single-color (SC) controls and fluorescence minus one (FMO) controls were included, along with unstained controls (Figure S11). All control samples were handled in parallel with fully stained samples, undergoing the same steps but omitting or substituting specific reagents as appropriate. Refer to Table S4 for further details on the antibody master mix and prepared buffers.

Reference controls and spectral unmixing

For spectral unmixing, both cell-based and bead-based SC reference controls were prepared (Table S5). UltraComp eBeads (Invitrogen, catalog no. 01-3333-42) or Cytek FSP CompBeads (Cytek Biosciences, catalog no. B7-10011) were used for fluorescent antibody capture when cell controls were unavailable due to low marker expression. Single-color reference samples using beads were prepared by mixing one drop of beads with the applicable antibody and stain buffer at its respective staining step. Notably, SC bead samples were only included in the relevant portions of the protocol (e.g., an intracellular marker control was not exposed to surface staining steps prior to fixation).

Spectral unmixing was performed using SpectroFlo software v3.3.0 (Cytek Biosciences). Reference controls were selected to meet strict quality criteria: each SC control was at least as bright as the corresponding signal in a fully stained sample and unmixed against a corresponding unstained bead control. For unmixing, at least 100 bright events were collected from each SC reference, ensuring a clear full-spectrum signature for each fluorochrome. Full-spectrum and normalized full-spectrum signatures were assessed for each reference control, and only controls that generated a matching spectral signature to the CytekCloud database were used (Cytek Biosciences). Specifically, controls that showed baseline expression in unexpected detectors were rejected. Using these criteria, a library of optimal reference controls (both cell-based and bead-based) was established.

Live unmixing was then applied, and results were checked by inspecting the N × N spectral matrix of all parameters in fully stained samples. Population distributions in each channel were examined for any

anomalies inconsistent with expected biology (e.g. distortions or shifts due to unmixing errors). When necessary, the unmixing was refined in a stepwise manner: (1) adjusting the gating of the reference control to capture a pure population, (2) substituting a dim reference control with a brighter one, (3) using an "internal negative" cell population (cells within the sample that lack expression of the marker) in place of the universal negative for a given marker, or (4) swapping a bead-based reference for a cell-based reference (or vice versa). Any residual spillover/unmixing error remaining after these adjustments was quantified and applied using SpectroFlo's Adjust Spillover tool. These values are reported in Figure S12, with representative unmixing errors and corrections shown in Figure S13.

Instrument setup and data acquisition

Flow cytometry data were acquired on a Cytek Aurora spectral cytometer equipped with four lasers (405 nm violet, 488 nm blue, 561 nm yellow-green, 640 nm red), using the configuration detailed in Figure S14. The instrument was allowed to warm up for 30 min, and standard daily QC was performed with SpectroFlo QC beads (Cytek, catalog no. B7-10001) before acquiring samples. Cytometer settings followed the Cytek-recommended assay settings. Antibody staining concentrations were optimized to yield in-scale signals for all channels, with particularly bright markers prone to fluorescence spread intentionally used at lower saturating concentrations to minimize spillover spreading. Sample tubes (200 μL volume) were gently vortexed immediately before acquisition to disrupt any cell aggregates. Unless stated otherwise, ~200 μL of each sample was acquired per run at a high flow rate (~80 μL/min).

Gating strategy

To ensure robust identification of cell populations in high-dimensional data, the gating strategy was carefully developed and validated using appropriate controls. A full set of FMO controls was used to inform gate thresholding for future experiments (Figure S11 provides a detailed overview of all FMO results). These FMO controls helped determine which markers should be included in the routine use of the panel. In addition, fluorescence spread was addressed in the gating approach. This two-dimensional gating strategy reduced the impact of spread on the discrimination of positive vs. negative populations. All gating was performed on biologically relevant parent populations (e.g., live singlet lymphocytes, $CD3^+$ T cells), and gating hierarchy and logic were kept consistent across all samples (Figure 1). Figure S15 illustrates enhanced marker resolution achieved through a representative manual gating strategy.

Data extraction

Subset frequencies were reported as percentages of the immediate parent population (e.g., $CD3^+CAR^+$), allowing for direct comparison of phenotypic distributions over time. For some intracellular markers (GLUT1, GAPDH, granzyme B, perforin, Hif-1a), MFI was extracted from the entire parent population and normalized to the average day 0 MFI across donors. This approach enabled accurate fold-change quantification over the course of manufacturing and stimulation. Ki-67 was analyzed separately due to its negative baseline expression. Using day 0 negative signal intensities for normalization may complicate interpretation of Ki-67 expression. To enable accurate comparison of proliferative activity across timepoints, MFI values were calculated exclusively from the $Ki\text{-}67^+$ gated population (Figure S16).

Cell culture and functional assays

CAR construct

For all manufacturing runs, we employed a second-generation CAR targeting human CD19. The construct consisted of an FMC63-derived single-chain variable fragment (scFv) for antigen recognition, joined to a human CD8α hinge and transmembrane domain. Intracellular signaling was mediated through a 4-1BB (CD137) co-stimulatory domain followed by CD3ζ (Figure S17A).

CAR T cell production

Human $CD3^+$ T cells were obtained from healthy donor PBMCs or purchased from Excellos (San Diego, CA). All biological materials were used under the oversight of the Institutional Biosafety Committee at the University of Southern California (BUA No. 22-00032). For lentiviral CAR T cell generation, T cells were activated with Human T Cell TransAct (Miltenyi Biotec, catalog no. 130-128-758) at a 1:100 dilution and simultaneously transduced with a second-generation anti-CD19 CAR lentiviral vector (Vector BioMed, Anti-CD19 CAR LV) at a multiplicity of infection (MOI) of 20. Transductions were carried out in G-Rex 6M (Wilson Wolf, catalog no. 80660M; 10 cm^2 gas-permeable culture area per well) to support high-density cell growth. Three days after transduction, fresh culture medium was added to fill the G-Rex well to its maximum volume. On day 5 post-transduction, an in-process sample was harvested for analysis, and the remaining cells were re-seeded into new G-Rex 6M vessels to continue expansion until day 10. All T cell cultures were maintained in complete T cell media consisting of TexMACS medium (Miltenyi Biotec, catalog no. 170-076-306), supplemented with 3% human AB serum (BioIVT, catalog no. HUMANABSRMP-HI-1), 12.5 ng/mL recombinant human interleukin-7 (IL-7) (Bio-Techne, catalog no. BT-007-AFL-025), and 12.5 ng/mL recombinant human IL-15 (Bio-Techne, catalog no. BT-015-AFL-025). Cultures were incubated at 37°C in a humidified 5% CO_2 atmosphere. This complete medium was used for all T cell expansion steps unless otherwise noted. Initial panel development and antibody optimization were performed using non-viral CAR T cells generated by electroporation of DNA encoding the CD19-targeting CAR construct, as previously described.[45] These early experiments facilitated the refinement of antibody titration, staining protocols, and gating strategies. For all subsequent manufacturing runs and phenotypic analyses, lentivirally transduced CAR T cells were used.

Cell lines

The Nalm6 acute lymphoblastic leukemia cell line ($CD19^+$) and a derivative Nalm6 GFP CD19 knockout line (lacking surface CD19 expression) were provided by the Stanford Laboratory for Cell and Gene Medicine. An additional Nalm6 variant expressing YFP (Nalm6 YFP) was generated in-house by transducing Nalm6 cells

with a lentiviral EF1α-eYFP (Topaz) vector (BPS Bioscience, catalog no. 79989) at an MOI of 1.25, following the manufacturer's protocol. On day 5 post-transduction, YFP^+ cells were enriched by FACS (BD FACSAria II), and purity was subsequently confirmed by flow cytometry. All cell lines were cultured in Advanced RPMI 1640 medium (Gibco, catalog no. 12633020), supplemented with 10% fetal bovine serum (Corning, catalog no. 35-015-CV), and 1% GlutaMAX (Gibco, catalog no. 35050-061). Cell cultures were maintained at 37°C with 5% CO_2.

Functional cytotoxicity assay

A standardized effector-target co-culture assay was used to evaluate CAR T cell cytotoxicity in tandem with phenotypic profiling. Viral CD19 CAR T cells at two expansion time points (day 5 and day 10 post-transduction) were cryopreserved in CryoStor CS10 (STEMCELL Technologies, catalog no. 07930) and stored in vapor-phase liquid nitrogen. For the assay, frozen cells were thawed, washed, resuspended in complete T cell media (TexMACS + 3% hAB serum + 12.5 ng/mL IL-7/IL-15), and rested for at least 3 h prior to co-culture. CAR T cells were then mixed with target and control tumor cells at a 2:1:1 ratio: 5E5 CAR T cells with 2.5E5 $CD19^+$ Nalm6-YFP^+ target cells and 2.5E5 $CD19^-$ Nalm6-GFP^+ control cells in 24-well plates. Plates were briefly centrifuged (100 × *g*, 3 min, room temperature) to promote cell-to-cell contact and incubated for 3 h at 37°C and 5% CO_2. Following co-culture, cells were pooled per donor, stained with the full 36-marker spectral flow cytometry panel, and analyzed in a single-tube workflow. Antigen-specific killing was quantified as the ratio of viable YFP^+ to GFP^+ cells (viable $CD19^+YFP^+$)/(viable $CD19^-GFP^+$), with a reduction in this ratio indicating selective killing of $CD19^+$ targets (Figure S17B). Non-transduced T cells were included as negative controls in three of six donors to confirm CAR-dependent target elimination.

Restimulation assay

To evaluate the durability and dynamics of CAR T cell responses following repeated antigen exposure, a restimulation assay was performed using CAR T cells from three donors (Figure 2B). At the 12 h timepoint, $CD19^+$ Nalm6-YFP^+ target cells (effector-to-target ratio 2:1) were added to the culture to simulate a second antigenic encounter. Samples were collected at baseline (0 h), after initial co-culture (3 h), and following extended incubation at 12 h, 15 h (3 h post-restimulation), and 24 h. In parallel, a matched condition without restimulation (24 h non-restimulated) was included to assess phenotype evolution independent of secondary antigen exposure. Cells were analyzed using the 36-marker spectral cytometry panel. Cytotoxic activity was assessed by measuring the frequency of viable YFP^+ cells at each time point, enabling assessment of both initial and sustained target cell elimination.

T cell activation

Resting human T cells (isolated from PBMCs or obtained from Excellos) were activated with Human T cell TransAct (Miltenyi) at a 1:100 dilution. Cells were incubated in a T-25 flask for 24–72 h (37°C, 5% CO_2) to achieve broad activation. These activated T cells were used to validate activation markers in the panel (e.g., CD69 and CD25 upregulation at 48 and 72 h).

UMAP clustering and high-dimensional analysis

High-dimensional visualization and clustering of immune phenotypes were performed using UMAP and Flow Self-Organizing Map (FlowSOM) algorithms implemented within FlowJo (version 10.10.1). For UMAP analysis, 50,000 $CD45^+$ events were randomly subsampled and concatenated per condition. The UMAP plugin (version 4.1.1) was applied using default parameters: Euclidean distance metric, 15 nearest neighbors, minimum distance of 0.5, and 2 output dimensions. All panel markers were included except for viability dye, GFP, and YFP. FlowSOM clustering was performed using the FlowJo FlowSOM plugin (version 4.1.0). SOMs were generated on the same concatenated datasets as were used for UMAP. Analysis parameters included a 10 × 10 grid size, generation of 10 meta-clusters, and default visualization settings (minimum spanning tree layout with pie chart representation of marker expression, hierarchical heatmaps based on both rows and columns, and node scaling set to 100%).

Combinatorial gating analysis

To assess multi-marker co-expression patterns across functional and differentiation axes, combinatorial gating was performed using FlowJo (version 10.10.1). Individual gates were first defined for all markers of interest based on FMO controls and pre-established positive/negative thresholds. Boolean logic was then applied within FlowJo to generate all possible combinations of marker expression across selected functional categories (e.g., activation, memory, exhaustion, metabolic, cytotoxic, lineage). Each Boolean gate reflected a unique combination of marker expression states and was used to quantify the frequency of complex phenotypes across time points and stimulation conditions. Outputs were exported using the FlowJo Table Editor for downstream analysis and graphical representation.

Statistical analysis and data presentation

Spectral unmixing and manual compensation were performed using SpectroFlo software (version 3.3.0, Cytek Biosciences). Gating was done using FlowJo (version 10.10.0). Unless otherwise specified, data are presented as mean ± standard deviation (SD). Differences in T cell subtypes and marker expression across key manufacturing time points (days 0, 5, and 10) and restimulation readouts were evaluated by two-way repeated measures ANOVA with Tukey's multiple comparisons test ($p < 0.05$). CD4/CD8 CAR^+ and CAR^-, fresh versus frozen products, and co-culture assay comparisons for day 5 and day 10 viral CAR T cells were performed using two-way repeated measures ANOVA with Šídák's multiple comparisons test ($p < 0.05$). For comparison of YFP:GFP ratios and relative YFP frequencies, a two-tailed Mann-Whitney *U* test or one-way ANOVA with Šídák's multiple comparisons test was used ($p < 0.05$). Differences in CAR expression and MFI on days 5 and 10 of manufacturing were assessed by paired, two-tailed Student's t test ($p < 0.05$). For all statistical tests, *p*-values are denoted with asterisks as follows: $^*p < 0.05$, $^{**}p < 0.01$,

***$p < 0.001$, and ****$p < 0.0001$. The absence of p-values denotes non-significance at $p = 0.05$ threshold. Schematics and graphs presented in the figures were created using BioRender (www.biorender.com), GraphPad Prism (version 10.2.3), and Microsoft Excel.

DATA AVAILABILITY

The datasets generated and analyzed during this study are available from the corresponding author upon reasonable request. All flow cytometry panel design and optimization details are included in the supplemental information.

ACKNOWLEDGMENTS

M.A. is supported in part by the National Cancer Institute under award no. P30CA014089. The content is solely the responsibility of the authors and does not necessarily reflect the official views of the National Cancer Institute or the National Institutes of Health. The authors acknowledge Mark Edinger and Aric Bitton (Cytek Biosciences) for their support during the design and interpretation of the panel.

AUTHOR CONTRIBUTIONS

A.C.-G., C.L.F., and M.A. conceptualized the study. A.C.-G., C.L.F., A.V., and M.A. designed the experiments. A.C.-G., C.L.F., and A.C. conducted the experiments. All authors analyzed the data. A.C.-G., C.L.F., and M.A. wrote the manuscript. All authors reviewed and approved the final manuscript.

DECLARATION OF INTERESTS

A.C.-G., C.L.F., and M.A. are inventors on a patent application related to the methods described in this study. A.C., E.J., and A.V. declare no competing interests.

SUPPLEMENTAL INFORMATION

Supplemental information can be found online at https://doi.org/10.1016/j.ymthe.2025.04.006.

REFERENCES

1. Maude, S.L., Frey, N., Shaw, P.A., Aplenc, R., Barrett, D.M., Bunin, N.J., Chew, A., Gonzalez, V.E., Zheng, Z., Lacey, S.F., et al. (2014). Chimeric Antigen Receptor T Cells for Sustained Remissions in Leukemia. N. Engl. J. Med. *371*, 1507–1517.
2. Lee, D.W., Kochenderfer, J.N., Stetler-Stevenson, M., Cui, Y.K., Delbrook, C., Feldman, S.A., Fry, T.J., Orentas, R., Sabatino, M., Shah, N.N., et al. (2015). T cells expressing CD19 chimeric antigen receptors for acute lymphoblastic leukaemia in children and young adults: a phase 1 dose-escalation trial. Lancet *385*, 517–528.
3. Maude, S.L., Laetsch, T.W., Buechner, J., Rives, S., Boyer, M., Bittencourt, H., Bader, P., Verneris, M.R., Stefanski, H.E., Myers, G.D., et al. (2018). Tisagenlecleucel in children and young adults with B-cell lymphoblastic leukemia. N. Engl. J. Med. *378*, 439–448. https://doi.org/10.1056/NEJMoa1709866.
4. Neelapu, S.S., Locke, F.L., Bartlett, N.L., Lekakis, L.J., Miklos, D.B., Jacobson, C.A., Braunschweig, I., Oluwole, O.O., Siddiqi, T., Lin, Y., et al. (2017). Axicabtagene Ciloleucel CAR T-Cell Therapy in Refractory Large B-Cell Lymphoma. N. Engl. J. Med. *377*, 2531–2544.
5. Schuster, S.J., Bishop, M.R., Tam, C.S., Waller, E.K., Borchmann, P., McGuirk, J.P., Jäger, U., Jaglowski, S., Andreadis, C., Westin, J.R., et al. (2019). Tisagenlecleucel in Adult Relapsed or Refractory Diffuse Large B-Cell Lymphoma. N. Engl. J. Med. *380*, 45–56.
6. Shah, N.N., and Fry, T.J. (2019). Mechanisms of resistance to CAR T cell therapy. Nat. Rev. Clin. Oncol. *16*, 372–385. https://doi.org/10.1038/s41571-019-0184-6.
7. Weber, E.W., Parker, K.R., Sotillo, E., Lynn, R.C., Anbunathan, H., Lattin, J., Good, Z., Belk, J.A., Daniel, B., Klysz, D., et al. (2021). Transient rest restores functionality in exhausted CAR-T cells through epigenetic remodeling. Science *372*, eaba1786.
8. Abou-el-Enein, M. (2024). The Fate(s) of CAR T-Cell Therapy: Navigating the Risks of CAR+ T-Cell Malignancy. Blood Cancer Discov. *5*, 249–257.
9. Baguet, C., Larghero, J., and Mebarki, M. (2024). Early predictive factors of failure in autologous CAR T-cell manufacturing and/or efficacy in hematologic malignancies. Blood Adv. *8*, 337–342.
10. Abou-El-Enein, M., Elsallab, M., Feldman, S.A., Fesnak, A.D., Heslop, H.E., Marks, P., Till, B.G., Bauer, G., and Savoldo, B. (2021). Scalable Manufacturing of CAR T Cells for Cancer Immunotherapy. Blood Cancer Discov. *2*, 408–422.
11. Maurer, K., Grabski, I.N., Houot, R., Gohil, S.H., Miura, S., Redd, R., Lyu, H., Lu, W., Arihara, Y., Budka, J., et al. (2024). Baseline immune state and T-cell clonal kinetics are associated with durable response to CAR-T therapy in large B-cell lymphoma. Blood *144*, 2490–2502.
12. Amini, L., Silbert, S.K., Maude, S.L., Nastoupil, L.J., Ramos, C.A., Brentjens, R.J., Sauter, C.S., Shah, N.N., and Abou-El-Enein, M. (2022). Preparing for CAR T cell therapy: patient selection, bridging therapies and lymphodepletion. Nat. Rev. Clin. Oncol. *19*, 342–355.
13. Fraietta, J.A., Lacey, S.F., Orlando, E.J., Pruteanu-Malinici, I., Gohil, M., Lundh, S., Boesteanu, A.C., Wang, Y., O'Connor, R.S., Hwang, W.T., et al. (2018). Determinants of response and resistance to CD19 chimeric antigen receptor (CAR) T cell therapy of chronic lymphocytic leukemia. Nat. Med. *24*, 563–571.
14. Monfrini, C., Stella, F., Aragona, V., Magni, M., Ljevar, S., Vella, C., Fardella, E., Chiappella, A., Nanetti, F., Pennisi, M., et al. (2022). Phenotypic Composition of Commercial Anti-CD19 CAR T Cells Affects In Vivo Expansion and Disease Response in Patients with Large B-cell Lymphoma. Clin. Cancer Res. *28*, 3378–3386.
15. Bai, Z., Woodhouse, S., Zhao, Z., Arya, R., Govek, K., Kim, D., Lundh, S., Baysoy, A., Sun, H., Deng, Y., et al. (2022). Single-cell antigen-specific landscape of CAR T infusion product identifies determinants of CD19-positive relapse in patients with ALL. Sci. Adv. *8*, eabj2820.
16. Arcangeli, S., Bove, C., Mezzanotte, C., Camisa, B., Falcone, L., Manfredi, F., Bezzecchi, E., El Khoury, R., Norata, R., Sanvito, F., et al. (2022). CAR T cell manufacturing from naive/stem memory T lymphocytes enhances antitumor responses while curtailing cytokine release syndrome. J. Clin. Invest. *132*, e150807.
17. Larson, S.M., Walthers, C.M., Ji, B., Ghafouri, S.N., Naparstek, J., Trent, J., Chen, J. M., Roshandell, M., Harris, C., Khericha, M., et al. (2023). CD19/CD20 Bispecific Chimeric Antigen Receptor (CAR) in Naive/Memory T Cells for the Treatment of Relapsed or Refractory Non-Hodgkin Lymphoma. Cancer Discov. *13*, 580–597.
18. Ledergor, G., Fan, Z., Wu, K., McCarthy, E., Hyrenius-Wittsten, A., Starzinski, A., Chang, H., Bridge, M., Kwek, S., Cheung, A., et al. (2024). CD4+ CAR T-cell exhaustion associated with early relapse of multiple myeloma after BCMA CAR T-cell therapy. Blood Adv. *8*, 3562–3575.
19. Good, Z., Spiegel, J.Y., Sahaf, B., Malipatlolla, M.B., Ehlinger, Z.J., Kurra, S., Desai, M.H., Reynolds, W.D., Wong Lin, A., Vandris, P., et al. (2022). Post-infusion CAR TReg cells identify patients resistant to CD19-CAR therapy. Nat. Med. *28*, 1860–1871.
20. Reddy, O.L., Stroncek, D.F., and Panch, S.R. (2020). Improving CAR T cell therapy by optimizing critical quality attributes. Semin. Hematol. *57*, 33–38.
21. Dias, J., Cadiñanos-Garai, A., and Roddie, C. (2023). Release Assays and Potency Assays for CAR T-Cell Interventions. In Potency Assays for Advanced Stem Cell Therapy Medicinal Products, pp. 117–137. https://doi.org/10.1007/978-3-031-30040-0_8.
22. Dickinson, M.J., Barba, P., Jäger, U., Shah, N.N., Blaise, D., Briones, J., Shune, L., Boissel, N., Bondanza, A., Mariconti, L., et al. (2023). A Novel Autologous CAR-T Therapy, YTB323, with Preserved T-cell Stemness Shows Enhanced CAR T-cell Efficacy in Preclinical and Early Clinical Development. Cancer Discov. *13*, 1982–1997.
23. Akel, S., Poston, L., Park, J.J., Schoultz, S.B., Alloush, L., Zheng, F., Zhou, S., Lockey, T., Willis, C., DeRenzo, C., and Gottschalk, S. (2023). Preparation of cryopreserved chimeric antigen receptor T cells for the locoreogional delivery to the neural axis. Cytotherapy *25*, 1149–1154.
24. Ghassemi, S., Durgin, J.S., Nunez-Cruz, S., Patel, J., Leferovich, J., Pinzone, M., Shen, F., Cummins, K.D., Plesa, G., Cantu, V.A., et al. (2022). Rapid manufacturing of non-activated potent CAR T cells. Nat. Biomed. Eng. *6*, 118–128.
25. Deng, T., Deng, Y., Tsao, S.T., Xiong, Q., Yao, Y., Liu, C., Gu, M.Y., Huang, F., and Wang, H. (2024). Rapidly-manufactured CD276 CAR-T cells exhibit enhanced persistence and efficacy in pancreatic cancer. J. Transl. Med. *22*, 633.

26. Yang, J., He, J., Zhang, X., Li, J., Wang, Z., Zhang, Y., Qiu, L., Wu, Q., Sun, Z., Ye, X., et al. (2022). Next-day manufacture of a novel anti-CD19 CAR-T therapy for B-cell acute lymphoblastic leukemia: first-in-human clinical study. Blood Cancer J. *12*, 104.

27. Finney, O.C., Brakke, H.M., Rawlings-Rhea, S., Hicks, R., Doolittle, D., Lopez, M., Futrell, R.B., Orentas, R.J., Li, D., Gardner, R.A., and Jensen, M.C. (2019). CD19 CAR T cell product and disease attributes predict leukemia remission durability. J. Clin. Invest. *129*, 2123–2132.

28. Deng, Q., Han, G., Puebla-Osorio, N., Ma, M.C.J., Strati, P., Chasen, B., Dai, E., Dang, M., Jain, N., Yang, H., et al. (2020). Characteristics of anti-CD19 CAR T cell infusion products associated with efficacy and toxicity in patients with large B cell lymphomas. Nat. Med. *26*, 1878–1887.

29. Ghassemi, S., Nunez-Cruz, S., O'Connor, R.S., Fraietta, J.A., Patel, P.R., Scholler, J., Barrett, D.M., Lundh, S.M., Davis, M.M., Bedoya, F., et al. (2018). Reducing Ex Vivo Culture Improves the Antileukemic Activity of Chimeric Antigen Receptor (CAR) T Cells. Cancer Immunol. Res. *6*, 1100–1109.

30. Pievani, A., Borleri, G., Pende, D., Moretta, L., Rambaldi, A., Golay, J., and Introna, M. (2011). Dual-functional capability of CD3+CD56+ CIK cells, a T-cell subset that acquires NK function and retains TCR-mediated specific cytotoxicity. Blood *118*, 3301–3310.

31. Good, C.R., Aznar, M.A., Kuramitsu, S., Samareh, P., Agarwal, S., Donahue, G., Ishiyama, K., Wellhausen, N., Rennels, A.K., Ma, Y., et al. (2021). An NK-like CAR T cell transition in CAR T cell dysfunction. Cell *184*, 6081–6100.e26.

32. Turtle, C.J., Hanafi, L.A., Berger, C., Gooley, T.A., Cherian, S., Hudecek, M., Sommermeyer, D., Melville, K., Pender, B., Budiarto, T.M., et al. (2016). CD19 CAR–T cells of defined CD4+:CD8+ composition in adult B cell ALL patients. J. Clin. Invest. *126*, 2123–2138.

33. Galli, E., Bellesi, S., Pansini, I., Di Cesare, G., Iacovelli, C., Malafronte, R., Maiolo, E., Chiusolo, P., Sica, S., Sorà, F., and Hohaus, S. (2023). The CD4/CD8 ratio of infused CD19-CAR-T is a prognostic factor for efficacy and toxicity. Br. J. Haematol. *203*, 564–570.

34. Sommermeyer, D., Hudecek, M., Kosasih, P.L., Gogishvili, T., Maloney, D.G., Turtle, C.J., and Riddell, S.R. (2016). Chimeric antigen receptor-modified T cells derived from defined CD8+ and CD4+ subsets confer superior antitumor reactivity in vivo. Leukemia *30*, 492–500.

35. Shi, Y., Kotchetkov, I.S., Dobrin, A., Hanina, S.A., Rajasekhar, V.K., Healey, J.H., and Sadelain, M. (2024). GLUT1 overexpression enhances CAR T cell metabolic fitness and anti-tumor efficacy. Mol. Ther. *32*, 2393–2405.

36. Guerrero, J.A., Klysz, D.D., Chen, Y., Malipatlolla, M., Lone, J., Fowler, C., Stuani, L., May, A., Bashti, M., Xu, P., et al. (2024). GLUT1 overexpression in CAR-T cells induces metabolic reprogramming and enhances potency. Nat. Commun. *15*, 8658.

37. Long, A.H., Haso, W.M., Shern, J.F., Wanhainen, K.M., Murgai, M., Ingaramo, M., Smith, J.P., Walker, A.J., Kohler, M.E., Venkateshwara, V.R., et al. (2015). 4-1BB costimulation ameliorates T cell exhaustion induced by tonic signaling of chimeric antigen receptors. Nat. Med. *21*, 581–590.

38. Ouyang, W., Jin, S.W., Xu, N., Liu, W.Y., Zhao, H., Zhang, L., Kang, L., Tao, Y., Liu, Y., Wang, Y., et al. (2024). PD-1 downregulation enhances CAR-T cell antitumor efficiency by preserving a cell memory phenotype and reducing exhaustion. J. Immunother. Cancer *12*, e008429.

39. García-Calderón, C.B., Sierro-Martínez, B., García-Guerrero, E., Sanoja-Flores, L., Muñoz-García, R., Ruiz-Maldonado, V., Jimenez-Leon, M.R., Delgado-Serrano, J., Molinos-Quintana, Á., Guijarro-Albaladejo, B., et al. (2023). Monitoring of kinetics and exhaustion markers of circulating CAR-T cells as early predictive factors in patients with B-cell malignancies. Front. Immunol. *14*, 1152498.

40. Yin, C., Thapa, B., Debes-Marun, C., Breckenridge, Z., Sandhu, I., Khan, F., and Chu, M.P. (2023). CAR T Cell Exhaustion but Not Ex Vivo Cytotoxicity Is Predictive of Patient Clinical Response: An Interim Analysis of ACIT001/EXC002, a Phase Ib/II Trial of Decentralized Production of CAR T Cells for Treatment of Relapsed/Refractory Aggressive NHL and AL. Blood *142*, 2092.

41. Panch, S.R., Srivastava, S.K., Elavia, N., McManus, A., Liu, S., Jin, P., Highfill, S.L., Li, X., Dagur, P., Kochenderfer, J.N., et al. (2019). Effect of Cryopreservation on Autologous Chimeric Antigen Receptor T Cell Characteristics. Mol. Ther. *27*, 1275–1285.

42. Dreyzin, A., Panch, S.R., Shalabi, H., Yates, B., Highfill, S.L., Jin, P., Stroncek, D., and Shah, N.N. (2023). Cryopreserved anti-CD22 and bispecific anti-CD19/22 CAR T cells are as effective as freshly infused cells. Mol. Ther. Methods Clin. Dev. *28*, 51–61.

43. Roemhild, A., Otto, N.M., Moll, G., Abou-El-Enein, M., Kaiser, D., Bold, G., Schachtner, T., Choi, M., Oellinger, R., Landwehr-Kenzel, S., et al. (2020). Regulatory T cells for minimising immune suppression in kidney transplantation: phase I/IIa clinical trial. BMJ *371*, m3734.

44. Kagoya, Y., Guo, T., Yeung, B., Saso, K., Anczurowski, M., Wang, C.H., Murata, K., Sugata, K., Saijo, H., Matsunaga, Y., et al. (2020). Genetic Ablation of HLA Class I, Class II, and the T-cell Receptor Enables Allogeneic T Cells to Be Used for Adoptive T-cell Therapy. Cancer Immunol. Res. *8*, 926–936.

45. Balke-Want, H., Keerthi, V., Gkitsas, N., Mancini, A.G., Kurgan, G.L., Fowler, C., Xu, P., Liu, X., Asano, K., Patel, S., et al. (2023). Homology-independent targeted insertion (HITI) enables guided CAR knock-in and efficient clinical scale CAR-T cell manufacturing. Mol. Cancer *22*, 100.

YMTHE, Volume *33*

Supplemental Information

High-dimensional temporal mapping of CAR T cells reveals phenotypic and functional remodeling during manufacturing

Amaia Cadinanos-Garai, Christian L. Flugel, Anson Cheung, Enzi Jiang, Alix Vaissié, and Mohamed Abou-el-Enein

Supplemental Methods: Design and Optimization of a 36-Marker Spectral Flow Cytometry Panel for CAR T Cell Profiling

T Cell Subset Phenotyping

To enable comprehensive profiling of T cell lineage and polarization states, the panel was configured to distinguish $CD4^+$ helper and $CD8^+$ cytotoxic T cells along with their specialized subsets.[1,2] Chemokine receptors and transcription factors were included to delineate canonical T helper lineages (Th1, Th2, Th9, Th17, Th22, and Tfh) and their cytotoxic analogs (Tc1, Tc2, Tc9, Tc17, Tc22, Tfc) (**Figure S15**).[3–7] CD25 and FoxP3 were used to identify Tregs within both $CD4^+$ and $CD8^+$ compartments, given their impact on CAR T efficacy.[8] CD56 was included to detect $CD3^+CD56^+$ NK-like T cells, a subset implicated in CAR T function and persistence.[9]

T Cell Differentiation and Memory Phenotypes

CD45RA, CCR7, and CD95 were used to define naïve and memory subsets. Naïve T cells (T_N) were defined as $CD45RA^+CCR7^+CD27^+CD28^+CD95^-$, and stem cell memory ($T_{SCM}$) as $CD45RA^+CCR7^+CD27^+CD28^+CD95^+$. Central memory ($T_{CM}$), effector memory ($T_{EM}$), and terminal effector (T_{TE}) subsets were further classified by their distinct CD45RA and CCR7 expression.[10] CD27 and CD28 provided additional resolution of maturation stages (early-like, early, and intermediate effector memory) (**Figure S15**).

Activation, Metabolic, Exhaustion, Senescence, and Apoptotic Markers

Surface and intracellular markers were selected to monitor activation status, metabolic programming, and exhaustion. Early activation was marked by CD69, whereas CD25 captured mid and late activation states.[11] Proliferation was assessed via Ki-67.[12] To profile metabolic fitness, GLUT1 and GAPDH were included, both upregulated during glycolysis. GLUT1 reflects increased glucose transport upon T cell activation, [13] and intracellular GAPDH serves as an alternative marker of glycolytic flux.[14] Hif-1a was used to evaluate potential shifts to anaerobic metabolism.[15] Exhaustion markers included PD1, TIM3, and LAG3 (**Figure S15**).[16] CD36, a fatty acid transporter linked to lipid stress and dysfunction in $CD8^+$ tumor-infiltrating lymphocytes, was included to assess metabolic impairment.[17] CD57 and active caspase 3 were used to identify senescent and apoptotic populations, respectively (**Figure 1**). [18,19]

Functionality Assessment

A dual-fluorescent protein cytotoxicity readout was integrated directly into the phenotyping assay. CAR T cells were co-cultured with $CD19^+$ Nalm6-YFP^+ target and $CD19^-$ Nalm6-GFP^+ control cells. YFP^+/GFP^+ quantification provided a killing index for antigen-specific cytotoxicity (analogous to a VITAL assay).[20] Granzyme B and perforin were also measured intracellularly to evaluate effector function (**Figure S15**). Degranulation inhibitors were not used during co-culture to preserve physiological function and marker expression.

Engineering Readouts

To assess product identity, the panel included markers relevant to CAR T cell engineering. HLA class I (A, B, C) and class II (DP, DQ, DR) molecules are genes of interest for allogeneic products. Further, HLA class

II can also be used as an activation marker. CD3 and CAR markers allow for monitoring of TCR knockout, and CAR editing efficiency, respectively.[21]

Gating Strategy and Controls

Dead cells and debris were excluded using Zombie NIR viability dye. CD45 served as a pan-leukocyte marker to include T cells and exclude non-hematopoietic events such as Nalm6 cells. Sequential gating on viable singlets, $GFP^{-}YFP^{-}$, and $CD45^{+}$ lymphocytes ensured a clean T cell population for downstream analysis. Due to the complexity of the 36-parameter panel on a 4-laser, 48-channel spectral cytometer, fluorochrome selection, and gating hierarchy were guided by evaluating spectral similarity, signal spread, and stain index values (**Figures S18 and S19**). Fluorescence-minus-one (FMO) controls were generated for each marker (**Figure S11**), and all gates were validated using fully stained reference samples. Staining was verified across manufacturing days using internal positive controls (**Figure S3**).

Panel Optimization

Each antibody was titrated using serial dilutions (typically 1:20 to 1:320), and the lowest concentration with optimal stain index and intensity was selected. Multiple optimization rounds were performed to adjust for fluorochrome interactions in the full master mix. This was critical in a high-dimensional context, where minor shifts in intensity could affect rare subset detection. Protocol refinements included sequential (pre-stain) staining for improved resolution of CXCR3 (**Figure S20A**), higher antibody volumes for CD69 (**Figure S20B**), and lower antibody volumes for CD25 to accommodate activation-induced expression changes (**Figure S20C**).

Spectral Overlap and Unmixing Performance

Spectral overlap was addressed using rigorous controls. FMO controls were acquired for all markers to enable accurate gating in the presence of full spectral complexity (**Figure S11**). This was particularly important for dim or partially overlapping fluorochromes such as GLUT1 Alexa Fluor 647 and IRF4 APC, which may be modulated by antigen engagement. For low-abundance targets, single-stain compensation beads were used, and bead- versus cell-derived spectral signatures were compared to quantify spectral differences (**Figure S21**).

Overall staining performance was evaluated by calculating stain index values for each marker in fully stained versus single-color conditions (**Figure S22**). To understand the influence of spread on fluorochrome resolution, we assessed the robust Standard Deviation (rSD) and stain indices of single-color controls when unmixed against the single color or the entire 36-color unmixing matrix. Fluorescent spread (rSD) of some markers (VioGreen, VioBlue, PerCP-Cy5.5) exhibited a 4- to 6-fold increase in the standard deviation of negative populations when unmixed with the 36-color matrix (**Figure S23**), highlighting the importance of careful marker to fluorophore matching. Spillover spreading matrices (**Figure S24**) were further used to guide fluorochrome-marker pairing, to minimize the impact of spread in co-expressed targets. For example, CXCR5 was placed on PE-Cy7 despite potential spread from RB780 (Ki-67) and PE-Cy5 (CXCR3). This was acceptable because, at day 0, Ki-67 expression is low and $CXCR5^{+}$ Tfh cells do not co-express CXCR3. By day 5, when Ki-67 is elevated, $CXCR5^{+}$ Tfh cells are rare (**Figure S25**). Despite the complexity of the panel, minimal manual compensation adjustments were required post-unmixing, with moderate spectral spread considered acceptable given the high dimensionality of the panel (**Figure S12**).

Panel Modularity

Panel flexibility was demonstrated by substituting GLUT1 with GAPDH in one donor sample, without impacting marker expression patterns (**Figure S26**). This modularity supports future adaptation, such as integrating alternative metabolic probes or additional markers, without compromising dataset continuity.

Staining Protocol for the Full-Spectrum Flow Cytometry Panel

1. **Cell aliquots:** Count cells and aliquot 1E6 cells for each fully stained sample. For each control condition (unstained, Single Color (SC) Controls, or FMO), harvest ~1E5 cells. Transfer cells into FACS tubes.
2. **Wash and blocking:** Resuspend cells in 1 mL of BSA stain buffer and centrifuge at 300 × g for 5 min. Decant the supernatant and blot the tube on a paper towel to remove residual liquid. Next, add 10 µL of Fc blocking mix (containing 1 µL human Fc receptor blocking reagent [BD Bioscience, 564220] and 9 µL Brilliant Stain Buffer [BD Biosciences, 566349]) to each sample and incubate for 10 min at RT. *For the unstained control, add 10 µL of Brilliant Stain Buffer in place of the Fc block mix.*
3. **CAR detection:** Add 15 µL of the CAR detection mix (anti-FMC63 CAR antibody diluted in Brilliant Stain Buffer) to each tube and incubate for 10 min at RT in the dark. *For unstained, non-CAR SC, and FMO controls, add 15 µL of Brilliant Stain Buffer instead of the CAR antibody mix.*
4. **Pre-stain:** Add 25 µL of the pre-stain antibody master mix containing chemokine and chemoattractant receptor antibodies (CXCR3, CXCR5, CCR6, CCR7, CRTH2, in Brilliant Stain Buffer) to each sample. Incubate on ice for 30 min, protected from light. *For unstained/SC/FMO controls, add 25 µL of Brilliant Stain Buffer in place of the antibody master mix.*
5. **Extracellular staining:** Add 50 µL of the main extracellular antibody master mix (remaining surface markers and viability dye, all diluted in Brilliant Stain Buffer) to each tube. Incubate on ice for 30 min, protected from light. *For unstained/non-extracellular SC/FMO controls, add 50 µL of buffer instead of the antibody master mix.*
6. **Post-stain wash:** Add 1 mL of BSA buffer to each tube and centrifuge at 300 × g for 5 min. Decant the supernatant and gently blot the tube dry.
7. **Fixation/permeabilization:** Add 1 mL of Fixation/Permeabilization buffer (eBioscience™ Foxp3 / Transcription Factor Staining Buffer Set, [eBioscience Cat. 00-5523-00] prepared according to the manufacturer's instructions) to each sample. Incubate for 30 min at 4°C in the dark.
8. **Washes:** Wash cells twice with 1 mL of 1× Permeabilization buffer. For each wash, resuspend the cells in 1 mL 1× Permeabilization buffer, then centrifuge at 300 × g for 5 min and decant the supernatant.
9. **Intracellular staining:** Add 100 µL of the intracellular antibody master mix (all intracellular antibodies diluted in 1× Permeabilization buffer) to each tube. Incubate on ice for 30 min, protected from light. *For unstained/non-intracellular SC/FMO controls, add 100 µL of permeabilization buffer with no antibody.*
10. **Intracellular washes:** Wash cells two times with 1× Permeabilization buffer. For each wash, add 2 mL of 1× Permeabilization buffer, then centrifuge at 300 × g for 5 min. Decant the supernatant and blot dry between washes.
11. **Final resuspension:** Resuspend the stained cell pellet in ~200 µL of DPBS for data acquisition. Keep samples at 4°C and protected from light until acquisition.

Table S1. Composition and functional categorization of the 36-marker spectral flow cytometry panel for CAR T cell phenotyping.

This table lists all 36 markers used in the spectral panel, organized by functional category, the conjugated fluorochrome used for spectral detection, and the antibody clone.

Group	Marker	Function	Fluorochrome	Clone
Live/Dead	Viability	Discrimination of live/dead cells	Zombie NIR	N/A
Cell Identification	GFP	Track control cell % during killing assays	GFP	N/A
	YFP	Track target cell % during killing assays	YFP	N/A
	CD45	Hematopoietic lineage, target cell discrimination and gating backbone	cFluor V547	HI30
CAR Characterization	CAR	CAR expression detection	PE	REA1297
	HLA-A, B, C	KO gene of interest	PerCP	W6/32
	HLA-DP, DQ, DR	KO gene of interest, upregulation in activated T cells	Spark Blue 574	Tü39
T Cell Markers	CD3	T cell identification and purity verification	APC-Fire 750	UCHT1
	CD4	Helper T cell subset gating	cFluor R840	SK3
	CD8	Cytotoxic T cell subset gating	BV786	SK1
Cell Subtyping	CD56	NK and NKT cell identification	Super Bright 436	TULY56
	FoxP3	Regulatory T cell identification	RB613	259D/C7
T Cell Subtyping	CXCR3 (CD183)	Th1 or Tc1 subset identification ($CXCR3^{+}$)	PE-Cy5	G025H7
	CRTH2 (CD294)	Th2 or Tc2 subset identification ($IRF4^{+}$ $CRTH2^{+}$)	BV711	BM16
	IRF4	Associated with Th9- or Tc9-like phenotypes (IRF4+ CRTH2-CCR6-CD161-)	APC	REA201
	CD161	Th17 or Tc17 subset identification ($IRF4^{+}$ $CRTH2^{-}$ $CCR6^{+}CD161^{+}$)	RB705	HP-3G10
	CCR6 (CD196)	Th22 or Tc22 subset identification ($IRF4^{-}$ $CRTH2^{-}$ $CXCR5^{-}CCR6^{+}$)	BV421	G034E3
	CXCR5 (CD185)	Tfh- or Tfc-like cell identification ($IRF4^{-}CRTH2^{-}$ $CXCR5^{+}CCR6^{-}$)	PE-Cy7	J252D4
Stemness	CCR7 (CD197)	Naïve, stem cell memory and central memory T cell marker	Spark NIR 685	G043H7
	CD45RA	Naïve, stem cell memory and terminal effector T cell marker	BV570	HI100

	CD95 (Fas)	Stem cell memory subset marker within the CD45RA+CCR7+ repertoire	PE-Fire 700	DX2
	CD27	Differentiation subtyping T cell marker	BV750	O323
	CD28	Differentiation subtyping T cell marker	BV605	CD28.2
Activation	CD69	Early activation marker, upregulated upon stimulation	BV480	FN50
	CD25	IL-2 receptor alpha, mid- and late activation marker	PE-Fire 744	M-A251
Exhaustion	PD1 (CD279)	Checkpoint molecule involved in T cell exhaustion (target of anti-PD1 therapy)	PE-Fire 640	EH12.2H7
	TIM3 (CD366)	Checkpoint molecule involved in T cell exhaustion	PE-Fire 810	F38-2E2
	LAG3 (CD223)	Checkpoint molecule involved in T cell exhaustion	PE-CF594	T47-530
Senescence & Proliferation	CD57	Senescence marker	PerCP-Cy5.5	QA17A04
	Active Caspase 3	Apoptosis marker	BV650	C92-605.rMAb
	Ki-67	Proliferation marker indicating active cell cycle	RB780	B56
Metabolism	GLUT1 or GAPDH	Glucose transporter and glycolytic enzyme, metabolic activation indicator	Alexa Fluor 647	EPR3915 or EPR16891
	Hif-1a	Hypoxia-induced metabolic regulator	Alexa Fluor 700	H1alpha67
	CD36	Fatty acid transporter linked to T cell dysfunction in tumors	VioBlue	REA760
Effector Function	Granzyme B	Cytotoxic effector molecule involved in target cell apoptosis	RB744	GB11
	Perforin	Cytotoxic effector molecule involved in granule-mediated apoptosis	VioGreen	REA1061

Table S2. Suggested condensed flow cytometry panel for GMP manufacturing and release.

A streamlined subset of the full 36-marker panel, designed to support key phenotypic characteristics for GMP manufacturing and routine quality control.

Marker	Justification
**Zombie NIR**	Live/dead discrimination (vital release criteria).
**CD45**	Pan-leukocyte marker used as main marker for $CD3^+$ cell identification.
**CD3**	Confirms T cell lineage and overall T cell purity.
**CD4**	Determines helper T cell subset proportion (CD4/CD8 ratio).
**CD8**	Determines cytotoxic T cell subset proportion (CD4/CD8 ratio).
**CAR**	Measures editing efficiency, a core release metric.
**CCR7**	(Pairs with CD45RA) Tracks differentiation trajectories (T_N/T_{SCM}, T_{CM}, T_{EM}, T_{TE}).
**CD45RA**	(Pairs with CCR7) Tracks differentiation trajectories (T_N/T_{SCM}, T_{CM}, T_{EM}, T_{TE}).

Table S3. Antibody titration cell sources.

This table summarizes antibody titration conditions, including tested cell types and stimulation reagents used to optimize signal resolution for each marker.

Marker	Target Cell Type	Stimulation Condition
CXCR3 (CD183)	PBMCs	Resting
CD56	PBMCs	Resting
FoxP3	PBMCs	Resting
CD36	PBMCs	Resting
CCR6 (CD196)	Isolated T cells	Resting
CD45	Isolated T cells	Resting
CD45RA	Isolated T cells	Resting
CD28	Isolated T cells	Resting
CD161	Isolated T cells	Resting
CD27	Isolated T cells	Resting
CD8	Isolated T cells	Resting
HLA-A, B, C	Isolated T cells	Resting
CRTH2 (CD294)	Isolated T cells	Resting
CCR7 (CD197)	Isolated T cells	Resting
Viability	Isolated T cells	Live/dead stain
CD3	Isolated T cells	Resting
CD4	Isolated T cells	Resting
CD69	Isolated T cells	TransAct Activated (48h)
IRF4	Isolated T cells	TransAct Activated (48h)
HLA-DP, DQ, DR	Isolated T cells	TransAct Activated (72h)
CD57	Isolated T cells	TransAct Activated (72h)
Ki-67	Isolated T cells	TransAct Activated (72h)
LAG3 (CD223)	Isolated T cells	TransAct Activated (72h)
PD1 (CD279)	Isolated T cells	TransAct Activated (72h)
CD95	Isolated T cells	TransAct Activated (72h)
CD25	Isolated T cells	TransAct Activated (72h)
CXCR5 (CD185)	Isolated T cells	TransAct Activated (72h)
TIM3 (CD366)	Isolated T cells	TransAct Activated (72h)
GLUT1	Isolated T cells	TransAct Activated (72h)
GAPDH	Isolated T cells	TransAct Activated (72h)
Hif-1a	Isolated T cells	TransAct Activated (72h)
CAR	CAR^+ T cells	Cultured CAR^+ T cells
Perforin	CAR^+ T cells + Nalm6	Co-culture with $CD19^+$ target cells
Granzyme B	CAR^+ T cells + Nalm6	Co-culture with $CD19^+$ target cells
Active Caspase 3	CAR^+ T cells	Induced apoptosis

Table S4. Prepared buffers and master mixes for staining protocol.

This table lists the composition of all buffers and master mixes used throughout the spectral staining workflow.

Buffer/Master Mix	Components	Purpose
Fixation/Permeabilization Buffer	1:4 dilution of Fixation/Permeabilization concentrate to diluent solution	Fixation buffer
1× Permeabilization Buffer	1:10 dilution of 10× Permeabilization Buffer in cell culture water	Intracellular staining and wash buffer
Cell Culture Media (Option A)	RPMI + 10% FBS + 1% GlutaMAX	Standard CAR T and T cell culture medium
Cell Culture Media (Option B)	TexMACS + 3% hABS + 12.5 ng/mL IL-7 and IL-15	Standard CAR T and T cell culture medium
Fc Receptor Blocking Master Mix	Fc block reagent + Brilliant Stain Buffer	Reduce non-specific Fc receptor antibody binding
CAR Detection Master Mix	Anti-FMC63 CAR antibody + Brilliant Stain Buffer	CAR detection reagent
Pre-Stain Master Mix	CXCR5 (CD185), CCR6 (CD196), CRTH2 (CD294), CCR7 (CD197), CXCR3 (CD183) + Brilliant Stain Buffer	Chemokine and chemoattractant receptor staining before extracellular stain
Extracellular Master Mix	CD36, CD69, HLA-DP, DQ, DR, CD57, LAG3 (CD223), PD1 (CD279), CD95, CD25, TIM3 (CD366), Viability, CD56, CD45, CD45RA, CD28, CD161, CD27, CD3, CD8, HLA-A, B, C, CD4 + Brilliant Stain Buffer	Comprehensive extracellular staining panel
Intracellular Master Mix	Ki-67, Hif-1a, Perforin, Granzyme B, Active Caspase 3, GLUT1 or GAPDH, FoxP3, IRF4 + Permeabilization Buffer	Comprehensive intracellular staining panel

Table S5. Reference controls for spectral unmixing

This table summarizes reference controls for each marker, including fluorophore and sample type, and reports the MFI of the brightest 200 events to support accurate spectral unmixing.

Marker	Fluorochrome	Reference Control	
		Type	MFI of top >200 events
GLUT1	Alexa Fluor 647	Cells	1.07E+06
GAPDH			
Hif-1a	Alexa Fluor 700	Beads	2.59E+05
IRF4	APC	Cells	3.38E+05
CD3	APC-Fire 750	Cells	2.65E+05
CCR6 (CD196)	BV421	Beads	3.37E+05
CD69	BV480	Cells	6.67E+05
CD45RA	BV570	Cells	2.00E+05
CD28	BV605	Beads	5.39E+05
Active Caspase 3	BV650	Cells	4.76E+05
CRTH2 (CD294)	BV711	Beads	4.56E+05
CD27	BV750	Beads	3.89E+05
CD8	BV786	Cells	2.70E+06
CD4	cFluor R840	Cells	4.13E+05
CD45	cFluor V547	Cells	1.73E+05
GFP	GFP	Cells	1.55E+06
CAR	PE	Cells	4.18E+06
LAG3 (CD223)	PE-CF594	Beads	5.70E+05
CXCR3 (CD183)	PE-Cy5	Cells	1.10E+06
CXCR5 (CD185)	PE-Cy7	Beads	1.26E+06
PD1 (CD279)	PE-Fire 640	Cells	5.29E+05
CD95 (Fas)	PE-Fire 700	Beads	1.20E+06
CD25	PE-Fire 744	Cells	1.51E+06
TIM3 (CD366)	PE-Fire 810	Beads	4.31E+05
HLA-A, B, C	PerCP	Cells	1.59E+06
CD57	PerCP-Cy5.5	Cells	1.25E+06
FoxP3	RB613	Beads	3.60E+05
CD161	RB705	Beads	1.09E+06
Granzyme B	RB744	Cells	9.44E+05
Ki-67	RB780	Cells	8.11E+05
HLA-DP, DQ, DR	Spark Blue 574	Cells	1.28E+06
CCR7 (CD197)	Spark NIR 685	Beads	2.79E+05
CD56	Super Bright 436	Beads	1.76E+06
CD36	VioBlue	Cells	2.50E+05
Perforin	VioGreen	Cells	1.00E+06
YFP	YFP	Cells	9.81E+04
Viability	Zombie NIR	Cells	4.13E+05

Table S6. Statistical analysis of Th/Tc and T cell differentiation subsets during 24 h restimulation.

p-values from two-way repeated measures ANOVA with Tukey's multiple comparisons test (p = 0.05) are shown for $CD4^+$ helper (Th), $CD8^+$ cytotoxic (Tc), T_N, T_{SCM}, T_{CM}, T_{EM}, and T_{TE} subsets within CAR^+ and CAR^- compartments across 0 h, 3 h, 12 h, 15 h, and 24 h. Data are presented separately for day 5 and day 10 products (n = 3 donors). Only Th/Tc subsets representing ≥0.5% of total $CD3^+$ T cells at any time point or in any donor are shown. AN, "all negative" ($CXCR3^-IRF4^-CRTH2^-CCR6^-CXCR5^-$). "NA" denotes comparisons with no significant difference between timepoints.

			Th / Tc Subsets												Memory & Differentiation Subsets						
Timepoint	CAR+/-	Comparison	Th1	Tc1	Th2	Tc2	Th9	Tc9	Th17	Th22	Tc22	Tfh	CD4+ AN	CD8+ AN	T_N	T_{SCM}	T_{CM}	Early T_{EM}	Early-like T_{EM}	Intermediate T_{EM}	T_{TE}
Day 5	CAR+	0h (baseline) vs. 3h (co-culture)	0.9937	0.0925	0.3862	0.8185	0.8356	0.7795	0.2631	0.1335	0.7600	0.8527	0.0243	0.4268	0.5985	0.9993	0.7132	0.5068	0.8429	0.8448	0.9295
		0h (baseline) vs. 12h (+9h co-culture)	0.8731	0.9998	0.5976	0.8368	0.9931	0.9793	0.9995	0.0750	0.7866	0.9883	0.4127	0.1258	0.6467	0.0457	0.9959	0.5133	0.8647	0.7911	0.9833
		0h (baseline) vs. 15h (+3h restim)	>0.9999	0.5066	0.5526	0.8596	0.3864	0.7991	0.7565	0.7288	0.4977	>0.9999	0.7813	0.0421	0.5313	0.2816	0.8743	0.4446	0.8575	0.8680	0.8085
		0h (baseline) vs. 24h (+12h restim)	0.8796	0.7894	0.4878	0.7832	0.3924	0.3622	0.5859	0.0106	0.3453	>0.9999	0.4309	0.1880	>0.9999	0.6405	0.1348	0.4870	0.9998	>0.9999	0.6953
		0h (baseline) vs. 24h (n.r.)	0.1355	>0.9999	0.3409	0.7833	0.5256	0.7825	0.4278	0.7538	0.9940	0.6089	0.6160	0.9500	0.9807	0.4331	0.4671	0.0560	0.1580	0.9488	0.8895
		3h (co-culture) vs. 12h (+9h co-culture)	0.8467	0.9297	0.8401	0.8543	0.8182	0.2798	0.5931	0.9993	0.9909	0.9987	>0.9999	>0.9999	0.7604	0.9415	0.9833	0.9978	0.9991	0.9099	0.9849
		3h (co-culture) vs. 15h (+3h restim)	0.9997	0.6977	0.8485	0.8891	0.9788	0.8142	0.4863	0.8778	0.9996	0.9851	0.9998	>0.9999	0.5733	0.4084	0.8108	0.3396	0.8431	0.9926	0.9684
		3h (co-culture) vs. 24h (+12h restim)	0.6680	>0.9999	0.5979	0.8374	0.1100	0.9998	0.4458	0.9867	0.8996	0.9894	0.9983	0.2288	0.7397	0.0055	0.0387	0.1280	0.9454	0.1205	0.3819
		3h (co-culture) vs. 24h (n.r.)	0.1079	0.9381	0.3467	0.8027	0.1379	0.7790	0.3685	0.4730	0.7942	0.5776	0.3792	0.8169	0.7448	0.1282	0.5333	0.1029	0.4109	0.0133	>0.9999
		12h (+9h co-culture) vs. 15h (+3h restim)	0.7429	0.2586	0.5258	0.9760	0.6259	0.6928	0.7370	0.6315	0.7491	0.8356	0.9751	0.9998	0.6869	0.1157	0.6558	0.2705	0.6016	0.5316	0.6467
		12h (+9h co-culture) vs. 24h (+12h restim)	0.0558	0.9911	0.8180	0.8475	0.3260	0.9998	0.6443	0.5702	0.3103	0.9417	0.7936	0.5087	0.7630	0.3195	0.2158	0.4649	0.9955	0.5695	0.4465
		12h (+9h co-culture) vs. 24h (n.r.)	>0.9999	>0.9999	0.4136	0.8237	0.9045	0.8683	0.4611	0.3142	0.0545	0.3207	0.1668	0.8103	0.7538	0.1586	0.4736	0.0147	0.3487	0.2514	0.9168
		15h (+3h restim) vs. 24h (+12h restim)	0.8454	0.9207	0.6115	0.8720	0.8991	0.9309	0.5900	0.5607	0.1149	>0.9999	0.5748	0.6208	0.7178	0.9965	0.3271	>0.9999	0.9939	0.5881	0.0870
		15h (+3h restim) vs. 24h (n.r.)	0.8059	0.7864	0.4559	0.8443	0.4256	0.7928	0.4017	0.2066	0.3152	0.2291	0.0774	0.7646	0.7276	0.9556	0.5596	0.1559	0.4439	0.1864	0.9228
		24h (+12h restim) vs. 24h (n.r.)	0.3069	0.1294	0.2956	0.7734	0.0708	0.4949	0.2982	0.3234	0.1399	0.2530	0.1184	0.7359	0.7637	>0.9999	0.2535	0.4039	0.7320	0.3834	0.4650
	CAR-	0h (baseline) vs. 3h (co-culture)	0.9059	0.9385	0.3255	0.7313	0.3691	0.4698	0.4629	0.6201	0.9007	0.9912	0.9698	0.9839	>0.9999	0.9991	0.4506	0.3633	0.8992	0.7728	0.9906
		0h (baseline) vs. 12h (+9h co-culture)	0.9391	0.9981	0.4277	0.7974	0.9461	0.9924	0.9791	0.1056	0.4582	0.9075	0.0858	0.3411	0.2827	0.1351	0.4255	0.0161	>0.9999	0.6192	0.0845
		0h (baseline) vs. 15h (+3h restim)	0.8348	0.2288	0.3999	0.2988	0.7592	>0.9999	0.4687	0.2829	0.2476	0.7120	0.9853	0.6525	0.2942	0.8708	0.1249	0.2697	0.6285	0.6760	0.2063
		0h (baseline) vs. 24h (+12h restim)	0.3155	0.9760	0.9977	0.6729	0.1790	0.5267	0.8142	0.1221	0.3002	0.0208	0.9998	0.6414	0.9576	0.3626	0.8264	0.0624	>0.9999	0.6853	0.0623
		0h (baseline) vs. 24h (n.r.)	0.4588	0.2676	0.0956	0.5168	0.3596	0.7071	0.3240	0.9990	0.5179	0.5507	0.3075	0.9996	0.7467	0.3809	0.3915	0.5791	0.9990	0.9375	0.4602
		3h (co-culture) vs. 12h (+9h co-culture)	0.4550	0.1669	0.7204	0.9267	0.9671	0.7226	0.7889	0.5292	0.8385	0.8563	>0.9999	0.8329	0.2317	0.8880	0.0811	0.2889	0.8298	0.6228	0.3794
		3h (co-culture) vs. 15h (+3h restim)	0.8194	0.6378	0.2880	0.8122	0.4383	0.5296	0.4560	0.9992	0.9904	0.7784	0.9758	0.9998	0.2173	0.9720	0.0854	0.9999	0.7117	0.7113	0.4059
		3h (co-culture) vs. 24h (+12h restim)	0.5103	0.3258	0.1501	0.8093	0.0555	0.9964	0.6266	0.3172	0.3421	0.9998	0.9702	0.2015	0.7360	0.6313	0.9993	0.4479	0.9927	0.6915	0.1756
		3h (co-culture) vs. 24h (n.r.)	0.9789	0.2141	0.0692	0.6455	0.3220	0.5490	0.3545	0.6722	0.9835	0.3995	0.5348	0.8775	0.7593	0.8521	0.3864	0.3296	0.9929	0.9833	0.7030
		12h (+9h co-culture) vs. 15h (+3h restim)	>0.9999	0.9329	0.5438	0.8534	0.8474	0.9975	0.7144	0.6552	0.9965	0.4680	0.7173	0.6331	0.3413	0.2468	0.9998	0.1248	0.7831	0.4378	0.9053
		12h (+9h co-culture) vs. 24h (+12h restim)	0.3541	0.8356	0.5023	0.8586	0.5747	0.3811	0.9997	0.2926	0.1038	0.3947	0.9905	0.9984	0.6775	0.0755	0.3195	0.3964	0.9999	0.5859	0.8518
		12h (+9h co-culture) vs. 24h (n.r.)	0.5918	0.3570	0.2535	0.7152	0.5781	0.8659	0.3397	0.3225	0.1463	0.4239	0.2599	0.7207	0.0557	0.0121	>0.9999	0.1234	0.9997	0.0624	>0.9999
		15h (+3h restim) vs. 24h (+12h restim)	0.1171	0.9765	0.9299	0.9948	0.2330	0.6134	0.3714	0.5951	0.6192	0.3317	0.9977	0.8222	0.7291	0.9932	0.3949	0.7978	0.9728	0.7405	>0.9999
		15h (+3h restim) vs. 24h (n.r.)	0.6487	0.9750	0.0508	0.4629	0.0746	0.6047	0.1622	0.4987	0.9972	0.4999	0.2870	0.9795	0.2000	0.9972	>0.9999	0.1273	0.5163	0.3129	0.8960
		24h (+12h restim) vs. 24h (n.r.)	0.5334	0.4731	0.1482	0.5051	0.1996	0.6096	0.2084	0.3147	0.0574	0.4150	0.6690	0.6028	0.9978	0.9828	0.5334	0.2700	0.9988	>0.9999	0.9928
Day 10	CAR+	0h (baseline) vs. 3h (co-culture)	0.1954	0.8128	0.1895	0.4122	0.5030	0.5115	0.5774	0.5521	0.4424	0.9070	0.8433	0.8262	NA	0.8596	0.4590	0.9941	0.9969	0.5557	0.7458
		0h (baseline) vs. 12h (+9h co-culture)	0.0388	0.9998	0.1817	0.7316	<0.0001	0.2835	0.6825	0.4384	0.4816	0.3611	0.0218	0.0622	NA	>0.9999	0.3069	0.4503	0.3421	0.7115	0.4948
		0h (baseline) vs. 15h (+3h restim)	0.0165	0.9998	0.2056	0.6861	0.0715	0.1867	0.5947	0.4554	0.2278	0.3148	0.0360	0.1766	NA	0.8404	0.4529	0.4723	0.9635	0.9240	0.9746
		0h (baseline) vs. 24h (+12h restim)	0.0663	0.9855	0.3704	0.7388	0.1052	0.3426	0.7870	0.4371	0.1349	0.2520	0.0459	0.1398	NA	0.8421	0.3382	0.4682	0.9810	>0.9999	0.9934
		0h (baseline) vs. 24h (n.r.)	0.0627	0.9973	0.3089	0.7511	0.4609	0.4177	0.8727	0.4207	0.6133	0.2744	0.0391	0.1802	0.7197	0.8863	0.2927	0.4788	0.1254	0.0436	0.9732
		3h (co-culture) vs. 12h (+9h co-culture)	0.0408	0.2511	0.4486	0.9193	0.9989	>0.9999	0.9580	0.6764	0.7918	0.6311	0.4319	0.4942	NA	0.4027	0.2858	0.2314	0.0067	0.2549	0.9839
		3h (co-culture) vs. 15h (+3h restim)	0.0114	0.4734	0.3480	0.8358	0.9994	0.9499	0.8448	0.6205	0.3524	0.4256	0.2825	0.4117	NA	0.9652	0.4523	0.3623	>0.9999	0.3151	0.8348
		3h (co-culture) vs. 24h (+12h restim)	0.0694	0.5545	0.9978	0.8960	0.9614	0.8304	0.8843	0.5415	0.3138	0.3274	0.1966	0.3206	NA	0.6474	0.3351	0.4198	>0.9999	0.9392	0.3902
		3h (co-culture) vs. 24h (n.r.)	0.0650	0.2021	0.6071	0.9253	0.8612	>0.9999	0.5984	0.6080	0.9987	0.3316	0.2853	0.4558	0.7197	0.9997	0.2864	0.4944	0.6260	0.7046	0.3047
		12h (+9h co-culture) vs. 15h (+3h restim)	0.2506	0.5351	0.2839	0.4901	0.9981	0.5856	0.9013	0.6435	0.7371	0.2814	0.2318	0.7939	NA	0.1461	0.9641	0.9403	0.5818	0.9243	>0.9999
		12h (+9h co-culture) vs. 24h (+12h restim)	0.1274	0.5307	0.0153	0.8380	0.1527	0.4123	0.5917	0.4698	0.8501	0.0964	0.2555	0.7650	NA	0.3646	0.3623	0.5943	0.4889	0.3090	0.8197
		12h (+9h co-culture) vs. 24h (n.r.)	0.0947	0.9091	0.1204	0.7235	0.1478	0.1509	0.5644	0.7888	0.0091	0.0540	0.3233	0.9964	0.7197	0.4960	0.3288	0.8916	0.1017	0.2982	0.6913
		15h (+3h restim) vs. 24h (+12h restim)	0.4671	0.9669	0.0848	0.9998	0.4585	0.7481	0.4087	0.2756	0.9842	0.0149	0.4097	>0.9999	NA	0.8931	0.3165	0.4962	0.9985	0.9253	0.2263
		15h (+3h restim) vs. 24h (n.r.)	0.3758	0.4278	0.1712	0.6635	0.3365	0.4593	0.4247	0.8092	0.2786	0.2337	0.1743	0.2271	0.7197	0.9780	0.2747	0.8673	0.9031	0.4652	0.1937
		24h (+12h restim) vs. 24h (n.r.)	0.8730	0.6685	0.4451	0.7341	0.1423	0.3256	0.5534	0.5375	0.4730	0.4817	0.2520	0.0456	0.7197	0.9998	0.4280	0.5757	0.3378	0.7926	0.8159
	CAR-	0h (baseline) vs. 3h (co-culture)	0.2826	0.9774	0.2836	0.3918	0.7436	0.7486	0.5314	0.5684	0.5833	0.3889	0.7797	0.9903	0.1207	0.8712	0.9933	0.3012	0.0309	0.0536	0.7879
		0h (baseline) vs. 12h (+9h co-culture)	0.9471	0.5302	0.0142	0.6905	0.0469	0.4382	0.7677	0.2768	0.5155	0.7250	0.8910	0.7478	0.8948	0.9991	0.5672	0.4760	0.0211	0.9257	0.9963
		0h (baseline) vs. 15h (+3h restim)	0.6103	0.8547	0.4491	0.6744	0.1112	0.4719	>0.9999	0.1318	0.5934	>0.9999	0.3120	0.7520	0.8383	0.7027	0.9997	0.4353	0.0289	0.7411	0.5607
		0h (baseline) vs. 24h (+12h restim)	0.7906	0.9274	0.5251	0.7714	0.1992	0.4003	0.9391	0.1804	0.4356	0.3267	0.1757	>0.9999	0.0085	0.4334	>0.9999	0.3782	0.0538	>0.9999	0.3369
		0h (baseline) vs. 24h (n.r.)	0.4089	0.4345	>0.9999	0.8095	>0.9999	0.9970	0.5099	0.0728	0.6506	0.1128	0.7507	0.5852	0.8148	0.1423	>0.9999	0.3177	0.2091	0.6713	0.5727
		3h (co-culture) vs. 12h (+9h co-culture)	0.1145	0.1527	0.7743	0.9195	0.9156	0.8576	0.5877	0.6014	0.5064	0.0239	0.9407	0.6363	0.7227	0.1815	0.5673	0.6189	0.2108	0.5887	0.7223
		3h (co-culture) vs. 15h (+3h restim)	0.0307	0.2487	0.7378	0.8137	0.9398	0.9624	0.6762	>0.9999	0.8989	0.5090	>0.9999	0.7813	0.6639	>0.9999	>0.9999	0.5987	0.8866	0.2423	>0.9999
		3h (co-culture) vs. 24h (+12h restim)	0.5770	>0.9999	0.9999	0.9711	0.9723	>0.9999	0.8342	0.8826	0.6074	0.9031	0.9580	0.9943	0.0263	0.1701	0.9989	0.5079	0.2055	0.6304	0.9985
		3h (co-culture) vs. 24h (n.r.)	0.0286	0.1346	0.5223	0.5070	0.7950	0.8076	0.5171	0.9997	0.8108	0.9768	0.9932	0.6139	0.6318	0.0408	0.9997	0.4649	0.9940	0.8969	0.9998
		12h (+9h co-culture) vs. 15h (+3h restim)	0.9794	>0.9999	0.8253	0.6615	0.9401	0.7627	0.6591	0.8294	0.8705	0.8832	0.6139	0.9982	0.1982	0.9210	0.1952	>0.9999	0.3128	>0.9999	0.2738
		12h (+9h co-culture) vs. 24h (+12h restim)	0.0606	0.1108	0.7546	0.0208	0.8194	0.5195	0.6072	0.9997	0.8765	0.5597	0.9997	0.0043	0.2590	0.0109	0.0372	0.6078	>0.9999	0.6031	0.0118
		12h (+9h co-culture) vs. 24h (n.r.)	0.0336	0.6747	0.0696	0.6314	0.2849	0.4547	0.6106	0.7696	0.7929	0.2317	0.9911	0.9939	0.9604	0.0264	0.4596	>0.9999	0.2321	0.4889	0.0811
		15h (+3h restim) vs. 24h (+12h restim)	0.1341	0.1735	0.4377	0.5487	0.5705	0.3415	0.5637	0.4825	0.9956	0.8763	0.4552	0.2858	0.2337	0.7455	0.9829	0.0369	0.5171	0.2569	0.8779
		15h (+3h restim) vs. 24h (n.r.)	0.5861	0.5138	0.4166	0.6415	0.2644	0.3564	0.6335	0.9219	0.9809	0.2659	0.1685	>0.9999	>0.9999	0.3166	0.9593	0.9999	>0.9999	0.4234	0.9653
		24h (+12h restim) vs. 24h (n.r.)	0.0056	0.0201	0.3754	0.7079	0.2916	0.3244	0.6059	0.6772	0.9990	0.8410	0.9956	0.2362	0.2057	0.3276	>0.9999	0.6785	0.6174	0.7920	0.9972

Table S7. Statistical analysis of phenotypic and functional markers, and Treg/NK-like T cell subsets during 24 h restimulation.

p-values from two-way repeated measures ANOVA with Tukey's multiple comparisons test (p = 0.05), comparing the expression activation, checkpoint, apoptotic, senescence, cytotoxic, proliferative, and metabolic markers (CD25, CD69, HLA-I, HLA-II, PD1, TIM3, LAG3, CD36, active caspase 3, CD57, granzyme B, perforin, Hif-1a, GLUT1, Ki-67), as well as Treg and NK-like T cell subsets in CAR^+ and CAR^- compartments across 0 h, 3 h, 12 h, 15 h, and 24 h. Data are presented separately for day 5 and day 10 products (n = 3 donors). "NA" denotes comparisons with no significant difference between timepoints.

			Activation, Checkpoint, Apoptotic & Senescence Markers										Cytotoxic Function		Metabolic & Proliferative Markers			Treg & NK T		
Timepoint	CAR+/-	Comparison	CD25	CD69	HLA-I	HLA-II	PD1	TIM3	LAG3	CD36	Active Caspase 3	CD57	Granzyme B	Perforin	Hif-1a	GLUT1	Ki-67	CD8+ Treg	CD4+ Treg	NK T
Day 5	CAR+	0h (baseline) vs. 3h (co-culture)	>0.9999	0.0201	0.0692	0.3612	0.0419	0.8314	0.0270	0.2762	0.2117	0.7201	0.9966	0.1679	0.7002	0.1166	0.2047	0.7422	0.9349	0.9210
		0h (baseline) vs. 12h (+9h co-culture)	0.1294	0.3340	0.1207	0.9947	0.0210	0.0359	0.0637	0.0514	0.4670	0.4787	0.0579	0.1523	0.0753	0.1351	0.6280	0.6308	0.1066	0.7628
		0h (baseline) vs. 15h (+3h restim)	0.1294	0.0140	0.0536	0.7248	0.2749	0.2370	0.0613	0.0477	0.1229	0.9740	0.0475	0.6701	0.4083	0.0171	0.2377	0.5860	0.2595	0.9818
		0h (baseline) vs. 24h (+12h restim)	0.1294	0.0405	0.9856	0.3585	0.0706	0.9995	0.0965	0.0154	0.2094	0.9937	0.1961	0.4632	<0.0001	0.0100	0.2827	0.5595	0.1644	0.9737
		0h (baseline) vs. 24h (n.r.)	0.9996	0.7840	0.3790	0.0516	0.2214	0.1970	0.0302	0.3548	0.0591	0.7497	0.1062	0.1466	0.9741	0.0582	0.4386	0.3102	0.2509	0.9985
		3h (co-culture) vs. 12h (+9h co-culture)	0.5647	0.5257	0.8866	0.9736	0.6885	0.4591	0.0957	0.0363	0.5117	0.7748	0.0324	<0.0001	0.9778	0.1625	0.3476	0.6008	0.1052	0.6207
		3h (co-culture) vs. 15h (+3h restim)	0.5647	0.0037	0.8866	0.9770	0.9412	0.8471	0.0786	0.0694	0.1727	0.8621	0.0095	0.0522	0.9989	0.0245	0.0899	0.5451	0.3554	0.9992
		3h (co-culture) vs. 24h (+12h restim)	0.5647	>0.9999	0.7616	0.4491	0.9729	0.9787	0.1476	0.0162	0.2149	>0.9999	0.1799	0.0825	0.3268	0.0130	0.0938	0.4825	0.2581	0.8245
		3h (co-culture) vs. 24h (n.r.)	0.9997	0.0024	0.2219	0.9990	0.0808	0.0191	0.0740	0.8517	0.0283	0.6320	0.0324	0.0458	0.7962	0.0484	0.0923	0.1713	0.3063	0.7748
		12h (+9h co-culture) vs. 15h (+3h restim)	NA	0.1693	0.8866	0.9664	>0.9999	0.3457	0.9896	0.9133	0.5935	0.6802	0.0688	0.4185	0.0156	0.7941	0.9999	0.3720	0.8657	0.4776
		12h (+9h co-culture) vs. 24h (+12h restim)	NA	0.4056	0.5753	0.9228	0.2092	0.3283	0.5166	0.9999	0.9167	0.9999	0.2977	0.9678	0.0464	0.5023	0.8414	0.9524	0.9314	0.4542
		12h (+9h co-culture) vs. 24h (n.r.)	0.7748	0.2941	0.1979	0.8896	0.0079	0.6976	0.5614	0.3086	0.6785	0.3947	0.0428	>0.9999	0.0082	0.0587	0.9829	0.3586	0.5110	0.6142
		15h (+3h restim) vs. 24h (+12h restim)	NA	0.0272	0.6688	0.9995	0.2166	0.8028	0.5792	0.9956	0.2388	0.5651	0.2358	0.6379	0.0335	0.0448	0.3926	0.6559	0.7703	0.5244
		15h (+3h restim) vs. 24h (n.r.)	0.7748	0.0013	0.2003	0.9951	0.0427	0.2749	0.6731	0.1293	0.9886	0.9996	0.1677	0.5428	0.0240	0.5312	0.0852	0.9868	0.2407	0.8705
		24h (+12h restim) vs. 24h (n.r.)	0.7748	0.0050	0.9040	0.9803	0.0088	0.3721	0.9270	0.1994	0.2759	0.3947	0.2185	0.8049	0.0174	0.8602	0.1730	0.0118	0.3689	0.8333
	CAR-	0h (baseline) vs. 3h (co-culture)	0.8233	0.8291	0.9725	0.9331	0.9996	0.8328	0.1287	0.9992	0.7518	0.9220	0.6812	0.1001	0.6898	0.9857	0.1003	0.8765	0.7160	0.8899
		0h (baseline) vs. 12h (+9h co-culture)	0.4672	0.4698	0.2387	0.1514	0.1114	0.0438	0.0279	0.2684	0.5367	0.9997	0.0695	0.0165	0.1108	0.7884	0.9055	0.5911	0.2245	0.8372
		0h (baseline) vs. 15h (+3h restim)	0.0748	0.1315	0.2177	0.1717	0.0687	0.1008	0.0225	0.6035	0.1169	0.9920	0.2022	0.0133	0.5874	0.7648	0.2389	0.3906	0.0164	<0.0001
		0h (baseline) vs. 24h (+12h restim)	0.3371	0.9864	0.8866	0.8103	0.0265	0.4409	0.2100	0.1723	0.3724	0.9897	0.2840	0.6157	0.0089	0.9751	0.4306	0.3578	0.5191	0.9922
		0h (baseline) vs. 24h (n.r.)	0.6552	0.1276	0.0768	0.9486	0.0764	0.4553	0.2517	0.6408	0.3342	0.0003	0.2470	0.3237	0.9288	0.9038	0.9939	0.1797	0.3503	0.0912
		3h (co-culture) vs. 12h (+9h co-culture)	>0.9999	0.0406	0.0618	0.0298	0.1077	0.0946	0.0338	0.2984	0.4567	0.8801	0.0204	<0.0001	0.9079	0.5985	0.6707	0.8519	0.7245	0.0311
		3h (co-culture) vs. 15h (+3h restim)	0.0780	0.2094	0.3579	0.0923	0.1613	0.1934	0.0310	0.6232	0.1891	0.8917	0.0125	0.0012	0.9988	0.5315	0.0269	0.7081	0.2525	0.9565
		3h (co-culture) vs. 24h (+12h restim)	0.3161	0.1601	0.9921	0.6330	0.0792	0.3392	0.4866	0.1085	0.2714	0.8920	0.1778	0.2061	0.4991	0.9259	0.1169	0.8906	0.9574	0.2984
		3h (co-culture) vs. 24h (n.r.)	0.7004	0.0100	0.2250	0.7621	0.0169	0.4102	0.3661	0.6687	0.1769	0.5549	0.0563	0.1167	0.7638	0.8250	0.1820	0.3908	0.2499	0.5526
		12h (+9h co-culture) vs. 15h (+3h restim)	0.0622	0.0925	0.3693	>0.9999	0.4218	0.2628	0.3143	>0.9999	0.8755	0.9942	0.0274	0.0803	<0.0001	0.8940	0.9953	0.9946	0.9934	0.7566
		12h (+9h co-culture) vs. 24h (+12h restim)	0.3513	0.4009	0.2887	0.9991	0.0928	0.2715	0.0080	0.5359	0.0205	0.9962	0.9945	0.8916	0.3118	0.8545	0.2926	0.8129	0.1868	0.7616
		12h (+9h co-culture) vs. 24h (n.r.)	0.8433	0.4180	0.0706	0.8909	0.1006	0.1385	0.5183	0.0256	>0.9999	0.0612	0.0008	>0.9999	0.0066	0.9983	0.8274	0.2001	0.9992	>0.9999
		15h (+3h restim) vs. 24h (+12h restim)	0.7348	0.1192	0.2706	0.9988	0.5756	0.9892	0.0067	0.8470	0.5909	0.9938	0.3287	0.6678	0.0557	0.9945	0.2280	0.4445	0.0692	0.9513
		15h (+3h restim) vs. 24h (n.r.)	0.2431	0.0151	0.0629	0.8887	0.8058	0.9433	0.6689	0.5809	0.6516	0.6259	0.3701	0.9576	0.0476	0.9910	0.5056	0.2237	>0.9999	0.0659
		24h (+12h restim) vs. 24h (n.r.)	0.1933	0.1365	0.1252	0.7679	0.9969	0.9046	0.2959	0.9916	0.8036	0.1213	0.3340	0.4496	0.0098	0.3599	0.0337	0.1749	0.5342	0.9161
Day 10	CAR+	0h (baseline) vs. 3h (co-culture)	0.8814	0.0121	0.3865	0.5402	0.0024	0.1604	0.3830	0.2311	0.7175	0.9821	0.8381	0.1673	0.7552	0.3038	0.9996	0.3947	0.2181	0.5364
		0h (baseline) vs. 12h (+9h co-culture)	0.8431	0.0402	0.3258	0.4805	0.0558	0.1729	0.2041	0.0896	0.3177	0.2227	0.0875	0.9920	0.1986	0.0334	0.0086	0.2404	0.1870	0.2364
		0h (baseline) vs. 15h (+3h restim)	0.6506	0.0085	0.3560	0.0642	0.0404	0.9425	0.1373	0.2059	0.2456	0.3871	0.2072	0.5573	0.4903	<0.0001	0.4082	0.0690	0.1562	0.2234
		0h (baseline) vs. 24h (+12h restim)	0.6609	0.0123	0.3148	0.0077	0.0101	0.7699	0.0082	0.1709	0.7806	0.5385	0.0362	0.9996	0.1456	0.0254	0.5268	0.0877	0.1934	0.6174
		0h (baseline) vs. 24h (n.r.)	0.7474	0.0300	0.3894	0.1348	0.0433	0.3951	0.1105	0.0104	0.0061	0.3721	0.0089	0.9817	0.9864	0.0192	0.1843	0.1364	0.0453	0.7322
		3h (co-culture) vs. 12h (+9h co-culture)	0.7637	0.9611	0.2113	0.6421	0.1128	0.8792	0.1863	0.0892	0.2760	0.5090	0.1473	0.0506	0.6039	0.0560	0.5868	0.9917	0.9331	0.1816
		3h (co-culture) vs. 15h (+3h restim)	0.3172	0.0562	0.3748	0.9996	0.0573	0.4566	0.1193	0.2075	0.4448	0.6639	0.0697	0.0545	0.9106	0.0067	0.7760	>0.9999	0.5715	0.6919
		3h (co-culture) vs. 24h (+12h restim)	0.3410	0.0596	0.1919	0.9844	0.0113	0.0593	0.0002	0.1967	0.8346	0.5227	0.0672	0.0528	0.3409	0.0403	0.9419	>0.9999	0.8745	0.6681
		3h (co-culture) vs. 24h (n.r.)	0.5128	0.8415	0.7452	0.9777	0.0586	>0.9999	0.0954	0.0207	0.0450	0.9292	0.1261	0.0437	0.9236	0.0422	0.5427	0.1266	0.0681	0.8874
		12h (+9h co-culture) vs. 15h (+3h restim)	0.5111	0.3180	0.8866	0.0161	0.0976	0.2522	0.2088	0.3875	0.7866	0.6267	0.9346	0.1864	0.4031	0.0194	0.3347	0.9945	0.6085	0.0747
		12h (+9h co-culture) vs. 24h (+12h restim)	0.5996	0.3735	0.5302	0.6117	0.0158	0.1061	0.7003	0.3677	0.1544	>0.9999	0.1160	0.1985	0.1166	0.0322	0.1103	0.9998	0.9965	0.4318
		12h (+9h co-culture) vs. 24h (n.r.)	0.9514	0.9995	0.3999	0.5324	0.0472	0.9568	0.1657	0.7059	0.8649	0.1501	0.3925	>0.9999	0.0807	0.1155	0.0728	0.2329	0.0178	0.4774
		15h (+3h restim) vs. 24h (+12h restim)	0.3901	0.2193	0.6977	0.3095	0.0063	0.8656	0.9374	0.9772	0.9142	0.9606	0.1022	0.1866	0.0131	0.8093	0.0171	0.8687	0.2492	0.8470
		15h (+3h restim) vs. 24h (n.r.)	0.0070	0.2560	0.2400	0.2535	0.3473	0.6388	0.1387	0.9076	0.6625	0.6436	0.3822	0.5102	0.0377	0.4521	0.1346	0.1788	0.0056	0.9920
		24h (+12h restim) vs. 24h (n.r.)	0.0193	0.2751	0.6177	>0.9999	0.0444	0.2351	>0.9999	0.6565	0.4233	0.8312	0.0818	0.9586	0.0088	0.9593	0.3469	0.1770	0.0073	0.3024
	CAR-	0h (baseline) vs. 3h (co-culture)	<0.0001	0.1864	0.7352	0.6180	0.4526	0.2149	0.7407	0.6230	0.8495	>0.9999	0.7418	0.3770	0.9584	>0.9999	>0.9999	0.6852	0.6901	0.9123
		0h (baseline) vs. 12h (+9h co-culture)	0.1593	0.6363	0.4324	0.5959	0.0390	0.3699	0.1438	0.2378	0.2036	0.9999	>0.9999	>0.9999	0.2645	0.3973	0.8390	0.6884	0.5548	0.4577
		0h (baseline) vs. 15h (+3h restim)	0.1619	0.0272	0.3896	>0.9999	>0.9999	0.3803	0.1578	0.4672	0.1416	0.8351	0.9690	0.9961	0.9641	0.9955	0.3335	0.6909	0.4485	0.7185
		0h (baseline) vs. 24h (+12h restim)	0.0702	0.0217	0.6464	0.4790	0.4647	0.9998	0.2357	0.6263	0.2015	0.9918	0.9982	0.9996	0.2779	0.0644	0.0156	0.8294	0.5977	0.3954
		0h (baseline) vs. 24h (n.r.)	0.0719	0.0254	0.5809	0.2283	0.0406	>0.9999	0.2593	0.2625	0.1465	0.2523	0.9537	0.9999	0.6510	0.4800	0.6587	0.8096	0.3873	0.4760
		3h (co-culture) vs. 12h (+9h co-culture)	0.2453	0.2504	0.1145	0.2716	0.0112	0.3579	0.1318	0.2092	0.2168	>0.9999	0.3521	0.3122	0.7634	0.1547	0.9144	0.8419	0.3912	0.7121
		3h (co-culture) vs. 15h (+3h restim)	0.2498	>0.9999	0.0416	0.6358	0.9947	0.1109	0.1793	0.4655	0.3107	0.7587	0.4891	0.1512	>0.9999	0.9887	0.5229	0.7977	0.2542	0.7773
		3h (co-culture) vs. 24h (+12h restim)	0.0991	0.6209	0.3029	0.1515	0.2375	0.2962	0.4420	0.6251	0.1234	0.8689	0.2978	0.6463	0.7858	0.1734	0.0691	0.9766	0.9384	0.7778
		3h (co-culture) vs. 24h (n.r.)	0.0959	0.3395	0.5534	0.4129	0.0141	0.0730	0.3602	0.2837	0.1602	0.1374	0.3380	0.3217	0.7372	0.4412	0.7927	0.9403	0.7767	0.8686
		12h (+9h co-culture) vs. 15h (+3h restim)	>0.9999	0.2934	0.9995	0.4813	0.2168	>0.9999	0.4683	0.5938	0.3184	0.6977	0.0079	0.3819	0.1761	0.7707	0.9982	0.7359	0.5868	0.7046
		12h (+9h co-culture) vs. 24h (+12h restim)	0.9536	0.8511	0.3082	0.9998	0.8557	0.4770	0.9982	0.8844	0.3449	0.8767	0.2020	0.9267	0.9687	0.6266	0.5602	>0.9999	0.5401	0.9016
		12h (+9h co-culture) vs. 24h (n.r.)	0.7026	0.9978	0.1122	0.7764	0.0455	0.5416	0.9870	0.9997	0.5977	0.3027	0.3733	0.9839	0.0696	0.9995	0.8329	0.9831	0.9511	0.9986
		15h (+3h restim) vs. 24h (+12h restim)	0.1601	0.0999	0.1769	0.3362	0.0331	0.7744	0.8435	0.6331	0.9998	0.6000	0.0744	0.7239	0.0851	0.0887	0.4054	0.3598	0.2719	0.7798
		15h (+3h restim) vs. 24h (n.r.)	0.0053	0.0977	0.0389	0.9893	0.0921	0.3535	0.9940	0.5690	0.3041	0.1198	0.1917	0.6818	0.0283	0.0134	>0.9999	0.9998	0.7202	>0.9999
		24h (+12h restim) vs. 24h (n.r.)	0.3745	0.1694	>0.9999	0.6819	0.2743	0.9989	0.8111	0.7769	0.2307	0.3023	0.6439	0.9984	0.0398	0.5957	0.5051	0.7722	0.6489	0.3542

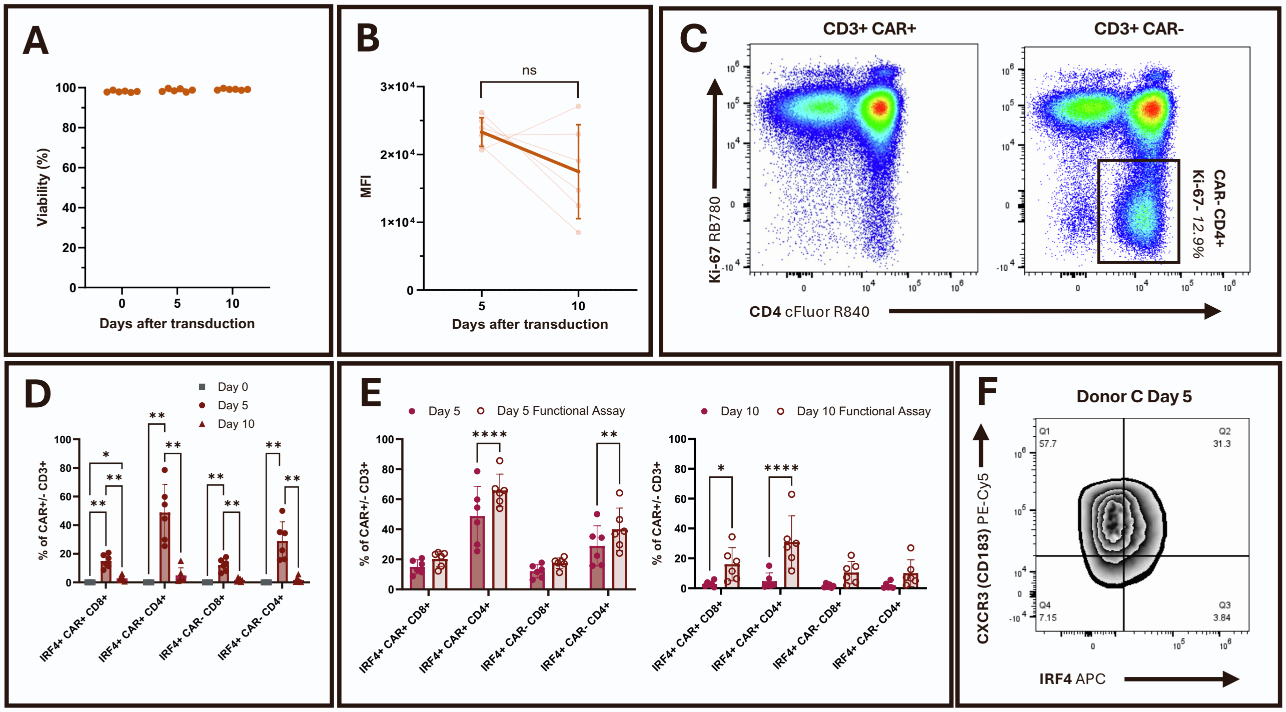

Figure S1. CAR T cell viability and subset-specific proliferation.

(**A**) Viability of $CD3^+$ T cells on days 0, 5, and 10 of culture (n = 6 donors, two independent experiments). (**B**) MFI of CAR in total $CD3^+$ T cells at days 5 and 10 (n = 6 donors, two independent experiments). (**C**) Representative flow cytometry plots showing Ki-67 and CD4 expression in $CD3^+CAR^+$ and $CD3^+CAR^-$ cells at day 5 (n = 1 donor). (**D**) Frequencies of $IRF4^+$ T cell subsets within CAR^+CD4^+, CAR^+CD8^+, CAR^-CD4^+, and CAR^-CD8^+ populations on days 0, 5, and 10 (n = 6 donors, two independent experiments). (**E**) Frequencies of $IRF4^+$ subsets following 3 h co-culture at days 5 and 10 in CAR^+ and CAR^-, $CD4^+$ and $CD8^+$ compartments (n = 6 donors, two independent experiments). (**F**) Contour plot showing IRF4 and CXCR3 co-expression in $CD3^+CAR^+$ T cells from a representative day 5 donor (n = 1 donor). Statistical analyses were performed using a paired, two-tailed Student's t-test (B), two-way repeated measures ANOVA with Tukey's (D) or Šídák's (E) multiple comparisons test. Data represent mean ± s.d.; *$p < 0.05$; **$p < 0.01$; ****$p < 0.0001$; n.s., not significant. Absence of p-values indicates non-significance at the $p = 0.05$ threshold.

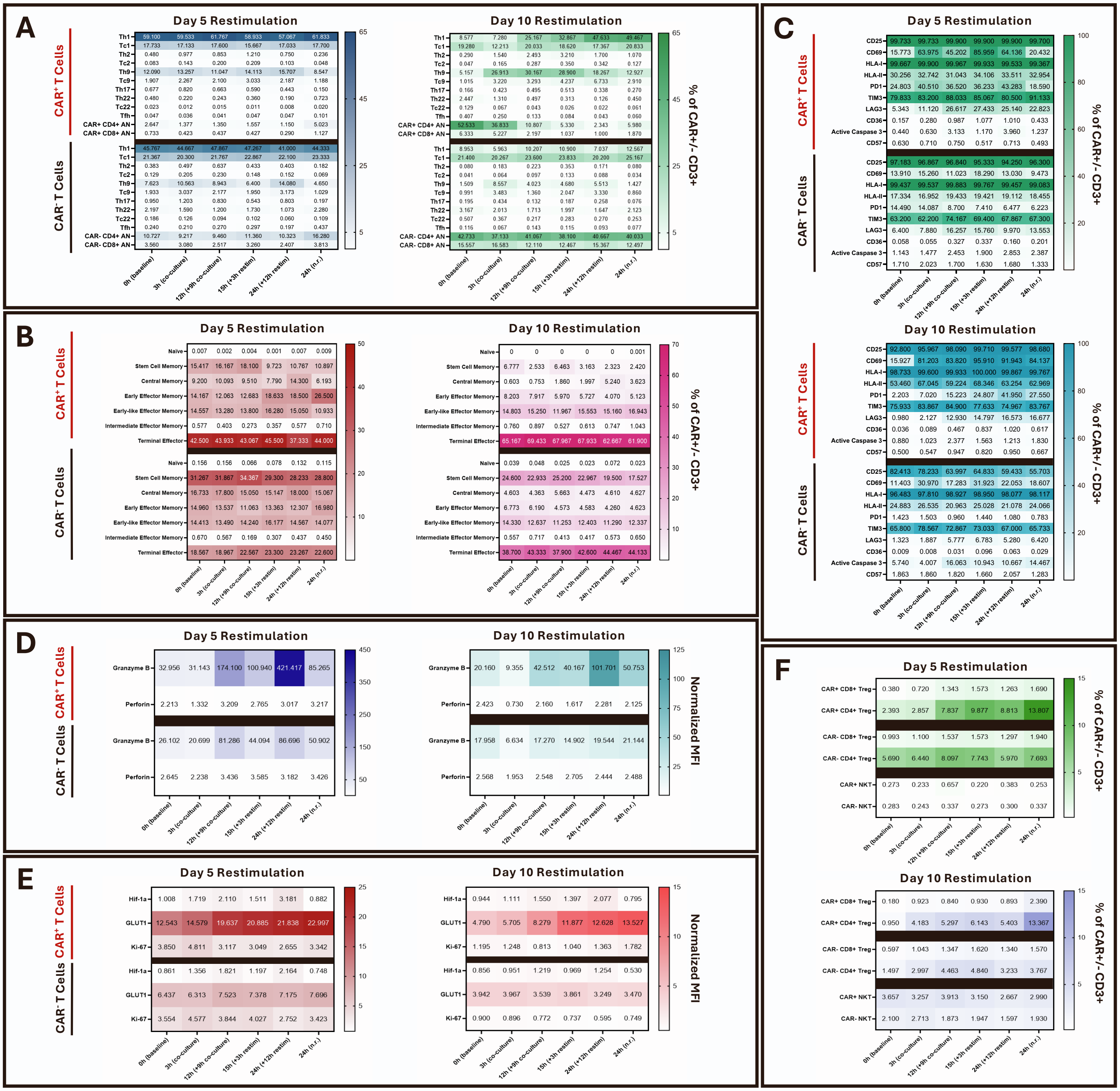

Figure S2. Heatmap of CAR$^+$ and CAR$^-$ T cell profiling during 24 h restimulation.

(**A**) Frequencies of CD4$^+$ helper (Th) and CD8$^+$ cytotoxic (Tc) subsets within CAR$^+$ and CAR$^-$ compartments across the 24 h restimulation assay, shown for day 5 (left) and day 10 (right) products (*n* = 3 donors). Only subsets representing ≥0.5% of total CD3$^+$ T cells at any time point or in any donor are shown. AN, "all negative" (CXCR3$^-$IRF4$^-$CRTH2$^-$CCR6$^-$CXCR5$^-$). (**B**) Distribution of memory and differentiation subsets (Naïve, Stem Cell Memory, Central Memory, Early Effector Memory, Early-like Effector Memory, Intermediate Effector Memory, Terminal Effector) within CAR$^+$ and CAR$^-$ T cells (*n* = 3 donors). (**C**) Frequencies of activation, checkpoint, apoptotic, and senescence markers (CD25, CD69, HLA-I and -II, PD1, TIM3, LAG3, CD36, active caspase 3, CD57) within CAR$^+$ and CAR$^-$ T cells (*n* = 3 donors). (**D**) Normalized MFI of granzyme B and perforin in CAR$^+$ and CAR$^-$ T cells (*n* = 3 donors). (**E**) Normalized MFI of Hif-1a, GLUT1, and Ki-67 in CAR$^+$ and CAR$^-$ T cells (*n* = 3 donors). (**F**) Frequencies of Tregs in CAR$^+$CD4$^+$, CAR$^+$CD8$^+$, CAR$^-$CD4$^+$, and CAR$^-$CD8$^+$ compartments, and NK-like T cell subsets in CAR$^+$ and CAR$^-$ T cells (*n* = 3 donors). Statistical analyses were performed using two-way repeated measures ANOVA with Tukey's multiple comparisons test (Tables S6 and S7). Data represent mean values from three healthy donors. n.r., non-restimulated; restim, restimulated.

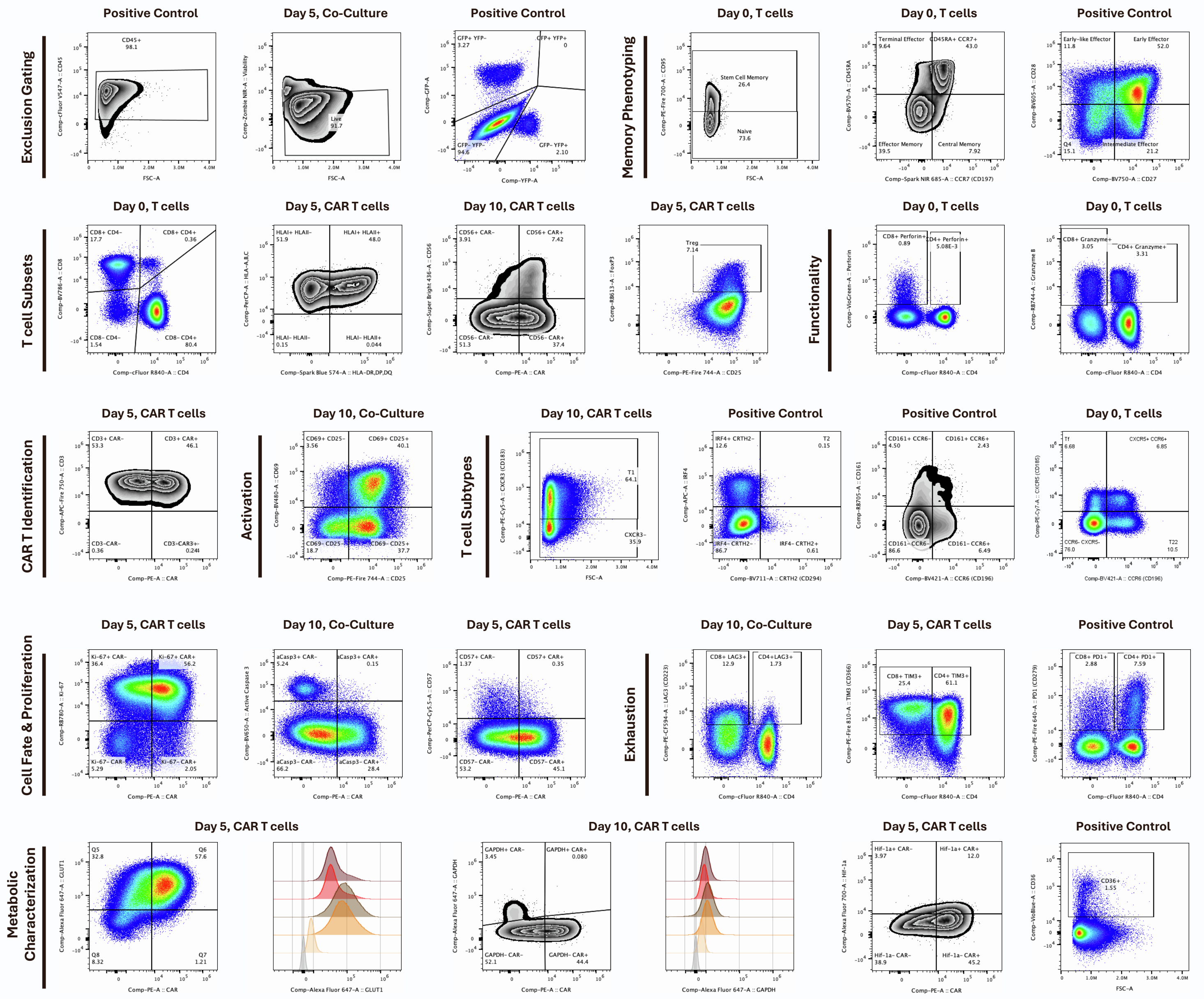

Figure S3. Representative positive controls for all major CAR T cell marker categories.

(**Top row**) CD45 lineage, viability, and YFP$^-$/GFP$^-$ exclusion gating were applied to isolate viable CD3$^+$ T cells. Memory subsets were defined using CD45RA, CCR7, CD27, CD28, and CD95. (**Second row**) CD4$^+$ and CD8$^+$ subsets were classified using CD3, CD4 and CD8. HLA-A, B, C and HLA-DP, DQ, DR were used to assess HLA knockout efficiency in allogeneic products. CD3$^+$CD56$^+$ cells were gated as NK-like T cells, and Tregs were identified as CD25$^+$FoxP3$^+$, example shown as CD4$^+$CD25$^+$FoxP3$^+$. Functional markers included intracellular granzyme B and perforin. (**Third row**) CAR$^+$ and CAR$^-$ populations were defined using anti-CAR staining and CD3 expression. Activation markers (CD25, CD69) were assessed in antigen-stimulated cells. Th and Tc subsets were phenotyped based on CXCR3, CRTH2, IRF4, CD161, CCR6, and CXCR5. (**Fourth row**) Proliferation was assessed by intracellular Ki-67 staining, apoptosis by active caspase 3, and senescence by extracellular CD57 staining. Exhaustion profiling included LAG3, TIM3, and PD1. (**Bottom row**) Metabolic profiling included GLUT1, GAPDH, Hif-1a, and CD36. Histograms illustrate fluorescence resolution for GLUT1 and GAPDH across unstained, day 0, day 5, day 5 co-culture, day 10, and day 10 co-culture conditions. Fluorescence intensities are shown as normalized counts. All gates were defined using FMO, biological negative controls, or validated positive controls. Plot titles indicate the sample source.

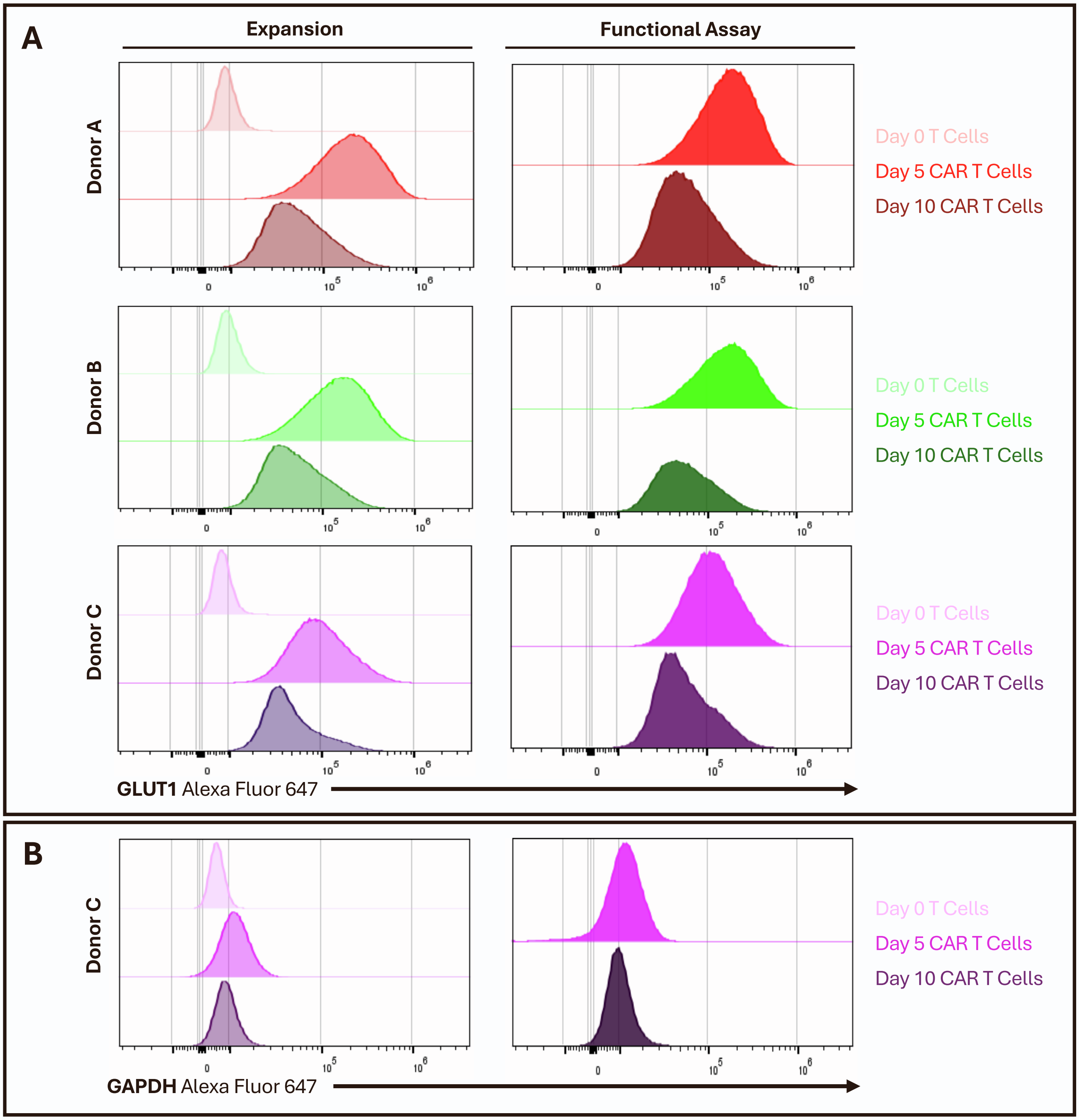

Figure S4. Switchable GLUT1 and GAPDH staining enables modular metabolic profiling of CAR T cells.

(**A**) Histogram overlays showing GLUT1 expression (Alexa Fluor 647) in three healthy donors (Donors A–C), measured at day 0, day 5, and day 10 (left), and after 3 h co-culture (right). (**B**) Histogram overlays for GAPDH expression (Alexa Fluor 647) from Donor C under the same conditions. Fluorescence intensities are shown as normalized counts. A darker color scheme (right) distinguishes co-culture (functional assay) histograms from expansion histograms.

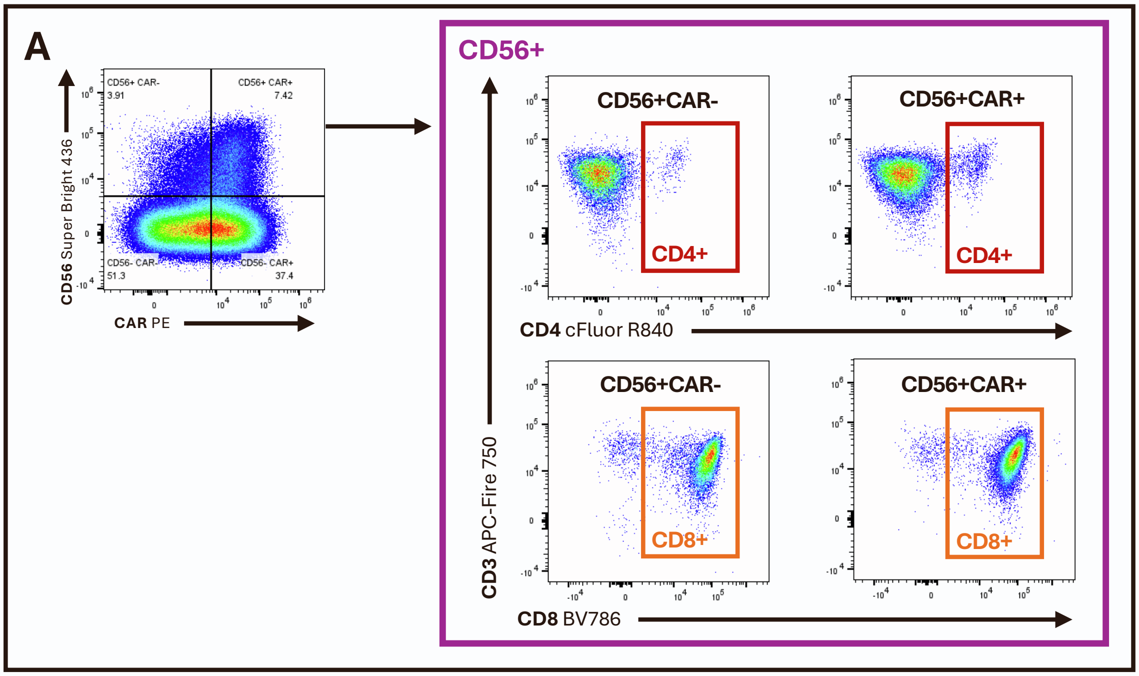

Figure S5. CD56$^+$ CAR$^+$ T cell enrichment within CD8$^+$ compartment.

(**A**) Representative gating of CD56$^+$CAR$^+$ and CAR$^-$ T cells from a day 10 sample. Plots show CD56$^+$ subsets based on CAR, CD4, and CD8 expression. CD56$^+$CAR$^+$ cells were predominantly localized within the CD8$^+$ compartment, indicating that CD56 expression in CAR$^+$ T cells was largely restricted to CD8$^+$CD3$^+$ cells. Data are representative of one donor (n = 1).

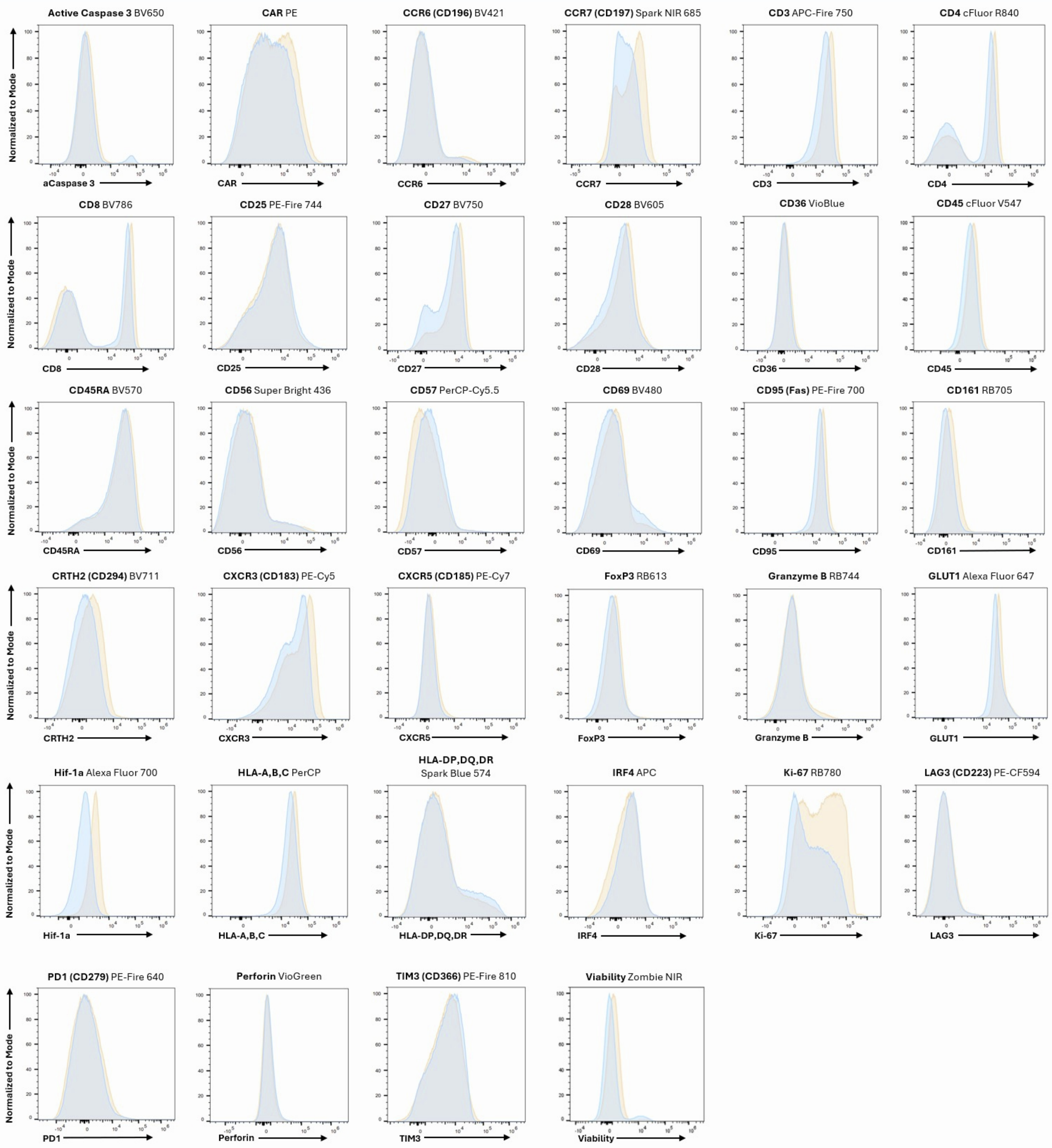

Figure S6. Histogram overlays of fresh versus cryopreserved cells.

Overlay plots showing normalized expression profiles of 34 spectral cytometry markers in day 10 cells under fresh (**orange**) and cryopreserved (**blue**) conditions. Fluorescence intensities are shown as normalized counts. Viability Zombie NIR was gated on single cells, and CD45 was gated on GFP^{-}YFP^{-}. All other markers were gated on CD45^{+}.

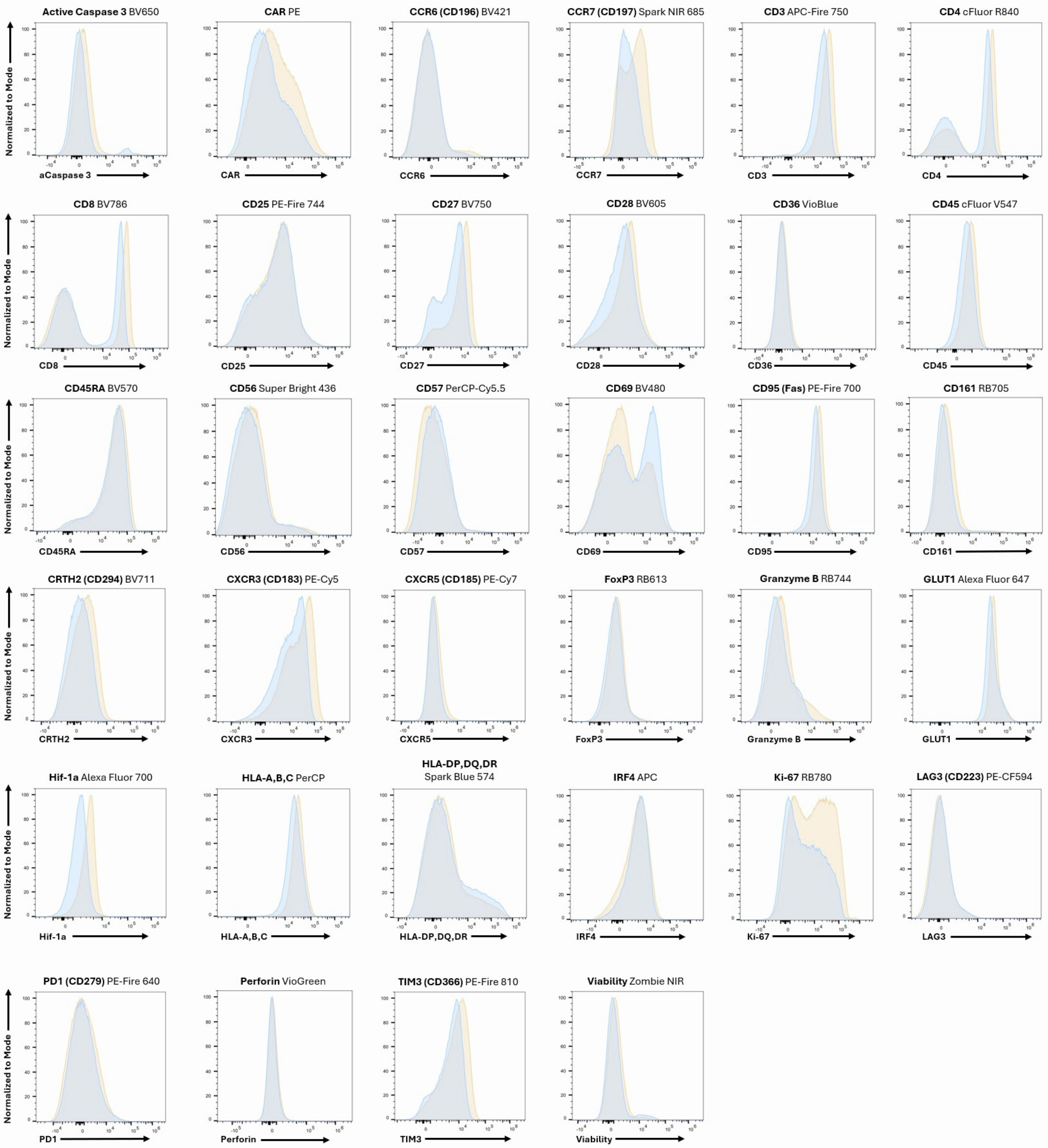

Figure S7. Marker expression in fresh versus cryopreserved cells following 3 h co-culture.

Histogram overlays comparing 34 spectral cytometry markers in day 10 cells under fresh (**orange**) and cryopreserved (**blue**) conditions after 3 h co-culture with $CD19^+$ Nalm6-YFP^+ target and $CD19^-$ Nalm6-GFP^+ control cells. Fluorescence intensities are shown as normalized counts. GFP and YFP were excluded from analysis, as they were introduced post-thaw. Viability Zombie NIR was gated on single cells, and CD45 was gated on GFP^- YFP^-. All other markers were gated on $CD45^+$.

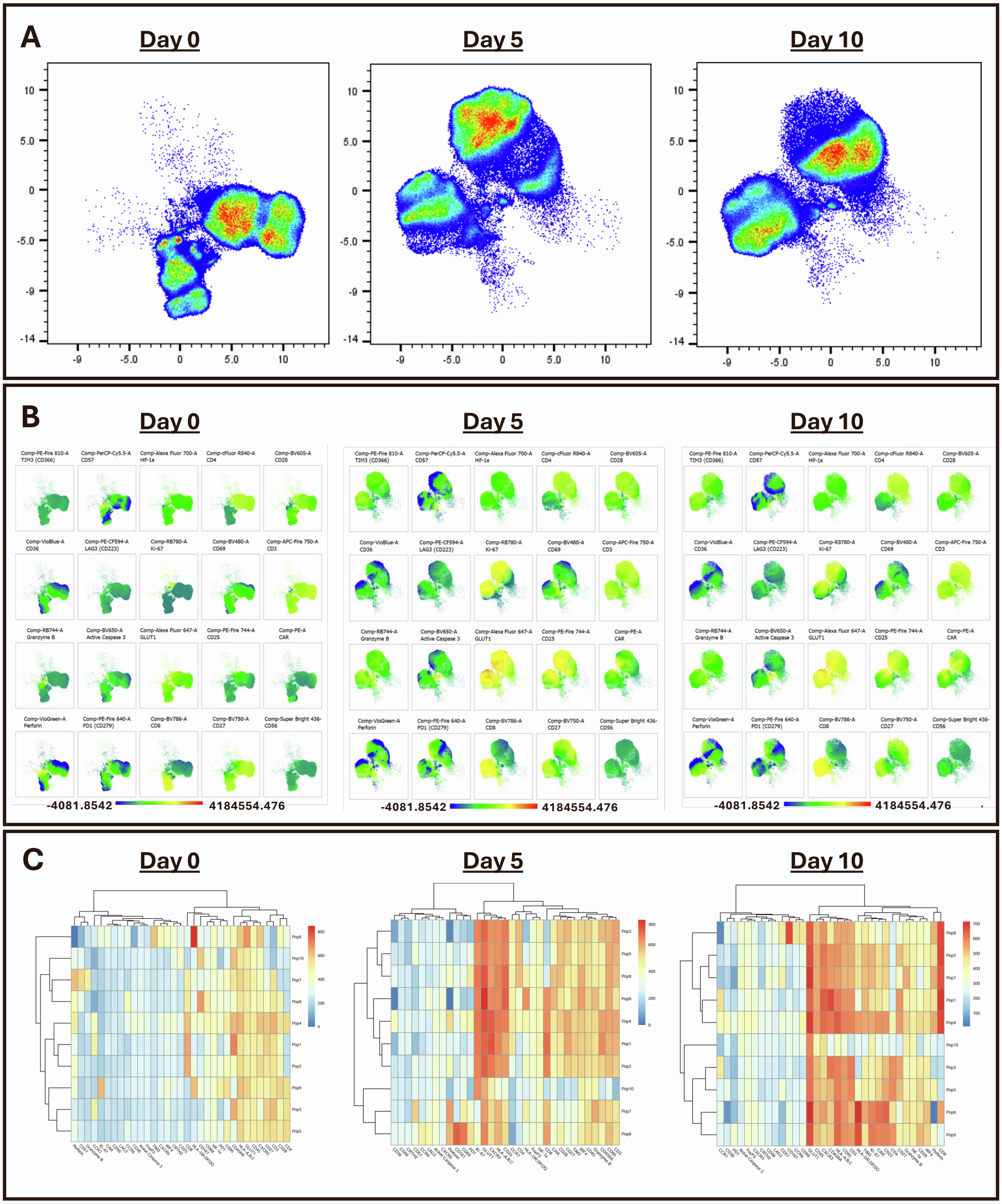

Figure S8. UMAP clustering across manufacturing timepoints.

(**A**) UMAP clustering of CD45$^+$ gated cells at day 0, day 5, and day 10, illustrating population shifts during expansion (*n* = 6 donors, two independent experiments). (**B**) UMAP marker overlay plots showing relative expression intensities of selected surface and intracellular markers across all timepoints (*n* = 6 donors, two independent experiments). (**C**) Hierarchically clustered heatmaps displaying MFI values for each marker, highlighting temporal changes in expression. Data represent concatenated and downsampled events (*n* = 6 donors, two independent experiments).

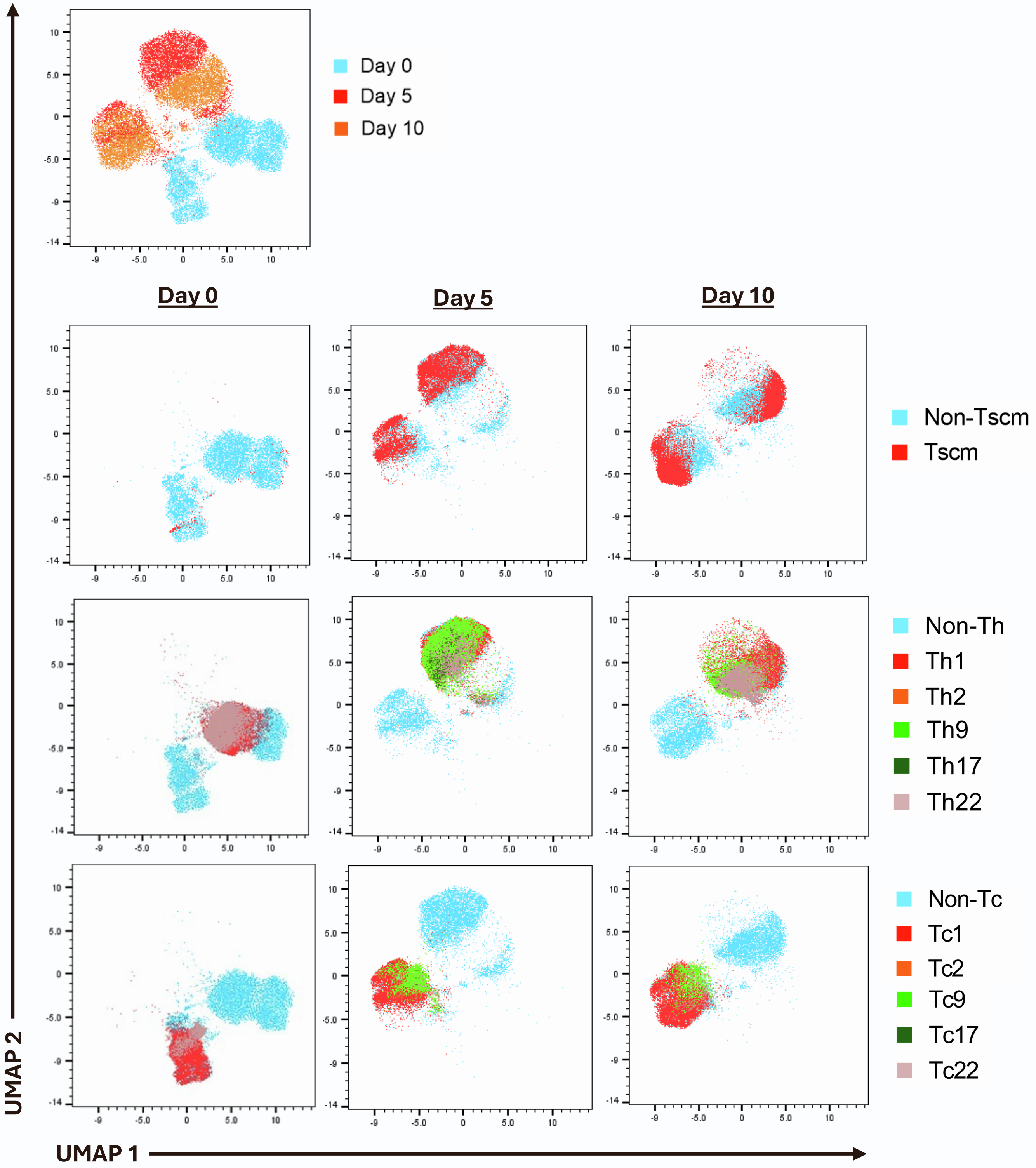

Figure S9. UMAP-based projection of T cell subset localization across manufacturing timepoints.

UMAP overlays showing the distribution of major T cell subsets within the clustered CD45$^+$ population at day 0, day 5, and day 10 (*n* = 6 donors, two independent experiments). **Top row**: Combined UMAP illustrating global shifts in phenotypes during expansion. **Subsequent rows**: Subset-specific overlays highlighting stem cell memory (T_{SCM}), CD4$^+$ helper subsets (Th1, Th2, Th9, Th17, Th22), and CD8$^+$ cytotoxic subsets (Tc1, Tc2, Tc9, Tc17, Tc22), with distinct localization and temporal remodeling. Each dot represents a single cell, colored by subset identity; blue populations represent cells that do not fall under an annotated subset identity.

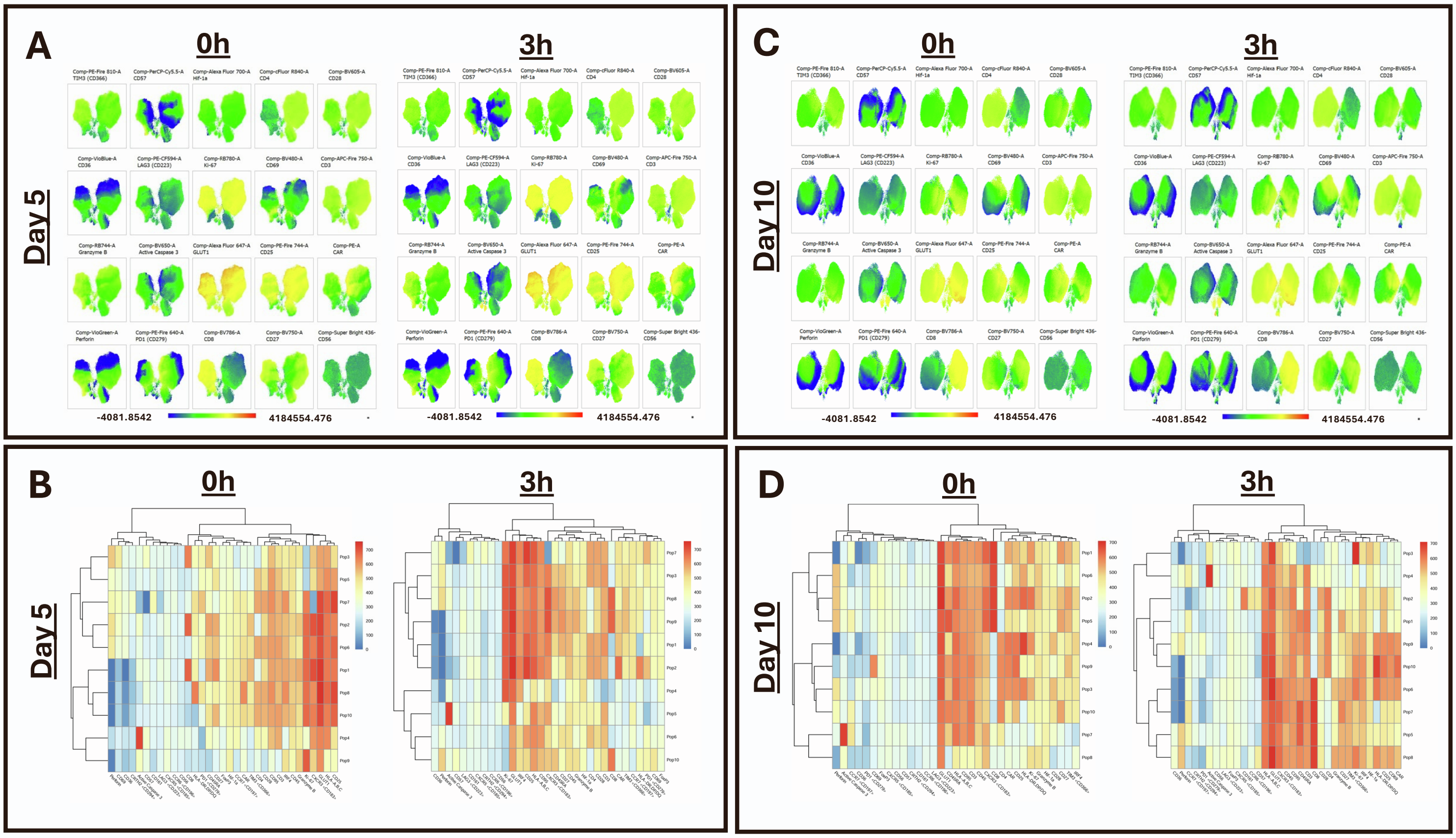

Figure S10. UMAP clustering of day 5 and day 10 cells at baseline and after 3 h co-culture.

(**A, C**) UMAP overlays of clustered $CD45^+$ gated cells at day 5 (A) and day 10 (C), comparing baseline (0 h) and 3 h co-culture with $CD19^+$ Nalm6-YFP^+ target and $CD19^-$ Nalm6-GFP^+ control cells. Expression patterns are shown for selected lineage, activation, checkpoint, metabolic, and functional markers (*n* = 6 donors, two independent experiments). (**B, D**) Heatmaps displaying normalized MFI of markers at day 5 (B) and day 10 (D), comparing 0 h (left) and 3 h (right) conditions (*n* = 6 donors, two independent experiments).

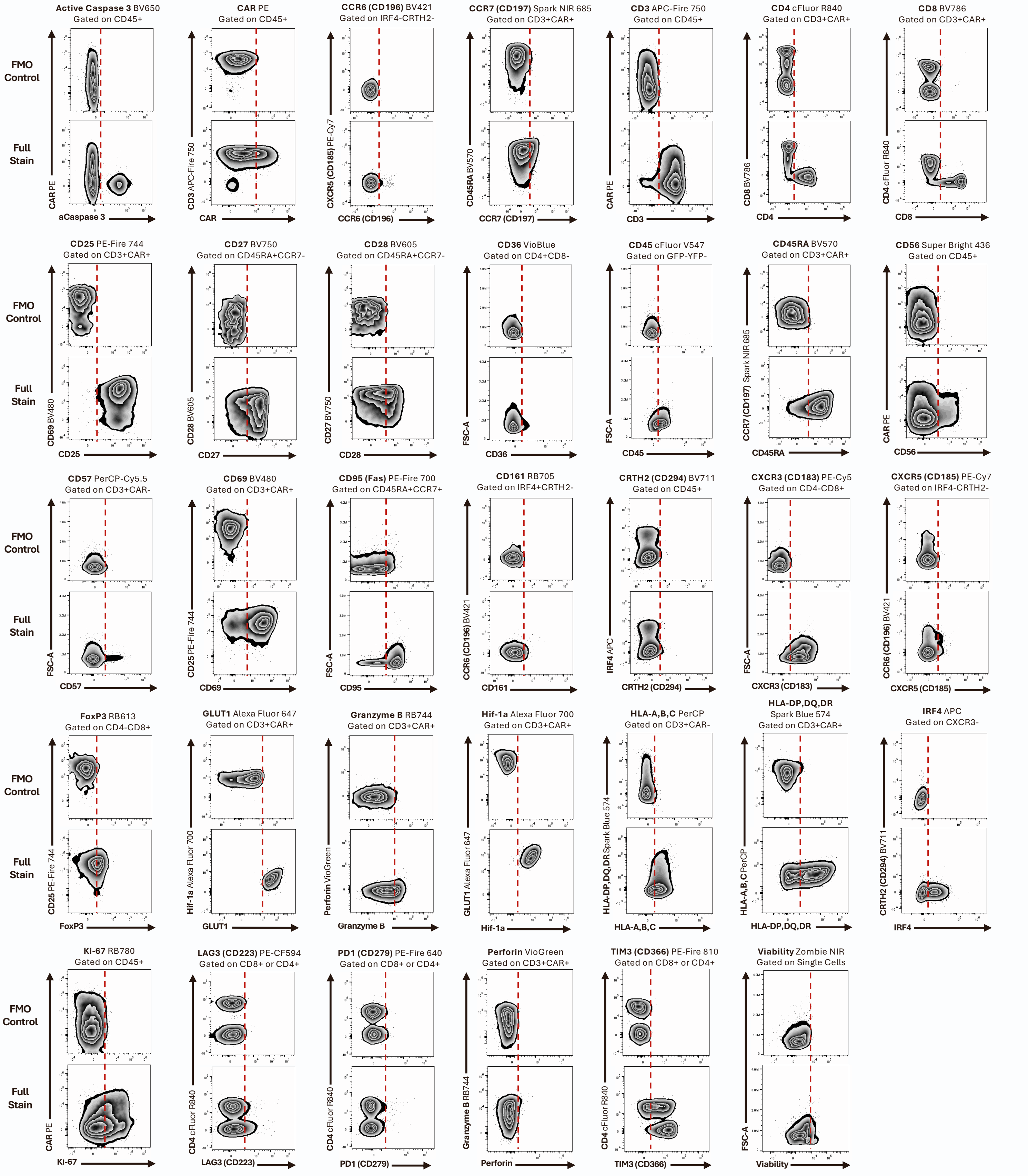

Figure S11. Fluorescence-minus-one (FMO) controls for spectral cytometry panel validation.

Representative contour plots comparing FMO controls (**top rows**) with fully stained samples (**bottom rows**) for each of the 34 antibody markers used in the spectral panel. Gating thresholds (red dashed lines) were defined using FMO controls and applied to fully stained samples for consistent marker quantification. Gates were drawn within biologically relevant parent populations, as indicated on each plot (e.g. Gated on CD3$^+$CAR$^+$, CD45$^+$). Data shown are from CAR$^+$ T cells following 3 h co-culture or from composite samples generated by pooling PBMCs, gene-edited CAR T cells, non-edited T cells, and activated T cells.

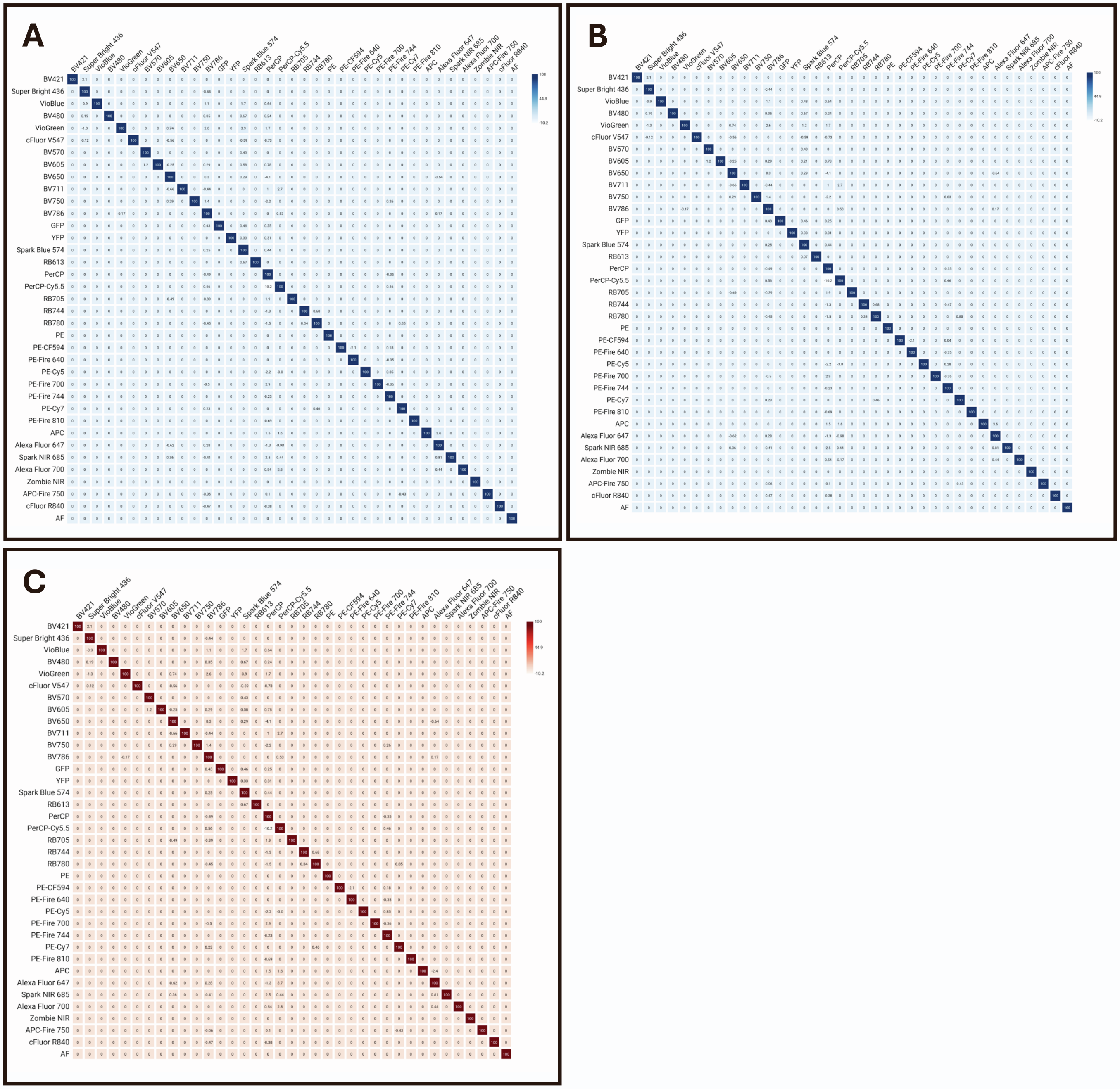

Figure S12. Compensation matrices and spectral spillover assessment.

(**A–C**) Compensation matrices for the 36-color panel applied to samples from independent experiments: (**A**) GLUT1 Experiment 1, (**B**) GLUT1 Experiment 2, and (**C**) GAPDH. Each matrix displays spillover values from individual fluorochromes ("from" columns) into other detectors ("to" rows), where higher values indicate greater unmixing error. For example, in panel A, manual compensation was applied from Alexa Fluor 647 into APC (spillover value: 3.6). Matrices were generated using manual compensation of residual unmixing errors in SpectroFlo.

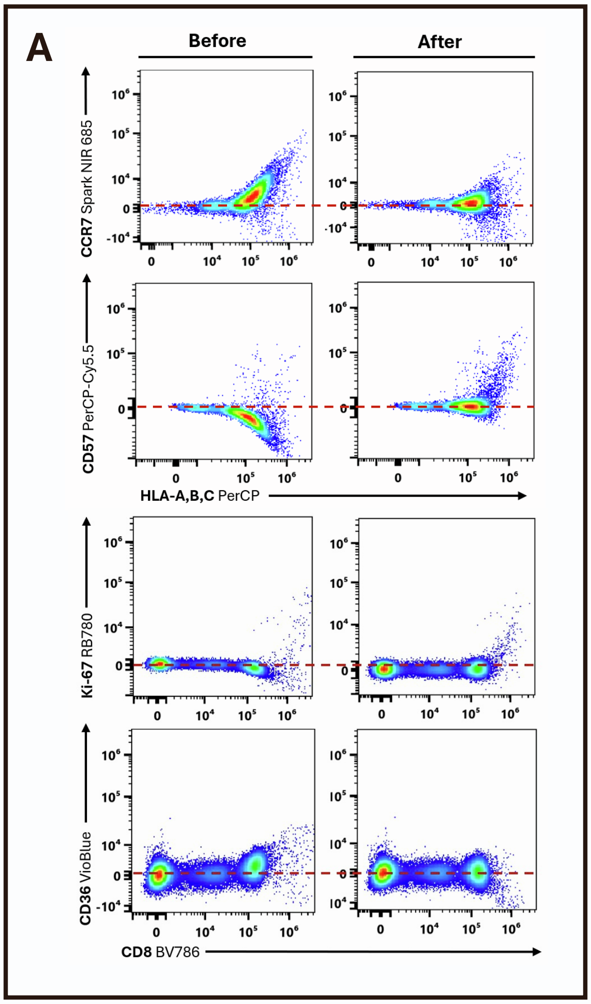

B

Fluorochromes		Correction of Unmixing Errors
From	To	
PerCP	Spark NIR 685	2.5
PerCP	PerCP-Cy5.5	-10.2
BV786	RB780	-0.45
BV786	VioBlue	1.1

Figure S13. Correction of spectral unmixing errors in 36-color panel analysis.

(**A**) Dot plots showing representative examples of signal artifacts caused by unmixing errors before (left) and after (right) manual correction in SpectroFlo. (**B**) Summary table of unmixing corrections applied between specific fluorochrome pairs, with correction values indicating the magnitude of signal adjusted ("From" donor fluorochrome into "To" receiver fluorochrome).

A

Violet (405nm, 100mW)			
Detector Channel	Center Wavelength (nm)	Bandwidth (nm)	Fluorochrome
V1	428	15	*BV421*
V2	443	15	*Super Bright 436*
V3	458	15	*VioBlue*
V4	473	15	
V5	508	20	*BV480*
V6	525	17	
V7	542	17	*VioGreen*
V8	581	19	*BV570 / cFluor V547*
V9	598	20	
V10	615	20	*BV605*
V11	664	27	*BV650*
V12	692	28	
V13	720	29	*BV711*
V14	750	30	*BV750*
V15	780	30	*BV786*
V16	812	34	

Blue (488nm, 50mW)			
Detector Channel	Center Wavelength (nm)	Bandwidth (nm)	Fluorochrome
B1	508	20	*GFP*
B2	525	17	
B3	542	17	*YFP*
B4	581	19	*Spark Blue 574*
B5	598	20	
B6	615	20	*RB613*
B7	661	17	
B8	679	18	*PerCP*
B9	697	19	*PerCP-Cy5.5*
B10	717	20	*RB705*
B11	738	21	
B12	760	23	*RB744*
B13	783	23	
B14	812	34	*RB780*

Yellow-Green (561nm, 50mW)			
Detector Channel	Center Wavelength (nm)	Bandwidth (nm)	Fluorochrome
YG1	577	20	*PE*
YG2	598	20	
YG3	615	20	*PE-CF594*
YG4	661	17	*PE-Fire 640*
YG5	679	18	*PE-Cy5*
YG6	697	19	
YG7	720	29	*PE-Fire 700*
YG8	750	30	*PE-Fire 744*
YG9	780	30	*PE-Cy7*
YG10	812	34	*PE-Fire 810*

Red (640nm, 80mW)			
Detector Channel	Center Wavelength (nm)	Bandwidth (nm)	Fluorochrome
R1	661	17	*APC*
R2	679	18	*Alexa Fluor 647*
R3	697	19	*Spark NIR 685*
R4	717	20	*Alexa Fluor 700*
R5	738	21	
R6	760	23	*Zombie NIR*
R7	783	23	*APC-Fire 750*
R8	812	34	*cFluor R840*

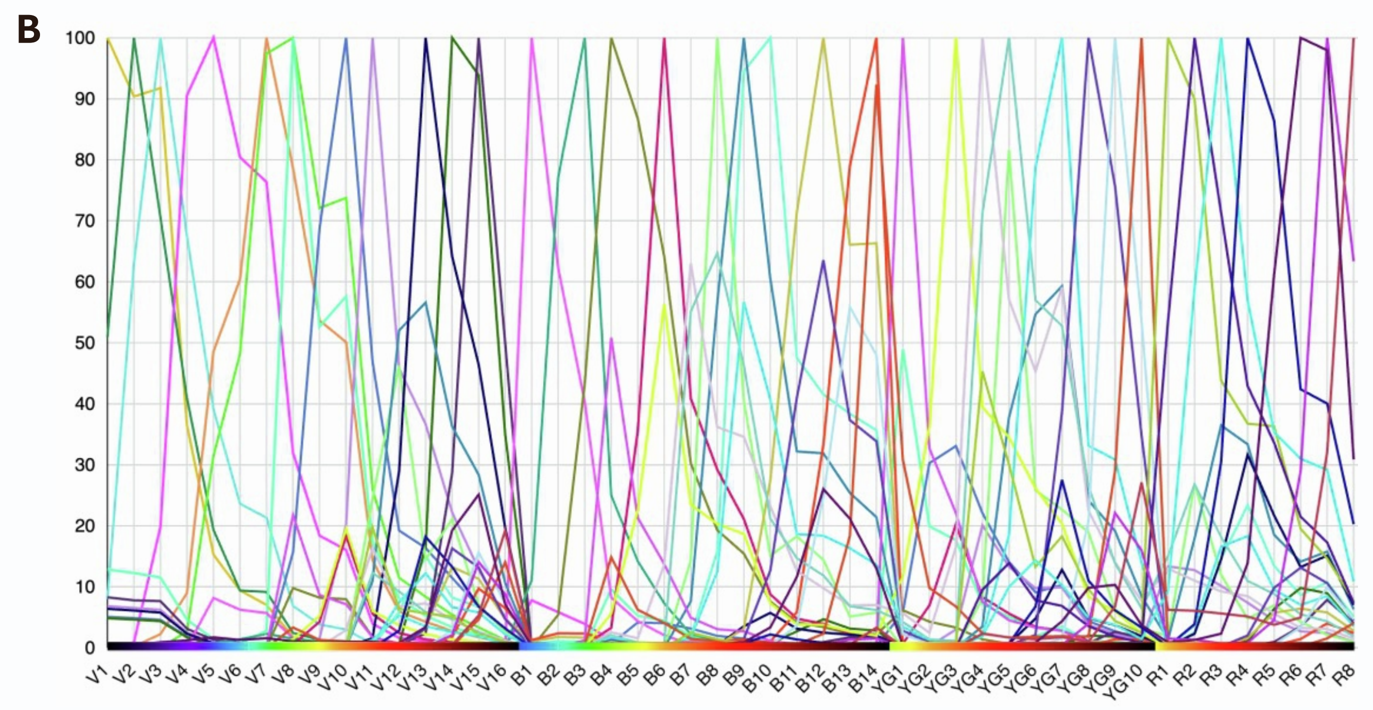

Figure S14. Instrument configuration for 4-Laser Cytek Aurora.

(**A**) Detector channels and bandpass filter specifications for the violet, blue, yellow-green, and red lasers on the Cytek Aurora. Fluorochromes used in the 36-color panel are organized by primary excitation laser and peak detector assignment. BV570 and cFluor V547 are grouped together due to overlapping peak emission in the same detector channel. (**B**) Normalized emission spectra of all 36 fluorochromes, illustrating intensity across detector channels.

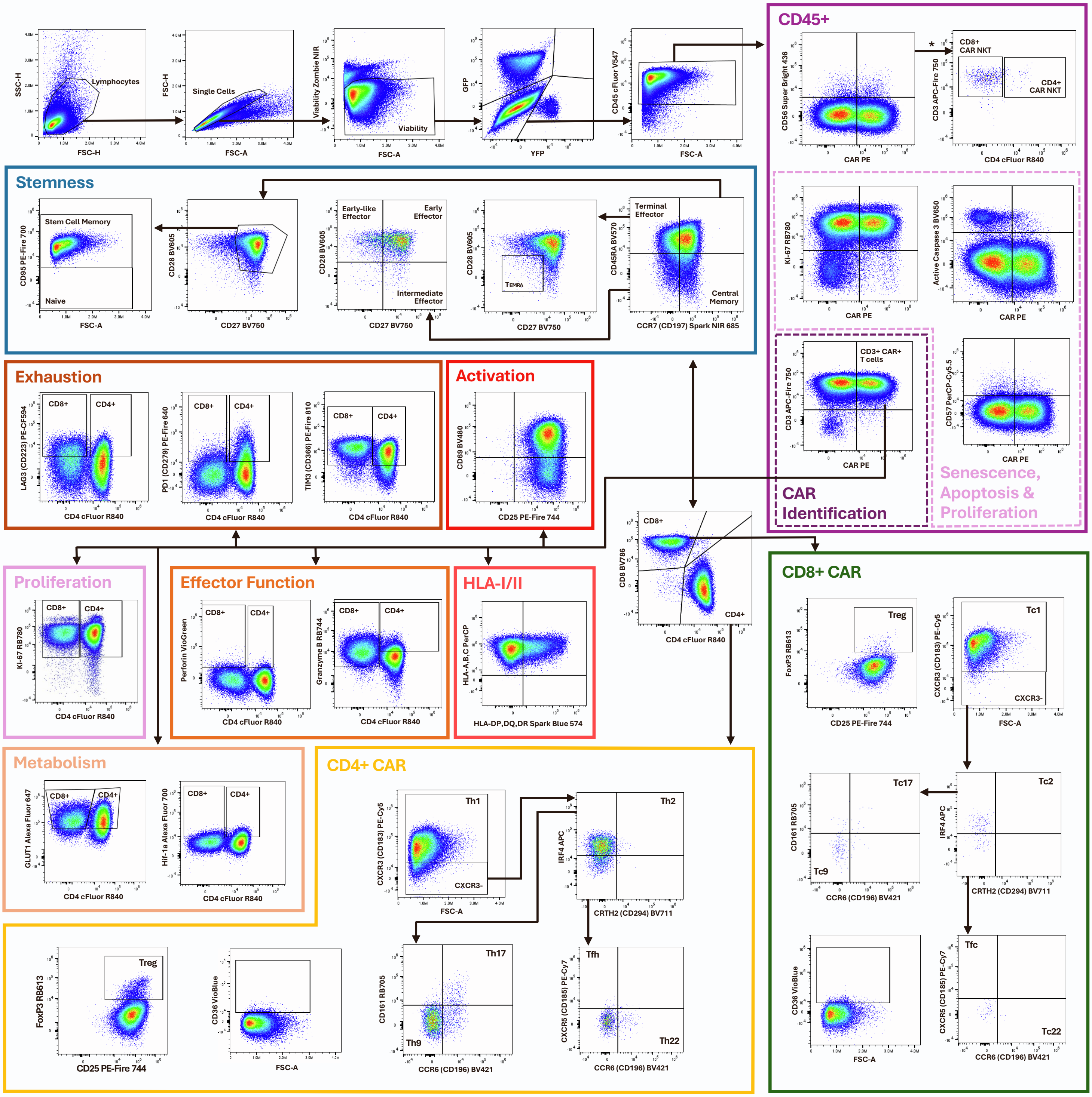

Figure S15. Manual gating strategy for multiparameter phenotypic analysis of CAR T cells.

Marker resolution using the 36-color panel under an exploratory gating strategy (*n* = 1 representative sample). Populations were first gated on FSC, SSC, viability, and CD45, followed by separation based on fluorescent proteins (GFP, YFP) and CD3/CAR expression. Subsequent gating included stemness (T_N, T_{SCM}, T_{CM}, T_{EM}, T_{TE}), exhaustion (LAG3, PD1, TIM3), activation (CD69, CD25), proliferation (Ki-67), effector function (perforin, granzyme B), HLA-I/-II expression, metabolism (GLUT1, Hif-1a), and CD4/CD8 subtypes (Th1, Tc1, Th2, Tc2, Th9, Tc9, Th17, Tc17, Th22, Tc22, Tfh, Tfc, Treg). High-level CD45 gating also included NK-like T cells, Ki-67, active caspase 3, and CD57. *CAR NK T cells and the NxCD4 panel of gates were defined using CD4 vs. CD8 (gate not shown) to exclude $CD4^{-}CD8^{-}$ and $CD4^{+}CD8^{+}$ populations, ensuring accurate representation in the subsequent CD3 vs. CD4 plot.

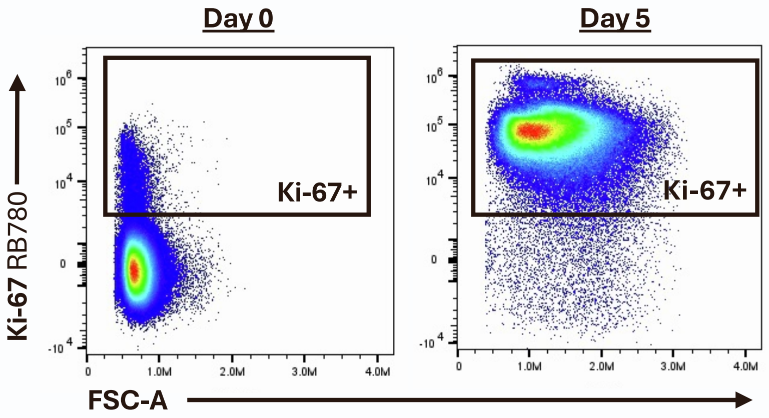

Figure S16. Representative Ki-67$^+$ plots from day 0 and day 5 CAR T cells.

Representative flow cytometry plots showing Ki-67 versus forward scatter area (FSC-A) at day 0 and day 5. At day 0, most CAR T cells are Ki-67$^-$, with negative baseline MFI attributable to spectral unmixing. To facilitate data interpretation, Ki-67 MFI values were calculated from the positively gated population only. Data are from a representative donor (n = 1).

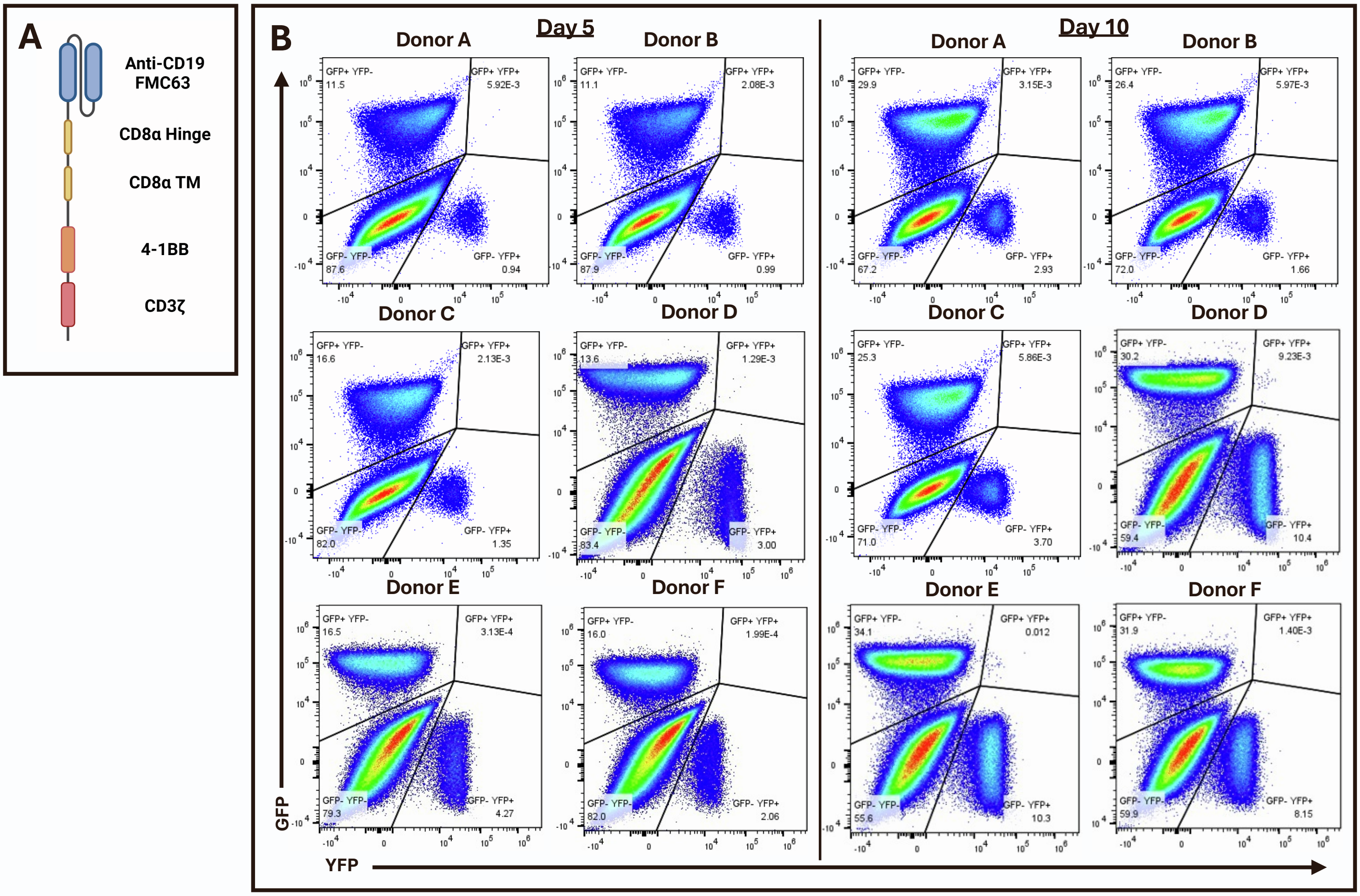

Figure S17. Representative CAR construct and cytotoxicity assay plots.

(**A**) Schematic of the anti-CD19 CAR construct used in this study, composed of the FMC63 single-chain variable fragment (scFv), a CD8α hinge and transmembrane domain, a 4-1BB costimulatory domain, and a CD3ζ signaling domain. (**B**) Representative flow cytometry plots showing cytotoxicity assay results from six healthy donors at day 5 and day 10 post-transduction. CAR T cells were co-cultured for 3 h at a 2:1:1 ratio (Effector : $CD19^{+}$ Nalm6-YFP^{+} target : $CD19^{-}$ Nalm6-GFP^{+} control). YFP:GFP ratios were calculated to assess antigen-specific killing. Each panel represents one donor and one timepoint.

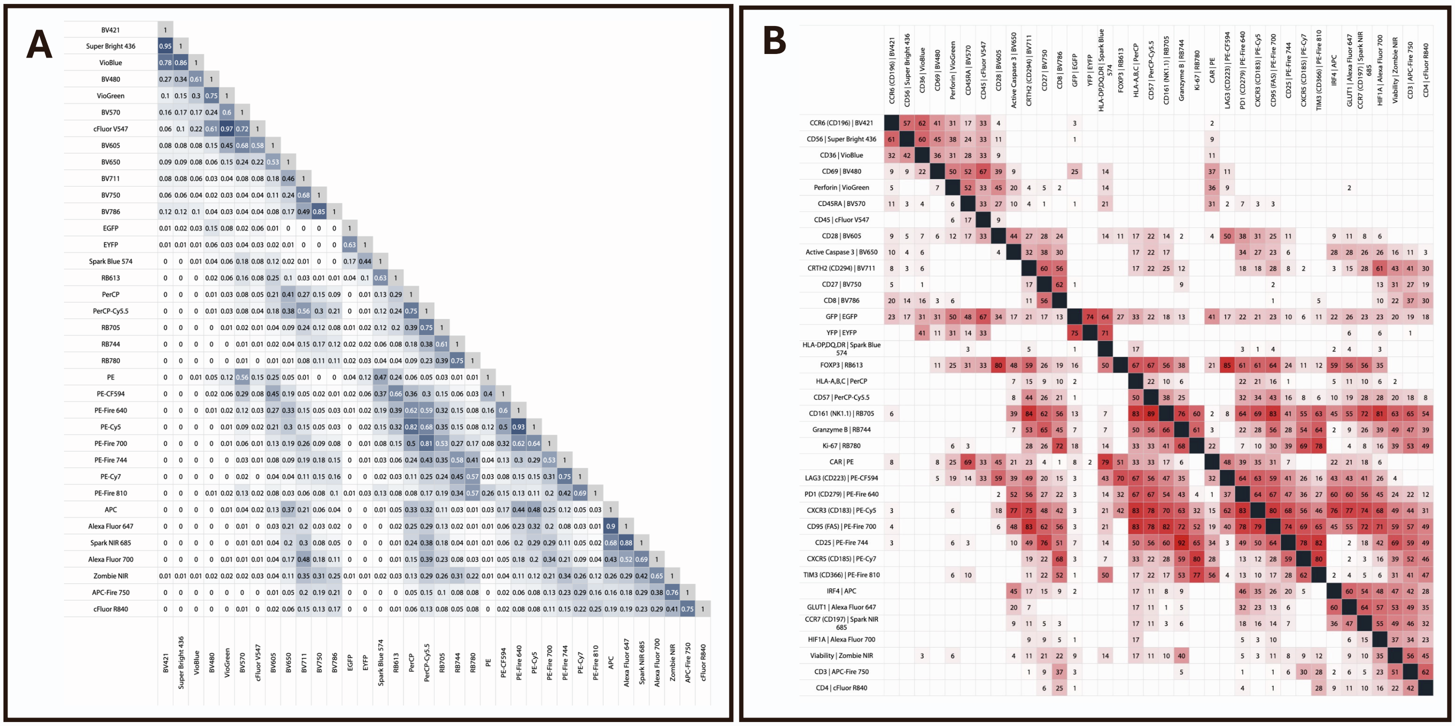

Figure S18. Spectral overlap and marker co-expression matrices.

(**A**) Pairwise similarity index illustrating spectral overlap between fluorochromes in the 36-color panel. Values indicate the degree of spectral overlap. (**B**) Expected reduction in stain index for each fluorochrome (columns) due to signal spread from other fluorochromes (rows). The theoretical stain index reduction is based on the Cytek Cloud library.

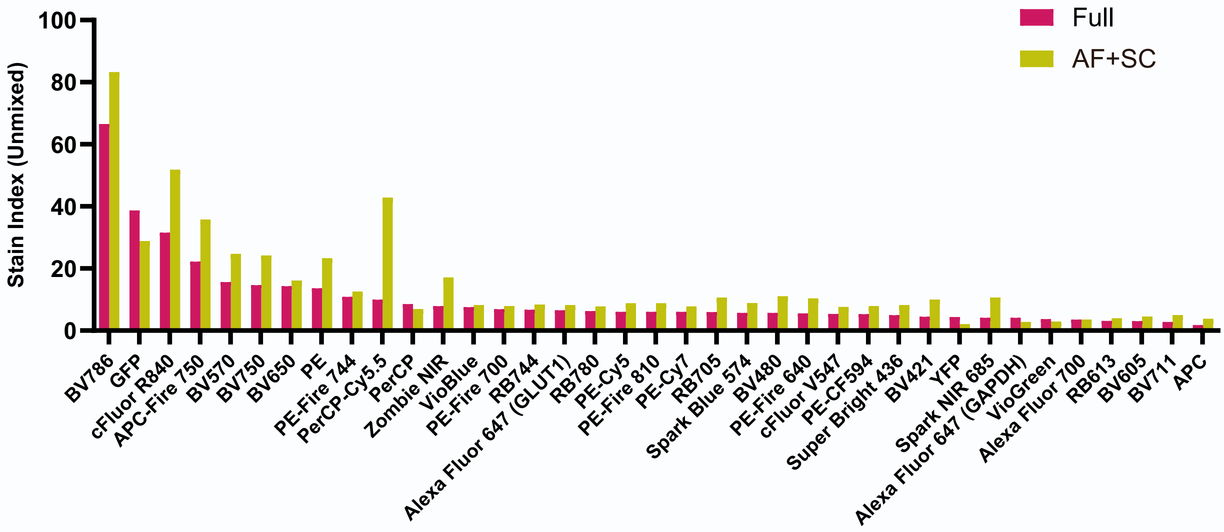

Figure S19. Stain index comparison of single color (SC) controls under full and SC unmixing matrix.

Stain index (SI) values were calculated for single-color (SC) controls when unmixed either with the full 36-marker panel and autofluorescence (“Full,” magenta) or with the individual SC tube and autofluorescence (“AF+SC,” yellow-green). Each bar represents the SI for the respective fluorochrome. Fluorochromes are ranked by SI magnitude under full unmixing conditions.

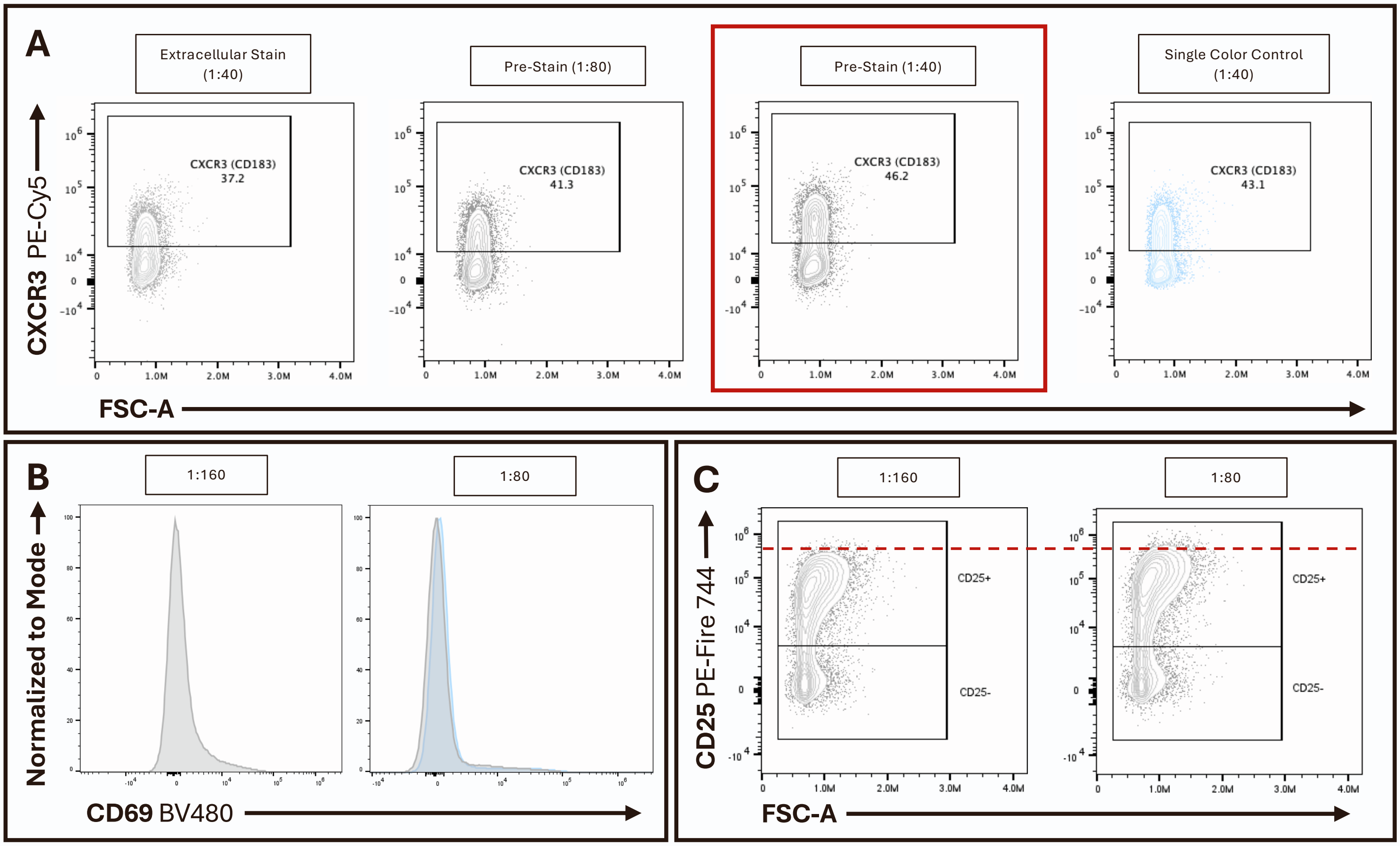

Figure S20. Titration and sequential staining optimization for CXCR3, CD69, and CD25.

(**A**) Resolution of CXCR3 (CD183) was improved by incorporating the antibody into a pre-stain step at its optimal titer (red), separated from the remaining extracellular panel. (**B**) Histogram overlays showing CD69 signal intensity at low (1:160) versus optimal (1:80) antibody titers, demonstrating enhanced resolution at the higher concentration. (**C**) Contour plots showing adjusted titers for CD25. Optimal staining was achieved at 1:160, which maintained marker resolution while reducing overall fluorescence intensity. Ratios above each plot indicate the antibody titer used. Populations in gray and blue represent fully stained and single-color samples, respectively.

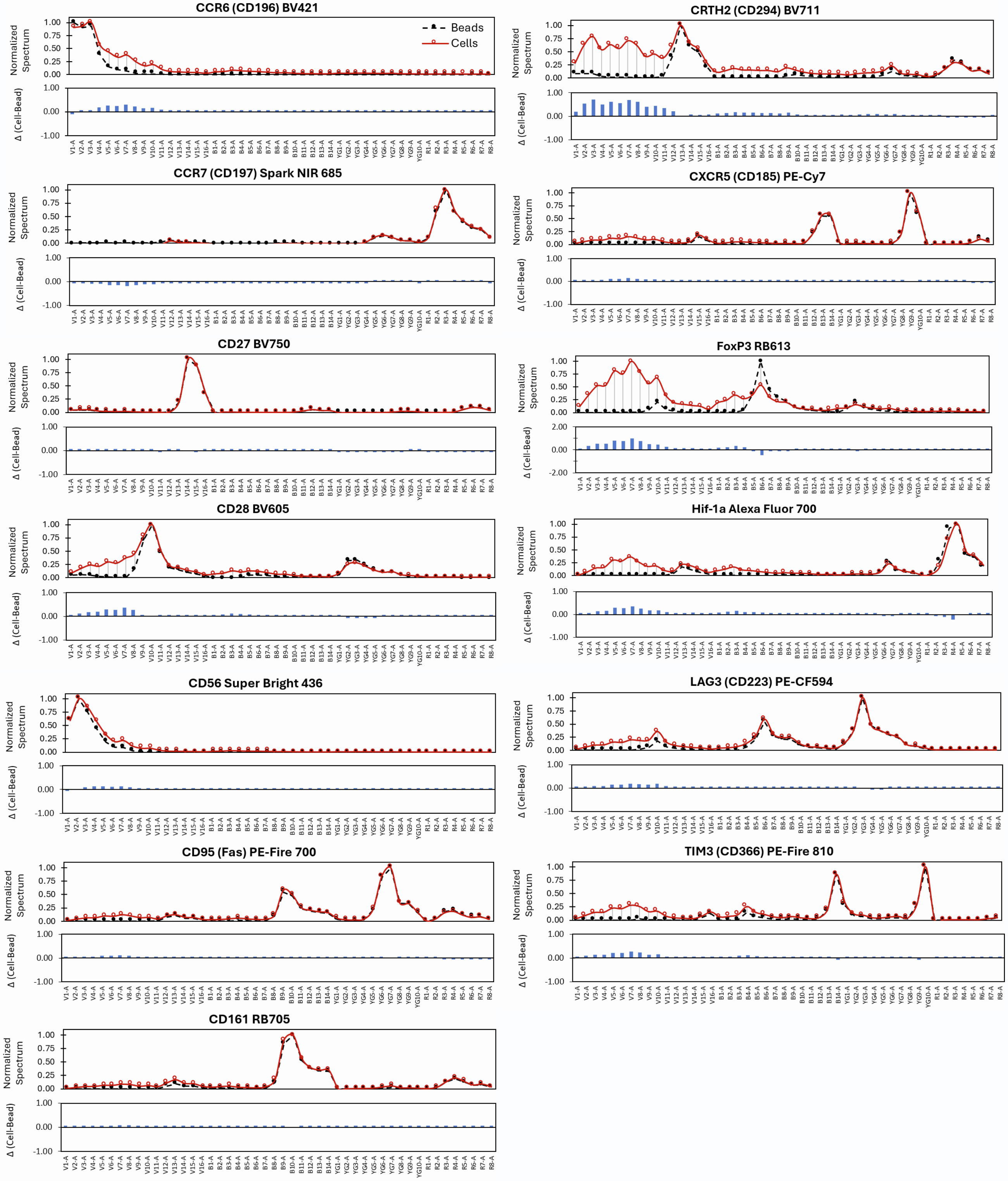

Figure S21. Spectral emission profiles and Δ values for cell- and bead-based reference controls.

Normalized emission spectra for 13 representative markers comparing bead-based (black, dashed) and cell-based (red, solid) reference controls across all 48 detectors. For each marker, the top panel displays normalized signal intensity, while the bottom panel shows the delta (Δ) values representing channel-by-channel differences in emission between cell and bead controls.

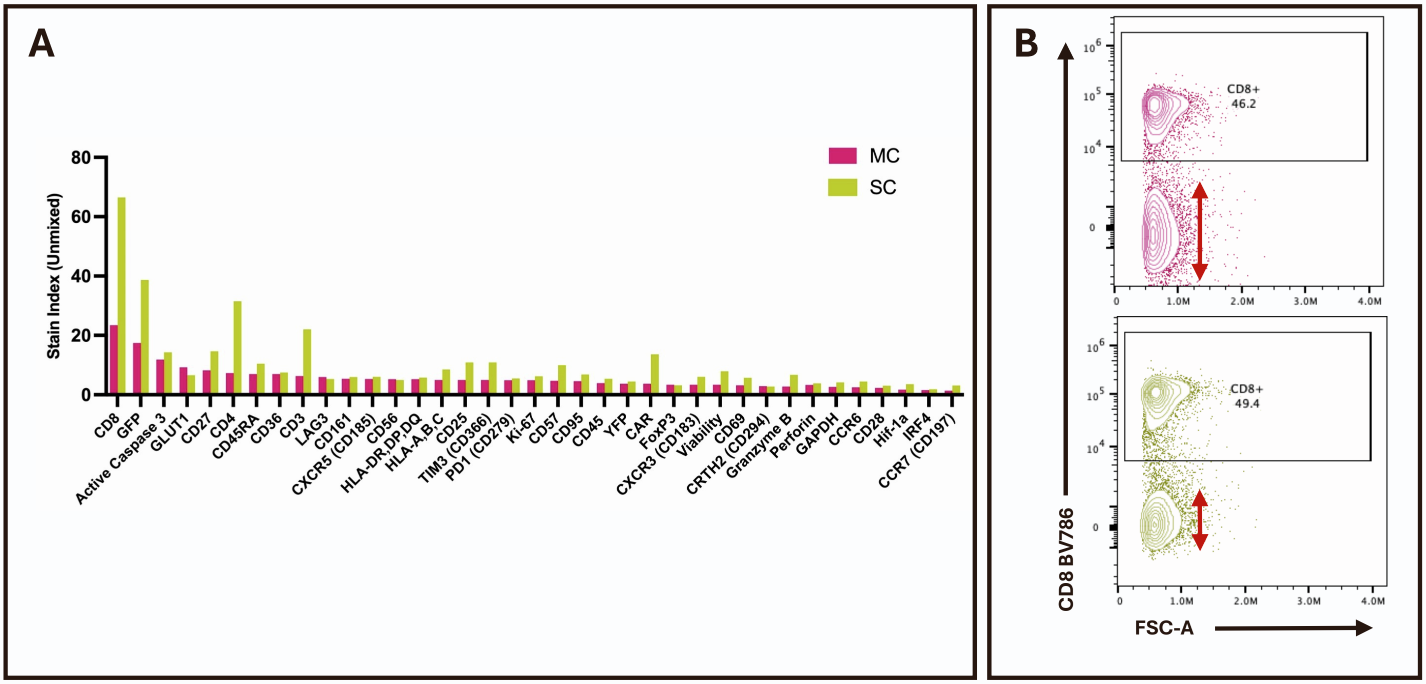

Figure S22. Comparison of stain index and resolution under single-color and multi-color staining conditions.

(**A**) Unmixed stain index (SI) values for all 36 markers under single-color (SC, yellow-green) and full multi-color (MC, magenta) staining conditions. Fluorochromes are ranked by SI magnitude under MC conditions. The greatest SI reductions were observed for CD8, GFP, CD4, and CD3 under MC staining. (**B**) Representative plots showing CD8 resolution in SC versus MC conditions from a representative sample. MC staining resulted in reduced CD8^{+} signal intensity and increased spread of the negative population compared to SC staining.

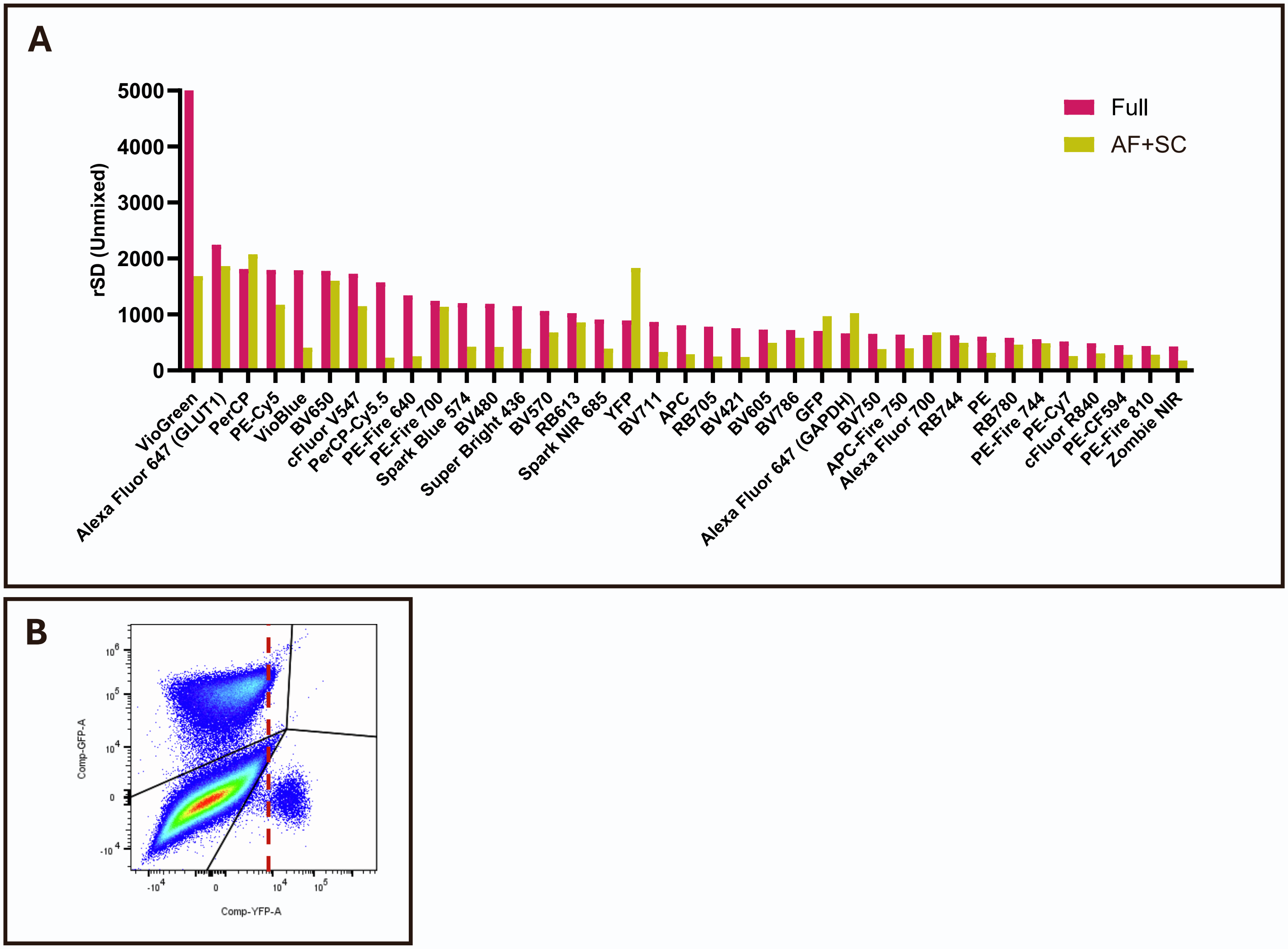

Figure S23. Spreading error of the negative population following spectral unmixing.

(**A**) Robust standard deviation (rSD) was used to quantify spreading error in the negative population after unmixing. rSD values were calculated for each single-color (SC) control when unmixed using either autofluorescence plus SC control (AF+SC, yellow-green) or the full 36-color panel with AF extraction (Full, magenta). Fluorochromes are ranked by rSD magnitude under full unmixing conditions. (**B**) Representative YFP vs. GFP plot from a day 5 CAR T cell co-culture showing unmixing-induced spreading in the $GFP^{-}YFP^{-}$ double-negative gate. This increased spread contributes to higher rSD and gating challenges in the YFP SC control. Biaxial gating of YFP versus GFP improves separation and enables resolution of GFP^{+}, YFP^{+}, and double-negative populations.

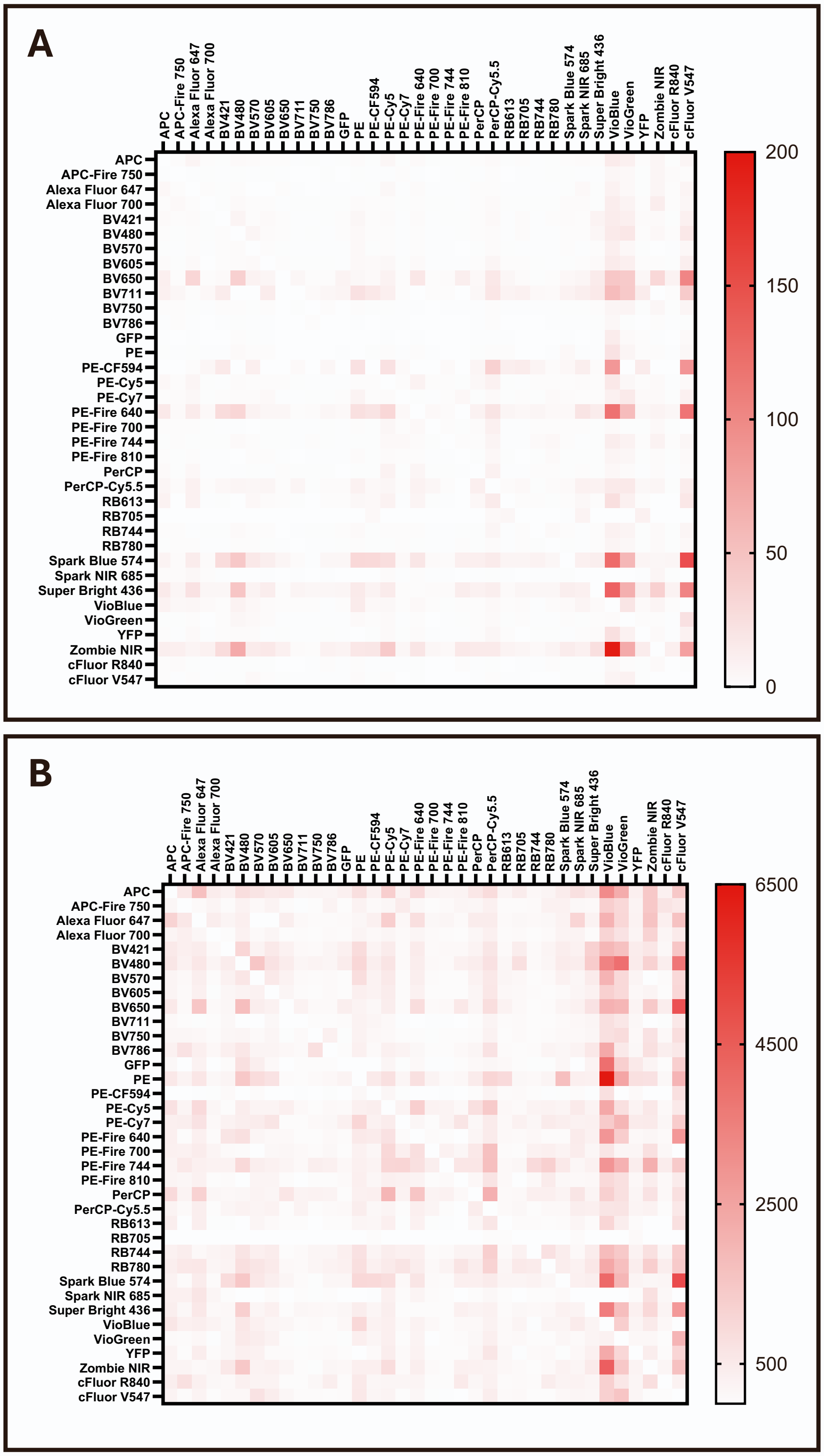

Figure S24. Spillover spreading and total spread matrices for the final 36-color panel.

(**A**) Spillover spreading matrix (SSM) and (**B**) total spread matrix (TSM) calculated from unmixed single-color stained cells for each fluorochrome in the 36-color panel. The SSM identifies potential spreading errors resulting from marker co-expression. The TSM highlights fluorochromes that contribute to spreading error due to spectral overlap or brightness.

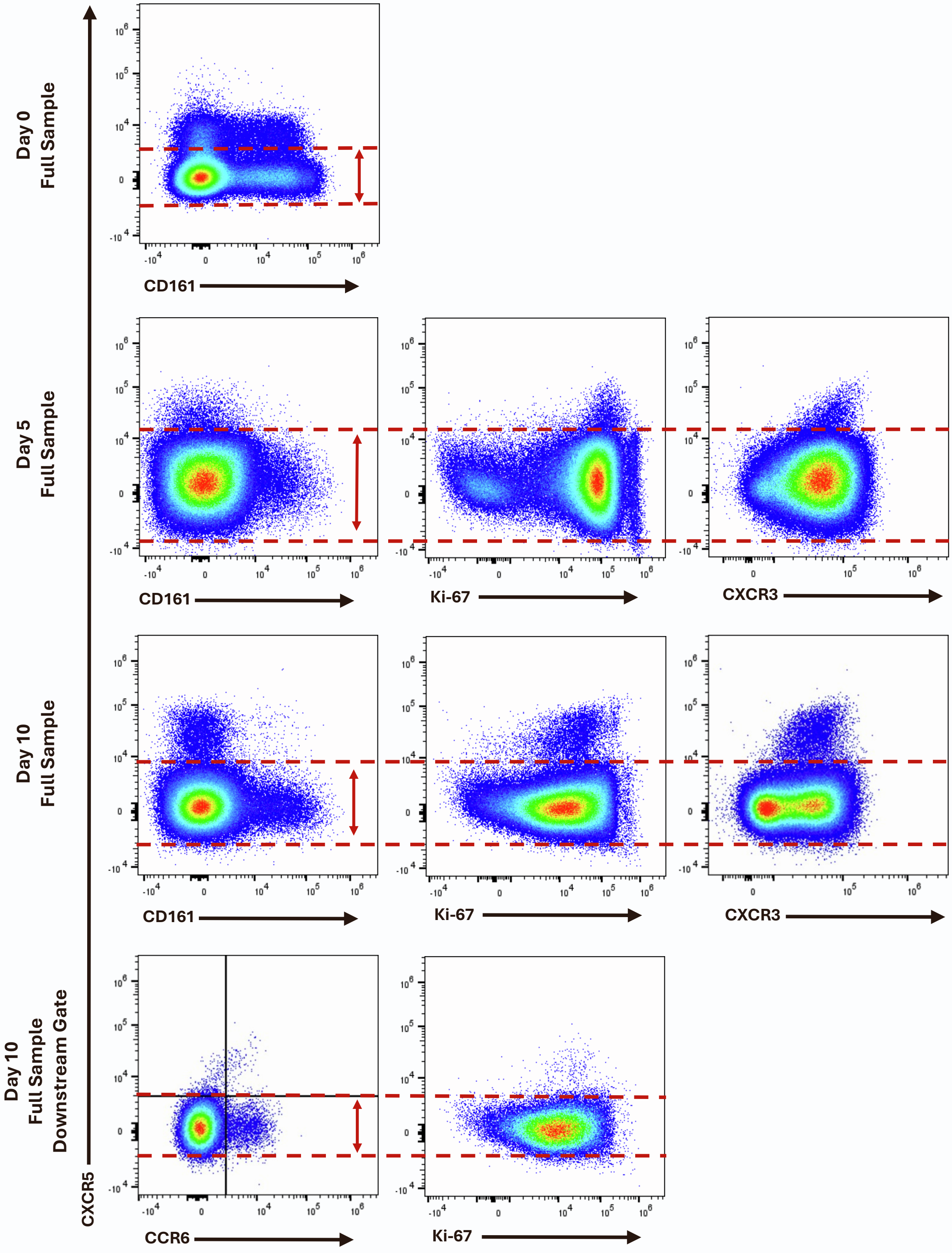

Figure S25. Improved CXCR5 resolution via downstream gating strategy.

CXCR5 resolution across 10 days of manufacturing or co-culture in a representative donor. Rows 1–3 show CXCR5 detection gated from total $CD45^{+}$ cells. Row 4 depicts the same sample gated from a downstream population based on the full gating hierarchy. This approach reduces spectral spread originating from CXCR3 (PE-Cy5), thereby improving CXCR5 (PE-Cy7) resolution.

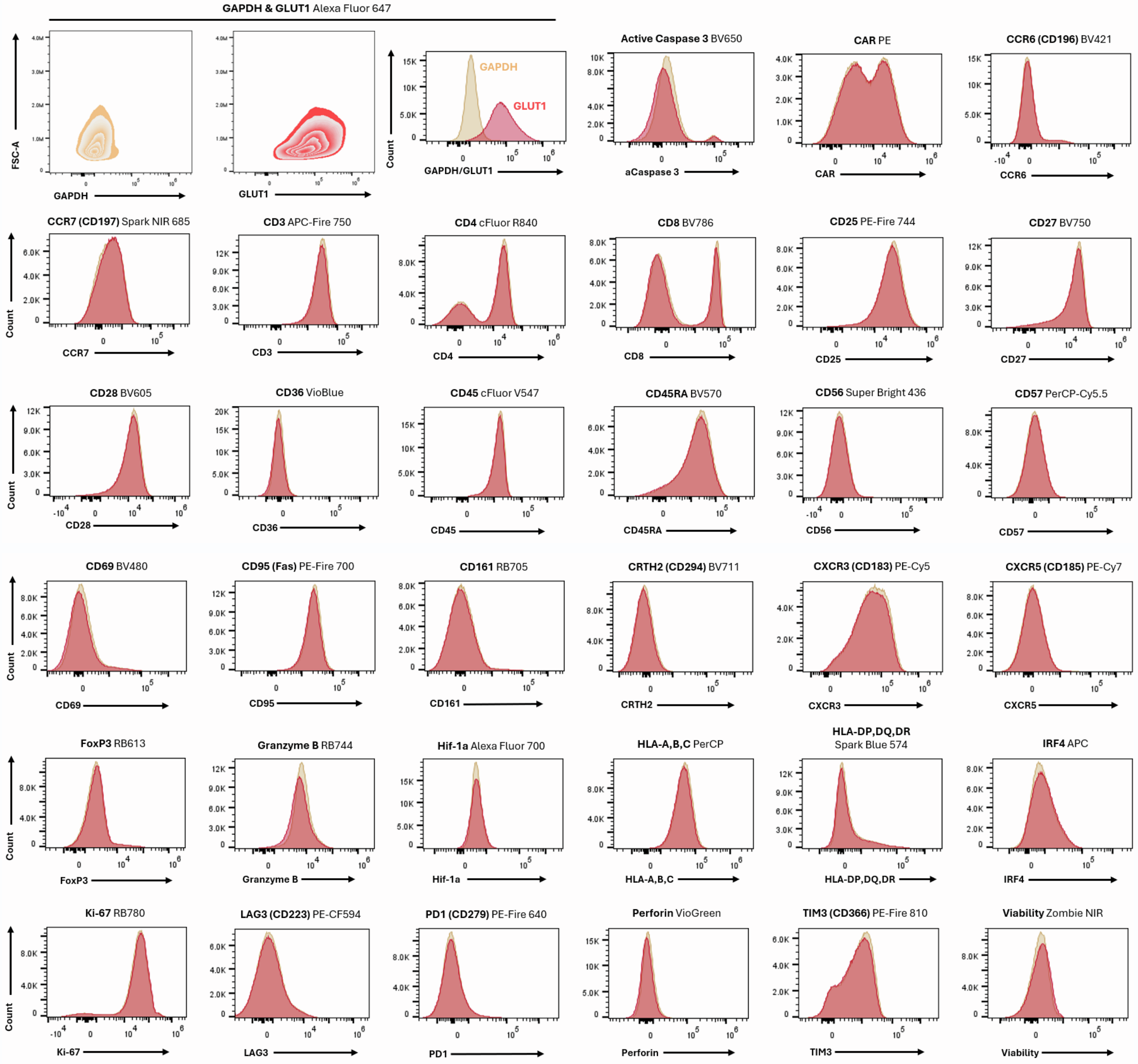

Figure S26. Marker expression profiles using GAPDH or GLUT1 (AF647) in spectral cytometry.

Histogram overlays showing expression of all 36 markers in day 5 CAR^{+} T cells incorporating either GAPDH or GLUT1 conjugated to Alexa Fluor 647 in the fully stained sample. Markers are grouped by functional category and displayed as normalized fluorescence intensity histograms. Data represent fully stained CAR^{+} cells from a single donor (Donor C, day 5 manufacturing). Fluorescence profiles for all markers were acquired under identical staining conditions and gating strategy.

Supplemental References

1. Sadelain, M., Rivière, I. & Brentjens, R. Targeting tumours with genetically enhanced T lymphocytes. *Nat Rev Cancer* **3**, 35–45 (2003).
2. Turtle, C. J. *et al.* CD19 CAR–T cells of defined CD4+:CD8+ composition in adult B cell ALL patients. *Journal of Clinical Investigation* **126**, 2123–2138 (2016).
3. Lohoff, M. *et al.* Dysregulated T helper cell differentiation in the absence of interferon regulatory factor 4. *Proceedings of the National Academy of Sciences* **99**, 11808–11812 (2002).
4. Quigley, M. F., Gonzalez, V. D., Granath, A., Andersson, J. & Sandberg, J. K. CXCR5 + CCR7 – CD8 T cells are early effector memory cells that infiltrate tonsil B cell follicles. *Eur J Immunol* **37**, 3352–3362 (2007).
5. Tsuda, H. *et al.* A novel surface molecule of Th2- and Tc2-type cells, CRTH2 expression on human peripheral and decidual CD4+ and CD8+ T cells during the early stage of pregnancy. *Clin Exp Immunol* **123**, 105–111 (2001).
6. Cosmi, L. *et al.* Human interleukin 17–producing cells originate from a CD161+CD4+ T cell precursor. *J Exp Med* **205**, 1903–1916 (2008).
7. Liu, Y. *et al.* Interleukin-21 induces the differentiation of human Tc22 cells via phosphorylation of signal transducers and activators of transcription. *Immunology* **132**, 540–548 (2011).
8. Good, Z. *et al.* Post-infusion CAR TReg cells identify patients resistant to CD19-CAR therapy. *Nat Med* **28**, 1860–1871 (2022).
9. Lanier, L. L., Testi, R., Bindl, J. & Phillips, J. H. Identity of Leu-19 (CD56) leukocyte differentiation antigen and neural cell adhesion molecule. *J Exp Med* **169**, 2233–2238 (1989).
10. Rufer, N. *et al.* Ex vivo characterization of human CD8+ T subsets with distinct replicative history and partial effector functions. *Blood* **102**, 1779–1787 (2003).
11. D'Ambrosio, D. *et al.* Transcriptional regulation of interleukin-2 gene expression by CD69-generated signals. *Eur J Immunol* **23**, 2993–2997 (1993).
12. Soares, A. *et al.* Novel application of Ki67 to quantify antigen-specific in vitro lymphoproliferation. *J Immunol Methods* **362**, 43–50 (2010).
13. Macintyre, A. N. *et al.* The Glucose Transporter Glut1 Is Selectively Essential for CD4 T Cell Activation and Effector Function. *Cell Metab* **20**, 61–72 (2014).
14. Balmer, M. L. *et al.* Memory CD8 + T Cells Require Increased Concentrations of Acetate Induced by Stress for Optimal Function. *Immunity* **44**, 1312–1324 (2016).
15. Palazon, A. *et al.* An HIF-1α/VEGF-A Axis in Cytotoxic T Cells Regulates Tumor Progression. *Cancer Cell* **32**, 669-683.e5 (2017).
16. Jin, H.-T. *et al.* Cooperation of Tim-3 and PD-1 in CD8 T-cell exhaustion during chronic viral infection. *Proceedings of the National Academy of Sciences* **107**, 14733–14738 (2010).
17. Ma, X. *et al.* CD36-mediated ferroptosis dampens intratumoral CD8+ T cell effector function and impairs their antitumor ability. *Cell Metab* **33**, 1001-1012.e5 (2021).
18. Brenchley, J. M. *et al.* Expression of CD57 defines replicative senescence and antigen-induced apoptotic death of CD8+ T cells. *Blood* **101**, 2711–2720 (2003).
19. Porter, A. G. & Jänicke, R. U. Emerging roles of caspase-3 in apoptosis. *Cell Death Differ* **6**, 99–104 (1999).
20. Hermans, I. F. *et al.* The VITAL assay: a versatile fluorometric technique for assessing CTL- and NKT-mediated cytotoxicity against multiple targets in vitro and in vivo. *J Immunol Methods* **285**, 25–40 (2004).
21. Depil, S., Duchateau, P., Grupp, S. A., Mufti, G. & Poirot, L. 'Off-the-shelf' allogeneic CAR T cells: development and challenges. *Nat Rev Drug Discov* **19**, 185–199 (2020).
